# Cyclization of interlocked fumaramides into β-lactams: experimental and computational mechanistic assessment of the key intercomponent proton transfer and the stereocontrolling active pocket†

**DOI:** 10.1039/d0sc05757f

**Published:** 2020-11-11

**Authors:** Alberto Martinez-Cuezva, Aurelia Pastor, Marta Marin-Luna, Carmen Diaz-Marin, Delia Bautista, Mateo Alajarin, Jose Berna

**Affiliations:** Department of Organic Chemistry, Faculty of Chemistry, University of Murcia, Regional Campus of International Excellence “Campus Mare Nostrum” 30100 Murcia Spain amcuezva@um.es aureliap@um.es ppberna@um.es; ACTI, University of Murcia 30100 Murcia Spain

## Abstract

A detailed mechanistic study of the diastereoselective CsOH-promoted cyclization of interlocked fumaramides to give β-lactams is described. The mechanistic analysis comprises the experimental evaluation of the structure-reactivity relationship for a wide range of fumaramides [2]rotaxanes (Hammet-plots), KIE studies with deuterium-labelled interlocked fumaramides and computational analysis of two alternative mechanistic pathways for the cyclization process. The obtained results confirm that: (a) the rate-determining step is the deprotonation of the *N*-benzyl group of the thread by the amidate group of the macrocycle generated by the external base, (b) the polyamide macrocycle plays an important role not only as activating element but also as the stereodifferenciating factor responsible for the observed diastereoselection and (c) the higher flexibility of the adamantyl core speeds up the cyclization process in diadamantyl-derived rotaxanes.

## Abstract 2

A mechanistic study of the diastereoselective cyclization of interlocked fumaramides to give β-lactams unveils the key factors for successfully taming the process.

## Graphical abstract

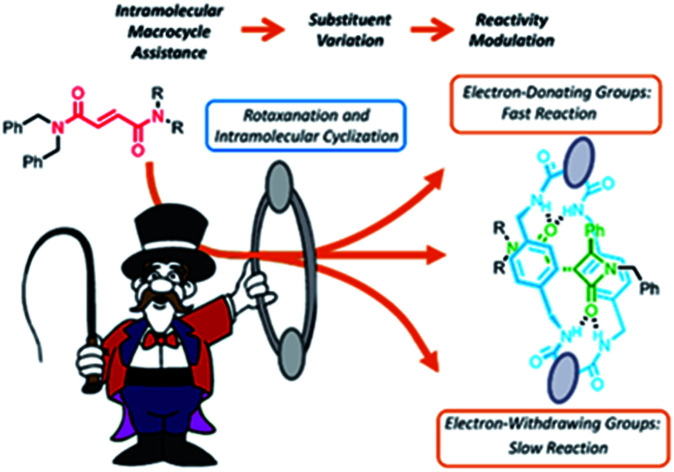

## 1. Introduction

The discovery and development of catalytic systems is one of the most captivating challenges in chemistry.^1,2^ Enzymes, the most efficient known catalysts, control a vast number of biochemical processes occurring in living organisms.^3^ The high specificity exhibited by enzymes relies on their complex three-dimensional structures, where the active site is located.^4–7^ In recent years, the development of artificial switchable catalysts has been the object of deep study. These synthetic systems are able to control the rate, reaction pathway or stereochemical outcome of a chemical process, triggered by a wide range of different external stimuli.^8–15^ Interlocked molecules have become ideal candidates for this purpose due to their unique properties derived from the presence of a mechanical bond^16,17^ giving rise to a series of selective and/or stimuli-responsive catalysts.^18–38^

In most of the reported examples of switchable rotaxane-based catalysts, the well-defined position of the macrocycle along the thread allows to control the reaction rate, usually inhibiting the catalysis when the active site is shielded by the macrocycle.^39–44^ A rare exception to this rule, is the recent example described by Goldup and coworkers in which a macrocycle actively participates in a reaction occurring inside its cavity. In this particular case, the functionality of the thread is dramatically altered by being prepared within the macrocycle.^45^

**Scheme 1:**
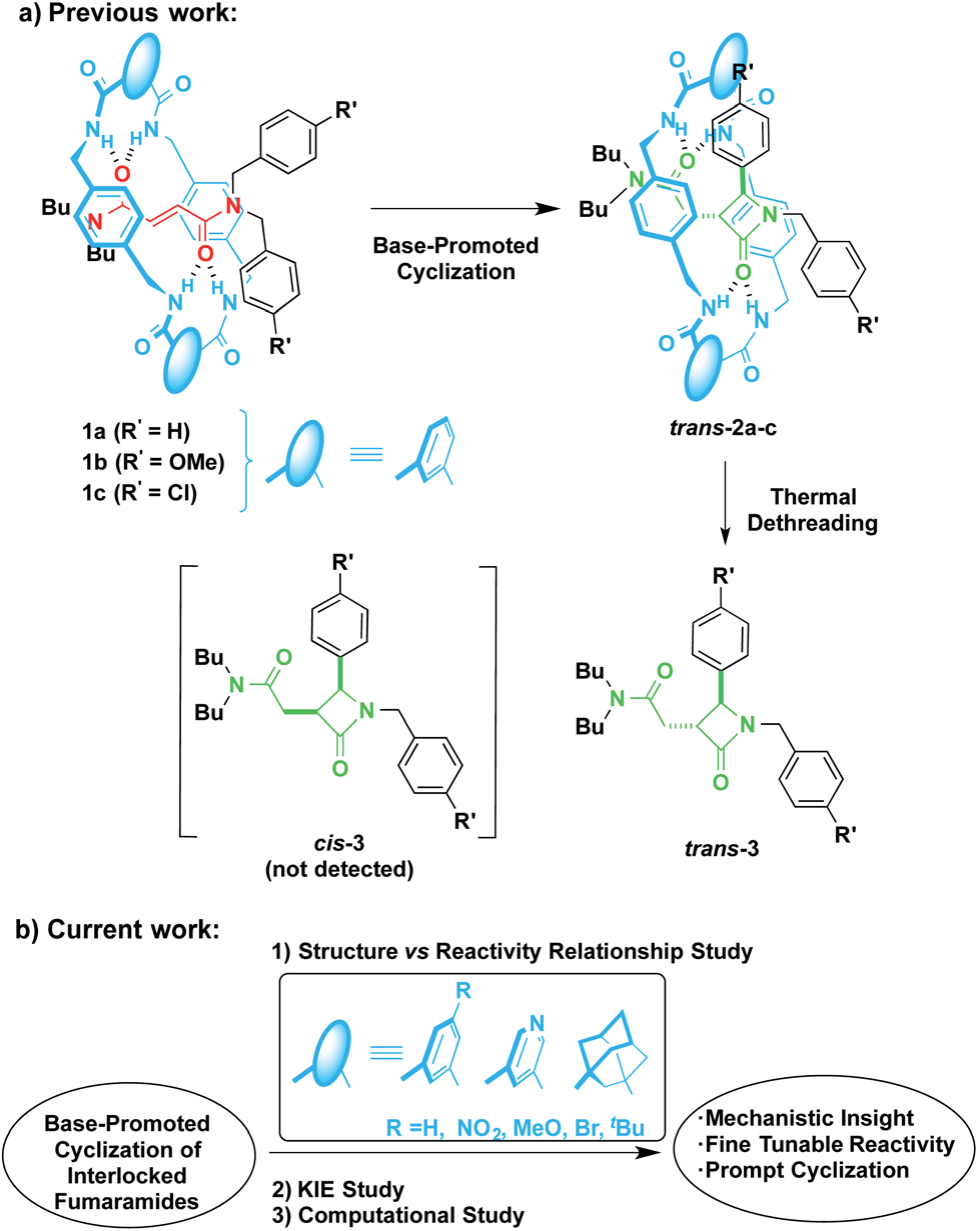
Base-promoted intramolecular cyclization of interlocked *N*,*N*-dibenzylfumaramides 1: (a) previously reported work and (b) novel results included herein.

In 2016 we reported the CsOH-promoted intramolecular cyclization of archetype *N*-benzylfumaramides 1a–c embedded in the isophthalamide-based ring of a [2]rotaxane (Scheme 1, part (a)).^46^ In these processes, the corresponding interlocked β-lactams *trans*-2a–c were isolated in almost quantitative yields as single diastereoisomers. Note that the cyclization of the noninterlocked fumaramide in the presence of CsOH (not shown) leads to a *cis*–*trans* mixture in poor yield. On the basis of these results, we assumed that the interlocked polyamide macrocycle must play a definitive activating role, making another exception to the rule of interlocked systems in which the macrocycle-effect alters the reaction outcomes. Besides, the macrocycle exerts a protective role once the cyclization step has taken place. As a result, the corresponding β-lactams *trans*-2a–c are isolated in almost quantitative yields in spite of their high instability in basic media. As a bonus, the extrusion of the chiral azetidinone threads (*trans*-3 in Scheme 1) through a thermal dethreading^47–49^ completes an unusual method to obtain high value-added β-lactams.^50,51^ By following a similar protocol we also described a related cyclization of enantiopure interlocked *N*-α-methylbenzylfumaramides, giving rise to enantioenriched *trans*-β-lactams bearing a chiral quaternary atom.^52^

Despite the CsOH-promoted cyclization of interlocked fumaramides has turned out an interesting tool for the diastereoselective synthesis of β-lactams, the little knowledge about the mechanism hinders further applications. With the aim of inspiring further research, new insights of the cyclization mode of [2]rotaxanes 1 are necessary to lead to a fine tuneable design of our interlocked molecules without compromising their structural integrity and expand the synthesis to more complex β-lactams.

Herein, we disclose the unravelling of the mechanism of the base-promoted cyclization of interlocked fumaramides 1 through an exhaustive inspection tackled from three different but complementary perspectives. First, a range of novel fumaramide [2]rotaxanes 1d–i (Table 1) differing in the structure and electronic features of the macrocyclic counterpart were synthesised to conduct a detailed structure-reactivity relationship analysis (Scheme 1, part (b)). This piece of research has revealed that diadamantyl-derived rotaxanes sparks the cyclization process, being possible to further enhance the scope of this transformation. Second, the KIE with deuterium-labelled interlocked fumaramides was evaluated to obtain some hints about the rate-determining step of the cyclization process. Third, despite the fact that computational analysis at DFT level of interlocked molecules is challenging, we have conducted computational mechanistic studies, including the computing of the possible intermediates, in order to gain a more detailed understanding of how interactions within the rotaxane structure might influence the course of the cyclization and the sense of diastereocontrol. The global overview of the mechanism described herein shows a detailed picture of the sequence of events at the molecular level, which allows the assessment of a intercomponent proton transfer and the stereocontrolling active pocket inside the macrocyclic counterpart as key features of these processes.

**Table 1:** Synthesis of interlocked fumaramides 1a, 1d–i^a^

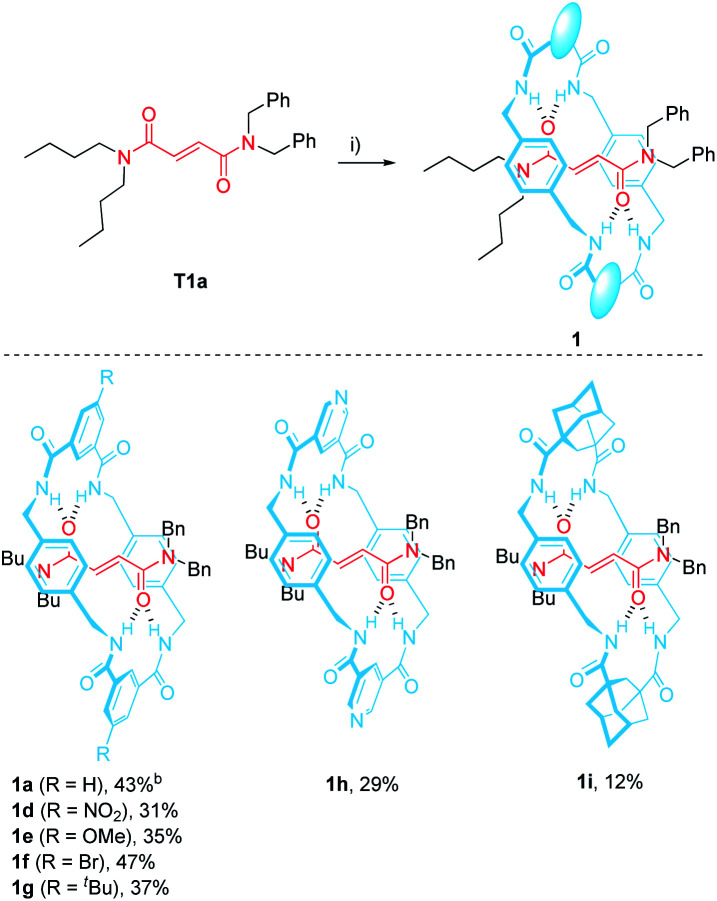

a Reaction conditions: (i) *p*-xylylenediamine, diacyl chloride, Et_3_N, CHCl_3_, 25 °C, 4 h.

b Reported in a previous reference.^46^

## 2. Results and discussion

### 2.1 Synthesis of the *N*-benzylfumaramide-based [2]rotaxanes 1d–i

The *N*,*N*-dibenzylfumaramide [2]rotaxanes 1d–i were prepared following a well-established methodology^53–55^ starting from *N*,*N*-dibenzyl-*N*′,*N*′-dibutylfumaramide T1a as the effective template already utilized in the synthesis of 1a.^46^ Accordingly, [2]rotaxanes 1d–i bearing different polyamide rings were assembled through a five-component reaction starting from T1a, *p*-xylylenediamine and the appropriate diacyl chloride in the presence of Et_3_N (Table 1). The interlocked fumaramides 1d–h were obtained in yields ranging 29–47%. However, the synthesis of the adamantane-derived rotaxane 1i took place in a significantly reduced yield (12%). The low yield in 1i could be tentatively explained in terms of the lower acidity of the NH hydrogen atoms of the transient precursors of the macrocycle, thus decreasing the stability of the supramolecular complex previous to the final interlocked structure.^48,56^

### 2.2 Synthesis of the interlocked β-lactams *trans*-2d–i

The optimal reaction conditions for quantitively converting rotaxane 1a into the interlocked β-lactam *trans*-2a had been already settled in our previous studies (25 mM, 1 equiv. of CsOH, DMF, 25 °C, 12 h).^46^ Unfortunately, we found that under the same reaction conditions the competitive dethreading of the fumaramide containing rotaxanes 1d (R = NO_2_), 1f (R = Br) and 1h (pyridine core) was also taking place in an important extension, affording increasing amounts of T1a and the corresponding macrocycle. The ability of related interlocked succinamide derivatives with electron-withdrawing groups at the macrocycle to experience a fast dethreading process has been earlier reported.^47^ For this reason, the dethreading of rotaxanes 1d–i in DMF-*d*_7_ at 25 °C was monitored by ^1^H NMR spectroscopy (see ESI for further details†) founding a half-life time 0.7 h for 1d (R = NO_2_), which showed to be the most prone to undergo the disassembly process. By contrast, this parameter increased until 190 h in the case of 1g (R = ^*t*^Bu). The diadamantyl-derived 1i did not show any dissociation after long periods of time.

Due to this competitive pathway, the reaction conditions for the cyclization of rotaxanes 1d–i needed to be individually optimized (Table 2). All the experiments were carried out at a 25 mM concentration of 1 except for the cyclization of 1g (R = ^*t*^Bu), which was conducted at 10 mM as a consequence of its lower solubility. Rotaxanes having electron-poor macrocycles such as 1d (R = NO_2_), 1f (R = Br) and 1h (pyridine core) demanded longer reaction times (24–48 h). In fact, the reaction of 1d was extremely slow in the presence of 1 equiv. of CsOH, thus favouring the dethreading process. For these rotaxanes the presence of an excess of base (3 equiv.) was mandatory not only to reach good conversions but, more importantly, to avoid the undesired dethreading process. The cyclization worked out faster with rotaxanes 1g (R = ^*t*^Bu, 10 h) and 1i (adamantane core, 1 h). All the interlocked lactams *trans*-2d–i were delightfully isolated as single diastereoisomers regardless of the structure of the macrocycle.

**Table 2:** Base-promoted cyclization of rotaxanes 1a, 1d–i^a^

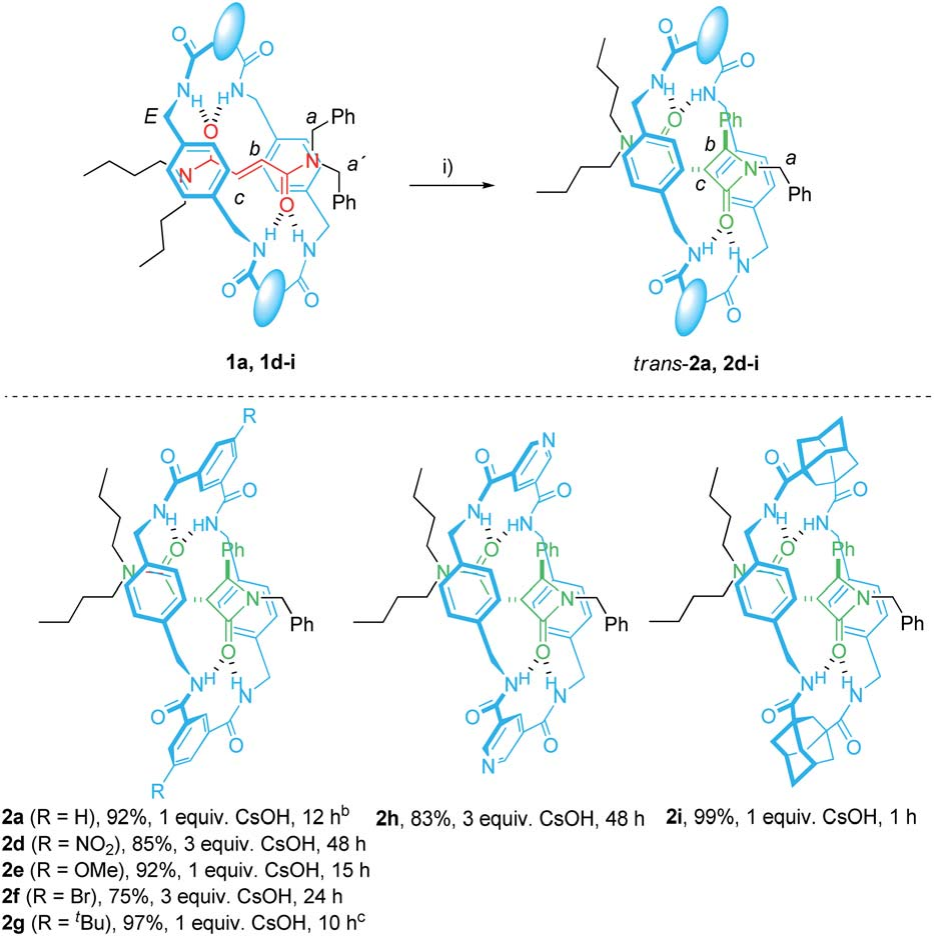

a Reaction conditions: (i) CsOH (1–3 equiv.), DMF (25 mM), 25 °C.

b Reported in a previous reference.^46^

c Reaction conducted under diluted conditions (10 mM).

### 2.3 Structure-reactivity relationship study of the cyclization of interlocked *N*-benzylfumaramides 1a–i: Hammett plots

**Fig. 1:**
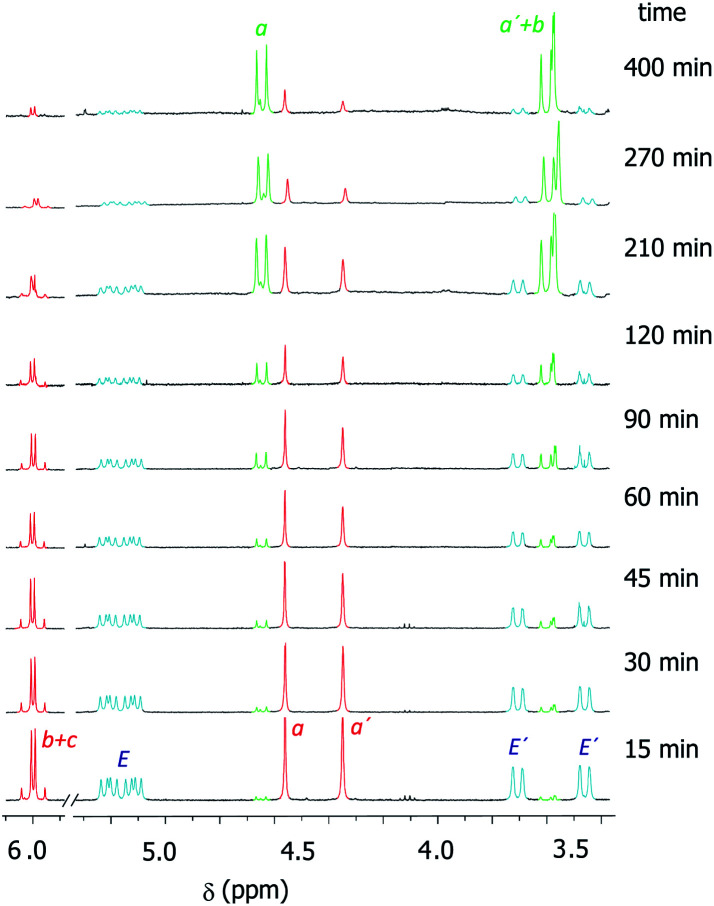
Kinetic experiment of the cyclization of 1a (DMF, 1 equiv. CsOH, 25 °C, 25 mM). Signals referred to 1a are in red (thread) and light blue (macrocycle). Signals of the forming lactam *trans*-2a are in green. For lettering see Table 2. Note that the signals referred to the macrocycle (E and E′) in *trans*-2a appeared as very broaden peaks, being difficult their observation at room temperature (see ESI for further details†).

**Fig. 2:**
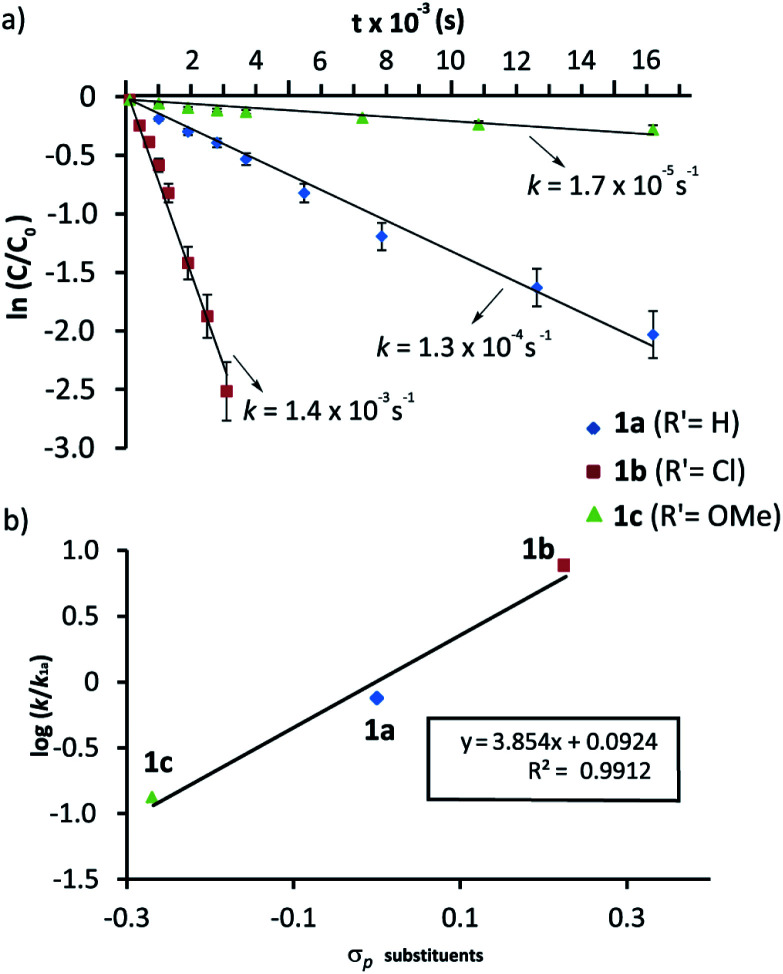
Cyclization of rotaxanes 1a–c in the presence of CsOH (1 equiv.) at 25 °C in DMF: (a) Plots of ln(*c*/*c*_o_) *versus* time for the determination of the rate constants; (b) Hammett plot of log(*k*/*k*_1a_) against the substrate electronics (*σ*_*p*_). The rate constant values are the average of two independent measurements.

We had previously observed that electron-withdrawing groups attached to the *para* position of the *N*-benzyl groups of the fumaramide thread accelerated the cyclization (Scheme 1).^46^ With the aim of expressing quantitatively such structure-reactivity relationship, we monitored the CsOH-promoted cyclization of rotaxanes 1a–c by ^1^H NMR spectroscopy. The cyclization of 1a in the presence of CsOH (1 equiv.) at 25 °C in DMF is illustrated in the stack plot of ^1^H NMR spectra depicted in Fig. 1. As the reaction takes place, signals due to *trans*-2a emerged (highlighted in green, for lettering see Table 2), whereas the signals of the interlocked fumaramide 1a (marked in red and blue) diminished. Next, similar experiments with [2]rotaxanes 1b,c bearing Cl and MeO substituents at *para* position of both *N*-benzyl groups, were carried out. After integrating the specific resonances attributable to both reactant and reaction product and fitting the data to a first order equation, we calculated the respective rate constants (*k*) (Fig. 2a, for further details see ESI†). The Hammett plot of log(*k*/*k*_1a_) against the *σ*_*p*_ constants of the X substituents at the benzylic groups of the fumaramide moiety clearly demonstrates the linearity of the correlation with *ρ* = +3.85 (*R*^2^ = 0.991) (Fig. 2b).

**Fig. 3:**
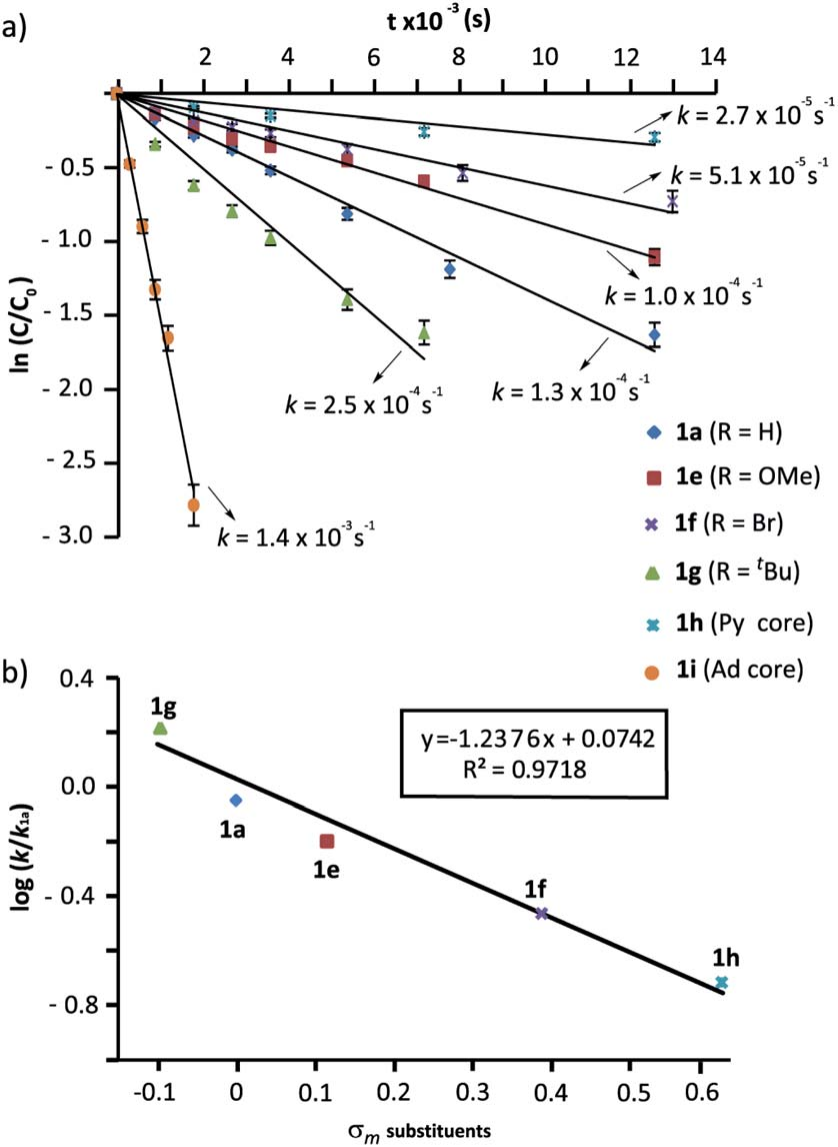
Cyclization of rotaxanes 1a and 1e–i in the presence of CsOH (1 equiv.) at 25 °C in DMF: (a) Plots of ln(*c*/*c*_o_) *versus* time for the determination of the rate constants; (b) Hammett plot of log(*k*/*k*_1a_) against the substrate electronics (*σ*_*m*_). The rate constant values are the average of two independent measurements.

We next evaluated the influence of the rings at the macrocycle (*m*-substituted benzene, pyridine, adamantane) on the reaction rate. The conversions of the rotaxanes 1e–h into the interlocked β-lactams *trans*-2e–i were monitored by ^1^H NMR, and their respective rate constants (*k*) estimated by fitting of the data to a first order equation (Fig. 3a, further details in the ESI†). All the experiments were carried out at a 25 mM concentration of 1 and with 1 equiv. of base, except for the cyclization of 1g (R = ^*t*^Bu), which was conducted at 10 mM due to its lower solubility.^57^ Under these reaction conditions the reaction of the 5-nitro-substituted rotaxane 1d was extremely slow favouring the dethreading process. Therefore, 1d was excluded from this study.

As alluded to above, the kinetic data revealed that electron-withdrawing groups at the benzene rings of the macrocycle decelerate the process (Fig. 3a). Thus, poor conversions were observed for the bromo-substituted rotaxane 1f and the dipyridyl rotaxane 1h after 8 h. Additionally, an appreciable amount of dethreaded T1a was observed, which increased over time. Rotaxane 1e, with a methoxy group, reacted slightly slower than the model substrate 1a. In contrast, the rate of converting rotaxane 1g, with an electron-releasing group (^*t*^Bu), was higher. Interestingly, the reaction of the diadamantyl rotaxane 1i experienced a 10-fold rate enhancement compared to that of the reference compound 1a (*k*_1a_ = 1.3 × 10^−4^ s^−1^*versus k*_1i_ = 1.4 × 10^−3^ s^−1^, Fig. 3a). Plots of log(*k*/*k*_1a_) *versus* the *σ*_*m*_ constants of the groups at the 5-position of the isophthaloyl group are shown in Fig. 3b. The data clearly fitted to a linear correlation with a negative value of *ρ* = −1.24 (*R*^2^ = 0.972).

In a nutshell, the analysis of the Hammett-plots shows that electron-releasing groups at the macrocycle speed the formation of the interlocked β-lactams (*ρ* = −1.24) indicating that the latter substructure takes an active part in the transformation. Moreover, an important enhancement of the reactivity is revealed, by using a diadamantyl-based macrocycle. On the contrary, electron-withdrawing groups at the *N*-benzylic group of the thread favoured the intramolecular cyclization process (*ρ* = +3.85). Significantly, the latter *ρ* value tripled the first, revealing that the reaction is more sensitive to these substituent effects.^58^

### 2.4 Study of kinetic isotopic effects (KIE) in the cyclization of interlocked *N*-benzylfumaramides 1a and 4

**Scheme 2:**
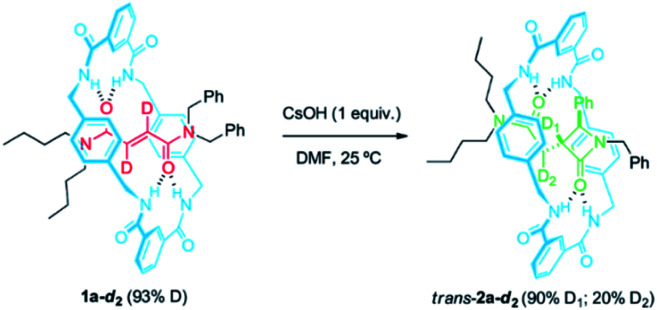
CsOH-promoted intramolecular cyclization of interlocked *N*,*N*-dibenzylfumaramide 1a-*d*_2_ (93% D).

At this point, we decided to evaluate the KIE over the reaction rate when the reference rotaxane 1a was labelled with deuterium atoms at different positions.^59^ First, we compared the cyclization rate of 1a and its labelled analog 1a-*d*_2_ (93% D) having two deuterium atoms at the olefinic moiety of the fumaramide fragment (Scheme 2). Similar rate constants for the cyclization of both 1a and 1a-*d*_2_ were obtained (*k*_1a_ = 1.28 × 10^−4^ s^−1^ and *k*_1a__-*d*_2__ = 1.27 × 10^−4^ s^−1^), indicating that the secondary KIE is negligible (see ESI for further details†). Having in mind the labelling degree of the starting material, a 97% of *D*_1_ is retained in *trans*-2a-*d*_2_ (12 h of reaction time), while the *D*_2_ labelling decreases to a 22% due to a progressive hydrogen-deuterium exchange occurring under the basic reaction conditions.

**Fig. 4:**
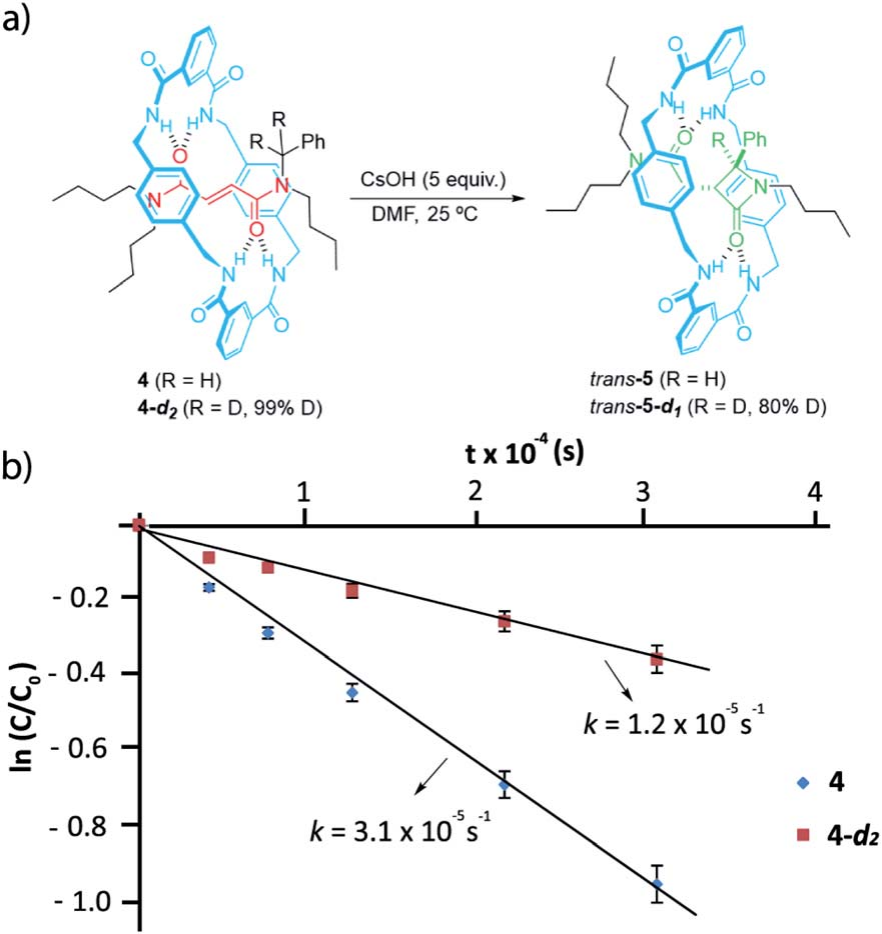
(a) Synthesis of interlocked β-lactams *trans*-5 and *trans*-5-*d*_1_; (b) plots of ln(*c*/*c*_o_) *versus* time for the determination of the rate constants of the cyclizations of rotaxanes 4 and 4-*d*_2_ in the presence of CsOH (5 equiv.) at room temperature in DMF.

We next labelled the benzylic methylene group. For experimental simplicity, we synthesised rotaxanes 4 and 4-*d*_2_ (Fig. 4a), having a thread with one benzyl group and three *n*-butyl chains as stoppers (threads T1b or T1b-*d*_2_, see ESI for further details†).^60^ The cyclization of both rotaxanes 4 and 4-*d*_2_ (99% D) needed 5 equiv. of CsOH in order to avoid the dethreading phenomenon by speeding up the reaction.^46^ The ^1^H-NMR monitoring allowed us to determine the respective rate constants for the formation of the interlocked β-lactams (Fig. 4b). After the cyclization, an 80% of the initial deuterium was maintained in *trans*-5-*d*_1_. The cyclization of the labelled 4-*d*_2_ occurred slower than the non-deuterated one, indicating a noticeable primary KIE (*k*_H_/*k*_D_ ∼ 3) which implies that the benzylic C–H(D) bond is broken during the rate-determining step.^61^

### 2.5 Computational study: a new mechanistic approach

**Fig. 5:**
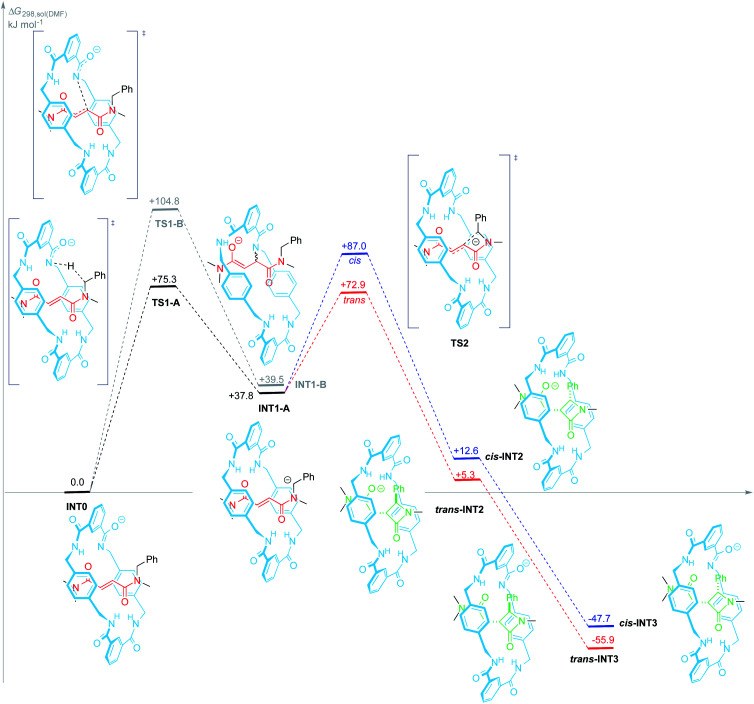
Proposed mechanism for the formation of β-lactams *trans*-INT3 and *cis*-INT3 from the amidate INT0 at the SMD(DMF)/DLPNO-CCSD(T)/ma-def2-SVP//wB97X-D/def2-SVP theoretical level. Gibbs free energies are reported in kJ mol^−1^ (1 atm and 298 K), relative to INT0. Path colours refer to the alternative trajectories for the 4-*exo-trig* cyclization step: *trans* (red) and *cis* (blue).

A putative mechanism for the cyclization of interlocked *N*-benzylfumaramides in basic media was described in our previous works.^46,62^ We proposed therein that the resulting β-lactams might be formed *via* a mechanism initiated by the deprotonation of the amide function of the ring followed by the intramolecular aza-Michael reaction (IMAMR) of the formed anion to the electrophilic C=C double bond of the thread. However, to the light of the experimental results shown herein, we presumed that a second alternative route should be also considered, that involving the same initial step but followed by the proton transfer from the benzyl group of the thread to the macrocyclic anion. Thus, we scrutinized these two alternative mechanistic pathways for the base-promoted cyclization of interlocked *N*-benzylfumaramides 1 into β-lactams 2 at the SMD(DMF)/DLPNO-CCSD(T)/ma-def2-SVP//wB97X-D/def2-SVP theoretical level. For simplicity the [2]rotaxane 1j, featuring a *N*-benzyl-*N*,*N*′,*N*′-trimethylfumaramide thread, was selected as a model structure. In this section we explain more in detail only the mechanistic pathway of lowest energy, whereas the most relevant steps of the not favoured mechanistic route are briefly summarized (see Fig. 5 and the ESI for a complete mechanistic description†).

The first stage of both mechanisms is reasonably the deprotonation of one amide function of the macrocycle, bearing *a priori* the most acidic proton, to give the amidate INT0. Proton transfer between oxygen and nitrogen acids and bases are usually fast and, since four amide groups are embedded into the macrocycle, the proton removal from 1j is also statistically favoured. Thus, we presumed this proton transfer to be diffusion controlled.

Amidate anion INT0 could evolve through two different pathways: (A) acting as an internal base by abstraction of one benzylic proton of the thread thus leading to the carbanion INT1-A (+37.8 kJ mol^−1^); or (B) adding to the electrophilic C=C bond of the fumaramide *via* an intramolecular aza-Michael reaction (IMAMR) to give a more energetic enolate anion INT1-B (+39.5 kJ mol^−1^). Computational results predict that the transition structure TS1-A is 29.5 kJ mol^−1^ lower in energy than TS1-B, most likely due to the strained conformation that the macrocycle must adopt in TS1-B in order to reach the alkene moiety. The successive computed stationary points along path B resulted all higher in energy than TS1-A, the rate-determining transition state of pathway A. Thus, the former aza-Michael proposal is a route non-competitive with the new revised mechanism (see ESI for further details, Fig. S6†).

Following with path A, the resulting *N*-benzyl carbanion could approach to the nearest C_sp^2^_ of the alkene moiety of the fumaramide unit following two alternative trajectories, leading to the diastereoisomeric enolates *trans*- and *cis*-INT2 (Δ*G*_*trans*-__INT2_ = +5.3 kJ mol^−1^ and Δ*G*_*cis*-__INT2_ = +12.6 kJ mol^−1^) in a 4-*exo-trig* cyclization. Both enolates would be then internally protonated by one of the amide functions of the macrocycle to give the more stable amidates *cis*- and *trans*-INT3, with respective energies of −47.7 and −55.9 kJ mol^−1^, which would finally transform into the interlocked β-lactams 2 by an external protonation. Pleasantly, our simulations predict that *cis*-TS2 is higher in energy than *trans*-TS2 by +14.1 kJ mol^−1^ a difference that translates into a theoretical diastereoisomeric ratio higher than 99 : 1 in favour of *trans*-INT2, in agreement with the observed experimental diastereoselectivity (*trans*-2 were the only reaction products).

**Fig. 6:**
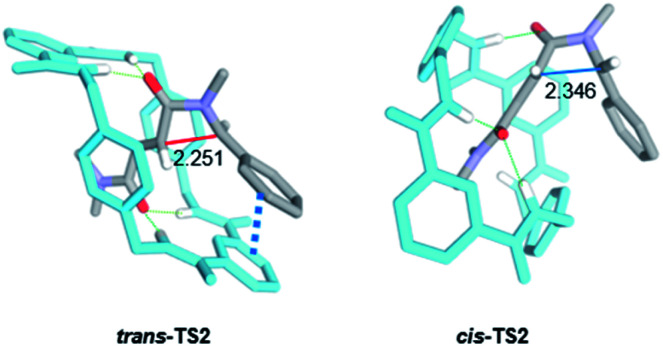
Computed transition structures *trans*-TS2 and *cis*-TS2. Distances shown in Angstroms (Å). Selected hydrogen atoms are hidden for clarity.

Aiming to shed light on the trajectory preference for the 4-*exo-trig* cyclization step, responsible of the selective formation of the *trans*-2 interlocked lactams, both transition structures *trans*-TS2 and *cis*-TS2 were analysed in detail (Fig. 6). The noncovalent interactions established between the two components of the [2]rotaxanes along with some other structural features justify the energy difference between the two transition states. The forming C–C bond distances are similar in both transition structures, that of *trans*-TS2 being 0.1 Å shorter. A chair-like conformation of the macrocycle is observed in *trans*-TS2, therefore keeping the four hydrogen bonds between the NH groups of the macrocycle and the two carbonyl units of the thread present in the starting rotaxane. In addition, the phenyl group of the thread sets a stabilizing aromatic–aromatic interaction with one isophthalamide unit of the macrocycle (the distance between the centroids of both rings is 3.72 Å). On the contrary, in *cis*-TS2 the thread adopts a tweaked conformation hampering the formation of the fourth hydrogen bond and forcing to the macrocycle to adopt a distorted conformation, which translates into an energy penalty.

Thus, the energetically favourable mechanistic proposal (path A) accounts for the active role of the macrocycle in the cyclization process in two ways. First, the deprotonated macrocycle acts as an activating element by internally removing one proton from the *N*-benzyl group of the fumaramide thread during the rate-determining step. And second, the steric constraint imposed on the interlocked transition state *cis*-TS2 is the stereo-differentiating element that controls the diastereoselective outcome of the cyclization process. Accordingly, the macrocycle offers an appropriate restricted environment where the stereoselective cyclization takes place. In fact, the cyclization of the noninterlocked thread T1a in the presence of CsOH leads to a *cis*–*trans* mixture of β-lactams in poor yield (see ESI for computational details, Fig. S7†).

The confined microenvironment governs the macrocycle–thread interactions, in terms of the orientation and reactive conformation, thereby exerting control over the chemo-, regio, and stereoselectivity of the transformation and resulting in much improved activity compared with that of the bulk solution. As a matter of the fact, the mechanistic pathway A agrees well with our results above on the structure-reactivity relationships and kinetic isotopic effects. Thus, we proved that electron-withdrawing groups attached to the *para* position of the *N*-benzyl groups of the fumaramide thread accelerated the cyclization. Consistently, the Hammett plot of log(*k*/*k*_1a_) against the *σ*_*p*_ constants affords a positive *ρ* value of +3.85 (Fig. 2b) reflecting that electron-withdrawing groups stabilize the incipient negative charge at the benzylic carbon by resonance effects. On the other hand, electron-donating groups at the 5-position of the isophthaloyl groups of the macrocycle accelerated the cyclization step, with electron-withdrawing substituents acting in the opposite direction (*ρ* = −1.24, Fig. 3b). This fact can be rationalized as a consequence of the amidate anion acting as an internal base in the rate-determining step and the effect of electron-donating groups increasing its basicity, favouring the *N*-benzyl deprotonation. Finally, the substantial primary isotopic effect observed in the cyclization of labelled 3-*d*_2_ (*k*_H_/*k*_D_ ∼ 3) is a strong evidence of the benzylic C–H bond being broken in the rate-determining step.

**Fig. 7:**
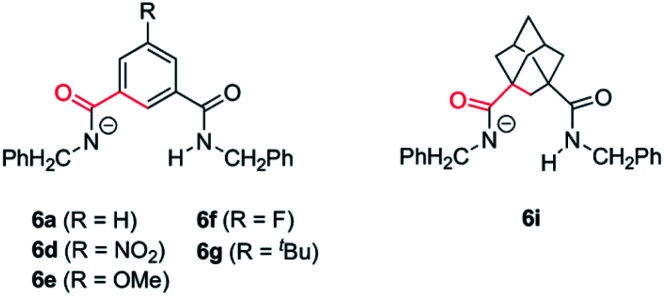
Structures of the monoamidates 6a, 6d–g and 6i. The conformational analysis was performed along the bonds coloured in red.

As we mentioned above rotaxane 1i, featuring two adamantyl fragments in the macrocycle, experiences the fastest cyclization. In order to explain that, we considered that the torsional flexibility of the adamantyl core^56^ enables the proper orientation of the amidate group, favouring the expeditious deprotonation of the *N*-benzyl group of the thread. In that vein we computationally studied the conformational preference of a range of structurally simplified monoamidates 6 (Fig. 7). Interestingly, we found a relationship between the rates of the cyclization measured with the real interlocked fumaramides of comparable structures 1 and the degrees of flexibility of the simplified amidate anions 6. Thus, the rotational barrier along the NCO–Ar bond increases in the following order: 6i ⋘ 6g ≈ 6a ≈ 6e < 6f < 6d (see ESI for further details, Fig. S8–S14†) which parallels the reactivity trend found with the real rotaxanes 1.

The formation of the amidate anion is also important in order to maintain the structural integrity of the interlocked systems. In fact, a control experiment of the stability was carried out with a rotaxane 1k, having the same macrocycle as 1a and *N*,*N*,*N*′,*N*′-tetrabutylfumaramide as thread,^48^ that precludes the cyclization to occur. While the dethreading of this rotaxane in DMF at 25 °C takes place in a 15% of conversion in 24 h, the presence of CsOH (and the subsequent formation of the amidate anion) almost inhibits this process (5% with 1 equiv. of base, 2% with 3 equiv. of base, see Table S4†).

One last point to be settled for the sake of completeness is the role of the cesium base. As we detailed above, we observed that the usual amount of CsOH (1 equiv.) needed to be increased (3–5 equiv.) in order to avoid the dethreading of rotaxanes either bearing electron-withdrawing substituents at the macrocycle or with only one benzyl group at the extremes of the thread. Thus, 3 equiv. of CsOH instead of 1 equiv. approximately tripled the reaction rate of 1a (see ESI for further details, Fig. S2†).

Unfortunately, CsOH is not completely soluble in DMF under the reaction conditions and thus its concentration cannot be accurately determined. Some authors have stated that cesium salts are nearly or completely dissociated in dipolar aprotic solvents, such as DMSO or DMF, producing very reactive “naked” anions.^63,64^ Thus, a close interaction between the cesium cation and the amidate anion is highly unlikely. Nevertheless, our results apparently point to the deprotonation of the –CONH– fragments at the macrocycle being somewhat slower than expected, probably due to the intercomponent hydrogen bonds established between those NH protons and the carbonyl groups of the fumaramide thread.

### 2.6 Synthesis of interlocked *trans* β-lactams with less sterically-hindered threads

**Scheme 3:**
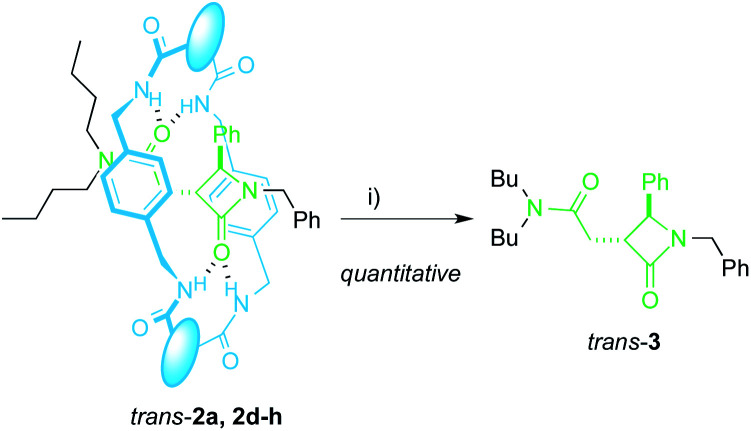
Dethreading of interlocked lactams *trans*-2a, 2d–h. Reaction conditions: (i) DMF, 12 h, 100 °C. The different fragments in the macrocycle are disclosed in Table 2.

The extrusion of the chiral azetidinone threads from the interlocked products *trans*-2 completes an unusual method to obtain high value-added β-lactams^50,51^ from *N*-benzylfumaramides with the intermediacy of the interlocked species 1 and 2. In fact, compounds *trans*-2 may be considered as kinetically stable pseudorotaxanes^65^ and their heating in DMF at 100 °C for 12 h quantitatively yielded the respective dethreaded lactam *trans*-3 (Scheme 3). Unfortunately, the deslipping of the Bu_2_N-stoppered lactam from the diadamantyl rotaxane *trans*-2i did not occur even after longer reaction times or higher temperatures (up to 130 °C).

**Scheme 4:**
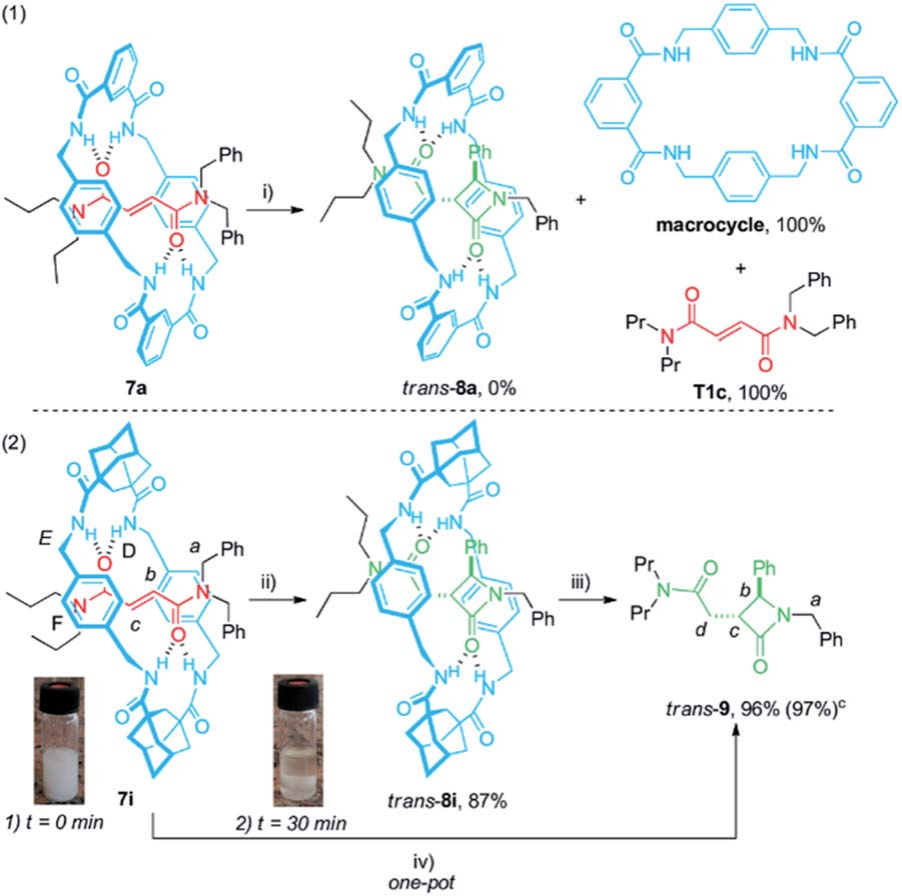
Cyclization of rotaxanes 7a and 7i. Synthesis of the unthreaded lactam *trans*-9. ^*a*,*b a*^ Reaction conditions: (i) CsOH (1 equiv.), DMF, 12 h, 25 °C; (ii) CsOH (1 equiv.), DMF, 30 min, 25 °C; (iii) DMF, 2 h, 100 °C; (iv) CsOH (1 equiv.), DMF, 30 min, 25 °C; then neutralization with HCl 1 M (1 equiv.) and subsequent heating 2 h at 100 °C; ^*b*^ insets: (1) picture of the reaction vial at 0 min reaction time; (2) picture of the reaction vial after 30 min; ^*c*^ yield obtained in the one-pot procedure.

With the aim of taking advantage of the fast cyclization experienced by the diadamantyl-based rotaxane 1i, we envisaged that a less sterically hindered thread could expedite the extrusion of the corresponding β-lactam. To explore this possibility, we synthesised rotaxanes 7a and 7i, bearing a dipropylamino-stoppered thread (T1c) in combination with isophthaloyl- and adamantyl-derived macrocycles (Scheme 4).

When rotaxane 7a^48^ was submitted to the standard cyclization conditions the corresponding lactam *trans*-8a was apparently not formed. Instead, only the dethreaded components T1c and the polyamide macrocycle, poorly soluble in DMF, were identified in the reaction mixture (Scheme 4, eqn (1)). In contrast, rotaxane 7i, which is moderately insoluble in DMF (Scheme 4, eqn (2), inset (1)), transformed in the presence of base under more diluted conditions (10 mM) after a short period of time (30 min) into *trans*-8i, fully soluble in DMF, in high yield (Scheme 4, eqn (2), inset (2)). The dethreading of *trans*-8i to give the β-lactam *trans*-9 took place successfully by heating at 100 °C in DMF solution for 2 h. As we presumed, a one-pot procedure quantitatively provided the desired azetidinone *trans*-9 from the interlocked fumaramide 7i.

**Fig. 8:**
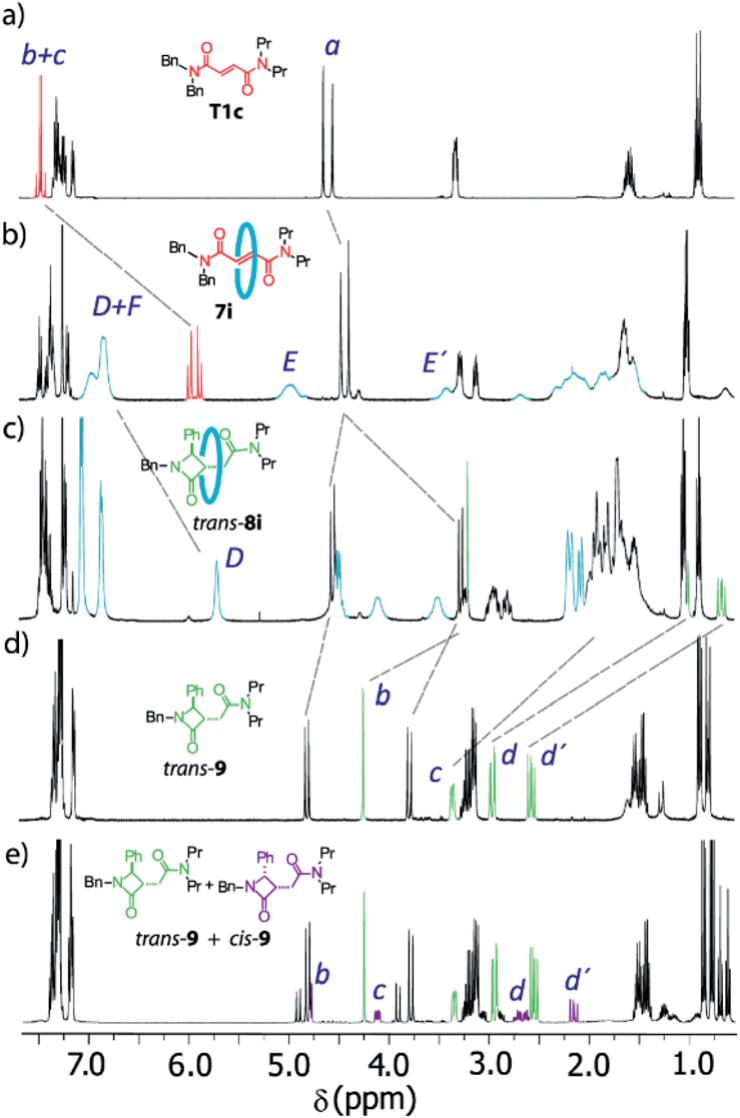
^1^H NMR spectra (400 MHz, CDCl_3_, 298 K) of (a) *N*,*N*-dibenzyl-*N*′,*N*′-dipropylfumaramide T1c; (b) fumaramide-based [2]rotaxane 7i; (c) interlocked β-lactam *trans*-8i; (d) β-lactam *trans*-9; and (e) mixture of β-lactams *cis*- and *trans*-9 obtained by cyclization of the thread T1c. Lettering as in Scheme 4.

**Fig. 9:**
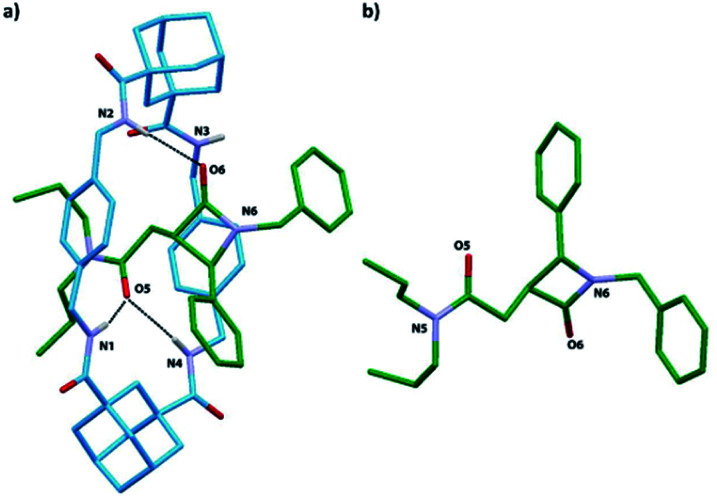
X-ray structure of: (a) the [2]rotaxane *trans*-8i; (b) the thread of [2]rotaxane *trans*-8i (the macrocycle was removed for clarity). Intramolecular hydrogen-bond lengths [Å] (and angles [deg.]): O5HN1 2.19 (164); O5HN4 2.34 (168); O6HN2 2.13 (164).

The ^1^H NMR spectra of the thread T1c, rotaxanes 7i and *trans*-8i, the free lactam *trans*-9 and a mixture of lactams *trans*- and *cis*-9 are stacked in Fig. 8. As expected, the protons of the fumaramide function in the rotaxane 7i (H_*b*_ and H_*c*_) experience shifts to higher field (Δ*δ* ∼ 1.54 ppm) due to the anisotropic effect caused by the macrocycle (Fig. 9b, signals in red colour). The highly diastereoselective CsOH-promoted cyclization of the rotaxane 7i leads to the interlocked β-lactam *trans*-8i as a single product (Fig. 8c). As a result of the thermal dethreading process, only the signals of the β-lactam *trans*-9 are observed, with large displacements to higher frequencies observed for the protons of the lactam core H_*b*_, H_*c*_ as well as those of the methylene group H_*d*_ (Δ*δ* = 1.04–1.90 ppm) (Fig. 8d, signals in green colour). In contrast, the cyclization of the non-interlocked fumaramide T1c gives a 2.9 : 1 mixture of diastereoisomeric β-lactams *trans*- and *cis*-9 (Fig. 8e, signals of *cis*-9 in violet colour) besides other minor compounds resulting from the decomposition of *trans*- and *cis*-9 under the reaction conditions. The last result demonstrates not only the protective function of the macrocycle, but also the definitive role of the mechanical bond controlling the diastereoselectivity of the cyclization.

Single crystals were obtained by slow diffusion of pentane into a dichloroethane solution of rotaxane *trans*-8i. The X-ray analysis confirmed the interlocked nature of *trans*-8i and the relative configuration of the chiral atoms at the azetidinone ring (Fig. 9). Interestingly, the diadamantyl-based macrocycle shows a distorted chair-like conformation, different to that usually found in isophthalamide-based systems.^66,67^ One of the two adamantanedicarboxamide units establishes bifurcated hydrogen bonds of the NH protons with the carbonyl group at the side-chain of the azetidinone, whereas the carbonyl group of the β-lactam ring forms a hydrogen bond with only one of the other NH protons of the macrocycle.

## 3. Conclusions

A precise inspection of the mechanism of the CsOH-promoted cyclization of fumaramides embedded in the tetraamide ring of a [2]rotaxane is presented. The mechanistic analysis has been addressed from both, experimental and computational approaches, which range experimental evaluation of the structure-reactivity relationship (Hammet-plots), KIE studies with deuterium-labelled interlocked fumaramides and last but not least, computational analysis of two alternative mechanistic pathways.

On the basis of the attained results we have excluded our previous mechanistic proposal consisting on the intramolecular aza-Michael reaction (IMAMR) of the deprotonated macrocycle to the electrophilic C=C double bond of the thread. By contrast, our new findings support a rate-determining deprotonation of the *N*-benzyl group of the thread through the short space separating the interlocked components, by the amidate group of the macrocycle generated by the external base. The benzylic carbanion resulting from this proton transfer undergoes a clean cyclization inside the cavity of the ring. The mechanically interlocked topology of this latter element is translated into a neat stereocontrol in favour of one of the two putative stereisomeric transition states, in a way resembling the interactions established at the active pocket of natural or artificial catalysts.

Our results also reveal that diadamantyl-derived rotaxanes speed up the cyclization process. The computational analysis has confirmed that the flexibility of the adamantyl core enables the proper orientation of the amidate group, favouring the fast deprotonation of the *N*-benzyl group of the thread. The expeditious cyclization of an adamantyl-derived pseudorotaxane and the subsequent high-yielding dethreading emphasize the synthetic applicability of this methodology.

In summary, we have clearly demonstrated the potential of the CsOH-promoted cyclization of fumaramide rotaxanes where, the appropriate combination of polyamide macrocycle and thread not only allows a fine tuneable design of our interlocked molecules without compromising their structural integrity but also presents some unique characteristics, such as accelerating the reaction rate, enhancing reactivity and selectivity which finally leads to a regio- and diastereoselective synthesis of β-lactams. The full understanding of this process opens the door to the study of other transformations that could occur in the cavity of a polyamide macrocycle, including the development of the asymmetric version of this cyclization of interlocked *N*-benzylfumaramides.

## Conflicts of interest

There are no conflicts to declare.

## Supplementary Material

SC-012-D0SC05757F-s001

SC-012-D0SC05757F-s002

## References

[cit1] Stimulating concepts in chemistry, ed. M. Shibasaki, J. F. Stoddart and F. Vögtle, Wiley-VCH, Weinheim, Germany, 2005

[cit2] Heard A. W., Goldup S. M. (2020). Chem.

[cit3] Knowles J. R. (1991). Nature.

[cit4] Enzyme catalysis in organic synthesis, ed. K. Drauz, H. Gröger and O. May, Wiley-VCH, New York, 2012

[cit5] Arnold F. H. (2018). Angew. Chem., Int. Ed..

[cit6] Lancaster L., Abdallah W., Banta S., Wheeldon I. (2018). Chem. Soc. Rev..

[cit7] Zhang X., Houk K. N. (2005). Acc. Chem. Res..

[cit8] Wang J., Feringa B. L. (2011). Science.

[cit9] Zhao D., Neubauer T. M., Feringa B. L. (2015). Nat. Commun..

[cit10] Blanco V., Leigh D. A., Marcos V. (2015). Chem. Soc. Rev..

[cit11] De Bo G., Leigh D. A., McTernan C. T., Wang S. (2017). Chem. Sci..

[cit12] Kassem S., Lee A. T. L., Leigh D. A., Marcos V., Palmer L. C., Pisano S. (2017). Nature.

[cit13] Romanazzi G., Degennaro L., Mastrorilli P., Luisi R. (2017). ACS Catal..

[cit14] van Dijk L., Tilby M. J., Szpera R., Smith O. A., Bunce H. A. P., Fletcher S. P. (2018). Nat. Rev. Chem..

[cit15] Choudhury J. (2018). Tetrahedron Lett..

[cit16] Neal E. A., Goldup S. M. (2014). Chem. Commun..

[cit17] Lewis J. E. M., Galli M., Goldup S. M. (2017). Chem. Commun..

[cit18] Thordarson P., Bijsterveld E. J. A., Rowan A. E., Nolte R. J. M. (2003). Nature.

[cit19] Tachibana Y., Kihara N., Takata T. (2004). J. Am. Chem. Soc..

[cit20] Hattori G., Hori T., Miyake Y., Nishibayashi Y. (2007). J. Am. Chem. Soc..

[cit21] Berna J., Alajarin M., Orenes R.-A. (2010). J. Am. Chem. Soc..

[cit22] Blanco V., Carlone A., Hänni K. D., Leigh D. A., Lewandowski B. (2012). Angew. Chem., Int. Ed..

[cit23] Lewandowski B., De Bo G., Ward J. W., Papmeyer M., Kuschel S., Aldegunde M. J., Gramlich P. M. E., Heckmann D., Goldup S. M., D'Souza D. M., Fernandes A. E., Leigh D. A. (2013). Science.

[cit24] Blanco V., Leigh D. A., Lewandowska U., Lewandowski B., Marcos V. (2014). J. Am. Chem. Soc..

[cit25] Blanco V., Leigh D. A., Marcos V., Morales-Serna J. A., Nussbaumer A. L. (2014). J. Am. Chem. Soc..

[cit26] Leigh D. A., Marcos V., Wilson M. R. (2014). ACS Catal..

[cit27] Galli M., Lewis J. E. M., Goldup S. M. (2015). Angew. Chem., Int. Ed..

[cit28] Hoekman S., Kitching M. O., Leigh D. A., Papmeyer M., Roke D. (2015). J. Am. Chem. Soc..

[cit29] Cakmak Y., Erbas-Cakmak S., Leigh D. A. (2016). J. Am. Chem. Soc..

[cit30] Pan T., Liu J. (2016). ChemPhysChem.

[cit31] Martinez-Cuezva A., Saura-Sanmartin A., Nicolas-Garcia T., Navarro C., Orenes R.-A., Alajarin M., Berna J. (2017). Chem. Sci..

[cit32] Mitra R., Zhu H., Grimme S., Niemeyer J. (2017). Angew. Chem., Int. Ed..

[cit33] Pairault N., Zhu H., Jansen D., Huber A., Daniliuc C. G., Grimme S., Niemeyer J. (2020). Angew. Chem., Int. Ed..

[cit34] Calles M., Puigcerver J., Alonso D. A., Alajarin M., Martinez-Cuezva A., Berna J. (2020). Chem. Sci..

[cit35] Martinez-Cuezva A., Marin-Luna M., Alonso D. A., Ros-Ñiguez D., Alajarin M., Berna J. (2019). Org. Lett..

[cit36] Dommaschk M., Echavarren J., Leigh D. A., Marcos V., Singleton T. A. (2019). Angew. Chem., Int. Ed..

[cit37] Biagini C., Fielden S. D. P., Leigh D. A., Schaufelberger F., Di Stefano S., Thomas D. (2019). Angew. Chem., Int. Ed..

[cit38] Lim J. Y. C., Yuntawattana N., Beer P. D., Williams C. K. (2019). Angew. Chem., Int. Ed..

[cit39] Parham A. H., Windisch B., Vögtle F. (1999). Eur. J. Org. Chem..

[cit40] Ghosh P., Mermagen O., Schalley C. A. (2002). Chem. Commun..

[cit41] Oku T., Furusho Y., Takata T. (2003). Org. Lett..

[cit42] Leigh D. A., Perez E. (2004). Chem. Commun..

[cit43] D'Souza D. M., Leigh D. A., Mottier L., Mullen K. M., Paolucci F., Teat S. J., Zhang S. (2010). J. Am. Chem. Soc..

[cit44] Winn J., Pinczewska A., Goldup S. M. (2010). J. Am. Chem. Soc..

[cit45] Modicom F., Jamieson E. M. G., Rochette E., Goldup S. M. (2019). Angew. Chem., Int. Ed..

[cit46] Martinez-Cuezva A., Lopez-Leonardo C., Bautista D., Alajarin M., Berna J. (2016). J. Am. Chem. Soc..

[cit47] Martinez-Cuezva A., Rodrigues L. V., Navarro C., Carro-Guillen F., Buriol L., Frizzo C. P., Martins M. A. P., Alajarin M., Berna J. (2015). J. Org. Chem..

[cit48] Martinez-Cuezva A., Morales F., Marley G. R., Lopez-Lopez A., Martinez-Costa J. C., Bautista D., Alajarin M., Berna J. (2019). Eur. J. Org. Chem..

[cit49] Affeld A., Hübner G. M., Seel C., Schalley C. A. (2001). Eur. J. Org. Chem..

[cit50] Alcaide B., Almendros P., Arangoncillo C. (2007). Chem. Rev..

[cit51] Pitts C. R., Lectka T. (2014). J. Am. Chem. Soc..

[cit52] Martinez-Cuezva A., Bautista D., Alajarin M., Berna J. (2018). Angew. Chem., Int. Ed..

[cit53] Berna J., Bottari G., Leigh D. A., Perez E. (2007). Pure Appl. Chem..

[cit54] Panman M. R., Bakker B. H., den Uyl D., Kay E. R., Leigh D. A., Buma W. J., Brouwer A. M., Geenevasen J. A. J., Woutersen S. (2013). Nat. Chem..

[cit55] Berna J., Alajarin M., Martínez-Espín J. S., Buriol L., Martins M. A. P., Orenes R.-A. (2012). Chem. Commun..

[cit56] Collins C. G., Johnson A. T., Connel R. D., Nelson R. A., Murgu I., Oliver A. G., Smith B. D. (2014). New J. Chem..

[cit57] We verified that the rate of the cyclization of 1a followed a first-order regime in the range of 10 to 25 mM (see ESI, Fig. S1†).

[cit58] Carey F. A. and Sundberg R. J., Advanced Organic Chemistry, Springer, New York, 2007

[cit59] Wiberg K. B. (1955). Chem. Rev..

[cit60] The corresponding non deuterated 4 has been previously synthetized and described in ref. 46.

[cit61] Westheimer F. H. (1961). Chem. Rev..

[cit62] Martinez-Cuezva A., Lopez-Leonardo C., Alajarin M., Berna J. (2019). Synlett.

[cit63] Dijkstra G., Kruizinga W. H., Kellogg R. M. (1987). J. Org. Chem..

[cit64] Salvatore R. N., Nagle A. S., Jung K. W. (2002). J. Org. Chem..

[cit65] Ashton P. R., Baxter I., Fyfe M. C. T., Raymo F. M., Spencer N., Stoddart J. F., White A. J. P., Williams D. J. (1998). J. Am. Chem. Soc..

[cit66] Lopez-Leonardo C., Martinez-Cuezva A., Bautista D., Alajarin M., Berna J. (2019). Chem. Commun..

[cit67] Gatti F. G., Leigh D. A., Nepogodiev S. A., Slawin A. M. Z., Teat S. J., Wong J. K. Y. (2001). J. Am. Chem. Soc..

