# Supplementary material for: Cyclization of interlocked fumaramides into β-lactams: experimental and computational mechanistic assessment of the key intercomponent proton transfer and the stereocontrolling active pocket
Source: Chem Sci. 2020 Nov 11;12(2):747–56. doi: 10.1039/d0sc05757f (PMC8178992; doi:10.1039/d0sc05757f)
Supplement: SC-012-D0SC05757F-s001 [file SC-012-D0SC05757F-s001.pdf]

Electronic Supplementary Material (ESI) for Chemical Science.
This journal is © The Royal Society of Chemistry 2020

Supporting Information

Cyclization of Interlocked Fumaramides into β-Lactams: Experimental and Computational Mechanistic Assessment of the Key Intercomponent Proton Transfer and the Stereocontrolling Active Pocket

Alberto Martinez-Cuezva,[†,*] Aurelia Pastor,[†,*] Marta Marin-Luna,[†] Carmen Diaz-Marin,[†] Delia Bautista,[‡] Mateo Alajarin,[†] Jose Berna[†,*]

[†] Department of Organic Chemistry, Faculty of Chemistry. University of Murcia. Regional Campus of International Excellence "Campus Mare Nostrum", 30100 Murcia, Spain. [‡] ACTI, University of Murcia. Campus de Espinardo, 30100 Murcia, Spain

E-mail: amcuezva@um.es

aureliap@um.es

ppberna@um.es

Table of Contents ………………………………………………………………………………

1. General Experimental Information …………………………………………………… S3

2. Synthesis of threads T1a-c, T1a-*d*$_2$ and T1b-*d*$_2$ ……………………………………….. S4

3. General procedure for the preparation of [2]rotaxanes 1, 4 and 7 …………………….. S6

4. General procedure for the base-catalyzed cyclization of [2]rotaxanes 1, 4 and 7 ……… S17

5. Synthesis of lactams *trans*-3 and *trans*-9 by thermal dethreading ……………………… S26

6. One-pot synthesis of lactam *trans*-9 ……………………………………………………… S27

7. Synthesis of β-lactams 3 and 9 from threads T1a and T1c ……………………………… S28

8. Kinetic studies for the cyclization of rotaxane 1a ……………………………………….. S29

9. Kinetic measurements for the calculation of the dethreading constant rates of the interlocked fumaramides 1a and 1d-h …………………………………………………… S30

10. Calculation of the rate constants for the CsOH-catalyzed intramolecular cyclization of interlocked fumaramides 1 and 4 …………………………………………………… S37

11. Hammet Plot values …………………………………………………………………… S58

12. Determination of the deuterium distribution in interlocked lactams *trans*-2a-*d*$_2$ and *trans*-5-*d*$_1$ ……………………………………………………………………………... S59

13. Crystal data and structure refinement for rotaxane *trans*-8i ………………………… S61

14. CsOH effect on the stability of rotaxane 1k …………………………………………… S64

15. ^{1}H and ^{13}C NMR spectra of synthesized compounds ………………………………….. S65

16. Computational methods ……………………………………………………………… S88

17. Alternative computed mechanism for the cyclization of the interlocked fumaramides 1 …………………………………………………………………………………………….. S88

18. Computed mechanism for the cyclization of *N*-benzyl-*N,N’,N’*-trimethylfumaramide S90

19. Conformational analysis of the monoamidates 6 and their corresponding *N,N'*-dimethylisophthalamides 6’ …………………………………………………………… S92

20. Computational data …………………………………………………………………… S96

21. Cartesian coordinates ………………………………………………………………… S101

22. References ……………………………………………………………………………... S115

1. General Experimental Information

Unless stated otherwise, all reagents were purchased from Aldrich Chemicals and used without further purification. CsOH (99.9 % purity) was purchased from *Alfa Aesar*. HPLC grade solvents (*Scharlab*) were nitrogen saturated and were dried and deoxygenated using an Innovative *Technology Inc. Pure-Solv 400 Solvent Purification System*. Column chromatography was carried out using silica gel (60 Å, 70-200 μm, *SDS*) as stationary phase, and TLC was performed on precoated silica gel on aluminum cards (0.25 mm thick, with fluorescent indicator 254 nm, *Fluka*) and observed under UV light. All melting points were determined on a *Kofler* hot-plate melting point apparatus and are uncorrected. ^{1}H- and proton-decoupled ^{13}C spectra were recorded on a *Bruker Avance* 300, 400 and 600 MHz instruments. ^{1}H NMR chemical shifts are reported relative to Me_4Si and were referenced via residual proton resonances of the corresponding deuterated solvent, whereas ^{13}C NMR spectra are reported relative to Me_4Si using the carbon signals of the deuterated solvent. Signals in the ^{1}H and ^{13}C NMR spectra of the synthesized compounds were assigned with the aid of DEPT-135, APT, or two-dimensional NMR experiments (COSY, HMQC and HMBC). Abbreviations of coupling patterns are as follows: br, broad; s, singlet; d, doublet; t, triplet; q, quadruplet; m, multiplet. Coupling constants (*J*) are expressed in Hz. High-resolution mass spectra (HRMS) were obtained using a time-of-flight (TOF) instrument equipped with electrospray ionization (ESI).

Abbreviation list:

DCC: *N,N'*-Dicyclohexylcarbodiimide
DMAP: dimethylaminopyridine
HOBt: hydroxybenzotriazole
TLC: thin layer chromatography

2. Synthesis of threads T1a-c, T1a-*d*$_2$ and T1b-*d*$_2$

Thread T1a

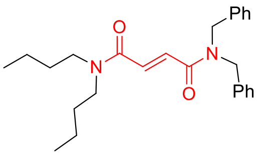

Thread **1a** was synthesized as described in bibliography and showed identical spectroscopic data as those reported therein.[1]

Thread T1a-*d*$_2$ (93% D)

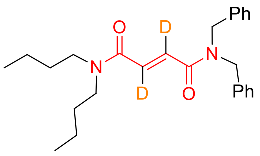

To a stirred solution of (*E*)-4-(dibenzylamino)-4-oxobut-2-enoic acid-*d*$_2$[2] (93% D) (1.91 g, 6.45 mmol) in anhydrous CH_2Cl_2 (50 mL) was added dibutylamine (1.0 g, 7.75 mmol), HOBt (1.05 g, 7.75 mmol) and DMAP (94 mg, 0.77 mmol). The reaction mixture was cooled to 0ºC and DCC (1.60 g, 7.75 mmol) was added. After 30 min, the reaction was warmed to room temperature and stirred for 24 h. After this time the resulting suspension was filtered and the filtrate was washed with an aqueous solution of 1 M HCl (2 x 30 mL), saturated $NaHCO_3$ (2 x 30 mL) and brine (2 x 30 mL). The organic phase was dried over anhydrous $MgSO_4$ and concentrated under reduced pressure. The resulting residue was subjected to column chromatography (silica gel) using a mixture of hexane/AcOEt (3/1). The solvent was removed under reduced pressure to give the title product as a yellow oil (**T1a-*d*$_2$**, 1.47 g, 56%); ^{1}H NMR (300 MHz, $CDCl_3$, 298 K): δ 7.39-7.14 (m, 10H), 4.66 (s, 2H), 4.56 (s, 2H), 3.41-3.32 (m, 4H), 1.62-1.50 (m, 4H), 1.39-1.20 (m, 4H), 0.96 (t, *J* = 7.2 Hz, 3H), 0.92 (t, *J* = 7.2 Hz, 3H); ^{13}C NMR (75 MHz, $CDCl_3$, 298 K): δ 166.1 (CO), 164.6 (CO), 136.9 (C), 136.1 (C), 132.5 (t, *J*= 24.5 Hz, CD), 130.6 (t, *J*= 23.5 Hz, CD), 129.0 (CH), 128.8 (CH), 128.4 (CH), 127.9 (CH), 127.7 (CH), 126.8 (CH), 50.1 (CH_2), 48.6 (CH_2), 48.2 (CH_2),

46.7 (CH_2), 32.1 (CH_2), 29.9 (CH_2), 20.4 (CH_2), 20.1 (CH_2), 13.9 (CH_3), 13.9 (CH_3); HRMS (ESI) calcd for $C_{26}H_{33}D_2N_2O_2$ $[M + H]^+$ 409.2819, found 409.2821.

Thread T1b

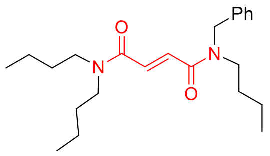

Thread **1b** was synthesized as described in reference 1 and showed identical spectroscopic data as those reported therein.

Thread T1b-*d*₂ (99% D)

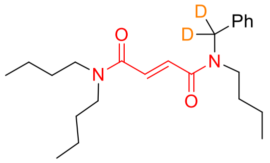

To a stirred solution of (2*E*)-4-(dibutylamino)-4-oxo-2-butenoic acid[1] (272 mg, 1.20 mmol) in anhydrous CH_2Cl_2 (20 mL) was added *N*-benzylbutan-1-amine-*d*$_2$ (99% D) (165 mg, 1 mmol), HOBt (183 mg, 1.20 mmol) and DMAP (14 mg, 0.12 mmol). The reaction mixture was cooled to 0 ºC and DCC (247 mg, 1.20 mmol) was added. After 30 min, the reaction was warmed to room temperature and was stirred for 24 h. After this time the resulting suspension was filtered and the filtrate was washed with an aqueous solution of 1 M HCl (2 x 30 mL), saturated $NaHCO_3$ (2 x 30 mL) and brine (2 x 30 mL). The organic phase was dried over anhydrous $MgSO_4$ and concentrated under reduced pressure. The resulting residue was subjected to column chromatography (silica gel) using a mixture of hexane/AcOEt (2/1). The solvent was removed under reduced pressure to give the title product as a yellow oil (**T1b-*d*$_2$**, 223 mg, 59%); *mixture of rotamers in* $CDCl_3$ (60:40); ^1H NMR (300 MHz, $CDCl_3$, 298 K): δ 7.45-7.10 (m, 7H), 3.44-3.24 (m, 6H), 1.60-1.45 (m, 6H), 1.35-1.20 (m, 6H), 0.95-0.80 (m, 9H); ^{13}C NMR (75 MHz, $CDCl_3$, 298 K): δ 165.7 (CO), 165.4 (CO), 164.7 (CO), 164.7 (CO), 137.2 (C), 136.5 (C), 132.2 (CH), 132.1 (CH), 131.3 (CH), 131.0 (CH),

128.9 (CH), 128.7 (CH), 128.0 (CH), 127.7 (CH), 127.5 (CH), 126.6 (CH), 48.1 (CH_2), 47.2 (CH_2), 46.7 (CH_2), 46.7 (CH_2), 46.4 (CH_2), 32.0 (CH_2), 32.0 (CH_2), 31.5 (CH_2), 29.9 (CH_2), 29.6 (CH_2), 20.3 (CH_2), 20.1 (CH_2), 13.9 (CH_3), 13.9 (CH_3), 13.8 (CH_3); the resonances for the CD carbon atoms are not observed; HRMS (ESI) calcd for $C_{23}H_{35}D_2N_2O_2$ $[M + H]^+$ 375.2975, found 375.2975.

Thread T1c

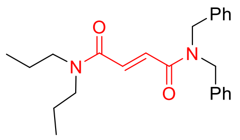

Thread **1c** was synthesized as described in bibliography and showed identical spectroscopic data as those reported therein.[3]

3. General procedure for the preparation of [2]rotaxanes 1, 4 and 7

A solution of the thread (1 equiv) and Et_3N (24 equivs) in anhydrous $CHCl_3$ (300 mL) was stirred vigorously whilst two solutions of: a) *p*-xylylene diamine (8 equivs) in anhydrous $CHCl_3$ (20 mL) and b) the corresponding acid dichloride (8 equivs) in anhydrous $CHCl_3$ (20 mL) were simultaneously added over a period of 4 h using motor-driven syringe pumps. After stirring for 4 h more, the resulting suspension was filtered through a Celite™ pad, and the filtrate washed with water (2 x 50 mL), 1 M HCl (2 x 50 mL), a saturated solution of $NaHCO_3$ (2 x 50 mL) and brine (2 x 50 mL). The organic phase was dried over $MgSO_4$ and the solvent removed under reduced pressure. The resulting solid was alternatively washed with Et_2O or subjected to column chromatography (silica gel).

Rotaxane 1a

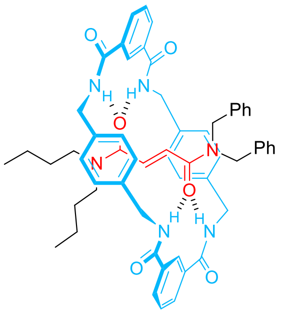

Rotaxane **1a** was synthesized as described in reference 1 and showed identical spectroscopic data as those reported therein.

Rotaxane 1b

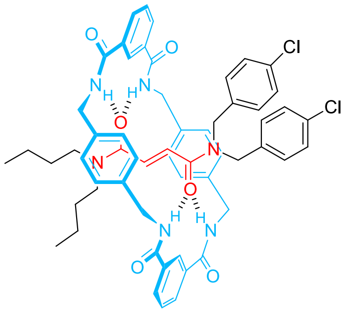

Rotaxane **1b** was synthesized as described in reference 1 and showed identical spectroscopic data as those reported therein.

Rotaxane 1c

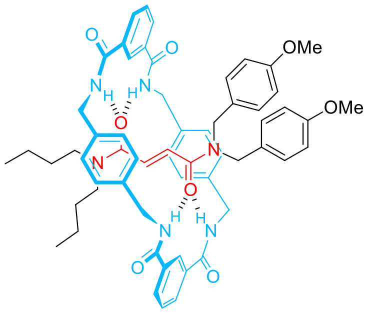

Rotaxane **1c** was synthesized as described in reference 1 and showed identical spectroscopic data as those reported therein.

Rotaxane 1d

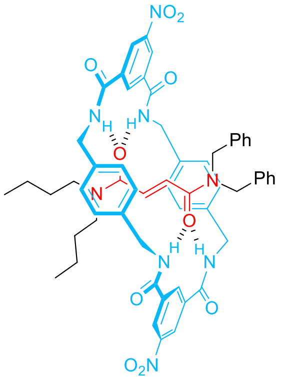

Rotaxane **1d** was obtained by using the previously described method from the thread **T1a** (1.00 g, 2.46 mmol). The crude product was suspended in Et_2O, filtered and dried, to give the title product as a white solid (783 mg, 31%); mp > 300 ºC; ^{1}H NMR (600 MHz, $CDCl_3$, 298 K): δ 9.20 (s, 2H), 9.11 (s, 1H), 8.91 (s, 2H), 8.84 (s, 1H), 7.60 (d, *J*= 8.4 Hz, 2H), 7.54 (d, *J*= 9.0 Hz, 2H), 7.42-7.40 (m, 1H), 7.37-7.34 (m, 2H), 7.28-7.27 (m, 2H), 7.23-7.21 (m, 1H), 7.10 (m, 2H), 6.98 (d, *J*= 7.8 Hz, 4H), 6.89 (d, *J*= 7.8 Hz, 4H), 6.72 (d, *J*= 7.2 Hz, 2H), 6.02 (d, *J*= 14.4 Hz, 1H), 5.91 (d, *J*= 14.4 Hz, 1H), 5.28-5.19 (m, 4H), 4.42 (s, 2H), 4.22 (s, 2H), 3.69 (d, *J*= 13.2 Hz, 2H), 3.56 (d, *J*= 13.8 Hz, 2H), 3.34-3.32 (m, 2H), 2.99-2.97 (m, 2H), 1.60-1.55 (m, 2H), 1.37-1.32 (m, 2H), 1.31-1.26 (m, 2H), 0.94 (t, *J*= 7.5 Hz, 3H), 0.69-0.64 (m, 2H), 0.60 (t, *J*= 7.2 Hz, 3H); ^{13}C NMR (151 MHz, $CDCl_3$, 298 K): δ 166.3 (CO), 165.0 (CO), 163.2 (CO), 162.8 (CO), 149.7 (C), 149.3 (C), 138.2 (C), 137.6 (C), 135.7 (C), 135.4 (C), 135.1 (C), 133.8 (C), 129.7 (CH), 129.5 (CH), 129.2 (CH), 129.2 (CH), 129.1 (CH), 129.1 (CH), 129.0 (CH), 128.9 (CH), 128.9 (CH), 127.2 (CH), 127.1 (CH), 127.0 (CH), 126.6 (CH), 125.7 (CH), 51.7 (CH_2), 51.1 (CH_2), 49.2 (CH_2), 48.2 (CH_2), 43.7 (CH_2), 43.6 (CH_2), 32.4 (CH_2), 30.2 (CH_2), 20.3 (CH_2), 20.0 (CH_2), 13.8 (CH_3), 13.6 (CH_3); HRMS (ESI) calcd for $C_{58}H_{61}N_8O_{10}$ $[M + H]^+$ 1029.4511, found 1029.4493.

Rotaxane 1e

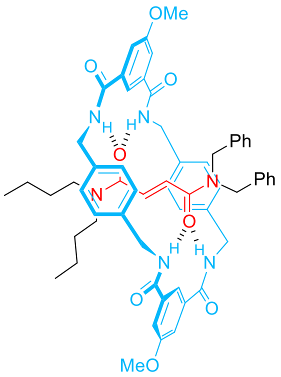

Rotaxane **1e** was obtained by using the previously described method from thread **T1a** (620 mg, 1.53 mmol). The crude product was subjected to column chromatography on silica gel using a $CHCl_3$/MeOH (98/2) mixture as eluent. The solvent was removed under reduced pressure and the resulting residue was suspended in Et_2O, filtered and dried, to give the title product as a white solid (534 mg, 35%); mp > 300 ºC; ^{1}H NMR (600 MHz, $CDCl_3$, 298 K): δ 8.40 (s, 1H), 8.15 (s, 1H), 7.93 (s, 2H), 7.70 (s, 2H), 7.54 (t, *J*= 8.7 Hz, 4H), 7.40-7.35 (m, 5H), 7.24 (t, *J*= 7.2 Hz, 1H), 7.15-7.12 (m, 2H), 6.88 (d, *J*= 7.8 Hz, 4H), 6.82-6.78 (m, 6H), 6.03-5.97 (m, 2H), 5.23-5.14 (m, 4H), 4.51 (s, 2H), 4.29 (s, 2H), 3.96 (s, 3H), 3.90 (s, 3H), 3.67 (d, *J*= 13.8 Hz, 2H), 3.46 (d, *J*= 13.2 Hz, 2H), 3.27-3.24 (m, 2H), 2.91-2.88 (m, 2H), 1.55-1.50 (m, 2H), 1.32-1.26 (m, 2H), 1.23-1.17 (m, 2H), 0.92-0.90 (t, *J*= 7.2 Hz, 3H), 0.64-0.57 (m, 5H); ^{13}C NMR (151 MHz, $CDCl_3$, 298 K): δ 166.3 (CO), 165.2 (CO), 165.1 (CO), 164.8 (CO), 160.9 (C), 160.5 (C), 138.1 (C), 138.0 (C), 135.8 (C), 135.1 (C), 134.7 (C), 134.5 (C), 129.8 (CH), 129.4 (CH), 129.1 (CH), 129.1 (CH), 129.0 (CH), 128.9 (CH), 128.9 (CH), 128.8 (CH), 128.4 (CH), 126.0 (CH), 117.9 (CH), 117.3 (CH), 115.0 (CH), 114.5 (CH), 55.9 (CH_3), 55.8 (CH_3), 51.7 (CH_2), 51.3 (CH_2), 49.1 (CH_2), 48.0 (CH_2), 43.5 (CH_2), 43.3 (CH_2), 32.4 (CH_2), 30.1 (CH_2), 20.3 (CH_2), 19.9 (CH_2), 13.9 (CH_3), 13.7 (CH_3); HRMS (ESI) calcd for $C_{60}H_{67}N_6O_8$ $[M + H]^+$ 999.5020, found 999.5029.

Rotaxane 1f

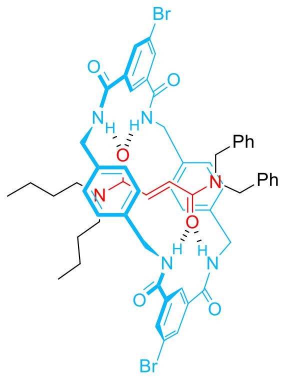

Rotaxane **1f** was obtained by using the previously described method from thread **T1a** (421 mg, 1.04 mmol). The crude product was subjected to column chromatography on silica gel using a $CHCl_3$/MeOH (98/2) mixture as eluent, to give the title product as a white solid (**1f**, 532 mg, 47%); mp 280-282 ºC; ^{1}H NMR (400 MHz, $CDCl_3$, 298 K): δ 8.74 (s, 1H), 8.53 (s, 2H), 8.44 (s, 1H), 8.27 (s, 2H), 7.49 (t, *J*= 8.6 Hz, 4H), 7.42-7.32 (m, 5H), 7.28-7.22 (m, 1H), 7.20-7.14 (m, 2H), 6.91 (d, *J*= 7.7 Hz, 4H), 6.83 (d, *J*= 7.7 Hz, 4H), 6.78-6.74 (m, 2H), 5.99 (d, *J*= 14.5 Hz, 1H), 5.92 (d, *J*= 14.5 Hz, 1H), 5.25-5.12 (m, 4H), 4.49 (s, 2H), 4.28 (s, 2H), 3.66 (d, *J*= 13.5 Hz, 2H), 3.48 (d, *J*= 14.0 Hz, 2H), 3.30-3.24 (m, 2H), 2.95-2.88 (m, 2H), 1.60-1.50 (m, 2H), 1.37-1.17 (m, 4H), 0.92 (t, *J*= 7.3 Hz, 3H), 0.47-0.41 (m, 5H); ^{13}C NMR (101 MHz, $CDCl_3$, 298 K): δ 166.4 (CO), 164.9 (CO), 164.0 (CO), 163.9 (CO), 138.1 (C), 137.9 (C), 135.7 (C), 135.5 (CH), 135.3 (C), 134.9 (CH), 134.1 (C), 129.8 (CH), 129.4 (CH), 129.2 (CH), 129.1 (CH), 129.0 (CH), 128.7 (CH), 125.6 (CH), 124.5 (C), 124.0 (C), 121.1 (CH), 120.7 (CH), 51.7 (CH_2), 51.4 (CH_2), 49.2 (CH_2), 48.1 (CH_2), 43.5 (CH_2), 43.5 (CH_2), 32.4 (CH_2), 30.2 (CH_2), 20.4 (CH_2), 20.0 (CH_2), 13.9 (CH_3), 13.7 (CH_3); HRMS (ESI) calcd for $C_{58}H_{61}Br_2N_6O_6$ $[M + H]^+$ 1095.3014, found 1095.2995.

Rotaxane 1g

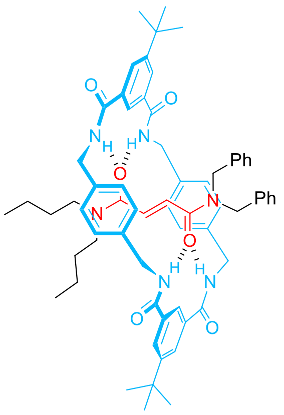

Rotaxane **1g** was obtained by using the previously described method from thread **T1a** (1.00 g, 2.46 mmol). The solid crude was subjected to column chromatography on silica gel using a $CHCl_3/Me_2CO$ (90/10) mixture as eluent, to give the title product as a white solid (955 mg, 37%); mp > 300 ºC; ^{1}H NMR (600 MHz, $CDCl_3$, 298 K): δ 8.67 (s, 1H), 8.44 (s, 2H), 8.35 (s, 1H), 8.26 (s, 2H), 7.58 (d, *J*= 8.4 Hz, 2H), 7.50 (d, *J*= 7.8 Hz, 2H), 7.41-7.37 (m, 5H), 7.18 (t, *J*= 7.5 Hz, 1H), 7.10-7.06 (m, 2H), 6.86 (d, *J*= 7.8 Hz, 4H), 6.84-6.79 (m, 6H), 6.04-6.00 (m, 2H), 5.23 (dd, *J*= 14.1, 9.4 Hz, 2H), 5.10 (dd, *J*= 14.1, 8.6 Hz, 2H), 4.57 (s, 2H), 4.36 (s, 2H), 3.72 (dd, *J*= 14.1,1.4 Hz, 2H), 3.44 (d, *J*= 14.1 Hz, 2H), 3.21-3.17 (m, 2H), 2.86-2.83 (m, 2H), 1.50-1.46 (m, 2H), 1.43 (s, 9H), 1.39 (s, 9H), 1.25-1.18 (m, 2H), 1.16-1.11 (m, 2H), 0.86 (t, *J*= 7.3 Hz, 3H), 0.55 (t, *J*= 7.2 Hz, 3H), 0.47-0.41 (m, 2H); ^{13}C NMR (151 MHz, $CDCl_3$, 298 K): δ 166.4 (CO), 165.9 (CO), 165.7 (CO), 164.7 (CO), 153.4 (C), 152.7 (C), 138.2 (C), 137.9 (C), 135.9 (C), 134.7 (C), 133.2 (C), 133.0 (C), 129.8 (CH), 129.6 (CH), 129.4 (CH), 129.1 (CH), 129.1 (CH), 129.0 (CH), 128.9 (CH), 128.8 (CH), 128.5 (CH), 126.1 (CH), 120.3 (CH), 119.6 (CH), 51.8 (CH_2), 51.6 (CH_2), 49.1 (CH_2), 47.9 (CH_2), 43.6 (CH_2), 43.2 (CH_2), 35.3 (C), 35.2 (C), 32.3 (CH_2), 31.3 (CH_3), 30.0 (CH_2), 20.3 (CH_2), 19.6 (CH_2), 13.8 (CH_3), 13.7 (CH_3); HRMS (ESI) calcd for $C_{66}H_{79}N_6O_6$ $[M + H]^+$ 1051.6061, found 1051.6079.

Rotaxane 1h

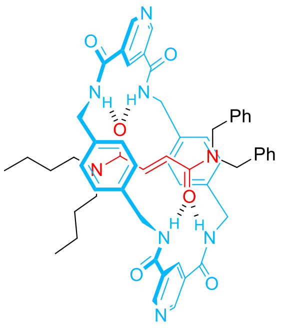

Rotaxane **1h** was obtained by using the previously described method from thread **T1a** (0.50 g, 1.23 mmol). The crude product was subjected to column chromatography on silica gel using a $CHCl_3$/MeOH (95:5) mixture as eluent, to give the title product as a white solid (330 mg, 29%); mp 245-247 ºC; ^{1}H NMR (300 MHz, $CDCl_3$, 298 K): δ 9.53 (s, 2H), 9.29 (s, 2H), 9.07 (s, 1H), 8.72 (s, 1H), 7.46-7.28 (m, 9H), 7.23-7.06 (m, 3H), 7.00-6.84 (m, 8H), 6.78-6.72 (m, 2H), 6.04 (d, *J*= 14.5 Hz, 1H), 5.91 (d, *J*= 14.5 Hz, 1H), 5.24-5.10 (m, 4H), 4.49 (s, 2H), 4.32 (s, 2H), 3.77-3.51 (m, 4H), 3.31-3.23 (m, 2H), 2.98-2.90 (m, 2H), 1.58-1.46 (m, 2H), 1.37-1.17 (m, 4H), 0.91 (t, *J*= 7.3 Hz, 3H), 0.71-0.60 (m, 5H); ^{13}C NMR (75 MHz, $CDCl_3$, 298 K): δ 166.4 (CO), 164.9 (CO), 163.9 (CO), 153.6 (CH), 152.9 (CH), 137.9 (C), 137.8 (C), 135.6 (C), 133.9 (C), 129.7 (CH), 129.3 (CH), 129.3 (CH), 129.2 (CH), 129.1 (CH), 128.7 (CH), 127.9 (C), 127.7 (C), 125.3 (CH), 51.8 (CH_2), 51.5 (CH_2), 49.2 (CH_2), 48.1 (CH_2), 43.5 (CH_2), 32.4 (CH_2), 30.2 (CH_2), 20.3 (CH_2), 20.0 (CH_2), 13.9 (CH_3), 13.7 (CH_3); HRMS (ESI) calcd for $C_{56}H_{61}N_8O_6$ $[M + H]^+$ 941.4709, found 941.4727.

Rotaxane 1i

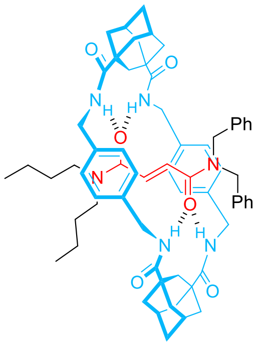

Rotaxane **1i** was obtained by using the previously described method from thread **T1a** (0.50 g, 1.23 mmol). The crude product was subjected to column chromatography on silica gel using a $CHCl_3$/AcOEt (75/25) mixture and subsequently pure AcOEt as eluent, to give the title product as a white solid (160 mg, 12%); mp > 300 °C; ^{1}H NMR (400 MHz, $CDCl_3$, 298 K): δ 7.53-7.33 (m, 8H), 7.23-7.19 (m, 2H), 7.09-6.77 (m, 12H), 5.99 (d, *J*= 14.2 Hz, 1H), 5.90 (d, *J*= 14.2 Hz, 1H), 5.10-4.90 (m, 4H), 4.48 (s, 2H), 4.40 (s, 2H), 3.49-3.29 (m, 6H), 3.20-3.13 (m, 2H), 2.76-2.65 (m, 1H), 2.40-2.00 (m, 10H), 1.95-1.35 (m, 23H), 1.05 (t, *J*= 7.3 Hz, 3H), 1.00 (t, *J*= 7.3 Hz, 3H), 0.68-0.59 (m, 2H); ^{13}C NMR (101 MHz, $CDCl_3$, 298 K): δ 177.3 (CO), 165.7 (CO), 164.7 (CO), 138.2 (C), 137.8 (C), 135.9 (C), 134.5 (C), 129.7 (CH), 129.6 (CH), 129.1 (CH), 129.1 (CH), 129.0 (CH), 128.8 (CH), 128.8 (CH), 127.9 (CH), 126.9 (CH), 51.6 (CH_2), 49.7 (CH_2), 48.8 (CH_2), 48.0 (CH_2), 43.2 (CH_2), 41.7 (C), 41.5 (C), 40.0 (CH_2), 39.5 (CH_2), 39.1 (CH_2), 38.7 (CH_2), 35.2 (CH_2), 32.5 (CH_2), 30.0 (CH_2), 28.2 (CH), 20.8 (CH_2), 20.5 (CH_2), 14.2 (CH_3), 13.9 (CH_3); HRMS (ESI) calcd for $C_{66}H_{83}N_6O_6$ $[M + H]^+$ 1055.6369, found 1055.6361.

Rotaxane 4

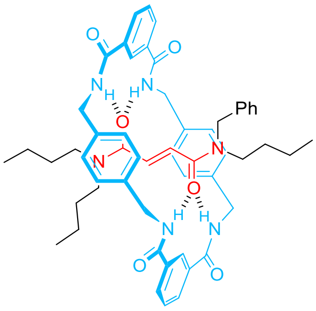

Rotaxane **4** was synthesized as described in reference 1 and showed identical spectroscopic data as those reported therein.

Rotaxane 1a-*d*$_2$

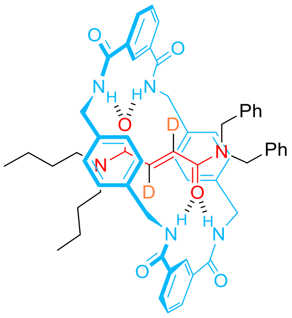

Rotaxane **1a-*d*$_2$** (93% D) was obtained by using the previously described method from thread **T1a-*d*$_2$** (0.50 g, 1.22 mmol). The crude product was subjected to column chromatography on silica gel using a $CHCl_3$/MeOH (98:2) mixture, to give the title product as a white solid (505 mg, 44 %); mp 245-247 ºC; ^{1}H NMR (400 MHz, $CDCl_3$, 298 K): δ 8.81 (s, 1H), 8.53 (s, 1H), 8.39 (d, *J* = 7.8 Hz, 2H), 8.20 (d, *J* = 7.6 Hz, 2H), 7.71 (t, *J* = 7.8 Hz, 1H), 7.60-7.50 (m, 5H), 7.43-7.35 (m, 5H), 7.20 (t, *J* = 7.4 Hz, 1H), 7.12-7.04 (m, 2H), 6.90-6.78 (m, 10H), 5.27-5.07 (m, 4H), 4.56 (s, 2H), 4.34 (s, 2H), 3.71 (d, *J* = 13.9 Hz, 2H), 3.47 (d, *J* = 13.6 Hz, 2H), 3.28-3.14 (m, 2H), 2.95-2.70 (m, 2H), 1.58-1.43 (m, 2H), 1.33-1.11 (m, 4H), 0.88 (t, *J* = 7.3 Hz, 3H), 0.60-0.51 (m, 5H); ^{13}C NMR (75 MHz, $CDCl_3$, 298 K): δ 166.5 (CO), 165.5 (CO), 164.8 (CO), 138.0 (C), 135.9 (C), 134.6 (C), 133.6 (C), 133.3 (C), 132.6 (CH), 132.0 (CH), 129.9 (CH), 129.4 (CH), 129.2 (CH), 129.1 (CH), 128.9 (CH), 128.6 (CH), 125.9 (CH), 122.9 (CH), 122.3 (CH), 51.9 (CH_2), 51.6 (CH_2), 49.2 (CH_2), 48.04 (CH_2), 43.6 (CH_2), 43.3 (CH_2), 32.4 (CH_2), 30.1 (CH_2), 20.4 (CH_2), 19.8 (CH_2), 13.9 (CH_3),

13.7 (CH_3); the signals CD are not observed; HRMS (ESI) calcd for $C_{58}H_{61}D_2N_6O_6$ $[M + H]^+$ 941.4929, found 941.4925.

Rotaxane 4-*d*₂

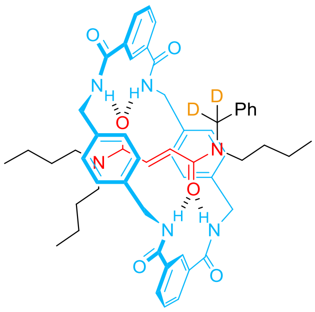

Rotaxane **4-*d*₂** (99% D) was obtained by using the previously described method from thread **T1b-*d*₂** (0.20 g, 0.53 mmol). The crude product was subjected to column chromatography on silica gel using a $CHCl_3$/MeOH (40/1) mixture, to give the title product as a white solid (166 mg, 34 %); mp 266-268 ºC; *mixture of rotamers in $CDCl_3$ at 298K* (72:28); ^1H NMR (300 MHz, $CDCl_3$, 298 K): δ 8.85 (s, 2H, *major rotamer*), 8.57 (s, 2H, *minor rotamer*), 8.40-8.20 (m, 4H), 7.76-7.52 (m, 6H), 7.40-7.15 (m, 5H), 7.08-6.70 (m, 8H), 6.10-5.90 (m, 2H), 5.40-5.10 (m, 4H), 3.80-3.64 (m, 2H), 3.52-3.44 (m, 2H), 3.32-3.20 (m, 2H), 3.14-3.04 (m, 2H), 2.96-2.84 (m, 2H), 1.65-1.45 (m, 2H), 1.35-1.15 (m, 6H), 0.96-0.48 (m, 13H); ^{13}C NMR (75 MHz, $CDCl_3$, 298 K): δ 166.3 (CO), 165.4 (CO), 165.3 (CO), 165.0 (CO), 164.8 (CO), 138.1 (C), 138.1 (C), 138.0 (C), 136.1 (C), 135.0 (C), 133.4 (C), 133.3 (C), 133.2 (C), 132.4 (CH), 132.2 (CH), 132.0 (CH), 129.7 (CH), 129.6 (CH), 129.3 (CH), 129.0 (CH), 129.0 (CH), 128.7 (CH), 128.4 (CH), 128.2 (CH), 125.7 (CH), 122.9 (CH), 122.7 (CH), 122.5 (CH), 49.1 (CH_2), 49.0 (CH_2), 48.0 (CH_2), 43.6 (CH_2), 43.5 (CH_2), 43.3 (CH_2), 32.3 (CH_2), 31.8 (CH_2), 30.1 (CH_2), 30.1 (CH_2), 29.9 (CH_2), 20.3 (CH_2), 19.8 (CH_2), 19.7 (CH_2), 13.9 (CH_3), 13.7 (CH_3); the signal CD_2 is not observed; HRMS (ESI) calcd for $C_{55}H_{63}D_2N_6O_6$ $[M + H]^+$ 907.5086, found 907.5092.

Rotaxane 7a

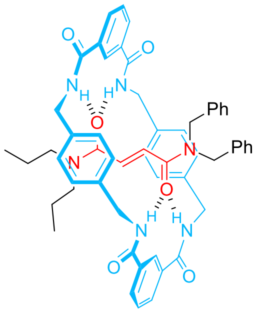

Rotaxane **7a** was synthesized as described in reference 2 and showed identical spectroscopic data as those reported therein.

Rotaxane 7i

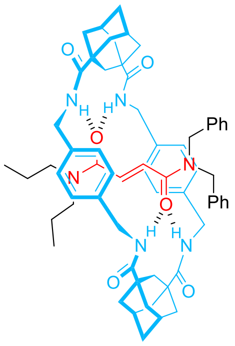

Rotaxane **7i** was obtained by using the previously described method from thread **T1c** (0.50 g, 1.32 mmol). The solid crude was subjected to column chromatography on silica gel using a CH_2Cl_2/AcOEt (1/1) mixture and subsequently AcOEt as eluent, to give the title product as a white solid (149 mg, 11%); mp 228-230 °C; ^{1}H NMR (400 MHz, $CDCl_3$, 298 K): δ 7.52-7.33 (m, 8H), 7.23-7.19 (m, 2H), 7.09-6.77 (m, 12H), 5.99 (d, *J*= 14.2 Hz, 1H), 5.89 (d, *J*= 14.2 Hz, 1H), 5.15-4.90 (m, 4H), 4.48 (s, 2H), 4.40 (s, 2H), 3.50-3.26 (m, 6H), 3.16-3.10 (m, 2H), 2.76-2.60 (m, 1H), 2.40-1.40 (m, 29H), 1.06-1.00 (t, 6H), 0.73-0.57 (m, 2H); ^{13}C NMR (75 MHz, $CDCl_3$, 298 K): δ 177.3 (CO), 165.8 (CO), 164.8 (CO), 138.2 (C), 137.9 (C), 135.9 (C), 134.5 (C), 129.8 (CH), 129.6 (CH), 129.2 (CH), 129.1 (CH), 129.0 (CH), 128.8 (CH), 127.8 (CH), 127.0 (CH), 51.6 (CH), 50.7 (CH_2), 50.3 (CH_2), 49.7 (CH_2), 43.1 (CH_2), 41.6 (CH_2), 39.6 (CH_2), 38.8 (CH_2), 35.2 (CH_2), 28.2 (CH), 23.4 (CH_2), 21.3 (CH_2), 12.2 (CH_3), 12.0 (CH_3); HRMS (ESI) calcd for $C_{64}H_{79}N_6O_6$ $[M + H]^+$ 1027.6056, found 1027.6047.

4. General procedure for the base-catalyzed cyclization of [2]rotaxanes 1, 4 and 7

The corresponding rotaxane (1 equiv) and CsOH (1-5 equivs) in anhydrous DMF were stirred vigorously at room temperature until complete conversion of the starting material. When the reaction was complete, AcOEt (10 mL) was added and the solution was washed with brine (3 x 10 mL). The organic phase was dried over $MgSO_4$ and the solvent removed under reduced pressure. The resulting solid was suspended in Et_2O, filtered and dried, to yield the corresponding interlocked *trans*-β-lactam. When rotaxane **7a** was submitted to the standard cyclization conditions the corresponding lactam *trans*-**8a** was not formed. Instead, only the dethreaded **T1c** and the polyamide macrocycle was obtained.

Interlocked β-lactam *trans*-2a

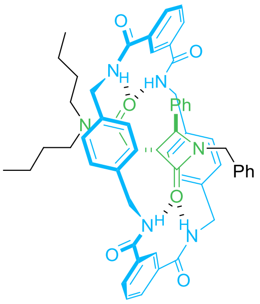

Interlocked lactam **2a** was synthesized as described in reference 1 and showed identical spectroscopic data as those reported therein.

Interlocked β-lactam *trans*-2b

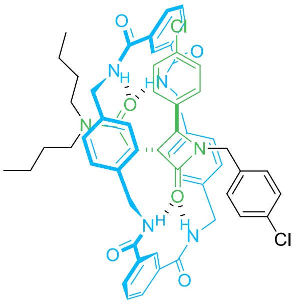

Interlocked lactam **2b** was synthesized as described in reference 1 and showed identical spectroscopic data as those reported therein.

Interlocked β-lactam *trans*-2c

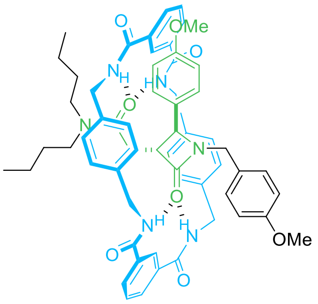

Interlocked lactam **2c** was synthesized as described in reference 1 and showed identical spectroscopic data as those reported therein.

Interlocked β-lactam *trans*-2d

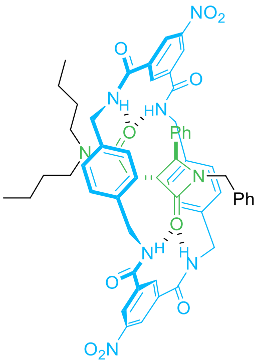

Compound *trans*-**2d** was obtained by using the previously described method from rotaxane **1d** (100 mg, 0.10 mmol) and CsOH (45 mg, 0.33 mmol) in 5 mL of anhydrous DMF during 48 h. White solid (85 mg, 85%); mp > 300 ºC; ^{1}H NMR (400 MHz, $CDCl_3$, 328 K): δ 9.13 (s, 2H), 8.93 (s, 2H), 8.52 (s, 2H), 8.28 (br s, 2H), 7.46-7.40 (m, 3H), 7.24-7.12 (m, 6H), 7.06-6.84 (m, 9H), 6.47-6.41 (m, 2H), 4.90-4.40 (m, 7H), 4.10-3.94 (m, 2H), 3.58 (d, *J*= 14.7 Hz, 1H), 3.54 (d, *J*= 1.7 Hz, 1H), 3.12-2.93 (m, 2H), 2.78-2.62 (m, 2H), 2.06 (dd, *J*= 16.1, 1.9 Hz, 1H), 1.45-1.15 (m, 4H), 0.96-0.87 (m, 4H), 0.70-0.60 (m, 7H), 0.48-0.40 (m, 1H); ^{13}C NMR (101 MHz, $CDCl_3$, 328 K): δ 171.7 (CO), 170.4 (CO), 164.1 (CO), 163.9 (CO), 149.6 (C), 135.8 (C), 135.4 (C), 130.0 (C), 129.8 (CH), 129.7 (CH), 129.4 (CH), 129.3 (CH), 128.9 (CH), 128.8 (CH), 128.6 (CH), 127.5 (CH), 127.1 (CH), 126.2 (CH), 62.2 (CH), 52.8 (CH), 49.6 (CH_2), 47.8 (CH_2), 45.1 (CH_2), 44.6 (CH_2), 31.4 (CH_2),

31.3 (CH_2), 30.4 (CH_2), 20.3 (CH_2), 20.0 (CH_2), 13.5 (CH_3), 13.47 (CH_3); HRMS (ESI) calcd for $C_{58}H_{61}N_8O_{10}$ [M + H]$^+$ 1029.4511, found 1029.4510.

Interlocked β-lactam *trans*-2e

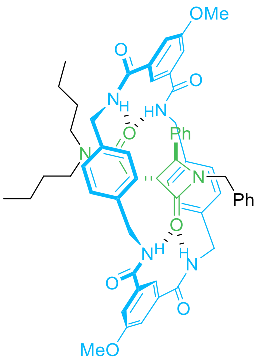

Compound *trans*-**2e** was obtained by using the previously described method from rotaxane **1e** (100 mg, 0.10 mmol) and CsOH (15 mg, 0.10 mmol) in 5 mL of anhydrous DMF during 15 h. White solid (92 mg, 92%); mp > 300 °C; ^{1}H NMR (400 MHz, $CDCl_3$, 328 K): δ 8.15 (s, 2H), 7.90-7.89 (m, 2H), 7.81 (s, 2H), 7.74-7.73 (m, 2H), 7.46-7.38 (m, 3H), 7.22-7.20 (m, 2H), 7.16-7.14 (m, 4H), 7.13-7.08 (m, 1H), 7.06-6.94 (m, 4H), 6.79-6.77 (m, 4H), 6.35-6.32 (m, 2H), 4.84-4.71 (m, 4H), 4.61 (d, *J*=14.5 Hz, 1H), 4.40 (s, 2H), 3.95 (s, 6H), 3.79-3.76 (m, 2H), 3.64 (d, *J*= 14.5 Hz, 1H), 3.58 (s, 1H), 3.06-2.92 (m, 2H), 2.76-2.63 (m, 2H), 2.22-2.14 (m, 1H), 1.45-1.10 (m, 4H), 0.96-0.87 (m, 4H), 0.68-0.56 (m, 7H), 0.48-0.0.39 (m, 1H); ^{13}C NMR (101 MHz, $CDCl_3$, 328 K): δ 171.5 (CO), 170.7 (CO), 166.2 (CO), 166.1 (CO), 160.8 (C), 138.5 (C), 137.8 (C), 135.8 (C), 135.5 (C), 135.4 (C), 129.9 (CH), 129.7 (CH), 129.5 (CH), 129.0 (CH), 128.9 (CH), 128.7 (CH), 127.5 (CH), 118.1 (CH), 117.8 (CH), 116.0 (CH), 62.2 (CH), 55.9 (CH_3), 53.4 (CH), 49.6 (CH_2), 47.6 (CH_2), 45.0 (CH_2), 44.3 (CH_2), 31.5 (CH_2), 31.4 (CH_2), 30.4 (CH_2), 20.3 (CH_2), 19.9 (CH_2), 13.6 (CH_3); HRMS (ESI) calcd for $C_{60}H_{67}N_6O_8$ [M + H]$^+$ 999.5020, found 999.5021.

Interlocked β-lactam *trans*-2f

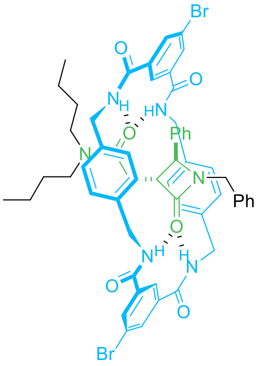

Compound *trans*-**2f** was obtained by using the previously described method from rotaxane **1f** (50 mg, 0.05 mmol) and CsOH (22 mg, 0.15 mmol) in 2.5 mL of anhydrous DMF during 24 h. The crude product was subjected to column chromatography on silica gel using a $CHCl_3$/MeOH (40/1) mixture as eluent to give the corresponding compound as a white solid (37.5 mg, 75%); mp 140-142 ºC; ^{1}H NMR (400 MHz, $CDCl_3$, 328 K): δ 8.46 (s, 2H), 8.27 (s, 2H), 8.19 (br s, 2H), 8.13 (s, 2H), 7.46-7.38 (m, 3H), 7.21-7.06 (m, 7H), 7.02-6.96 (m, 2H), 6.93-6.89 (m, 2H), 6.85-6.76 (m, 4H), 6.41 (t, *J*= 4.8 Hz, 2H), 4.90-4.20 (m, 7H), 3.88 (d, *J*=13.9 Hz, 2H), 3.60 (d, *J*= 14.5 Hz, 1H), 3.52 (d, *J*= 1.5 Hz, 1H), 3.06-2.87 (m, 2H), 2.74-2.56 (m, 2H), 2.05 (d, *J*= 14.5 Hz, 1H), 1.43-1.05 (m, 5H), 0.99-0.85 (m, 3H), 0.69-0.54 (m, 7H), 0.46-0.34 (m, 1H); ^{13}C NMR (101 MHz, $CDCl_3$, 328 K): δ 171.6 (CO), 170.5 (CO), 165.0 (CO), 164.8 (CO), 138.3 (C), 137.7 (C), 135.6 (CH), 135.4 (C), 134.8 (CH), 129.9 (CH), 129.7 (CH), 129.7 (CH), 129.4 (CH), 129.2 (CH), 128.9 (CH), 128.7 (CH), 127.4 (CH), 124.0 (C), 122.2 (CH), 62.1 (CH), 53.0 (CH), 49.63 (CH_2), 47.7 (CH_2), 44.9 (CH_2), 44.4 (CH_2), 31.3 (CH_2), 30.4 (CH_2), 20.3 (CH_2), 20.0 (CH_2), 13.6 (CH_3); HRMS (ESI) calcd for $C_{58}H_{61}Br_2N_6O_6$ $[M + H]^+$ 1095.3014, found 1095.2995.

Interlocked β-lactam *trans*-2g

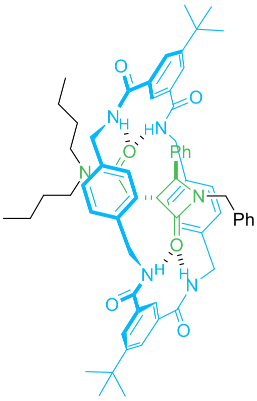

Compound *trans*-**2g** was obtained by using the previously described method from rotaxane **1g** (100 mg, 0.10 mmol) and CsOH (15 mg, 0.10 mmol) in 10 mL of anhydrous DMF during 10 h. White solid. (97 mg, 97%); mp > 300 ºC; ^{1}H NMR (400 MHz, $CDCl_3$, 328 K): δ 8.41 (s, 2H), 8.24 (s, 2H), 8.01 (s, 2H), 7.46-7.40 (m, 3H), 7.22-7.20 (d, 2H), 7.17-7.15 (m, 4H), 7.10-7.07 (m, 1H), 6.95-6.70 (m, 4H), 6.79-6.78 (m, 4H), 6.35-6.33 (m, 2H), 4.91-4.76 (s, 4H), 4.61 (d, *J*=14.5 Hz, 1H), 4.38 (s, 2H), 3.76 (s, 2H), 3.64 (d, *J*= 14.5 Hz, 1H), 3.57 (d, *J*= 1.7 Hz, 1H), 3.03-2.88 (m, 2H), 2.74-2.62 (m, 2H), 2.21 (dd, *J*= 15.2, 1.2 Hz, 1H), 1.44 (s, 18H), 1.43-1.12 (m, 4H), 1.02-0.75 (m, 4H), 0.65-0.55 (m, 6H), 0.53-0.46 (m, 1H), 0.39-0.27 (m, 1H); ^{13}C NMR (101 MHz, $CDCl_3$, 328 K): δ 171.4 (CO), 170.8 (CO), 166.9 (CO), 166.7 (CO), 153.1 (C), 138.5 (C), 137.9 (C), 135.9 (C), 135.5 (C), 133.6 (C), 129.9 (CH), 129.8 (CH), 129.7 (CH), 129.5 (CH), 129.2 (CH), 129.0 (CH), 128.9 (CH), 128.7 (CH), 127.5 (CH), 120.9 (CH), 62.2 (CH), 53.4 (CH), 49.6 (CH_2), 47.6 (CH_2), 45.0 (CH_2), 44.3 (CH_2), 35.4 (C), 31.4 (CH_3+ 2 x CH_2), 31.3 (CH_2), 30.4 (CH_2), 20.2 (CH_2), 19.7 (CH_2), 13.5 (CH_3); HRMS (ESI) calcd for $C_{66}H_{79}N_6O_6$ $[M + H]^+$ 1051.6061, found 1051.6045.

Interlocked β-lactam *trans*-2h

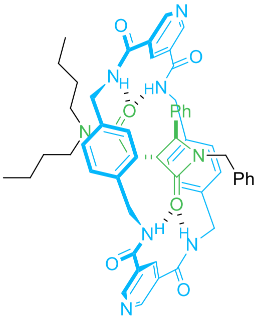

Compound *trans*-**2h** was obtained by using the previously described method from rotaxane **1h** (50 mg, 0.053 mmol) and CsOH (24 mg, 0.16 mmol) in 2 mL of anhydrous DMF. After 48 h at room temperature the conversion was 96%. The reaction crude was subjected to column chromatography on silica gel using a $CHCl_3$/MeOH (20/1) mixture as eluent to give the corresponding compound as a white solid (41 mg, 83%); mp 139-141 ºC; ^{1}H NMR (300 MHz, $CDCl_3$, 318 K): δ 9.44 (d, *J*= 1.5 Hz, 2H), 9.27 (d, *J*= 1.6 Hz, 2H), 8.45-8.42 (m, 2H), 8.13 (s, 2H), 7.50-7.40 (m, 3H), 7.25-6.80 (m, 15H), 6.34 (s, 2H), 4.95-4.30 (m, 7H), 4.96 (dd, *J*=14.1, 3.6 Hz, 2H), 3.55 (d, *J*= 14.5 Hz, 1H), 3.47 (d, *J*= 1.7 Hz, 1H), 3.10-2.85 (m, 2H), 2.78-2.54 (m, 2H), 2.05 (d, *J*= 14.4, 1.0 Hz, 1H), 1.45-0.85 (m, 8H), 0.71-0.60 (m, 7H), 0.39-0.27 (m, 1H); ^{13}C NMR (75 MHz, $CDCl_3$, 298 K): δ 171.3 (CO), 170.2 (CO), 164.7 (CO), 164.4 (CO), 153.4 (CH), 152.5 (CH), 137.6 (C), 135.3 (C), 135.1 (C), 131.1 (CH), 129.9 (CH), 129.7 (CH), 129.5 (CH), 129.4 (CH), 129.0 (CH), 128.7 (CH), 128.2 (CH), 127.3 (CH), 61.9 (CH), 52.7 (CH), 49.6 (CH_2), 47.8 (CH_2), 44.7 (CH_2), 44.2 (CH_2), 31.2 (CH_2), 30.3 (CH_2), 20.2 (CH_2), 19.8 (CH_2), 13.7 (CH_3), 13.6 (CH_3); HRMS (ESI) calcd for $C_{56}H_{61}N_8O_6$ $[M + H]^+$ 941.4709, found 941.4685.

Interlocked β-lactam *trans*-2i

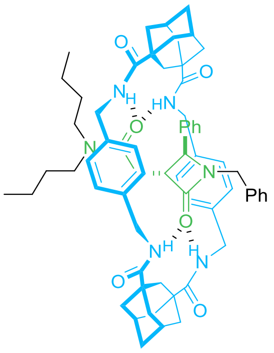

Compound *trans*-**2i** was obtained by using the previously described method from rotaxane **1i** (30 mg, 0.028 mmol) and CsOH (4.3 mg, 0.028 mmol) in 1.5 mL of anhydrous DMF during 1 h. White solid. (29 mg, 99%); mp 149-151 ºC; ^{1}H NMR (400 MHz, $CDCl_3$, 328 K): δ 7.50-7.30 (m, 8H), 7.24-7.20 (m, 2H), 7.13-7.07 (m, 6H), 6.93-6.78 (m, 4H), 5.72 (s, 2H), 4.58-4.47 (m, 5H), 4.16 (d, *J*=14.3 Hz, 2H), 3.63-3.57 (m, 2H), 3.35-3.23 (m, 3H), 3.07-2.97 (m, 2H), 2.93-2.83 (m, 1H), 2.25-1.30 (m, 37H), 1.18 (d, *J*=12.2 Hz, 1H), 1.09 (t, *J*=7.1 Hz, 3H), 0.95 (t, *J*=7.3 Hz, 3H), 0.74 (dd, *J*=16.9, 12.4 Hz, 1H); ^{13}C NMR (101 MHz, $CDCl_3$, 328 K): δ 177.8 (CO), 177.7 (CO), 171.2 (CO), 169.8 (CO), 137.7 (C), 137.5 (C), 136.8 (C), 136.7 (C), 130.2 (CH), 129.7 (CH), 129.6 (CH), 129.4 (CH), 129.3 (CH), 128.7 (CH), 127.9 (CH), 61.9 (CH), 52.6 (CH), 49.3 (CH_2), 47.4 (CH_2), 44.9 (CH_2), 44.4 (CH_2), 41.7 (CH_2), 41.6 (CH_2), 39.0 (CH_2), 38.9 (CH), 38.8 (CH_2), 38.4 (CH_2), 35.5 (CH_2), 32.2 (CH_2), 31.3 (CH_2), 30.6 (CH_2), 28.6 (CH_2), 28.3 (CH_2), 20.9 (CH_2), 20.5 (CH_2), 14.0 (CH_3), 13.9 (CH_3); HRMS (ESI) calcd for $C_{66}H_{83}N_6O_6$ $[M + H]^+$ 1055.6369, found 1055.6364.

Interlocked β-lactam *trans*-5

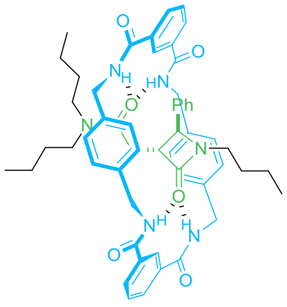

Interlocked lactam **5** was synthesized as described in reference 1 and showed identical spectroscopic data as those reported therein.

Interlocked β-lactam *trans*-2a-*d*$_2$

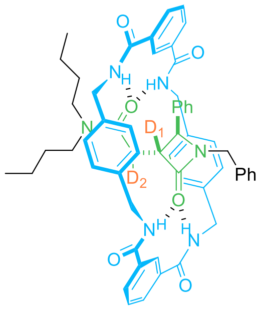

Interlocked β-lactam *trans*-**2a-*d*$_2$** (90% D_1; 20% D_2) was obtained by using the previously described method from rotaxane **1a-*d*$_2$** (47 mg, 0.05 mmol) and CsOH (7.5 mg, 0.05 mmol) in 2 mL of anhydrous DMF during 12 h. White solid (*trans*-**2a-*d*$_1$**, 40 mg, 85%); mp 155-157 ºC; ^{1}H NMR (400 MHz, $CDCl_3$, 298 K): δ 9.00 (br s, 2H), 8.35 (d, *J* = 7.3Hz, 2H), 8.21 (s, 2H), 8.17 (d, *J* = 7.7 Hz, 2H), 7.63 (t, *J* = 7.7 Hz, 2H), 7.50-7.40 (m, 3H), 7.25-7.06 (m, 7H), 6.98-6.92 (m, 4H), 6.73 (bs, 4H), 6.41 (s, 2H), 4.64 (d, *J* = 14.5 Hz, 1H), 3.60 (d, *J* = 14.5 Hz, 1H), 3.57 (s, 1H), 5.20-3.20 (m, 8H), 3.05-2.85 (m, 2H), 2.75-2.55 (m, 2H), 2.10 (d, *J* = 14.6 Hz, 0.75H, related to the non-labelled *trans*-**2a**), 1.45-1.00 (m, 4H), 0.90-0.70 (m, 4H), 0.65-0.40 (m, 7H), 0.26-0.12 (m, 1H); ^{13}C NMR (75 MHz, $CDCl_3$, 298 K): δ 171.4 (CO), 170.6 (CO), 166.4 (CO), 166.3 (CO), 137.8 (C), 135.6 (C), 135.2 (C), 133.7 (C), 132.7 (CH), 132.1 (CH), 129.9 (CH), 129.7 (CH), 129.5 (CH), 129.1 (CH), 129.0 (CH), 128.7 (CH), 127.4 (CH), 123.5 (CH), 61.9 (CH), 52.9 (CH, related to the non-labelled *trans*-**2a**), 49.6 (CH_2), 47.7 (CH_2), 44.8 (CH_2), 44.1 (CH_2), 31.3 (CH_2), 30.3 (CH_2),

20.2 (CH_2), 19.8 (CH_3), 13.7 (CH_3); the signal for CD is not observed; HRMS (ESI) calcd for $C_{58}H_{62}DN_6O_6$ $[M + H]^+$ 940.4866, found 940.4874.

Interlocked β-lactam *trans*-5-*d₁*

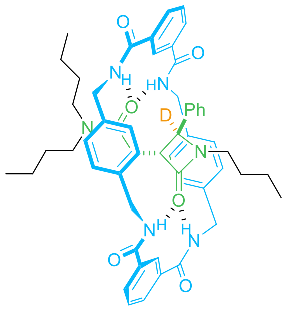

Interlocked β-lactam *trans*-**5**-***d_1*** (80% D) was obtained by using the previously described method from rotaxane **4**-***d_2*** (30 mg, 0.028 mmol) and CsOH (21.5 mg, 0.14 mmol) in 1.5 mL of anhydrous DMF during 12 h. White solid. (29 mg, 99%); mp 149-151 ºC; ^{1}H NMR (300 MHz, $CDCl_3$, 298 K): δ 8.31 (d, *J* = 7.5 Hz, 2H), 8.21 (s, 2H), 8.14 (d, *J* = 7.5 Hz, 2H), 7.59 (t, *J* = 7.5 Hz, 2H), 7.30-6.88 (m, 15H), 6.58 (s, 2H), 5.60-3.80 (m, 8H), 3.32 (d, *J*= 1.1 Hz, 0.20H, related to the non-labelled *trans*-**5a**), 3.15-2.60 (m, 6H), 2.17 (d, *J* = 14.9 Hz, 1H), 1.45-1.05 (m, 10H), 0.90-0.0.75 (m, 5H), 0.65-0.39 (m, 7H), 0.30-0.16 (m, 1H); ^{13}C NMR (75 MHz, $CDCl_3$, 298 K): δ 171.5 (CO), 171.4 (CO), 166.5 (CO), 166.2 (CO), 138.1 (C), 136.3 (C), 133.5 (C), 132.7 (CH), 132.0 (CH), 129.7 (CH), 129.5 (CH), 129.0 (CH), 128.4 (CH), 127.2 (CH), 123.7 (CH), 62.5 (CH, related to the non-labelled *trans*-**5a**), 52.5 (CH), 49.5 (CH_2), 47.7 (CH_2), 44.3 (CH_2), 41.8 (CH_2), 31.8 (CH_2), 31.3 (CH_2), 30.4 (CH_2), 20.5 (CH_2), 20.1 (CH_2), 19.8 (CH_3), 13.7 (CH_3); the signal CD is not observed; HRMS (ESI) calcd for $C_{55}H_{64}DN_6O_6$ $[M + H]^+$ 906.5023, found 906.5031.

Interlocked β-lactam *trans*-8i

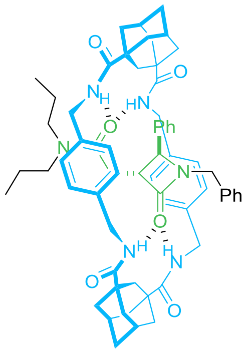

Compound *trans*-**8i** was obtained by using the previously described method from rotaxane **7i** (30 mg, 0.029 mmol) and CsOH (4.4 mg, 0.029 mmol) in 2 mL of anhydrous DMF during 30 min. White solid. (26 mg, 87%); mp 95-97 ºC; ^{1}H NMR (400 MHz, $CDCl_3$, 298 K): δ 7.53-7.35 (m, 7H), 7.26-7.22 (m, 3H), 7.10-7.05 (m, 6H), 6.90-6.84 (m, 4H), 5.72 (s, 2H), 4.65-4.45 (m, 5H), 4.11 (br s, 2H), 3.52 (br s, 2H), 3.32-3.19 (m, 3H), 3.04-2.76 (m, 3H), 2.25-1.45 (m, 33H), 1.09-1.00 (m, 4H), 0.91 (t, J=7.3 Hz, 3H), 0.68 (dd, J=16.6, 12.6 Hz, 1H); ^{13}C NMR (101 MHz, $CDCl_3$, 298 K): δ 177.9 (CO), 177.7 (CO), 171.1 (CO), 169.7 (CO), 137.6 (C), 137.3 (C), 136.7 (C), 130.1 (CH), 129.7 (CH), 129.6 (CH), 129.4 (CH), 129.3 (CH), 128.7 (CH), 127.8 (CH), 61.6 (CH), 52.6 (CH), 51.2 (CH_2), 49.5 (CH_2), 44.8 (CH_2), 44.3 (CH_2), 41.5 (CH_2), 41.4 (CH_2), 38.8 (CH_2), 38.7 (CH_2), 38.5 (CH_2), 38.4 (CH_2), 35.4 (CH_2), 32.1 (CH_2), 28.5 (CH), 28.2 (CH), 22.2 (CH_2), 21.6 (CH_2), 12.0 (CH_3), 11.6 (CH_3); HRMS (ESI) calcd for $C_{64}H_{79}N_6O_6$ $[M + H]^+$ 1027.6056, found 1027.6040.

5. Synthesis of non-interlocked lactams *trans*-3 and *trans*-9 by thermal dethreading

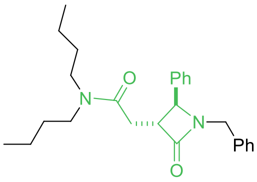

Non-interlocked lactam *trans*-**3** was synthesized as described in reference 1 and showed identical spectroscopic data as those reported therein.

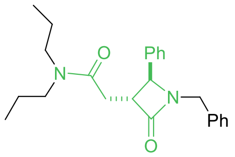

A solution of the rotaxane *trans*-**8i** (24 mg, 0.022 mmol) in DMF (1 mL) was heated at 100 ºC for 2 hours. After this time the solid was filtered and the filtrate was diluted with Et_2O (5 mL). The solution was washed with brine (3 x 5 mL). The organic phase was dried over $MgSO_4$ and the solvent removed under reduced pressure. The residue was subjected to column chromatography on silica gel using a pentane/Et_2O (2/1) mixture as eluent. After removal of the solvent, the title product was isolated as a colorless oil (*trans*-**9**, 8 mg, 96%); ^{1}H NMR (400 MHz, $CDCl_3$, 298 K): δ 7.37-7.26 (m, 8H), 7.17-7.12 (m, 2H), 4.82 (d, *J* = 14.9 Hz, 1H), 4.26 (d, *J* = 1.8 Hz, 1H), 3.80 (d, *J* = 14.9 Hz, 1H), 3.39-3.33 (m, 1H), 3.30-3.12 (m, 4H), 2.97 (dd, *J* = 16.1, 4.0 Hz, 1H), 2.58 (dd, *J* = 16.1, 11.1 Hz, 1H), 1.59-1.42 (m, 4H), 0.90 (t, *J* = 7.4 Hz, 3H), 0.81 (t, *J* = 7.4 Hz, 3H); ^{13}C NMR (101 MHz, $CDCl_3$, 298 K): δ 169.9 (CO), 169.5 (CO), 137.7 (C), 135.9 (C), 128.9 (CH), 128.8 (CH), 128.6 (CH), 128.3 (CH), 127.8 (CH), 127.0 (CH), 61.4 (CH), 56.5 (CH), 49.8 (CH_2), 47.6 (CH_2), 44.6 (CH_2), 32.4 (CH_2), 22.3 (CH_2), 21.0 (CH_2), 11.5 (CH_3), 11.3 (CH_3); HRMS (ESI) calcd for $C_{24}H_{31}N_2O_2$ $[M + H]^+$ 379.2380, found 379.2386.

6. One-pot synthesis of lactam *trans*-9

A solution of the rotaxane **7i** (1 equiv) and CsOH (1 equiv) in anhydrous DMF was stirred vigorously at room temperature for 30 min. After this time the reaction mixture was neutralized by the addition of an aqueous solution of HCl 1 M (1 equiv) and was heated at 100 ºC for 2 hours more. After this time the solid (macrocycle) was filtered and the filtrate was diluted with Et_2O (5 mL). The solution was washed with brine (3 x 5 mL). The organic phase was dried over $MgSO_4$ and the solvent removed under reduced pressure. The residue was subjected to column chromatography on silica gel using a pentane/Et_2O (2/1) mixture as eluent. After removal of the solvent, the title product was isolated as colorless oil (*trans*-**9**, 8 mg, 97%).

7. Synthesis of β-lactams 3 and 9 from threads T1a and T1c

Diastereomeric mixture of *trans*-3 and *cis*-3

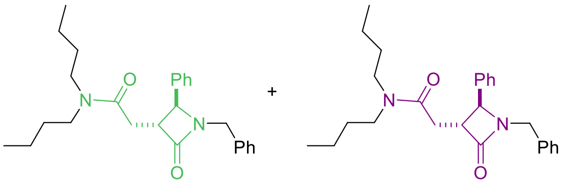

trans-**3** *cis*-**3**

A solution of the thread **T1a** (25 mg, 0.062 mmol) and CsOH (9.2 mg, 0.062 mmol) in anhydrous DMF (2 mL) was stirred vigorously at room temperature during 24 h. After this time Et_2O (10 mL) was added and the solution was washed with brine (4 x 10 mL). The organic phase was dried over $MgSO_4$ and the solvent removed under reduced pressure. The resulting residue was subjected to column chromatography on silica gel using a hexane/AcOEt (2/1) mixture as eluent. A diastereomeric mixture of *trans*-**3** and *cis*-**3** (d.r. 2.7:1) was obtained (8.75 mg, 35%).

Diastereomeric mixture of *trans*-9 and *cis*-9 mixture

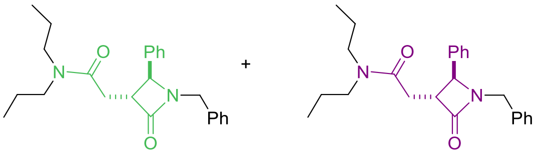

trans-**9** *cis*-**9**

A solution of the thread **T1c** (50 mg, 0.13 mmol) and CsOH (19 mg, 0.13 mmol) in anhydrous DMF (4 mL) was stirred vigorously at room temperature during 20 h. After this time Et_2O (10 mL) was added and the solution was washed with brine (4 x 10 mL). The organic phase was dried over $MgSO_4$ and the solvent removed under reduced pressure. The resulting residue was subjected to column chromatography on silica gel using a hexane/AcOEt (1/1) mixture as eluent. A diastereomeric mixture of *trans*-**9** and *cis*-**9** (d.r. 2.9:1) was obtained (15 mg, 30%).

8. Kinetic studies for the cyclization of rotaxane 1a

The base-catalyzed cyclization reactions of rotaxane **1a** in the presence of of CsOH were followed over time. The conversion percentage of each reaction was plotted *versus* time.

Concentration effect:

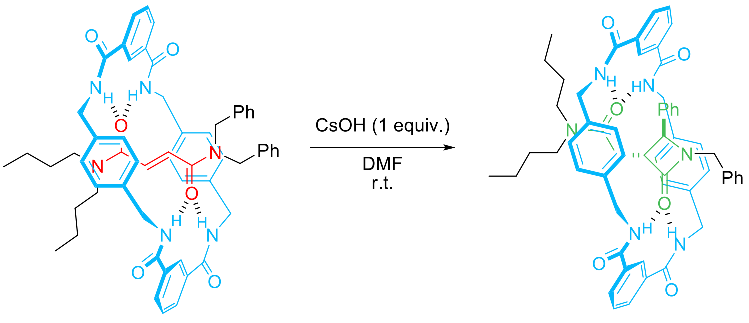

Rotaxane **1a** (0.05 mmol) and CsOH (0.05 mmol, 1 equiv.) were dissolved in anhydrous DMF (2 mL, 25 mM; or 5 mL, 10 mM). Different aliquots (0.1/0.2 mL) were taken over time.

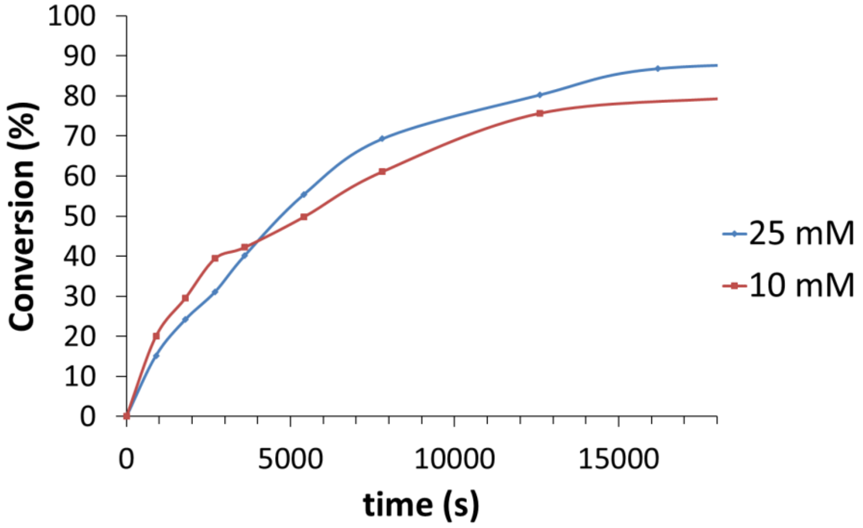

Figure S1. Cyclization reaction of rotaxane **1a** (25 mM and 10 mM) in the presence of CsOH (1 equiv) followed over time.

Effect of the number of equivalents of CsOH:

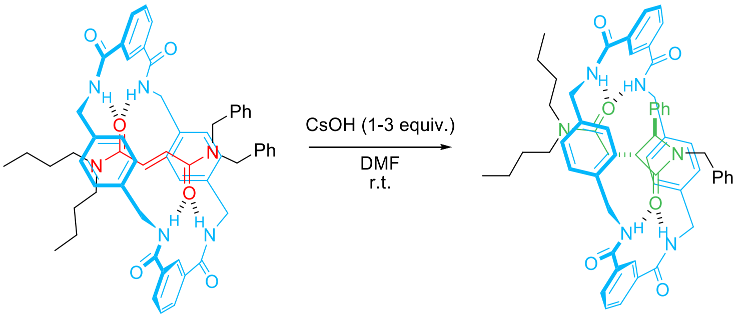

1a *trans*-**2a**

Rotaxane **1a** (0.05 mmol) and CsOH (0.05 or 0.15 mmol) were dissolved in anhydrous DMF (2 mL, 25 mM). Different aliquots (0.2 mL) were taken over time and analyzed by ^{1}H NMR.

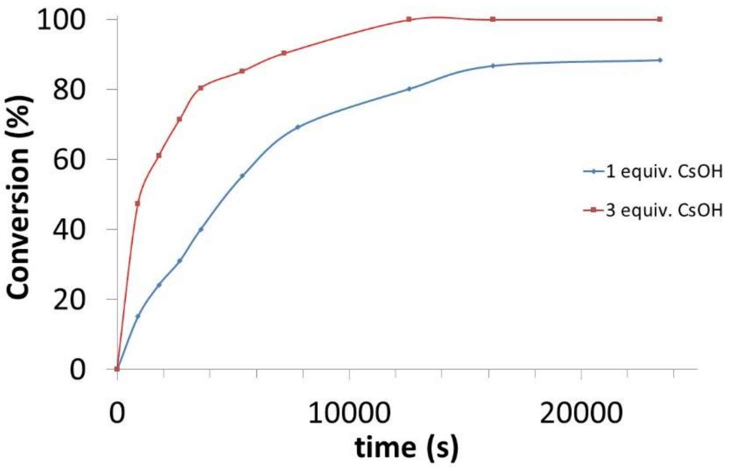

Figure S2. Cyclization reaction of rotaxane **1a** (25 mM) in the presence of CsOH (1 equiv. or 3 equiv.) followed over time.

9. Kinetic measurements for the calculation of the dethreading constant rates of the interlocked fumaramides 1a and 1d-h

For each of the rotaxanes **1**, the rate constant *k* of the dethreading reaction have been calculated at room temperature, following the disappearing of the rotaxane and formation of the free thread during time by ^{1}H-NMR experiments. Measurements were carried out inside the NMR instrument. For the evaluation of the data, the integration of as many signals as possible was averaged in order to reduce experimental error. The rate constant, half-life time and the free energy ΔG‡ of each process were determined by using the Eyring equation.

Typical procedure: Rotaxanes **1** (0.005 mmol) were dissolved in DMF-d_7 (0.4 mL, 12.5 mM) and introduce in an NMR tube. The reaction was followed over time maintaining the sample at room temperature.

Dethreading reaction of rotaxane 1a

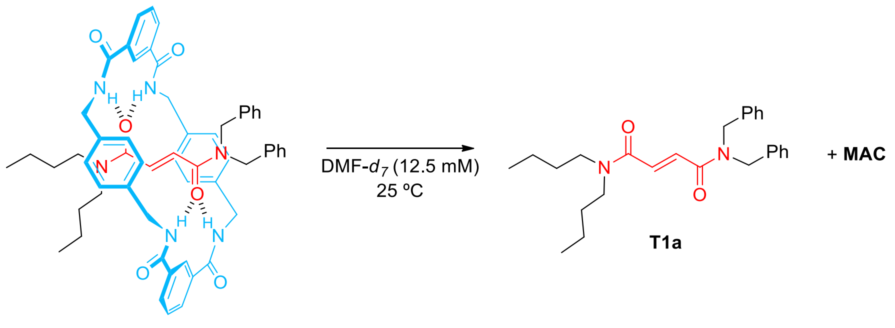

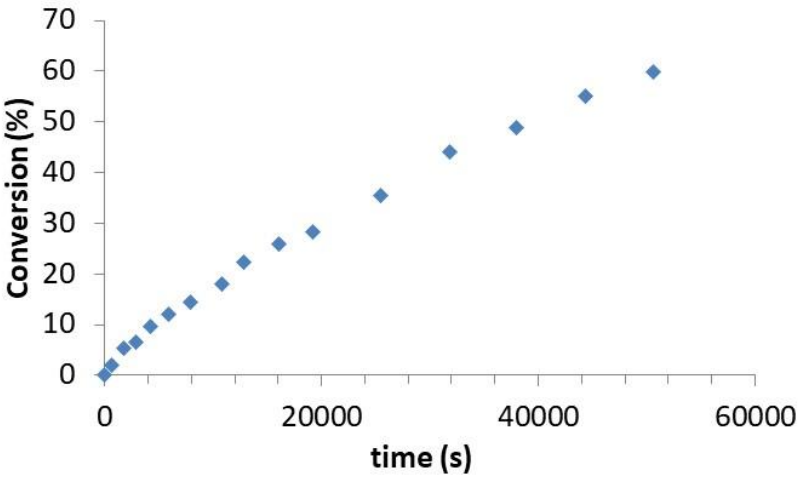

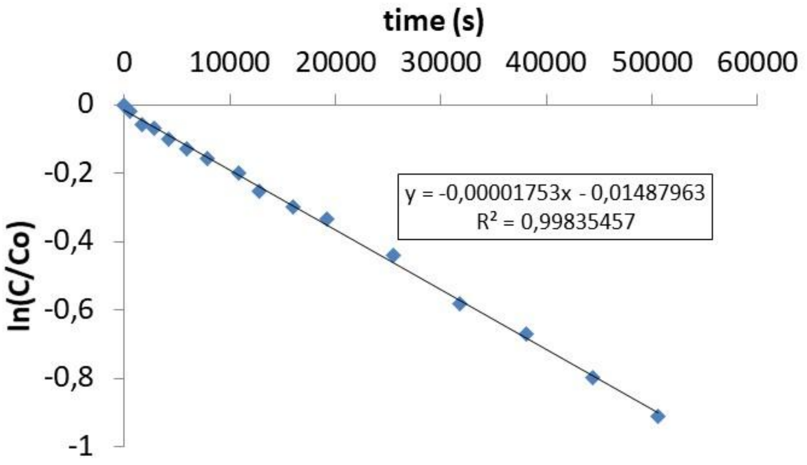

$t_{1/2}$ = 39540 ± 428 seconds

$k = (1.75 \pm 0.2) \times 10^{-5}\ s^{-1}$

$\Delta G^{\ddagger} = 100.12 \pm 0.03\ kJ \cdot mol^{-1}$

Dethreading reaction of rotaxane 1d

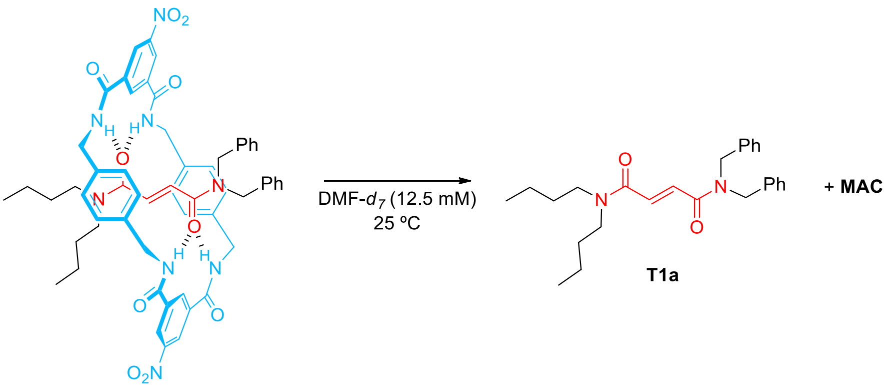

1d

T1a

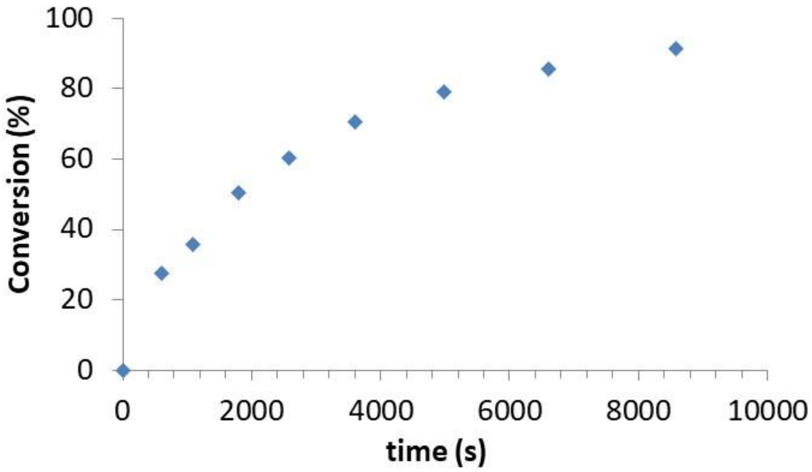

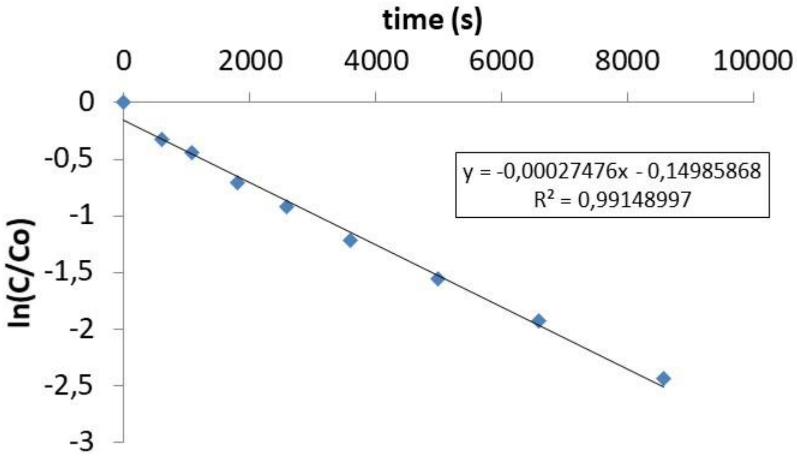

$t_{1/2}$ = 2522 ± 85 seconds

$k = (2.75 \pm 0.10) \times 10^{-4}\ s^{-1}$

$\Delta G^{\ddagger} = 93.30 \pm 0.08\ kJ{\cdot}mol^{-1}$

Dethreading reaction of rotaxane 1e

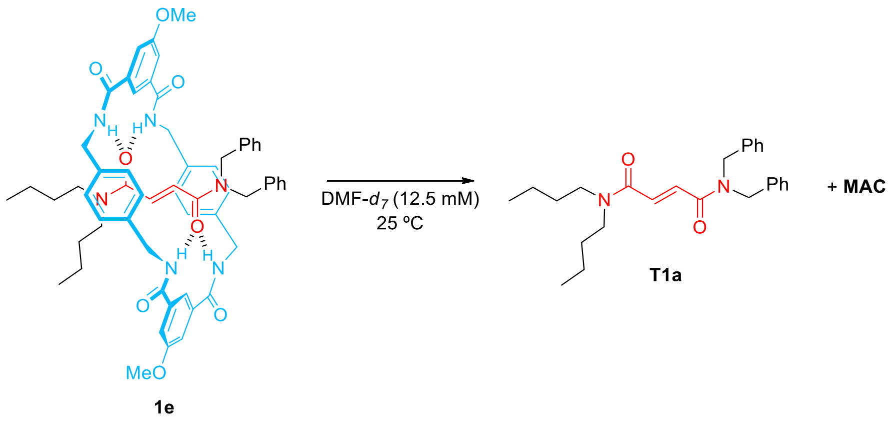

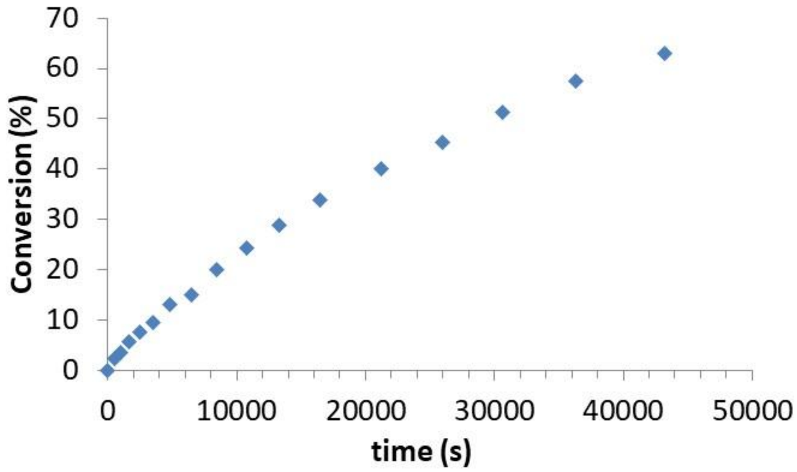

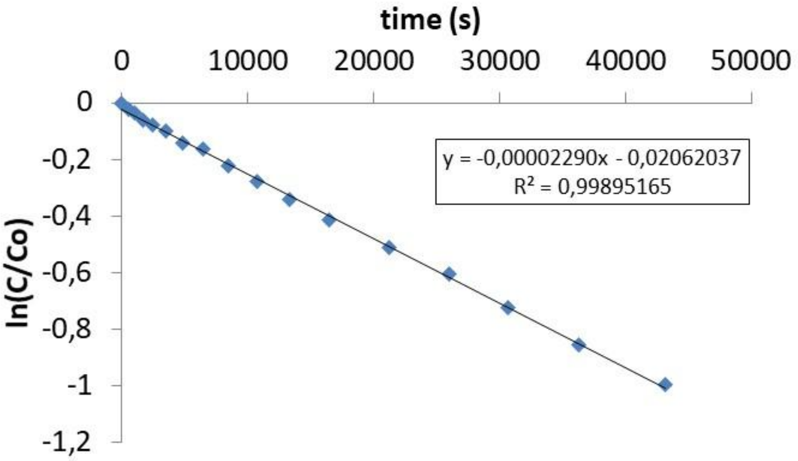

$t_{1/2}$ = 30268 ± 248 seconds

$k = (2.29 \pm 0.02) \times 10^{-5}\ s^{-1}$

$\Delta G\ddagger = 99.46 \pm 0.02\ kJ\cdot mol^{-1}$

Dethreading reaction of rotaxane 1f

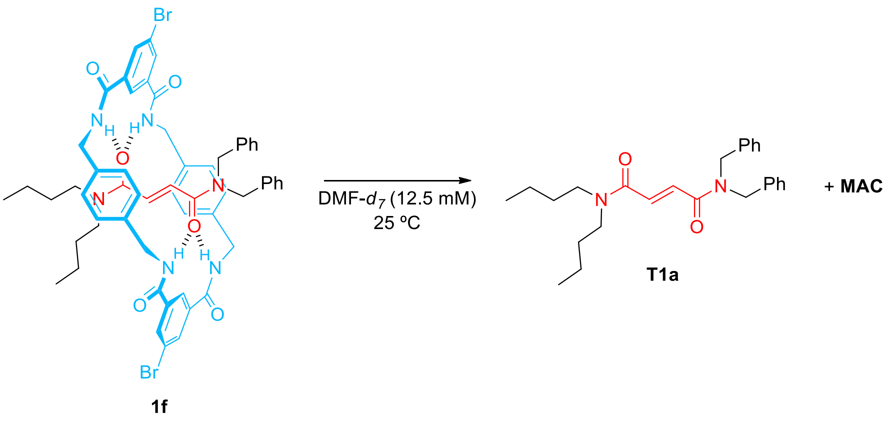

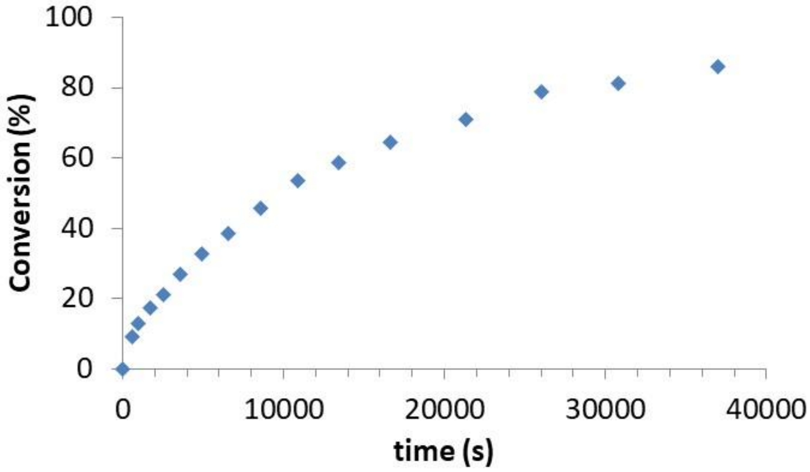

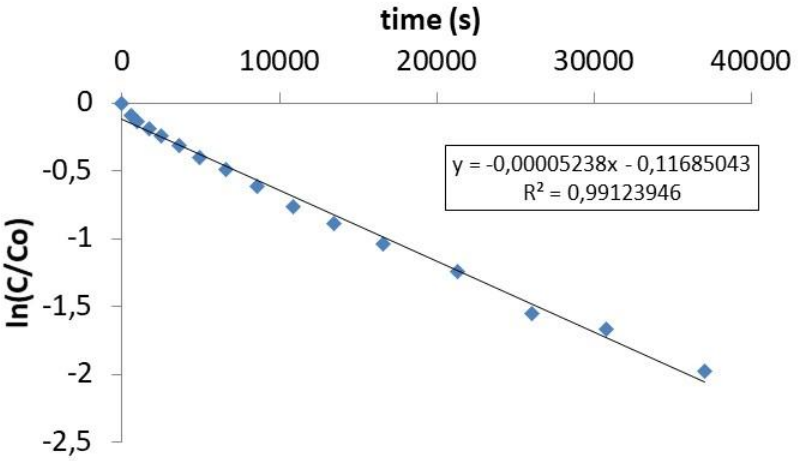

$t_{1/2}$ = 13233 ± 325 seconds

k = (5.24 ± 0.13) x 10^{-5} s^{-1}

ΔG‡ = 97.41 ± 0.06 kJ·mol^{-1}

Dethreading reaction of rotaxane 1g

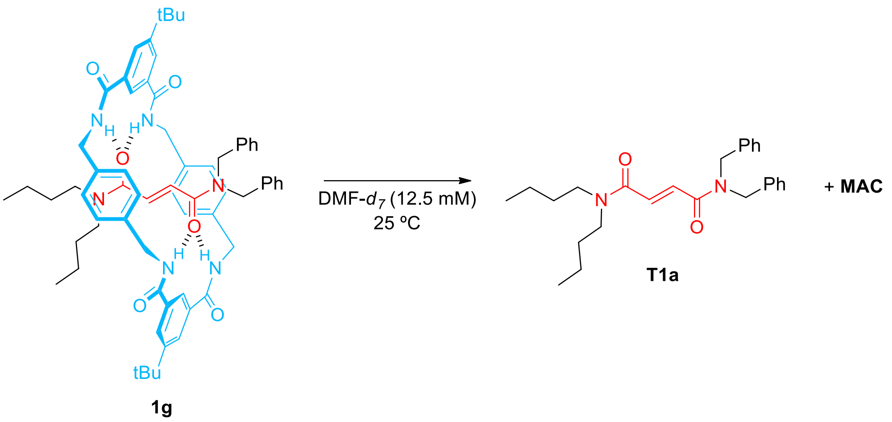

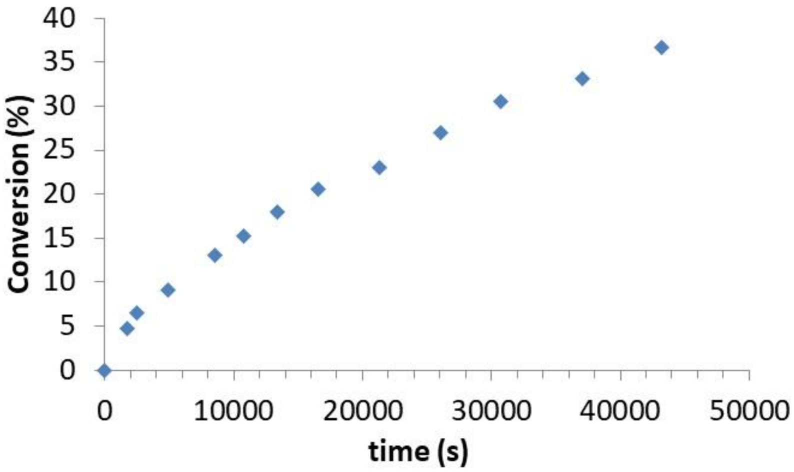

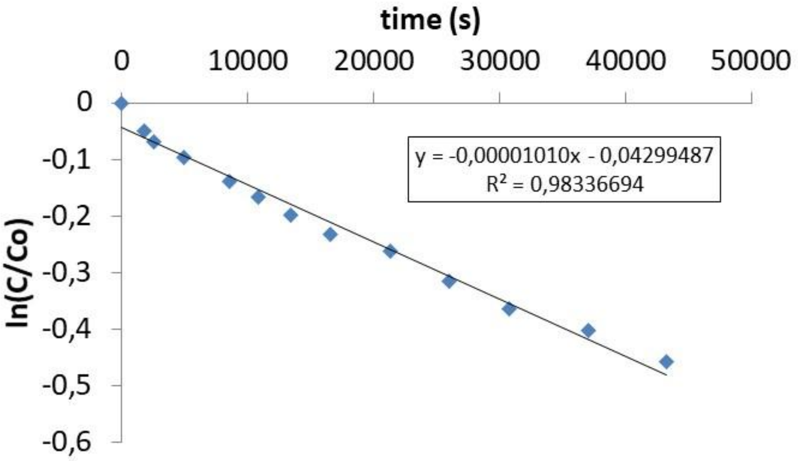

$t_{1/2}$ = 686284 ± 25893 seconds

$k = (1.01 \pm 0.04) \times 10^{-5}\ s^{-1}$

$\Delta G‡ = 107.19 \pm 0.09\ kJ \cdot mol^{-1}$

Dethreading reaction of rotaxane 1h

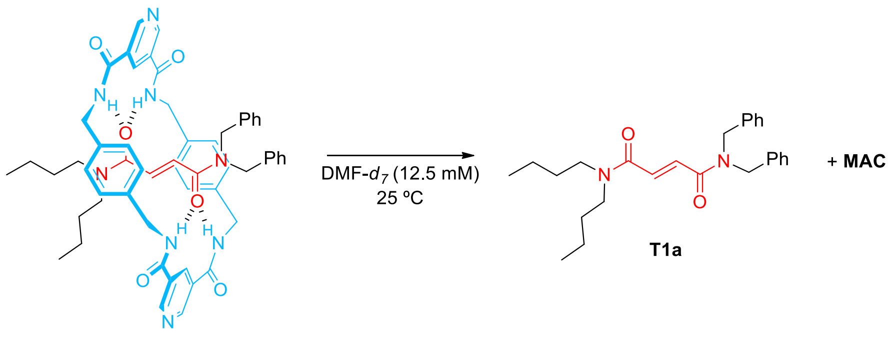

1h

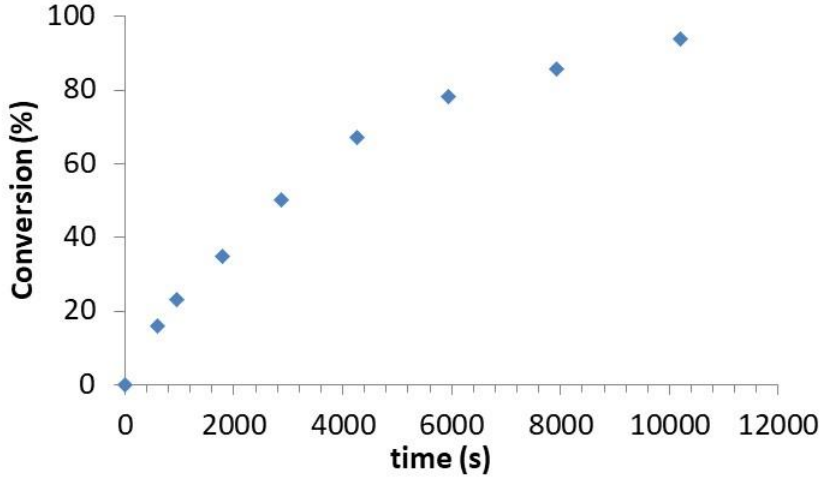

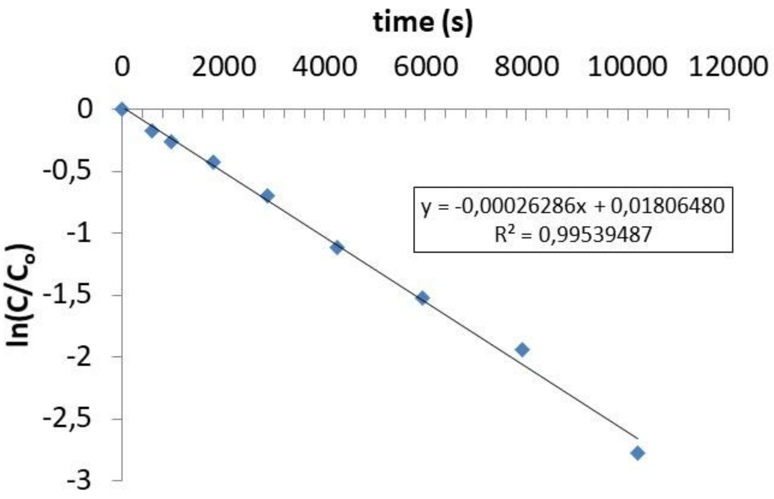

$t_{1/2}$ = 2636 ± 66 seconds

k = (2.63 ± 0.07) x 10^{-4} s^{-1}

ΔG‡ = 93.41 ± 0.06 kJ·mol^{-1}

10. Calculation of the rate constants for the CsOH-catalyzed intramolecular cyclization of interlocked fumaramides 1 and 4

For rotaxanes **1a-c, 1e-i**, **1a-*d*$_2$, 4** and **4-*d*$_2$** the rate constants *k* of their cyclization reactions have been calculated following the disappearance of the starting material and the formation of the interlocked lactam over time by ^{1}H-NMR experiments. For the evaluation of the data, the integration values of as many signals as possible were averaged in order to reduce the experimental error. The rate constant, half-life time and the free energy ΔG‡ of each process were determined by using the Eyring equation. The experiments for rotaxanes **1a-i** were run in duplicate and the media of the reaction constant were used for further discussions in the main text.

Observations:

The reaction of rotaxane **1d** was followed over time, although most of the starting material was dethreaded before the cyclization occurred, being impossible an accurated measuring of the conversion percentage.

For rotaxane **1g** the reaction was carried out under more diluted conditions (5 mL of DMF, 10 mM) due to solubility issues.

In the reaction of rotaxane **1f** and **1h** a noticeable amount of thread **T1a** was observed, which was increasing over time. In those cases the amount of free thread was taken in account for the mathematical treatment for the calculation of the conversion.

In the reaction of rotaxanes **4** and **4-*d*$_2$**, the employment of 5 equivalents of CsOH was required.

Typical procedure: Rotaxanes **1a-c, 1e-i 1a-*d*$_2$, 4** and **4-*d*$_2$** (0.05 mmol) and CsOH (0.05 mmol) were dissolved in anhydrous DMF (2 mL, 25 mM; or 5 mL, 10 mM). The reaction mixture was stirred vigorously at room temperature. Different aliquots were taken over time. Each aliquot was diluted with AcOEt (3 mL) and washed with brine (3 x 5 mL). The organic phase was dried over $MgSO_4$ and the solvent removed under reduced pressure. The resulting residue was analyzed by ^{1}H NMR for the calculation of the percentage of conversion. We integrate as many signals as possible in order to reduce experimental error.

Cyclization reaction of rotaxane 1a

Run 1

Variation of the cyclization percentage was measured over time using ^{1}H NMR spectroscopy (400 MHz, $CDCl_3$). From the corresponding data, reaction constant k, half-life time $t_{1/2}$ and free energy ΔG‡ were obtained.

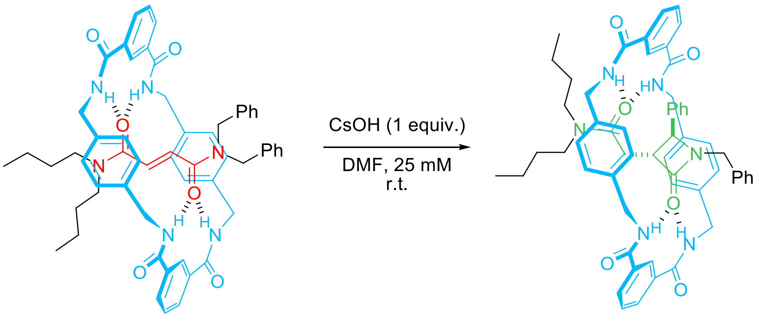

1a *trans*-**2a**

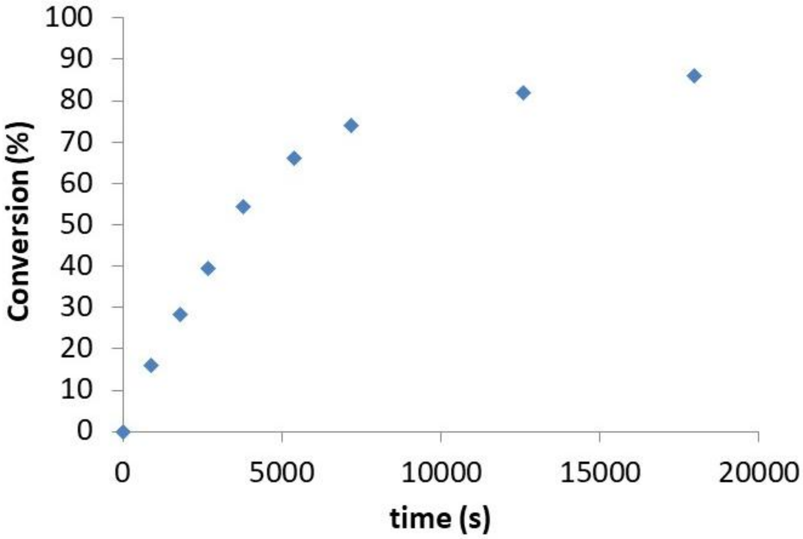

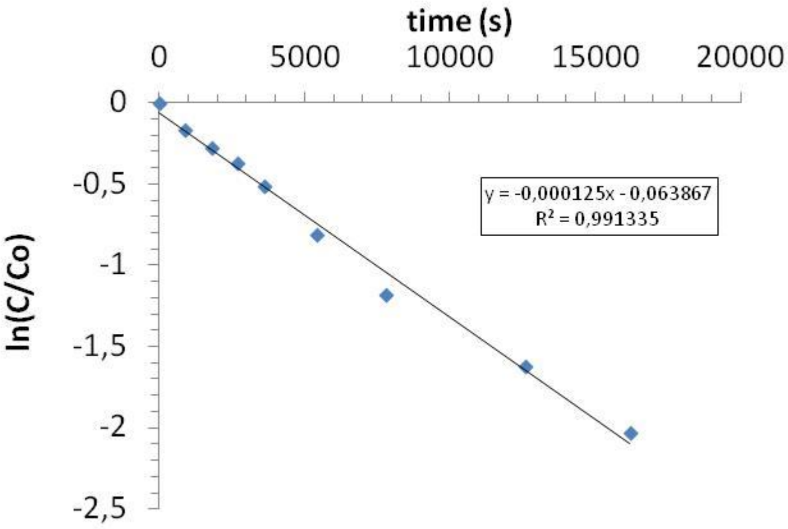

$t_{1/2} = 5545 \pm 172$ seconds

$k = (1.25 \pm 0.04) \times 10^{-4}\ s^{-1}$

$\Delta G^{‡} = 95.25 \pm 0.07\ kJ{\cdot}mol^{-1}$

Run 2

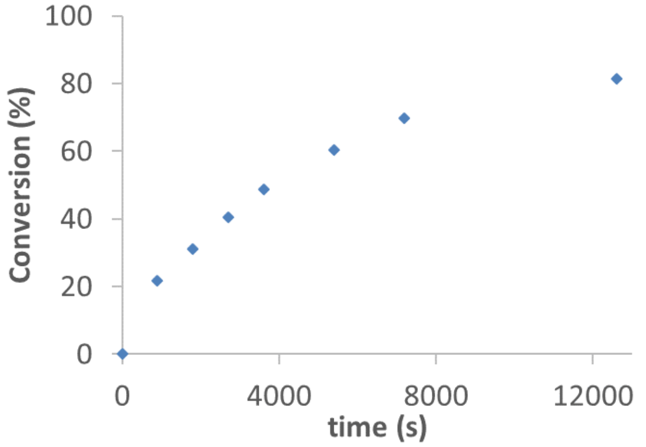

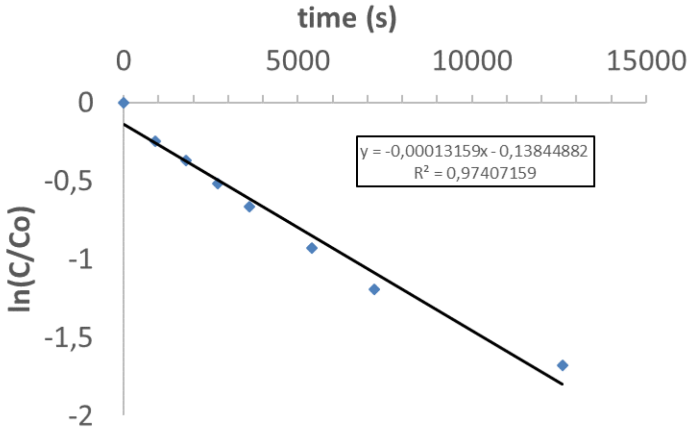

$t_{1/2}$ = 5267 ± 351 seconds

k = (1.32 ± 0.09) x 10^{-4} s^{-1}

ΔG‡ = 95.13 ± 0.17 kJ·mol^{-1}

Cyclization reaction of rotaxane 1a with 3 equivalents of CsOH

Variation of the cyclization percentage was measured over time using ^{1}H NMR spectroscopy (400 MHz, $CDCl_3$). From the corresponding data, reaction constant k, half-life time $t_{1/2}$ and free energy ΔG‡ were obtained.

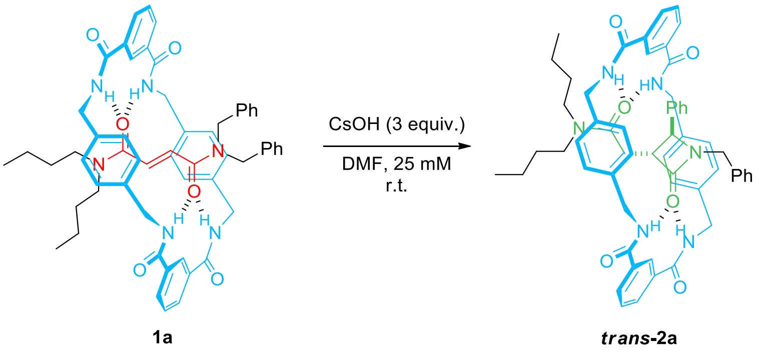

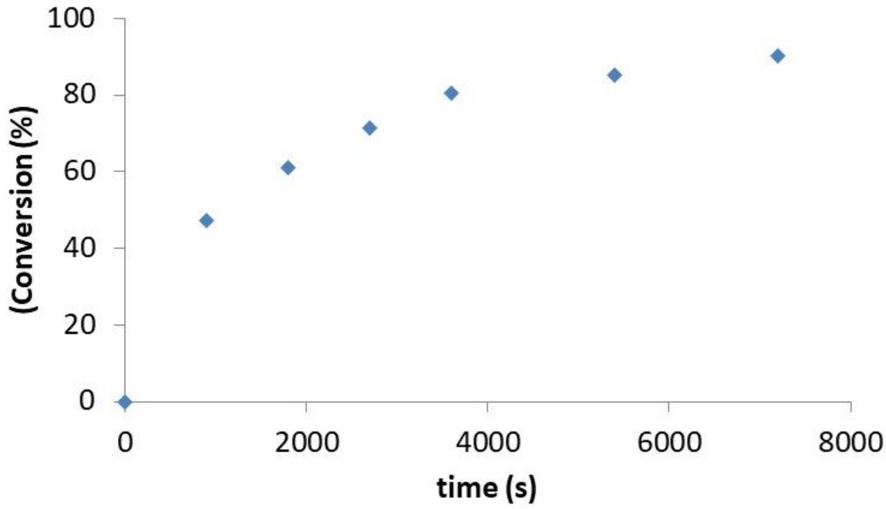

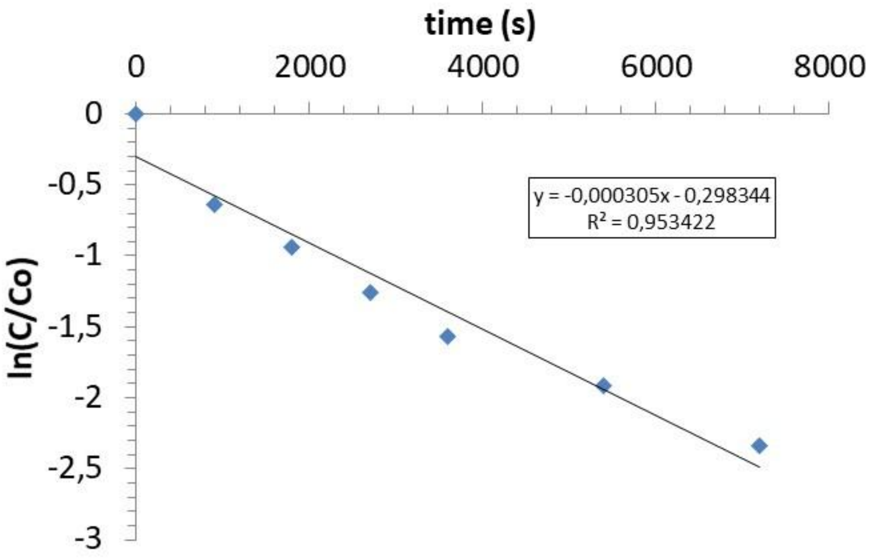

$t_{1/2}$ = 2272 ± 201 seconds

k = (3.05 ± 0.30) x 10^{-4} s^{-1}

ΔG‡ = 93.04 ± 0.23 kJ·mol^{-1}

Cyclization reaction of rotaxane 1b

Run 1

Variation of the cyclization percentage was measured over time using ^{1}H NMR spectroscopy (400 MHz, $CDCl_3$). From the corresponding data, reaction constant k, half-life time $t_{1/2}$ and free energy ΔG‡ were obtained.

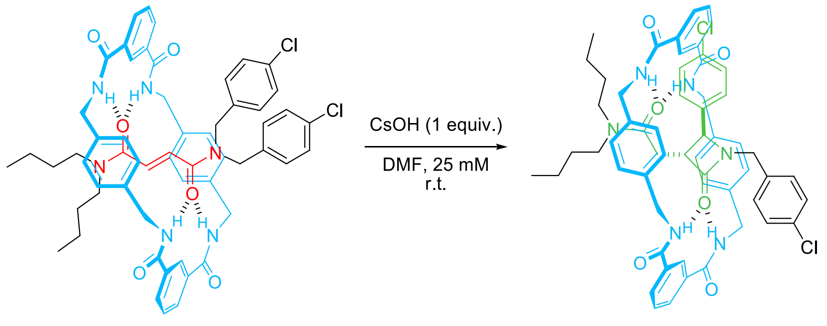

1b *trans*-**2b**

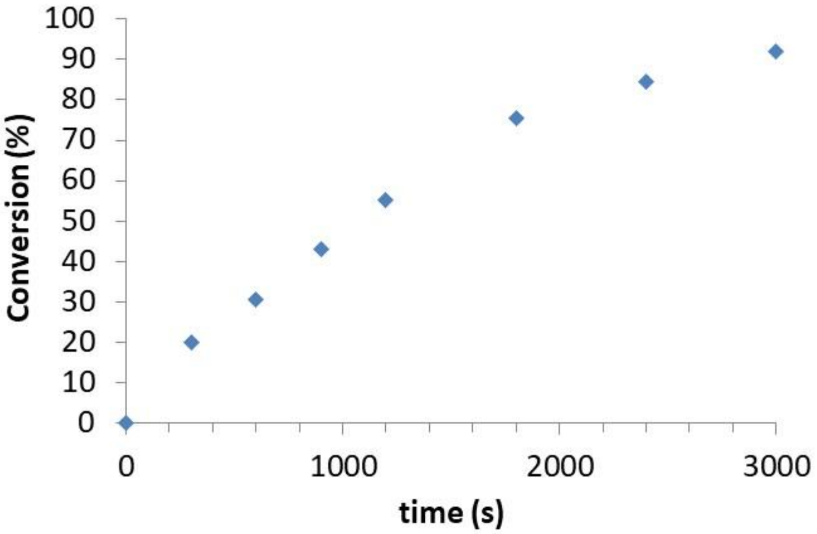

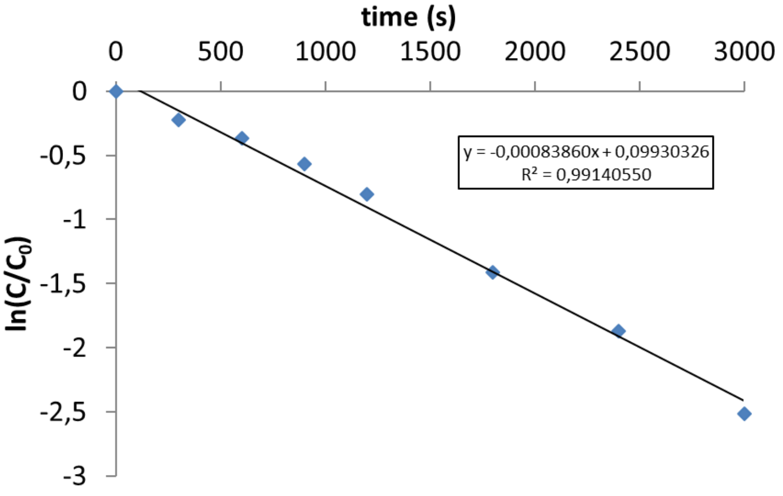

$t_{1/2}$ = 826 ± 30 seconds

k = (8.39 ± 0.32) x 10^{-4} s^{-1}

ΔG‡ = 90.54 ± 0.10 kJ·mol^{-1}

Run 2

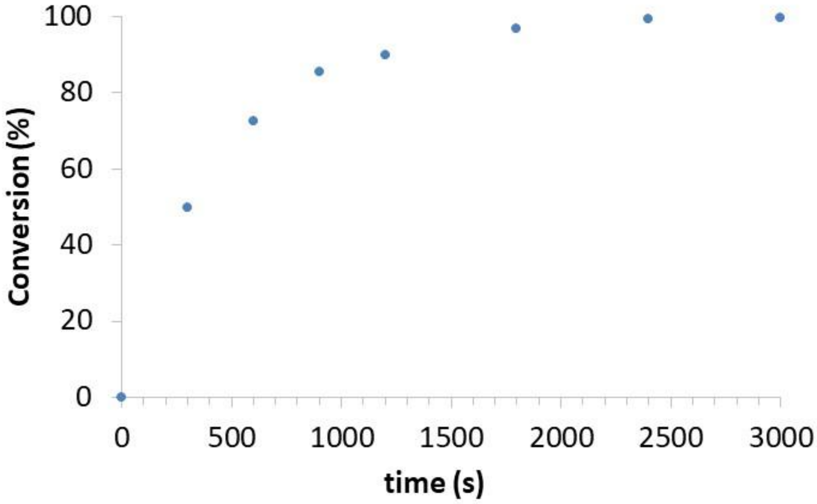

v

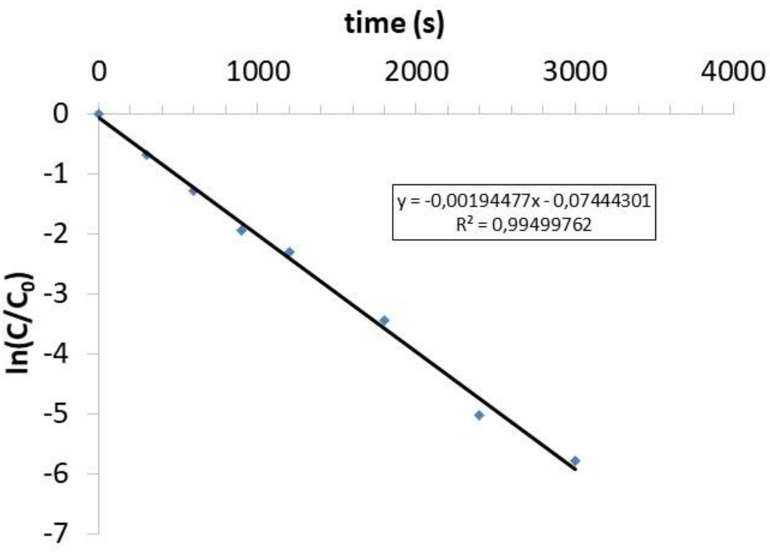

$t_{1/2}$ = 356 ± 18 seconds

k = (1.95 ± 0.11) x 10^{-3} s^{-1}

ΔG‡ = 88.45 ± 0.13 kJ·mol^{-1}

Cyclization reaction of rotaxane 1c

Run 1

Variation of the cyclization percentage was measured over time using ^{1}H NMR spectroscopy (400 MHz, $CDCl_3$). From the corresponding data, reaction constant *k*, half-life time $t_{1/2}$ and free energy ΔG‡ were obtained.

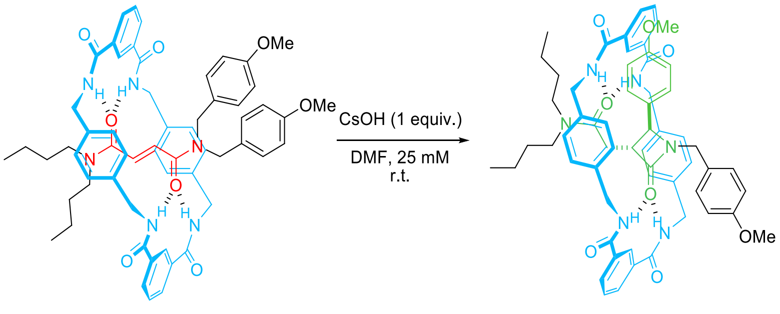

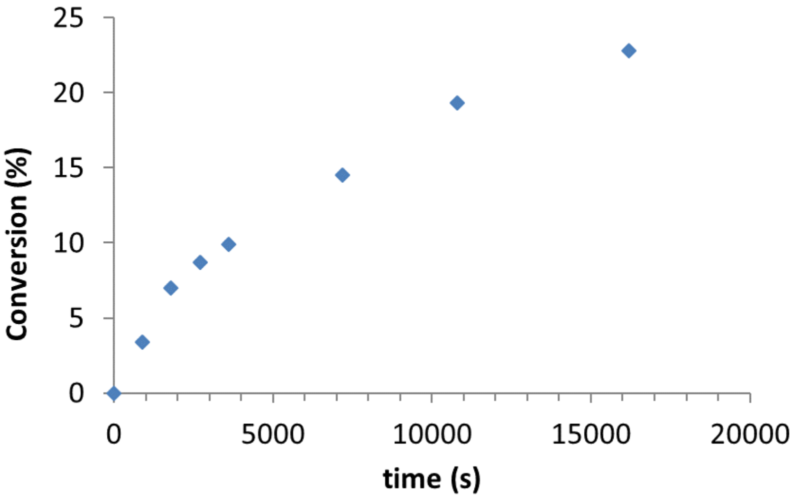

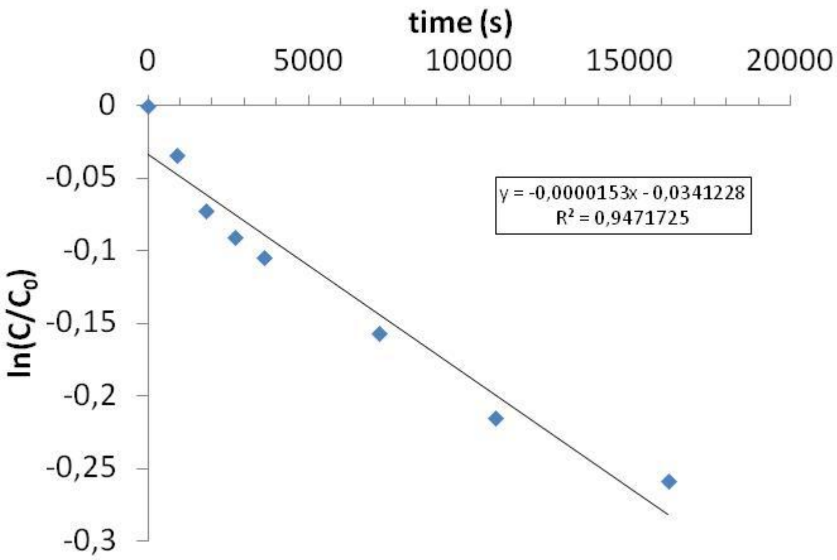

$t_{1/2}$ = 45392 ± 3980 seconds

$k = (1.53 \pm 0.15) \times 10^{-5}\ s^{-1}$

$\Delta G^{\ddagger} = 100.46 \pm 0.20\ kJ \cdot mol^{-1}$

Run 2

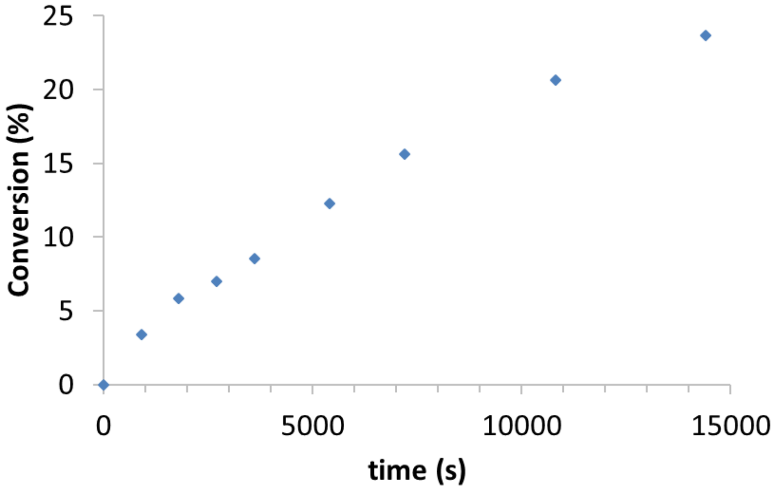

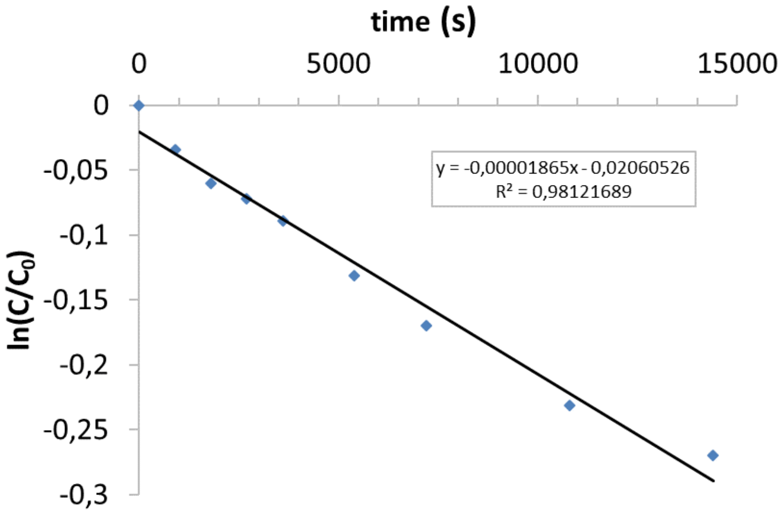

$t_{1/2}$ = 37166 ± 1847 seconds

$k = (1.87 \pm 0.10) \times 10^{-5}\ s^{-1}$

$\Delta G‡ = 99.96 \pm 0.12\ kJ \cdot mol^{-1}$

Cyclization reaction of rotaxane 1e

Run 1

Variation of the cyclization percentage was measured over time using ^{1}H NMR spectroscopy (400 MHz, $CDCl_3$). From the corresponding data, reaction constant k, half-life time $t_{1/2}$ and free energy ΔG‡ were obtained.

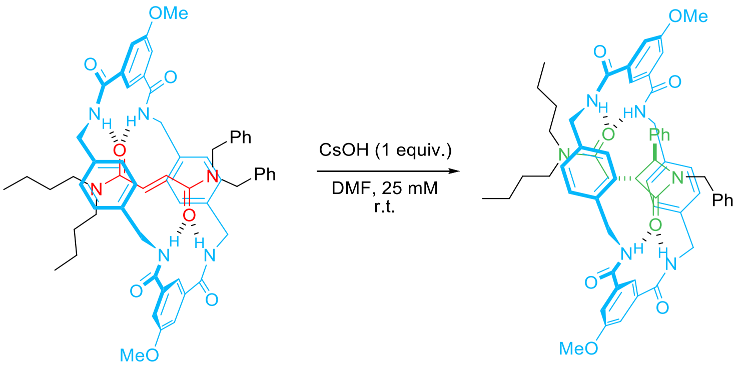

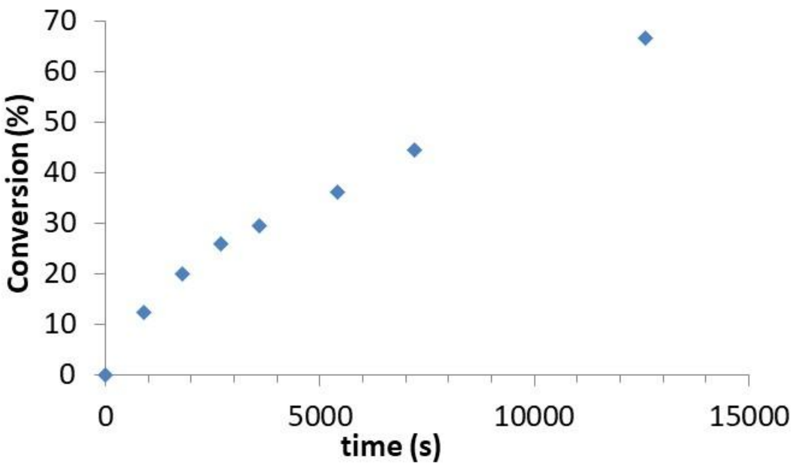

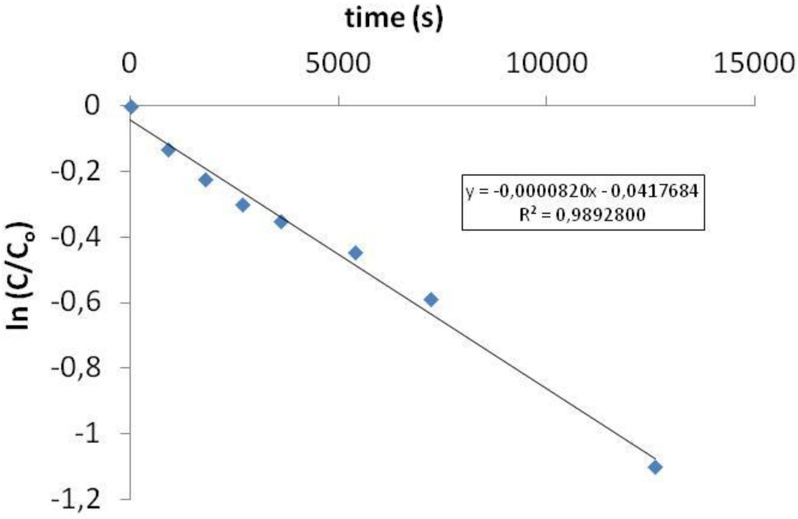

$t_{1/2}$ = 8453 ± 345 seconds

$k = (8.20 \pm 0.03) \times 10^{-5}\ s^{-1}$

ΔG‡ = 96.30 ± 0.10 kJ·mol^{-1}

Run 2

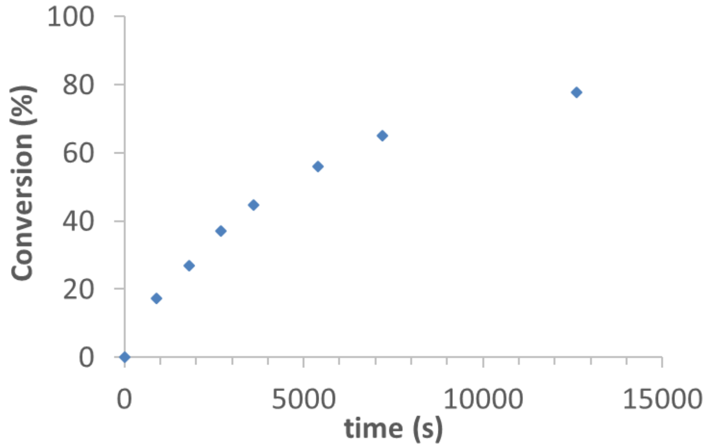

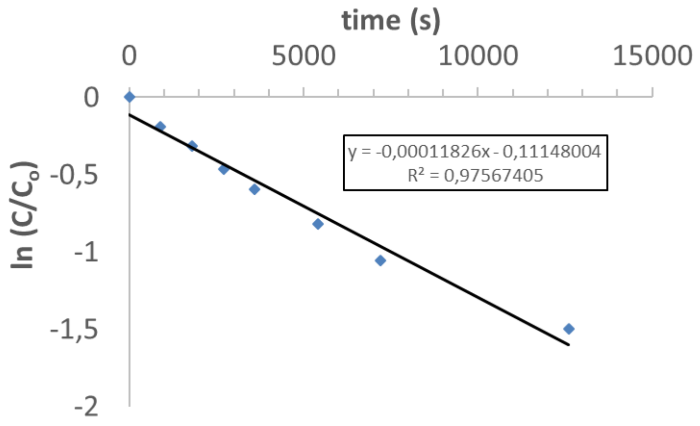

$t_{1/2}$ = 5861 ± 356 seconds

k = (1.18 ± 0.08) x 10^{-4} s^{-1}

ΔG‡ = 95.39 ± 0.15 kJ·mol^{-1}

Cyclization reaction of rotaxane 1f

Run 1

Variation of the cyclization percentage was measured over time using ^{1}H NMR spectroscopy (400 MHz, $CDCl_3$). From the corresponding data, reaction constant k, half-life time $t_{1/2}$ and free energy ΔG‡ were obtained.

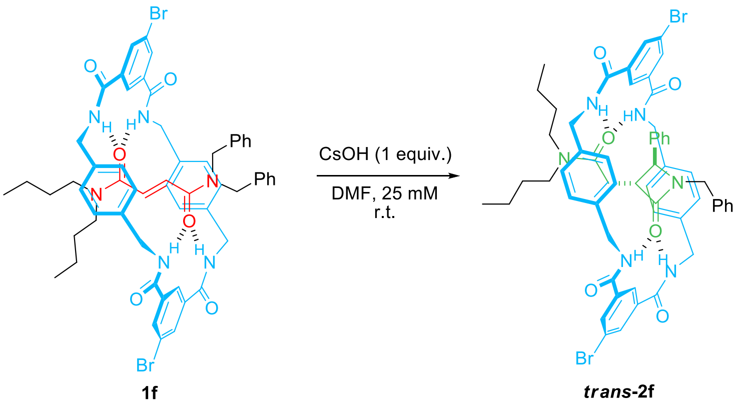

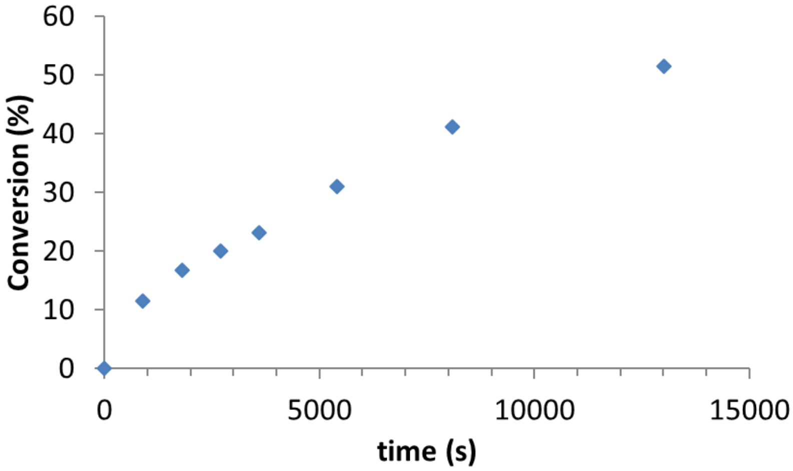

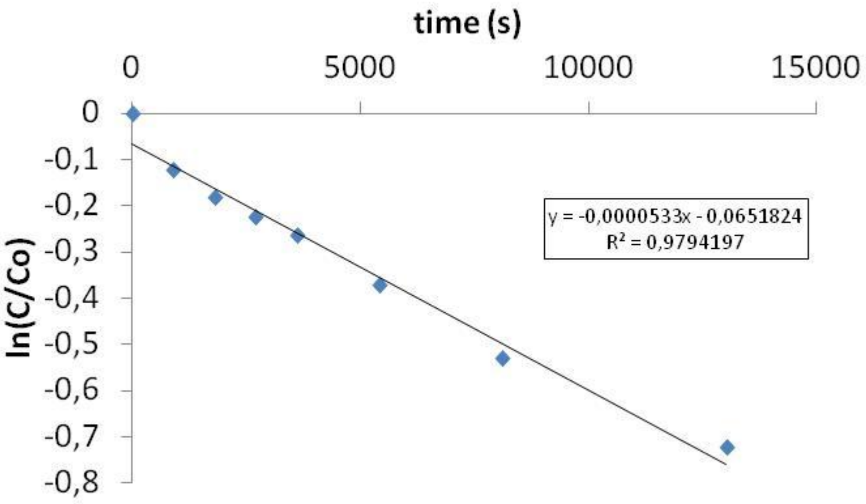

$t_{1/2}$ = 13003 ± 735 seconds

k = (5.33 ± 0.32) x 10^{-5} s^{-1}

ΔG‡ = 97.36 ± 0.14 kJ·mol^{-1}

Run 2

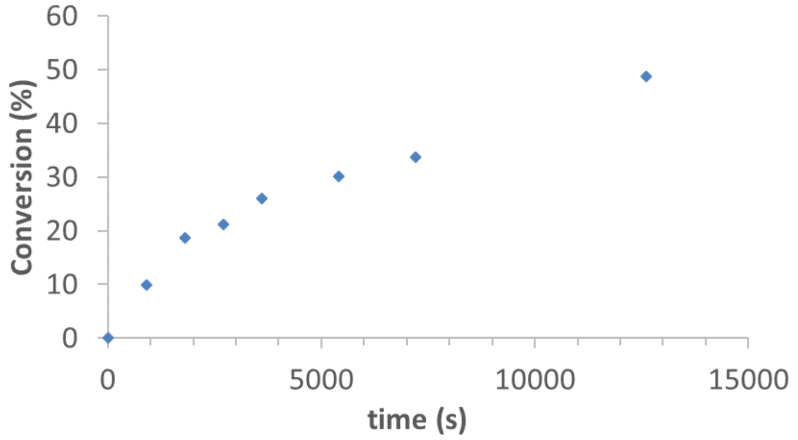

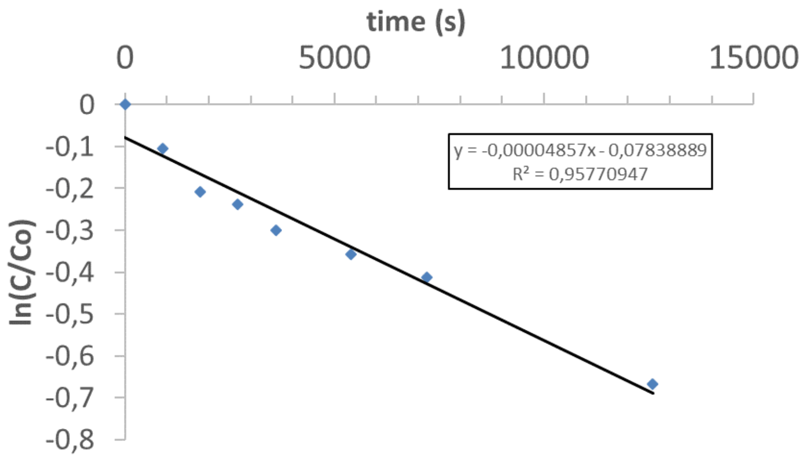

$t_{1/2}$ = 14271 ± 1144 seconds

k = (4.86 ± 0.42) x 10^{-5} s^{-1}

ΔG‡ = 97.60 ± 0.21 kJ·mol^{-1}

Cyclization reaction of rotaxane 1g

Run 1

Variation of the cyclization percentage was measured over time using ^{1}H NMR spectroscopy (400 MHz, $CDCl_3$). From the corresponding data, reaction constant *k*, half-life time $t_{1/2}$ and free energy ΔG‡ were obtained.

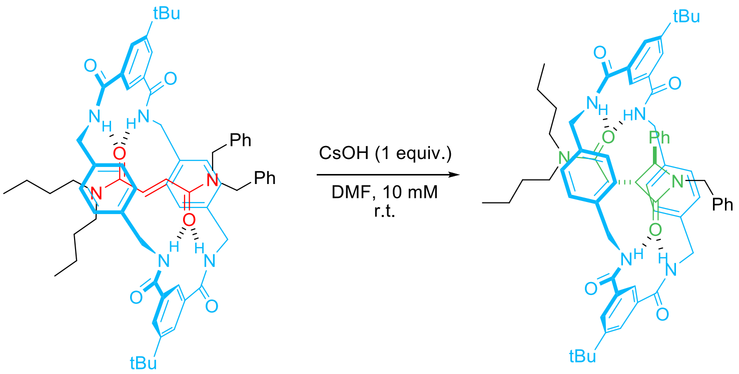

1g *trans*-**2g**

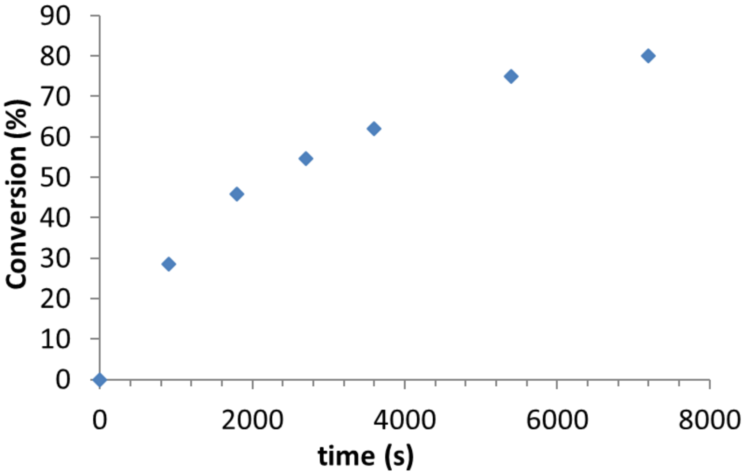

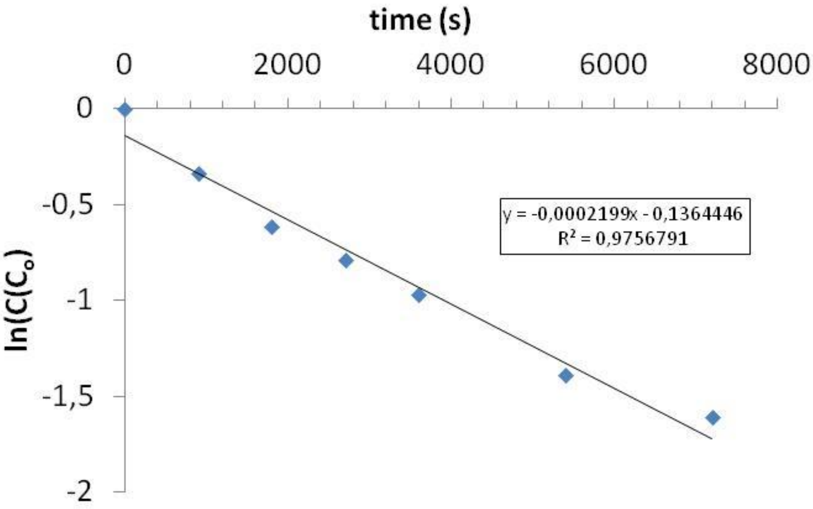

$t_{1/2}$ = 3152 ± 208 seconds

k = 2.20 ± 0.16 x 10^{-4} s^{-1}

ΔG‡ = 93.85 ± 0.16 kJ·mol^{-1}

Run 2

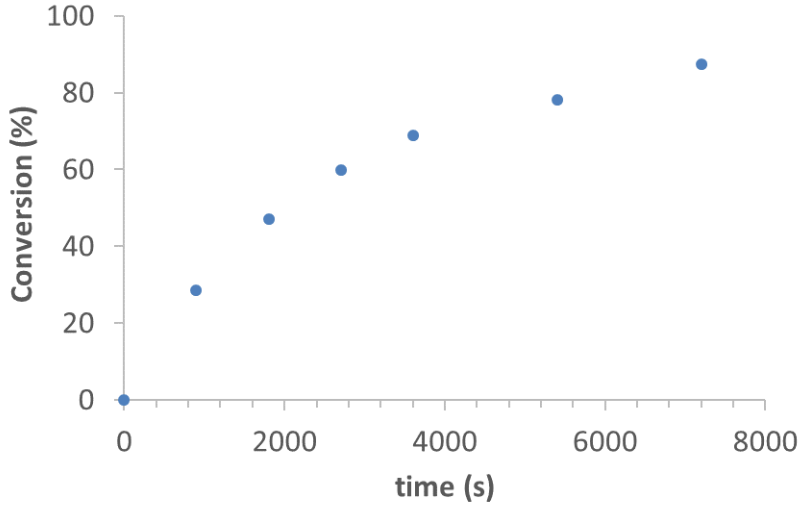

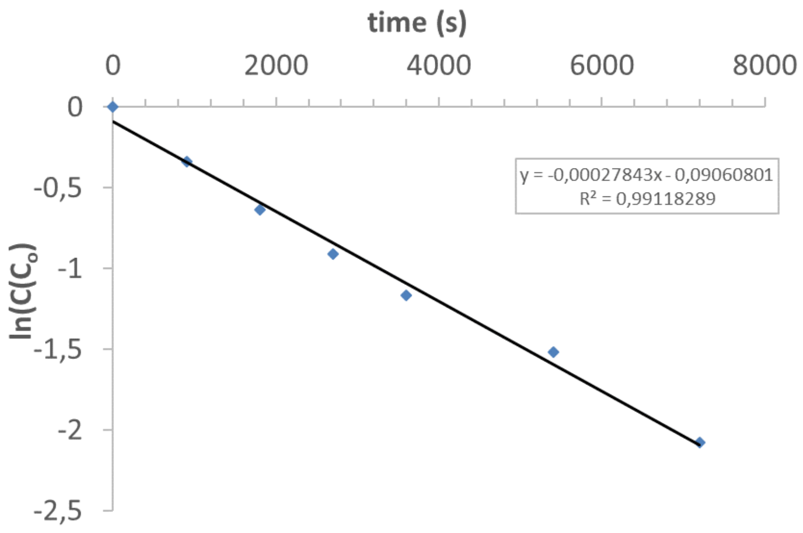

$t_{1/2}$ = 2489 ± 101 seconds

k = 2.78 ± 0.12 x 10^{-4} s^{-1}

ΔG‡ = 93.27 ± 0.10 kJ·mol^{-1}

Cyclization reaction of rotaxane 1h
Run 1

Variation of the cyclization percentage was measured over time using ^{1}H NMR spectroscopy (400 MHz, $CDCl_3$). From the corresponding data, reaction constant *k*, half-life time $t_{1/2}$ and free energy ΔG‡ were obtained.

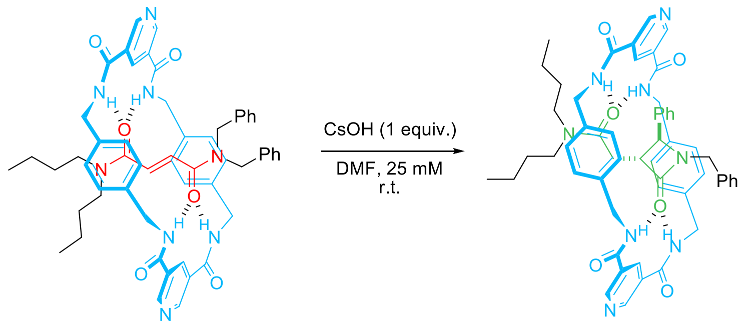

1h *trans*-**2h**

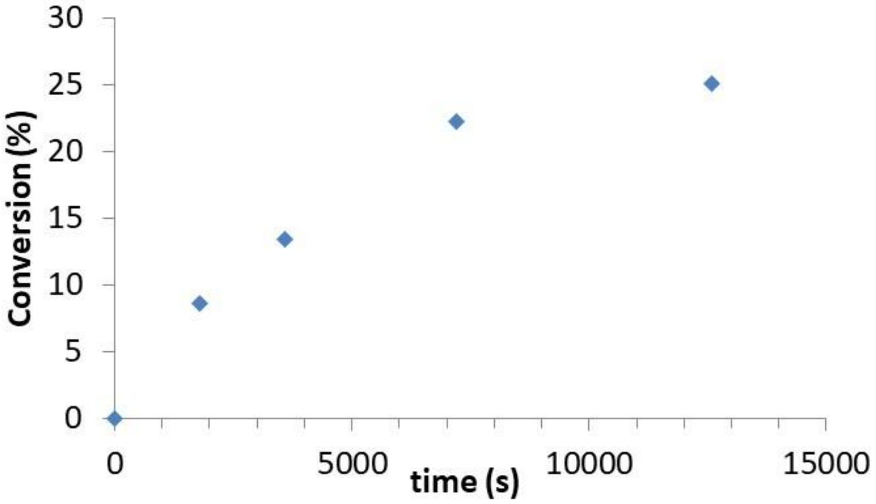

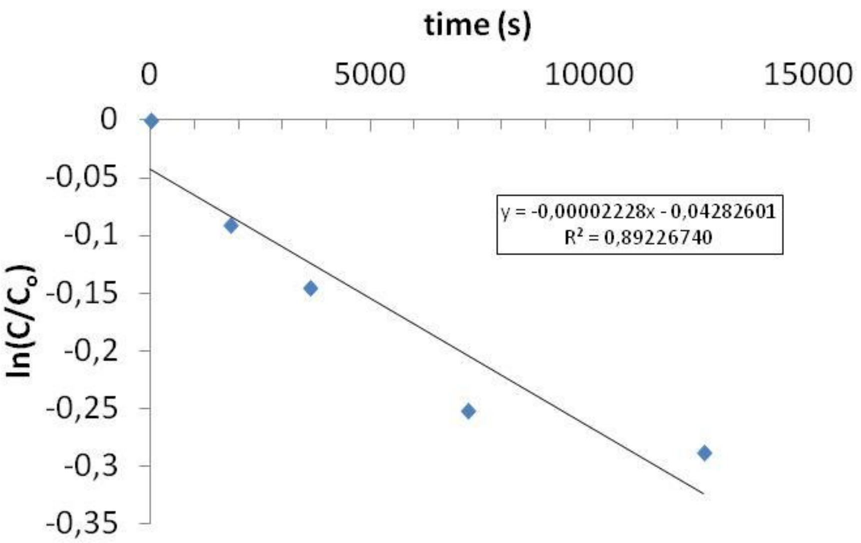

$t_{1/2} = 31110 \pm 5119$ seconds

$k = 2.22 \pm 0.45 \times 10^{-5}\ s^{-1}$

$\Delta G‡ = 99.52 \pm 0.45\ kJ\cdot mol^{-1}$

Run 2

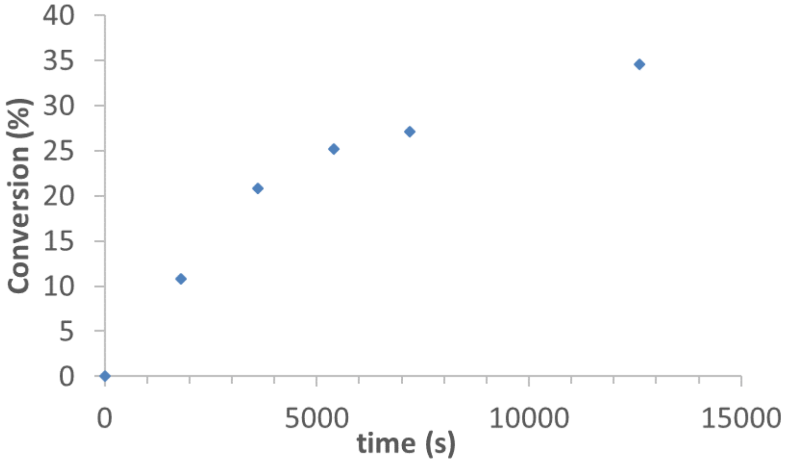

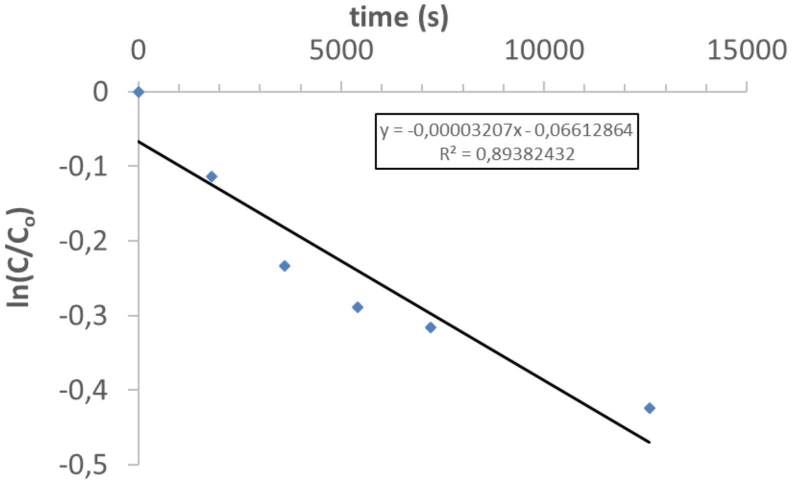

$t_{1/2}$ = 21613 ± 3117 seconds

k = 3.21 ± 0.55 x 10^{-5} s^{-1}

ΔG‡ = 98.62 ± 0.39 kJ·mol^{-1}

Cyclization reaction of rotaxane 1i

Run 1

Variation of the cyclization percentage was measured over time using ^{1}H NMR spectroscopy (400 MHz, $CDCl_3$). From the corresponding data, reaction constant *k*, half-life time $t_{1/2}$ and free energy ΔG‡ were obtained.

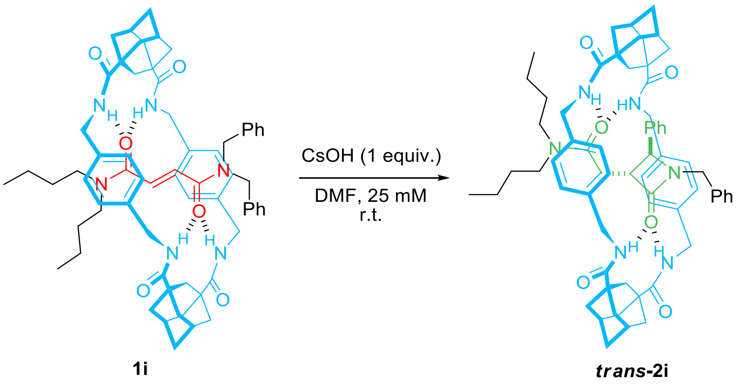

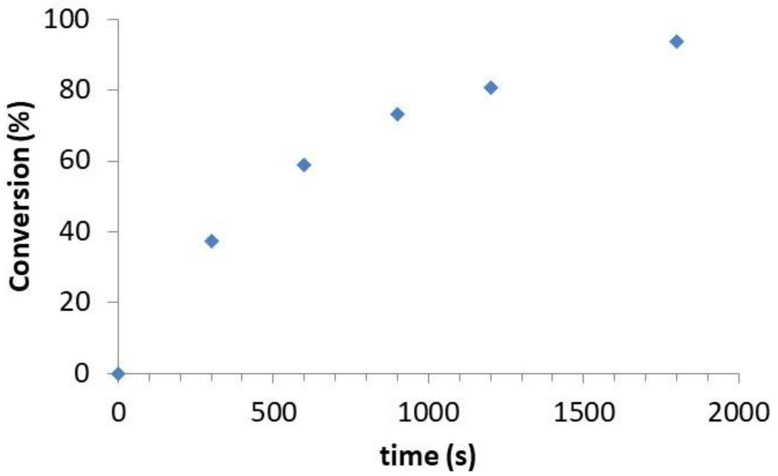

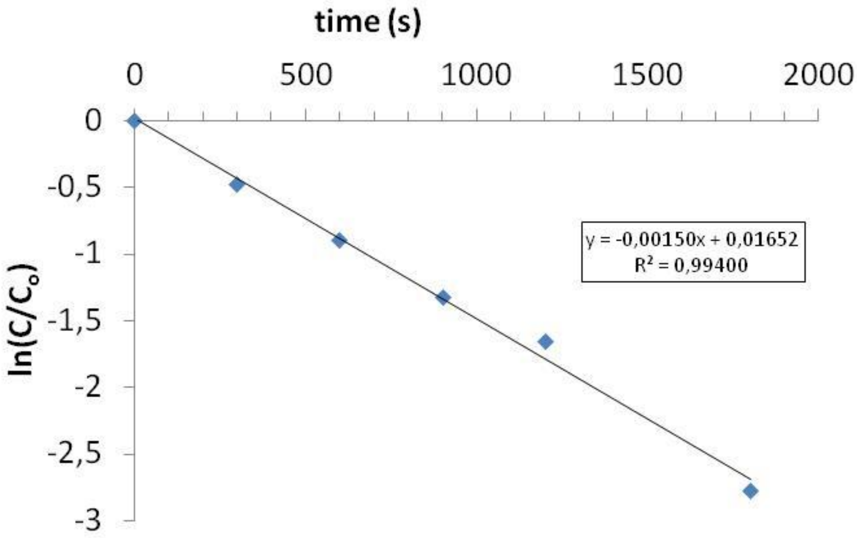

$t_{1/2}$ = 462 ± 18 seconds

$k = (1.50 \pm 0.06) \times 10^{-3}\ s^{-1}$

ΔG‡ = 89.10 ± 0.10 kJ·mol^{-1}

Run 2

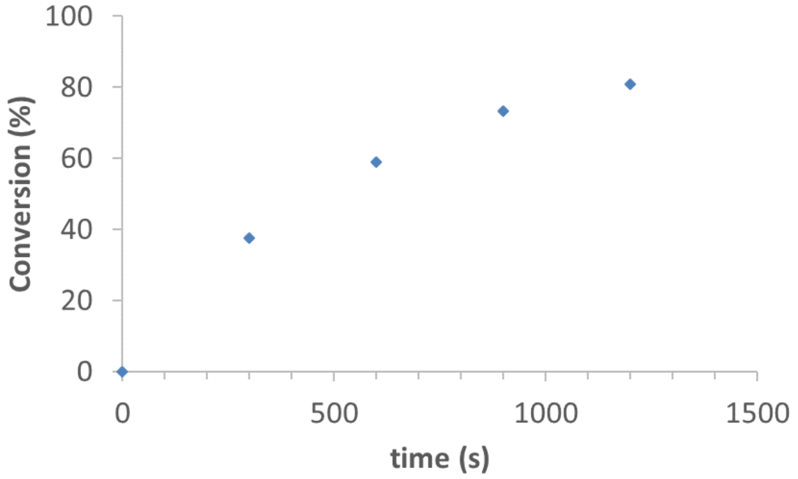

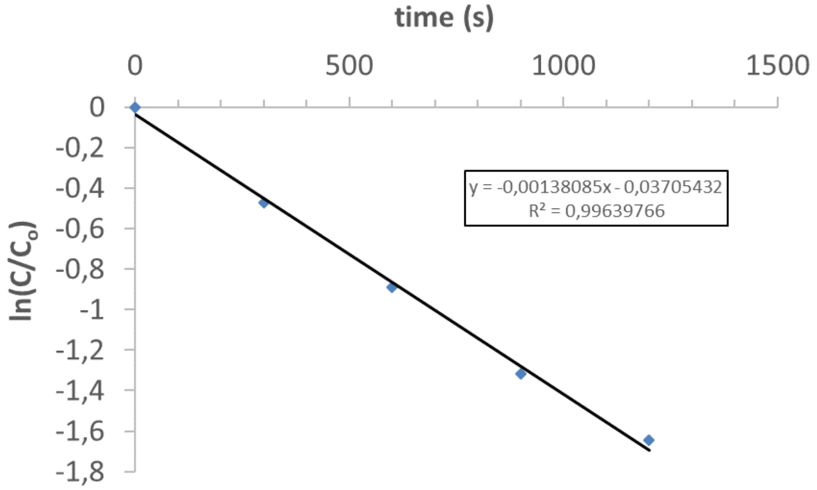

$t_{1/2}$ = 502 ± 17 seconds

k = (1.38 ± 0.05) x 10^{-3} s^{-1}

ΔG‡ = 89.30 ± 0.09 kJ·mol^{-1}

Cyclization reaction of rotaxane 1a-d_2

Run 1

Variation of the cyclization percentage was measured over time using ^{1}H NMR spectroscopy (400 MHz, $CDCl_3$). From the corresponding data, reaction constant k, half-life time $t_{1/2}$ and free energy ΔG‡ were obtained.

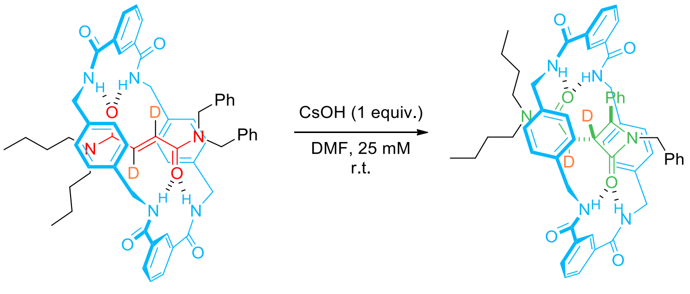

1a-d_2 **trans-2a-d_2**

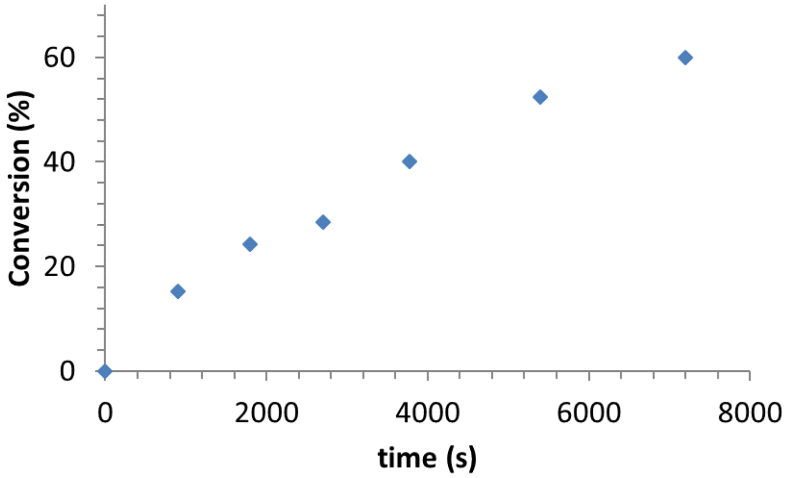

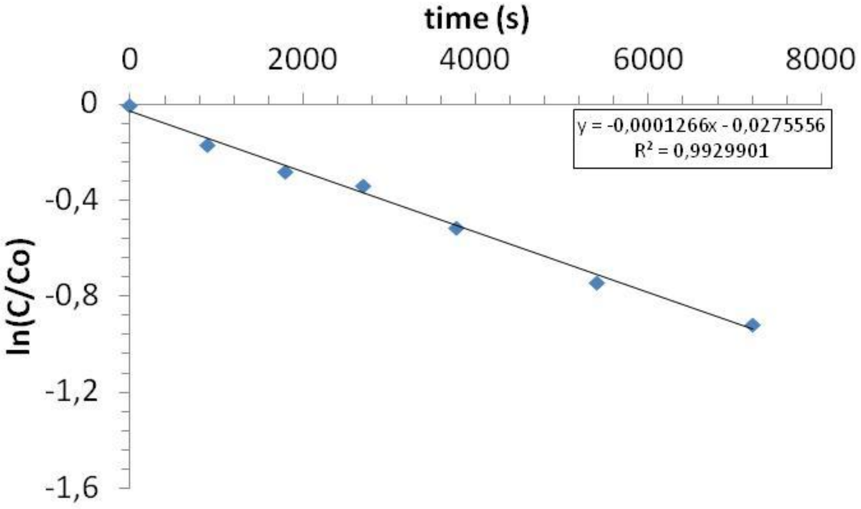

$t_{1/2}$ = 5475 ± 198 seconds

k = (1.27 ± 0.05) x 10^{-4} s^{-1}

ΔG‡ = 95.21 ± 0.08 kJ·mol^{-1}

Run 2

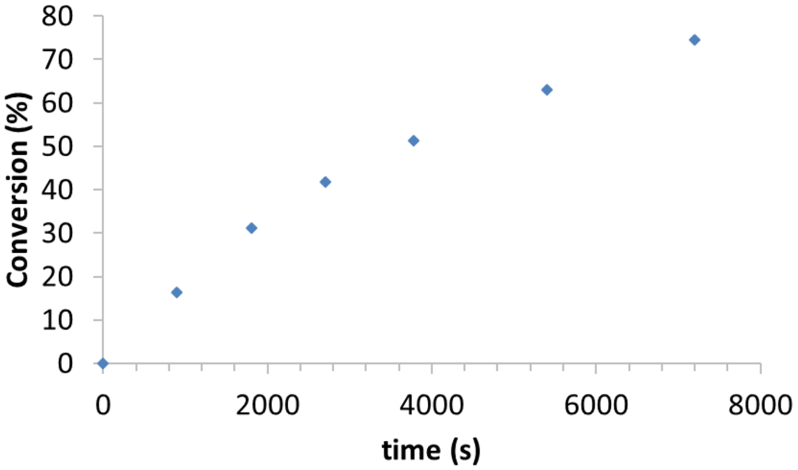

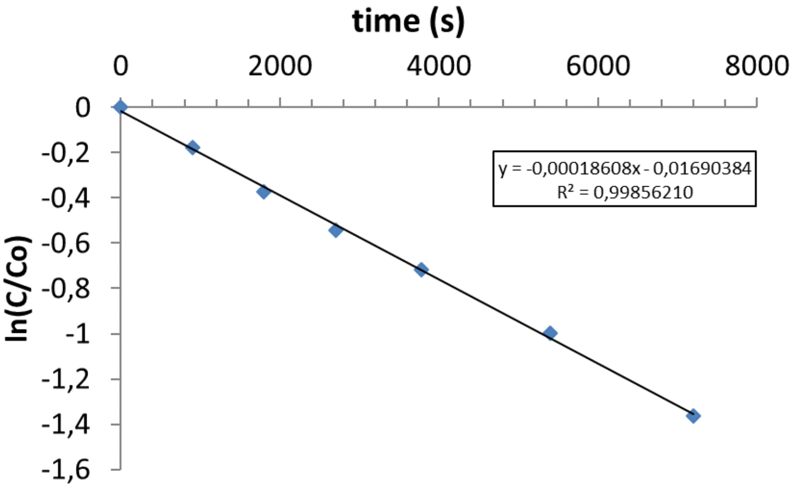

$t_{1/2}$ = 3724 ± 62 seconds

k = (1.86 ± 0.03) x 10^{-4} s^{-1}

ΔG‡ = 94.27 ± 0.04 kJ·mol^{-1}

Cyclization reaction of rotaxane 4

Variation of the cyclization percentage was measured over time using ^{1}H NMR spectroscopy (400 MHz, $CDCl_3$). From the corresponding data, reaction constant k, half-life time $t_{1/2}$ and free energy ΔG‡ were obtained.

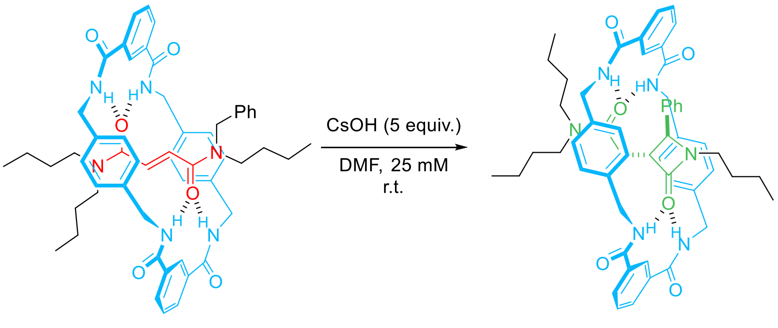

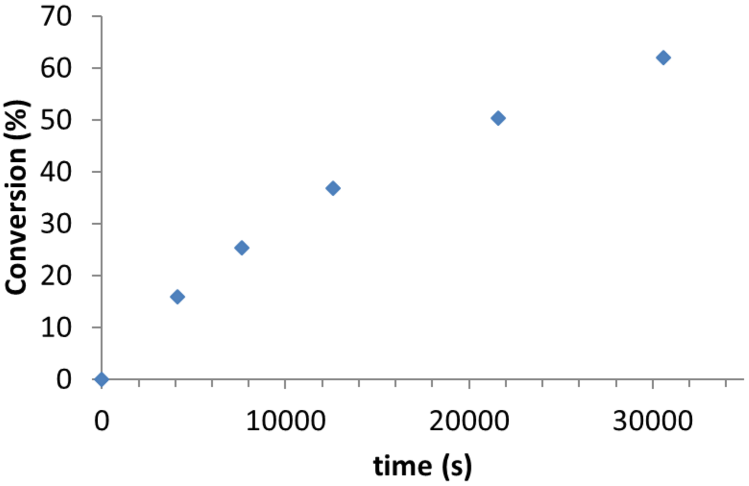

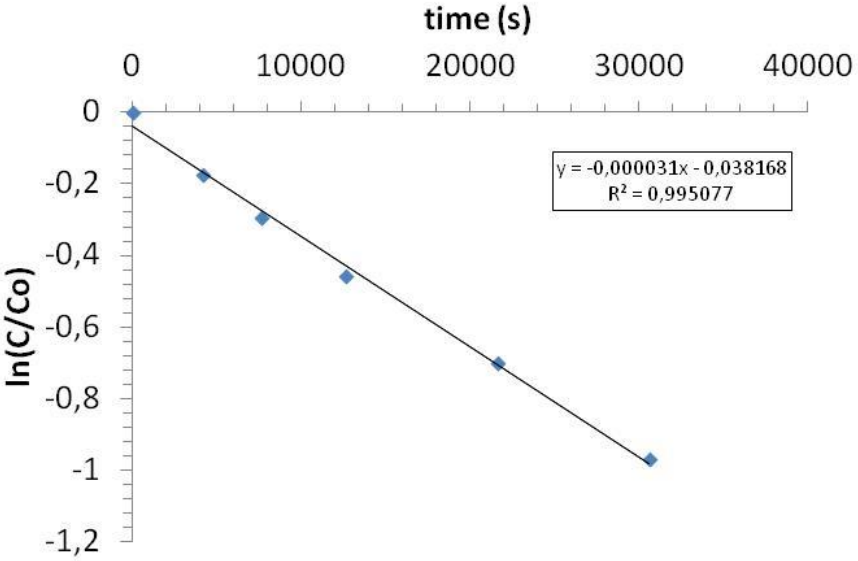

$t_{1/2}$ = 22359 ± 639 seconds

$k = (3.08 \pm 0.11) \times 10^{-5}\ s^{-1}$

ΔG‡ = 98.71 ± 0.07 kJ·mol^{-1}

Cyclization reaction of rotaxane 4-*d*$_2$

Variation of the cyclization percentage was measured over time using ^{1}H NMR spectroscopy (400 MHz, $CDCl_3$). From the corresponding data, reaction constant *k*, half-life time $t_{1/2}$ and free energy ΔG‡ were obtained.

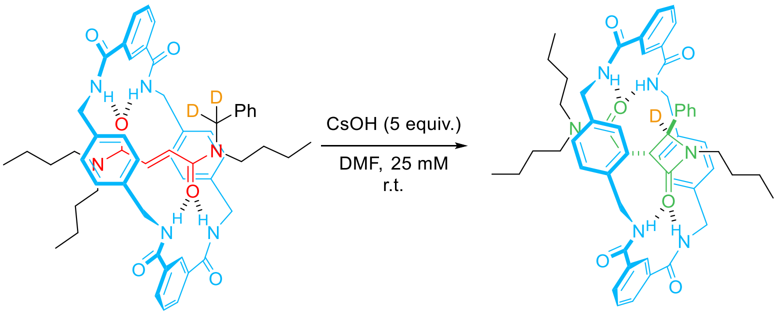

4-*d*$_2$ *trans*-5-*d*$_1$

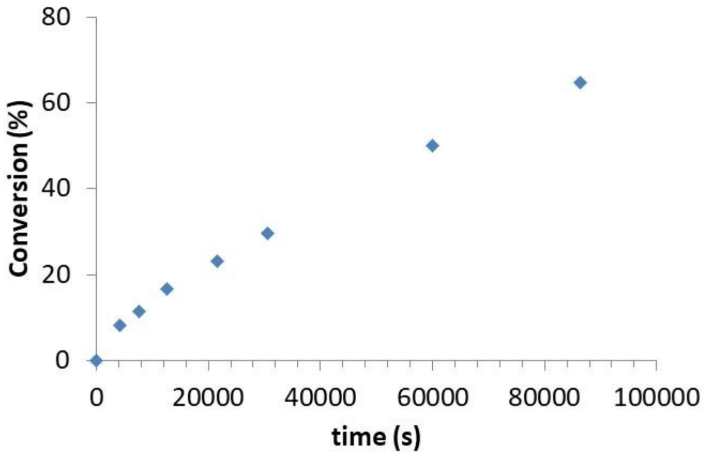

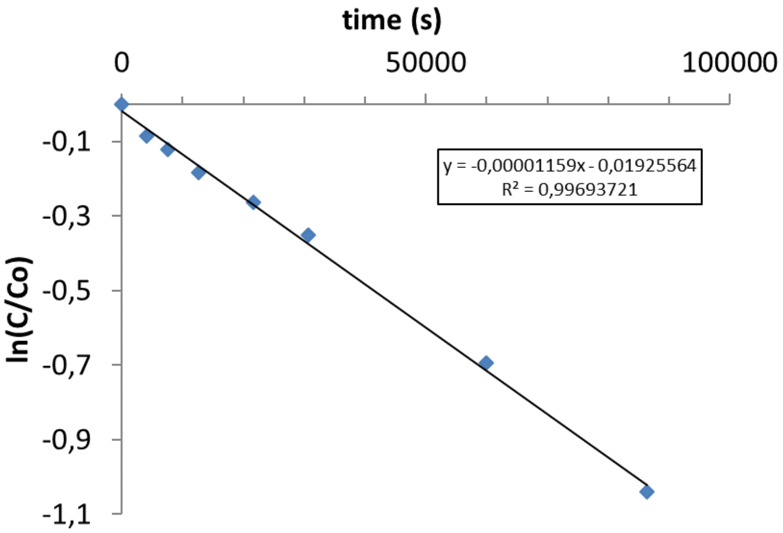

$t_{1/2}$ = 59828 ± 1324 seconds

$k = (1.16 \pm 0.03) \times 10^{-5}\ s^{-1}$

ΔG‡ = 101.15 ± 0.06 kJ·mol^{-1}

11. Hammet plot values

Table S1. σ values and reaction constants of rotaxanes **1a-c** and **1d-i.**

Rotaxane	σ value[a]	*k* (s^{-1}) [b]
**1a**	0	0.0001285
**1b**	+ 0.227	0.0013934
**1c**	– 0.268	0.0000169
**1d**	+ 0.710	-
**1e**	+ 0.115	0.0001001
**1f**	+ 0.391	0.0000509
**1g**	– 0.100	0.0002492
**1h**	+ 0.620 [c]	0.0000271
**1i**	-	0.0014403

[a] σ values obtained from the compilation by D. H. McDaniel and H. C. Brown, *J. Org. Chem.* **1958**, *23*, 420; [b] media obtained from two different experiments; [c] value obtained from: D. J. Brown, "*The Pyrimidines*" Chapter 13. Interscience, New York, **1962.**

12. Determination of the deuterium distribution in interlocked lactams *trans*-2a-d_2 and *trans*-4-d_1

***trans*-2a-d_2**

Most of the resonances of the hydrogen atoms of the lactam core in *trans*-**2a**-d_2 appeared in the aliphatic region, being impossible to quantify the percentage of deuterium retained after the base-promoted cyclization of the rotaxane **1a**-d_2 (93% of D). Thus, we performed a dethreading protocol to obtain the non-interlocked lactam *trans*-**3**-d_2, which was analyzed by ^{1}H NMR spectroscopy and compared to the non-labelled lactam *trans*-**3** (reported in reference 1). Having in mind the initial deuterium abundance in rotaxane **1a**-d_2 (93% of D), the level of retention of D_1 is 97 %. In the case of deuterium D_2 (20%), its low abundance is due to a progressive hydrogen-deuterium exchange occurring under the basic reaction conditions.

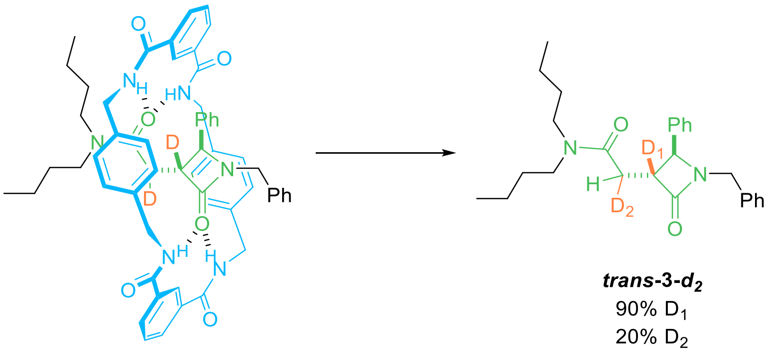

***trans*-2a-d_2**

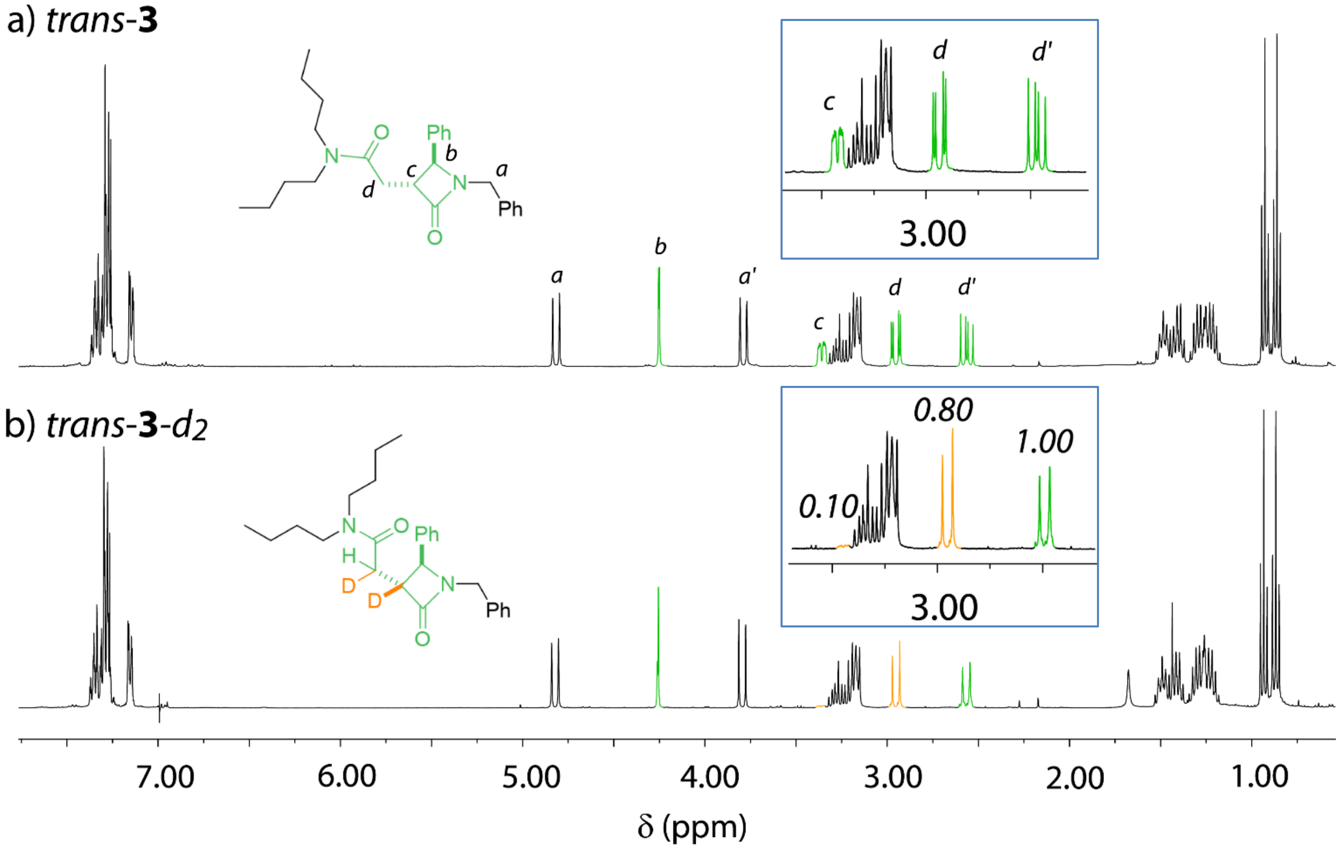

Figure S3. ^{1}H NMR spectra (400 MHz, $CDCl_3$, 298 K) of: a) *trans*-**3**; b) *trans*-**3**-d_2. Insets: selected region of the lactam core with the signals of labelled atoms.

***trans*-5-*d*₁**

In order to determine the percentage of deuterium in compound *trans*-**4**-d_1 retained after the base-catalyzed cyclization of the rotaxane **4**-d_2 (99% of D), we analyzed the ^{1}H NMR spectrum and compared with the non-labelled lactam *trans*-**5** (reported in reference 1).

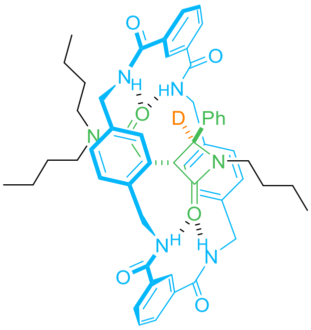

***trans*-5-*d*₁**
80% D

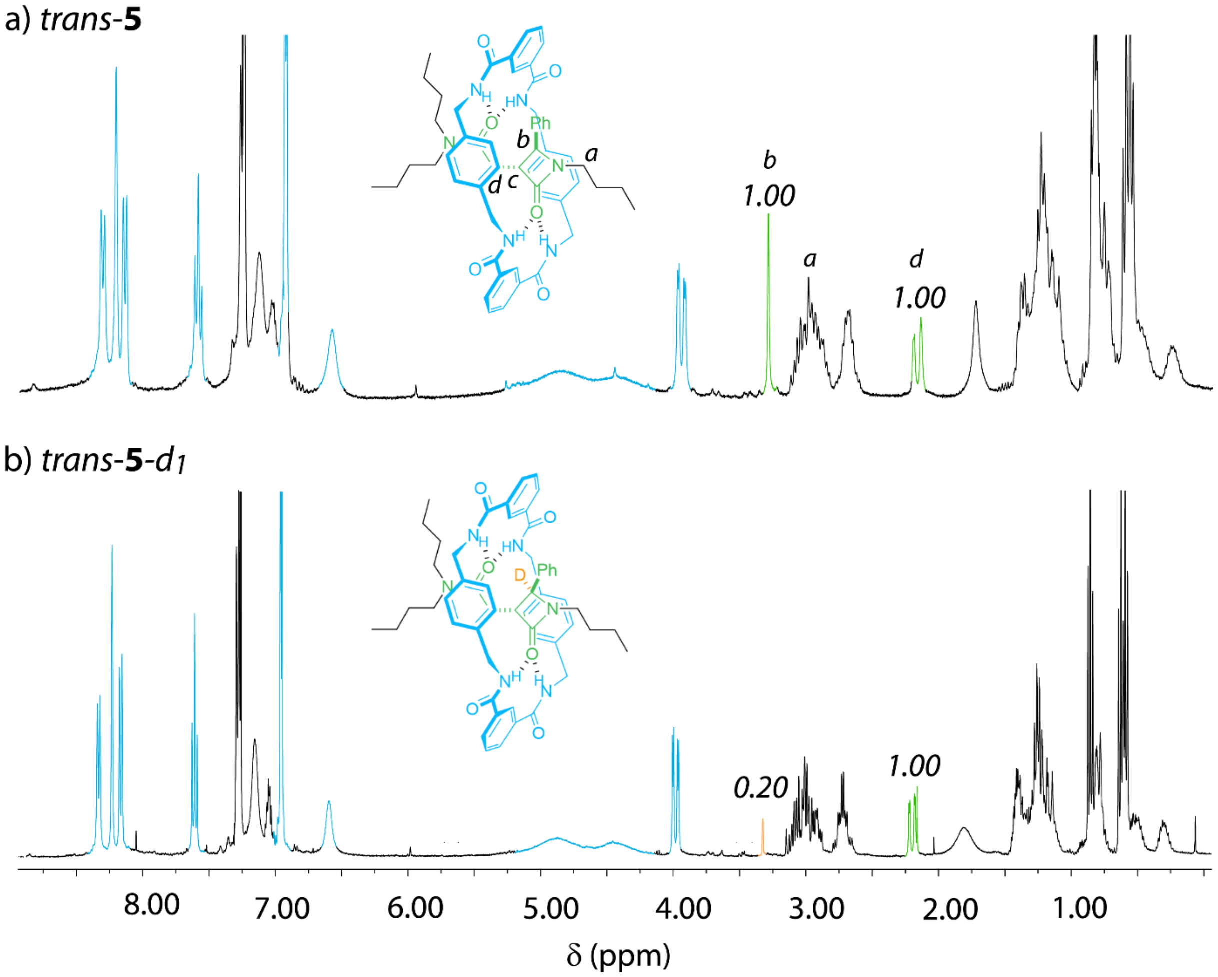

Figure S4. ^{1}H NMR spectra (400 MHz, $CDCl_3$, 298 K) of: a) *trans*-**5**; b) *trans*-**5**-d_1.

13. Crystal data and structure refinement for rotaxane *trans*-8i

Table S2. Crystal data and structure refinement for *trans*-**8i.**

	trans-**8i**
Empirical formula	$C_{65} H_{80} Cl N_6 O_6$
Formula weight	1076.80
T [K]	100(2)
Wavelength [Å]	1.54178
Crystal system	Triclinic
Space group	P-1
a (Å)	12.0771(6)
b (Å)	13.4199(6)
c (Å)	18.1881(9)
α (°)	75.562(2)
β (°)	80.992(2)
γ (°)	87.013(2)
V [Å^3]	2819.2(2)
Z	2
ρ [g·cm^{-3}]	1.268
μ [mm^{-1}]	1.064
F_{000}	1154
Crystal size [mm^3]	0.190 x 0.140 x 0.060
θ range (º)	2.537 -74.720
h	-15 to 15
k	-16 to 16
l	-22 to 22
Reflections collected	123263
Independent reflections	11510
R(int)	0.0373
Refinement method	
Parameters	759
Restraints	6
Goodness-of-fit on F^2	1.032
$R1$ [I > 2σ(I)]	0.0532
$wR2$ [I > 2σ(I)]	0.1369
$R1$ (all data)	0.0575
$wR2$ (all data)	0.1408
$\Delta\rho$ [e·Å^{-3}]	0.949 /-0.441

a) b)

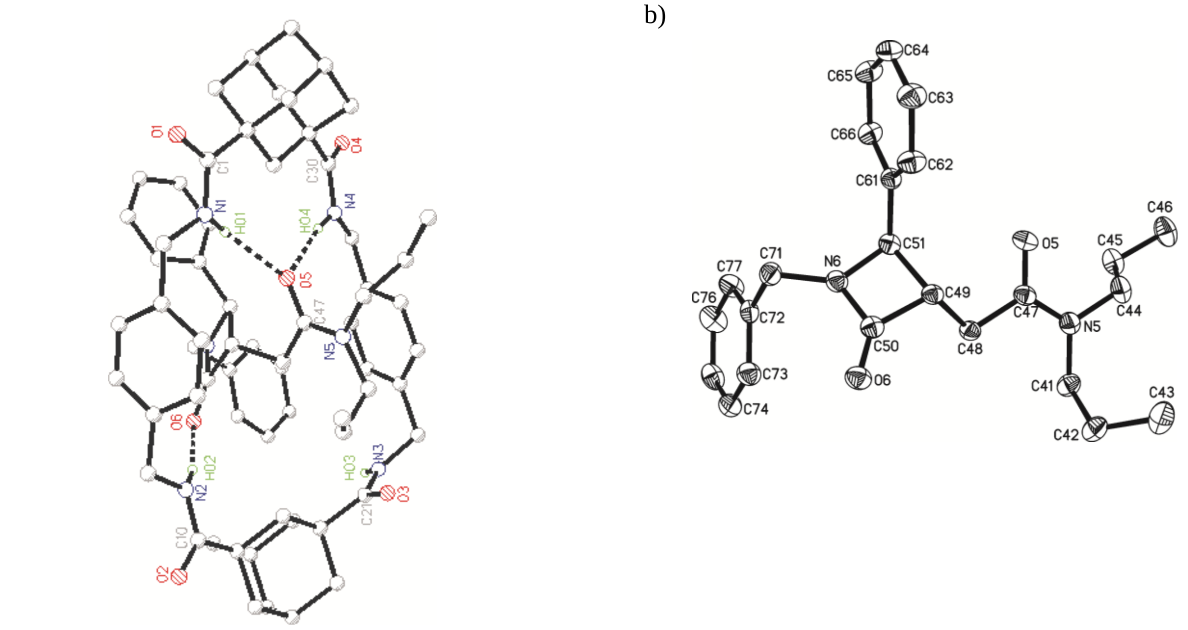

Figure S5. a) Molecular structure of β-lactam *trans*-**8i** with thermal ellipsoids drawn at 50% probability. One molecule of solvent was removed for clarity; b) Thread of β-lactam *trans*-**8i** (the macrocycle was removed for clarity) with thermal ellipsoids drawn at 50% probability.

X-ray Structure Determinations.

Intensities were registered at low temperature on a Bruker D8QUEST diffractometer using monochromated Cu *K*α radiation (λ = 1.54178 Å). Absorption corrections were based on multi-scans (program SADABS). Structures were refined anisotropically using SHELXL-2018.[4] Hydrogen atoms were included using rigid methyl groups or a riding model. The NH hydrogens were located in a difference synthesis and refined freely with SADI: The structure contains one 1,2 dichloroethane molecule disordered over an inversion center.

Table S3. Hydrogen bonds for rotaxane *trans*-**8i** [Å and (°)].

**D-H...A**	**d(D-H)**	**d(H...A)**	**d(D...A)**	**<(DHA)**
N(1)-H(01)...O(5)	0.856 (16)	2.188(17)	3.0201(18)	164(2)
N(2)-H(02)...O(6)	0.852(16)	2.134(16)	2.9744(19)	168.8(19)
N(3)-H(03)...O(1)#2	0.838(16)	2.240(18)	2.924(2)	139.0(18)
N(4)-H(04)...O(5)	0.845(16)	2.336(17)	3.1687(19)	168(2)
C(29)-H(29B)...O(2)#3	0.99	2.39	3.351(2)	164.1

Symmetry transformations used to generate equivalent atoms:

#1 -x+1,-y+2,-z+1 #2 x,y+1,z #3 x-1,y,z

14. CsOH effect on the stability of rotaxane 1k

The stability of rotaxane **1k**[3] towards the dethreading process was studied, in the presence or absence of CsOH as a base. The presence of base slowed down the deslipping into the two free components. The deprotonation of one of the amide NH groups of the macrocycle, forming an amidate, seems to stabilize the mechanical bond.

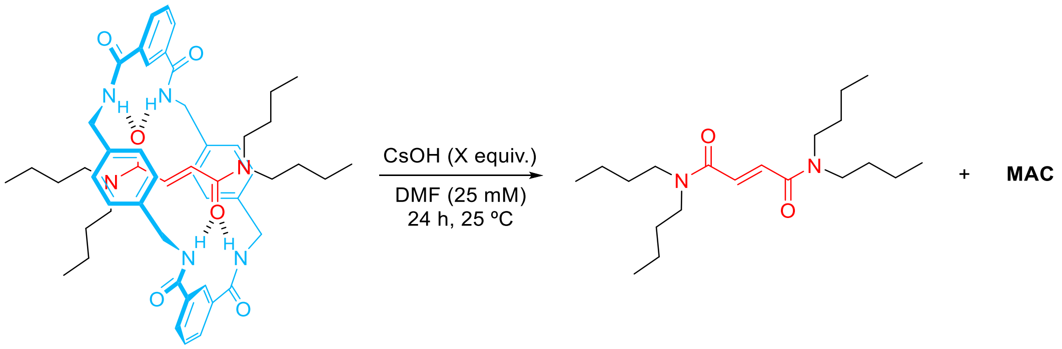

1k

Table S4. Dethreading process of rotaxane 1k in DMF in the absence or presence of base.[a]

entry	CsOH (x equiv)	Conversion (%)[b]
1	0	15
2	1	5
3	3	2

[a] *Reactions conditions*: rotaxane **1k** (20 mg, 0.023 mmol), CsOH (x equiv.), DMF (1 mL); [b] Determined by ^{1}H NMR from the reaction crude.

15. ^{1}H and ^{13}C NMR Spectra of synthesized compounds

T1a-*d*$_2$ (^{1}H NMR, 300 MHz, $CDCl_3$, 298K)

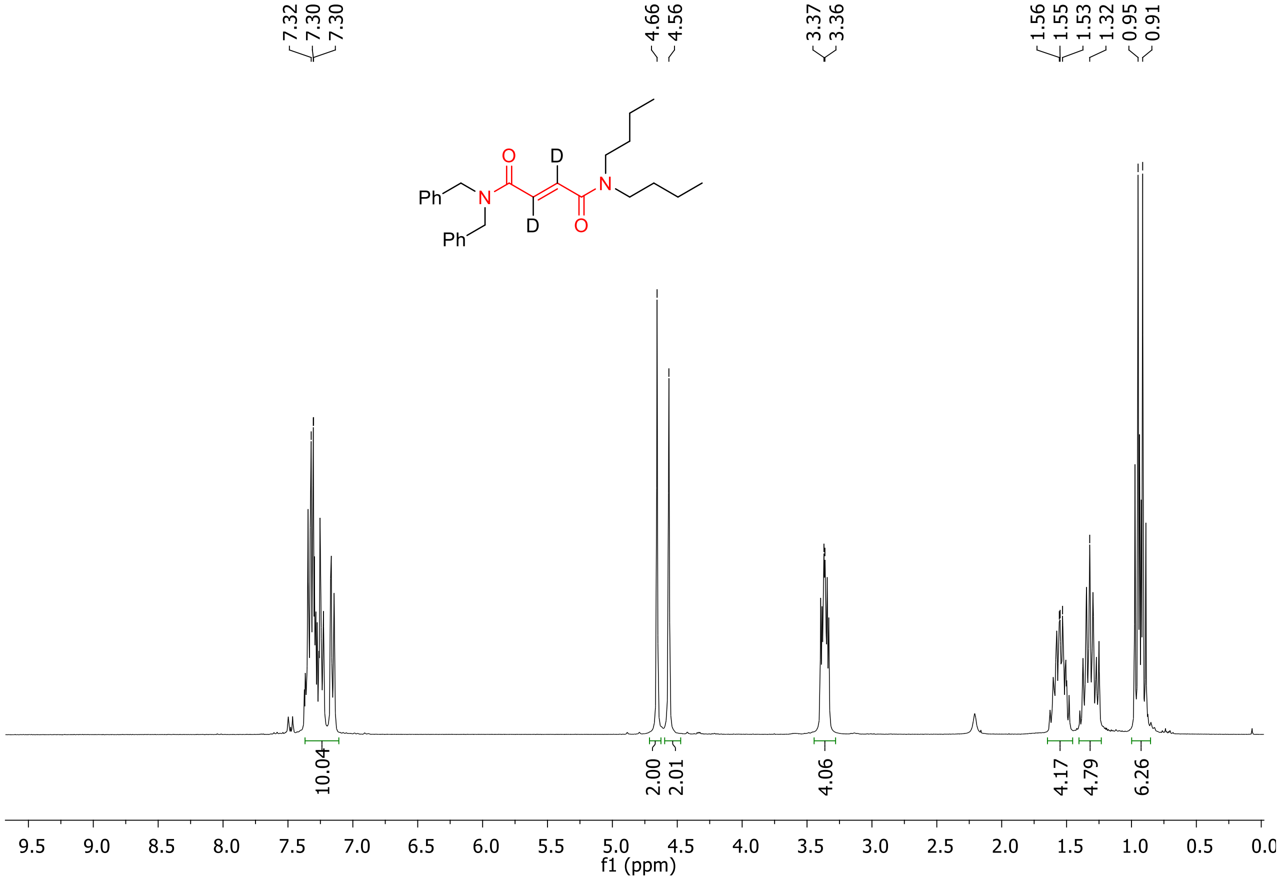

T1a-*d*$_2$ (^{13}C NMR, 75 MHz, $CDCl_3$, 298K)

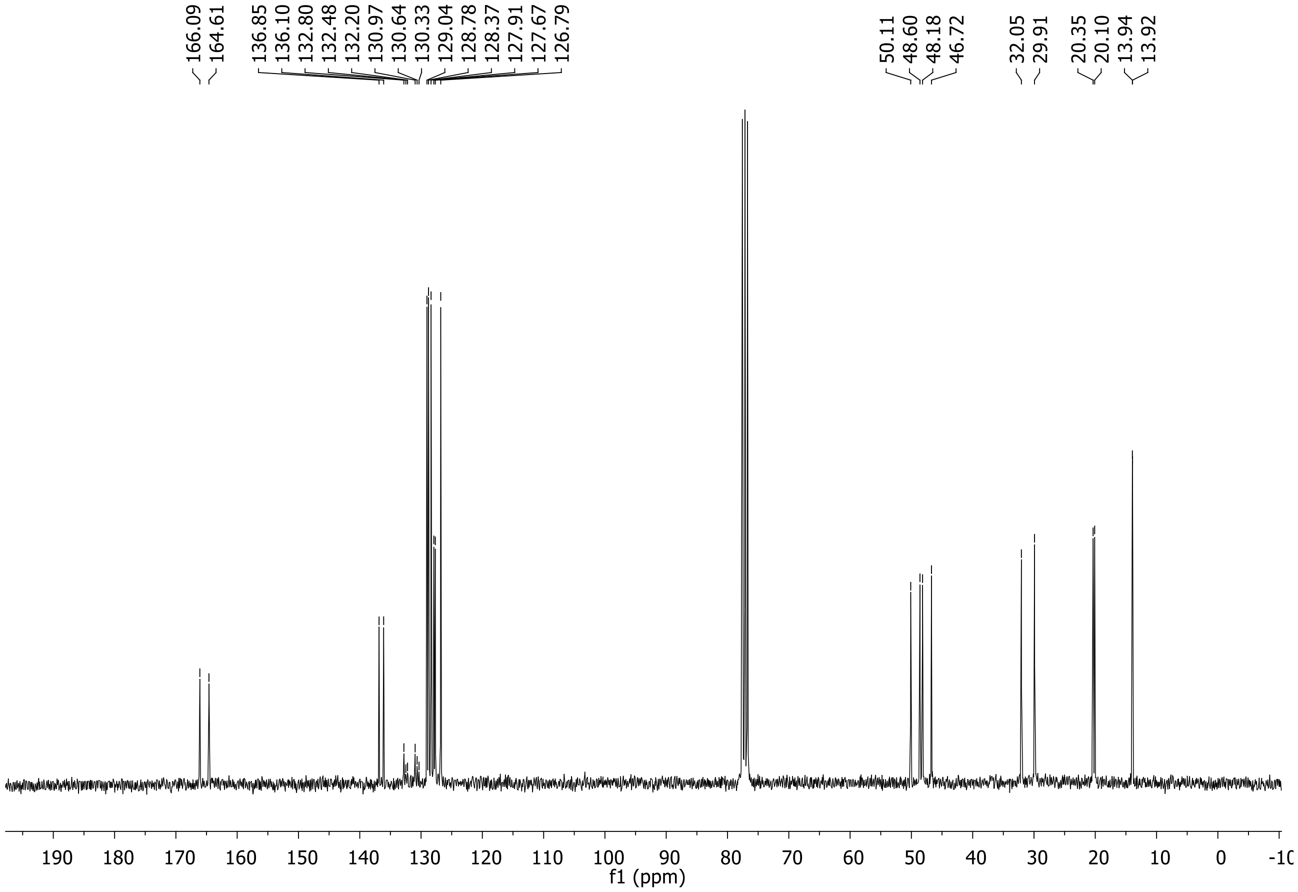

T1b-*d₂* (^{1}H NMR, 300 MHz, $CDCl_3$, 298K)

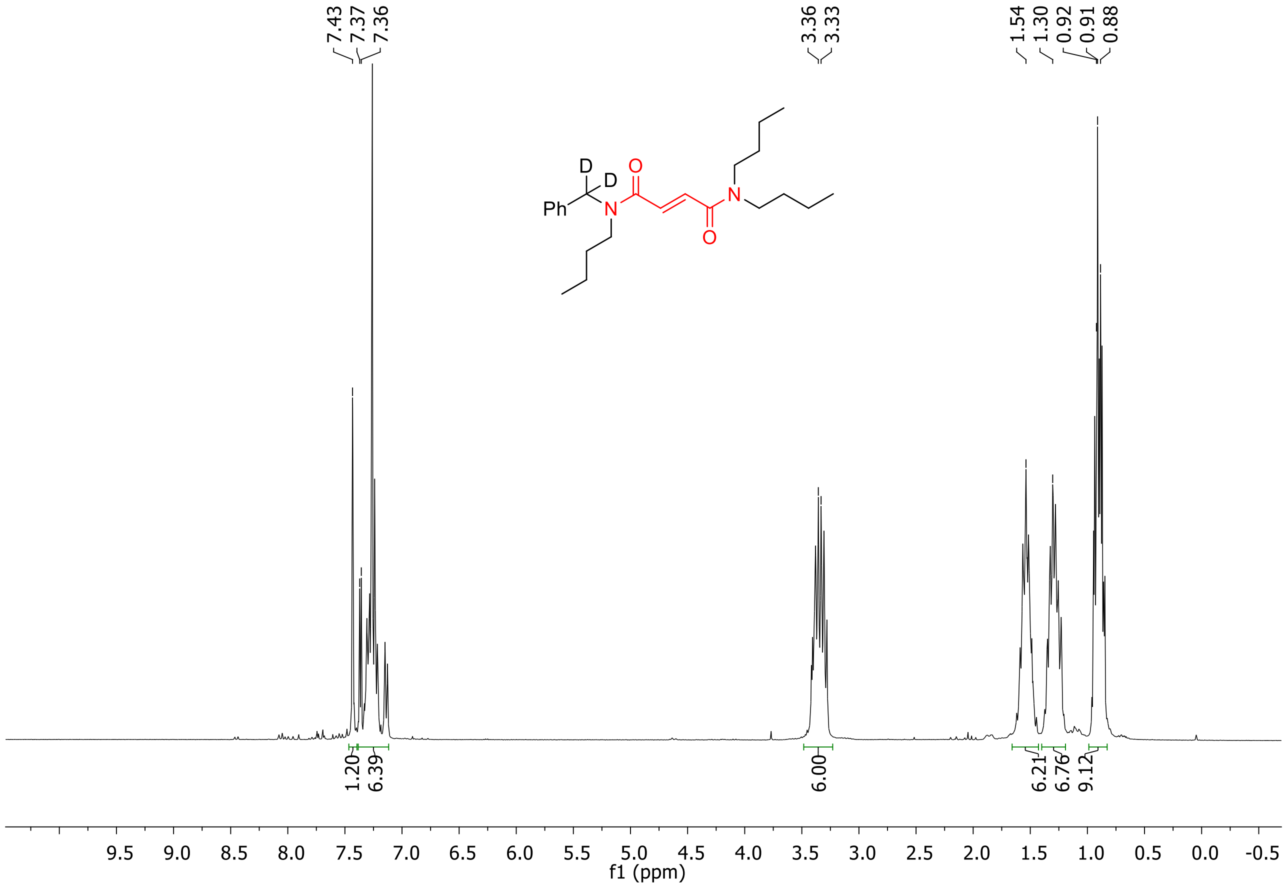

T1b-*d₂* (^{13}C NMR, 75 MHz, $CDCl_3$, 298K)

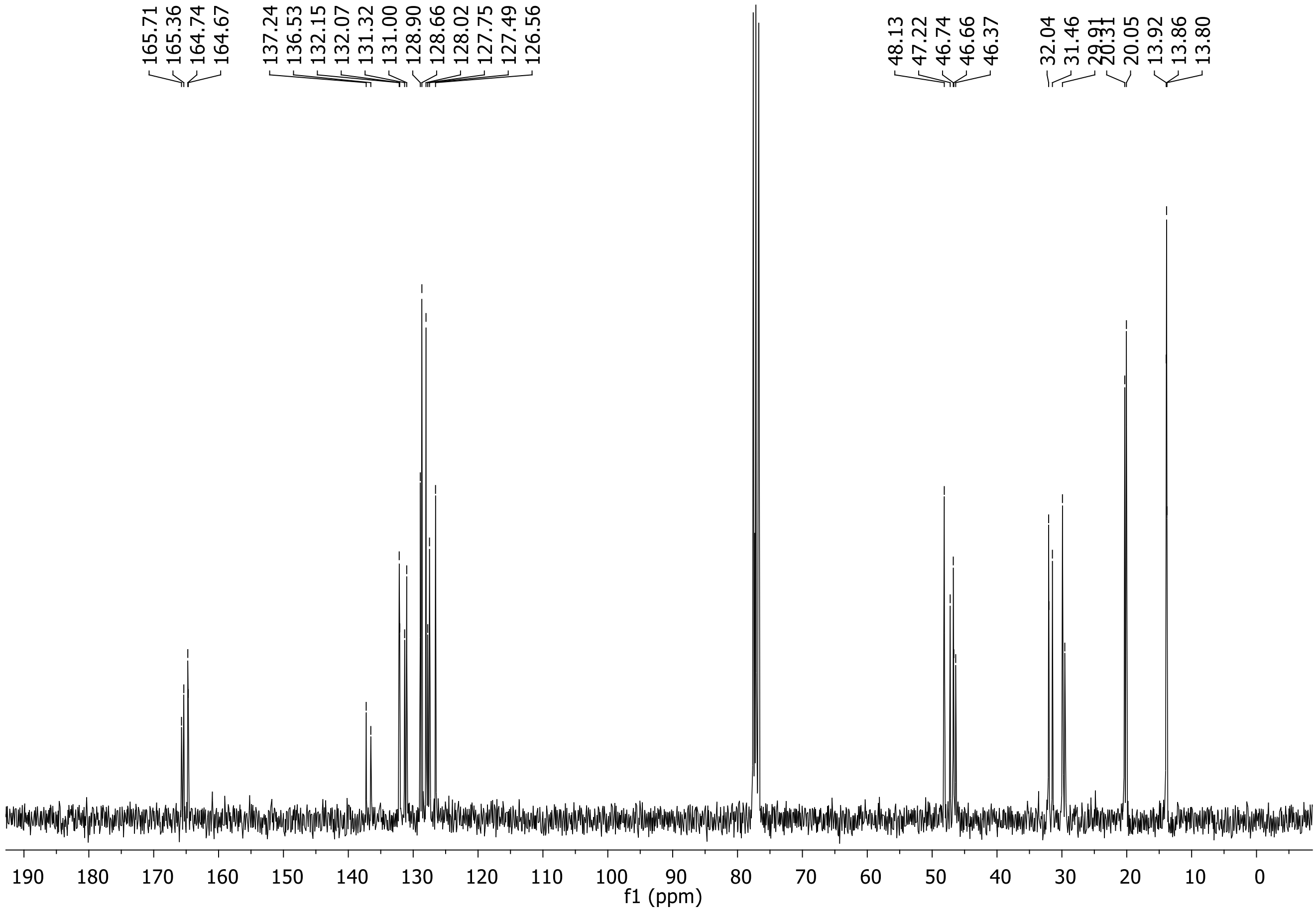

1d (^{1}H NMR, 600 MHz, $CDCl_3$, 298K)

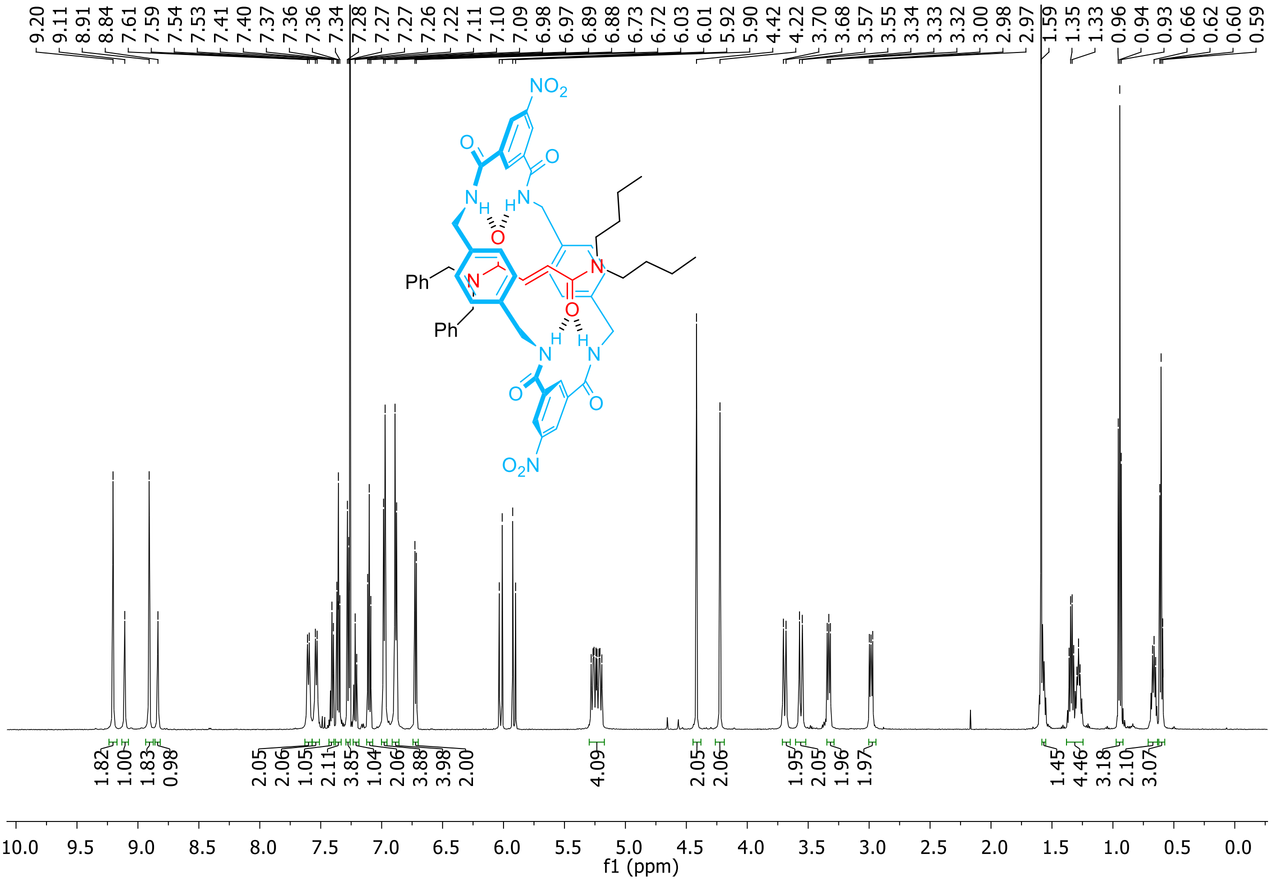

1d (^{13}C NMR, 151 MHz, $CDCl_3$, 298K)

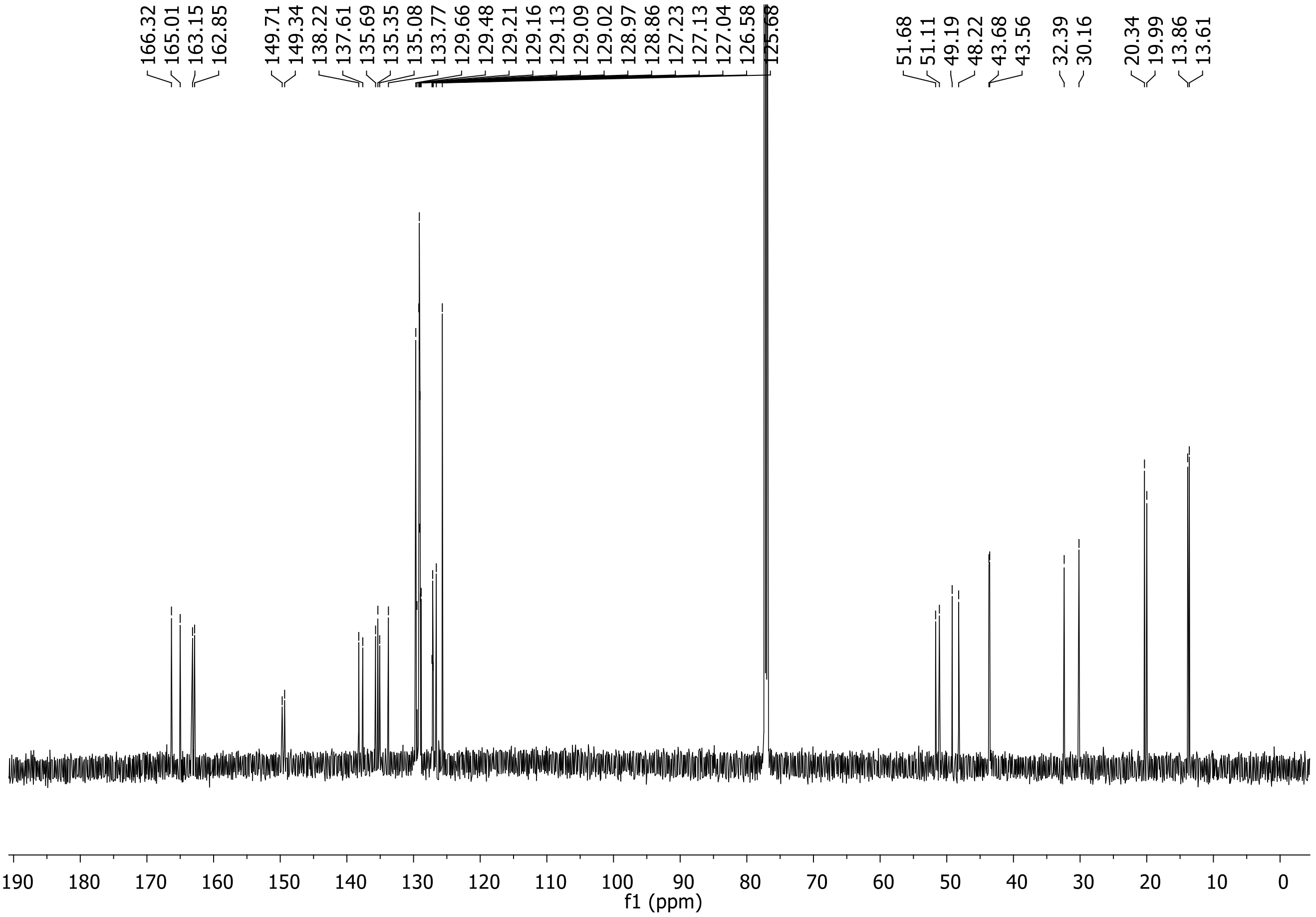

1e (^{1}H NMR, 600 MHz, $CDCl_3$, 298K)

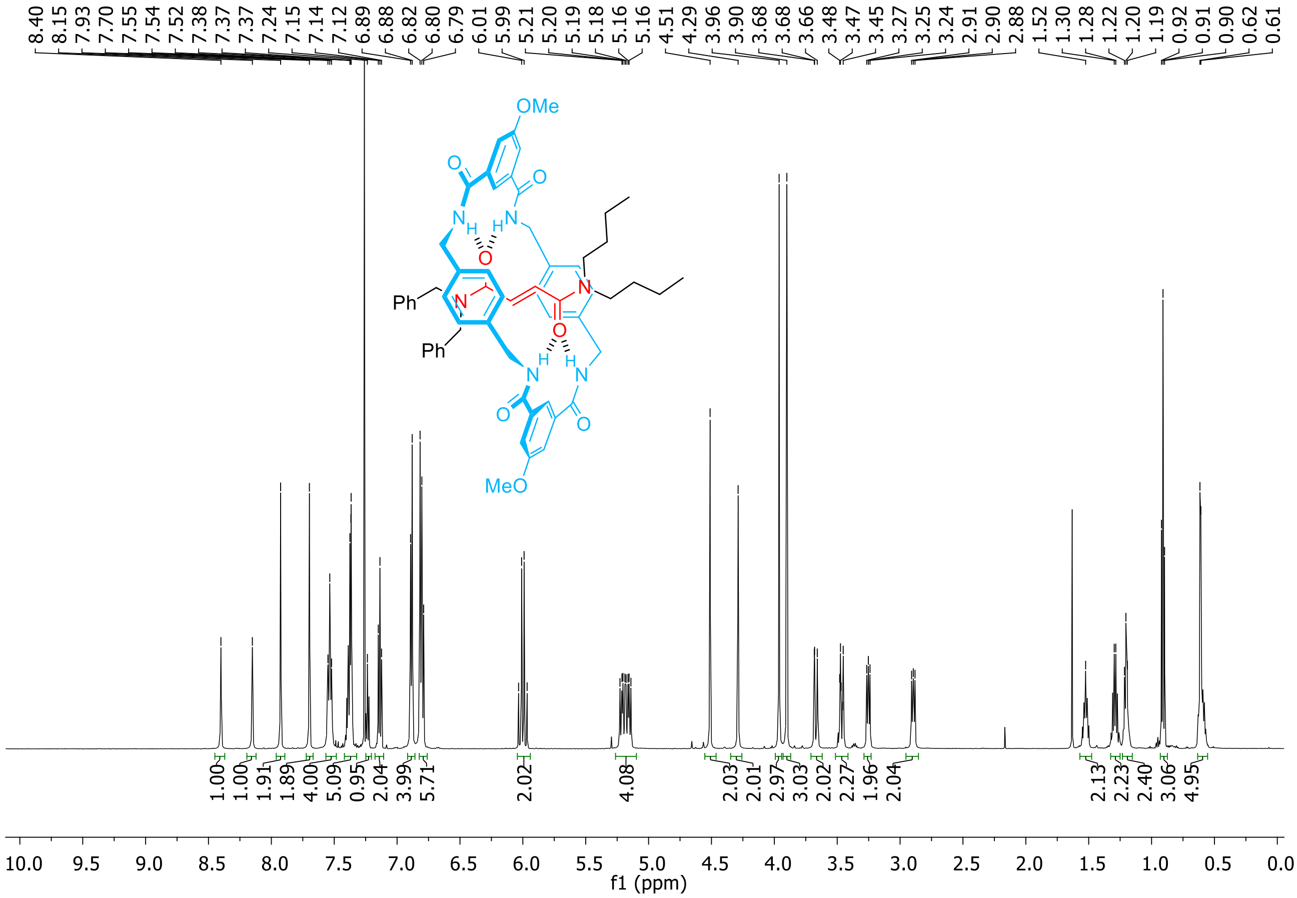

1e (^{13}C NMR, 151 MHz, $CDCl_3$, 298K)

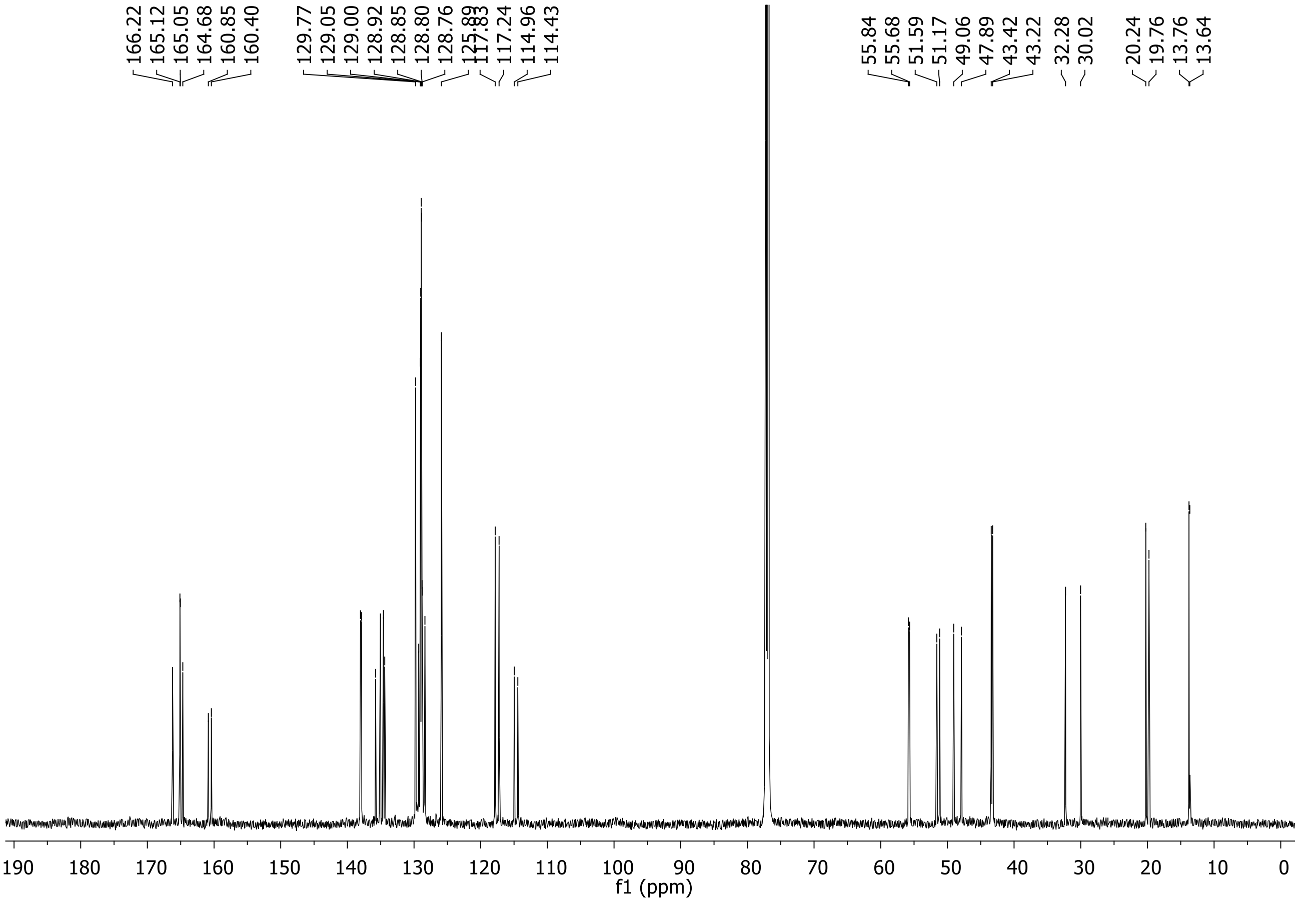

1f (^{1}H NMR, 400 MHz, $CDCl_3$, 298K)

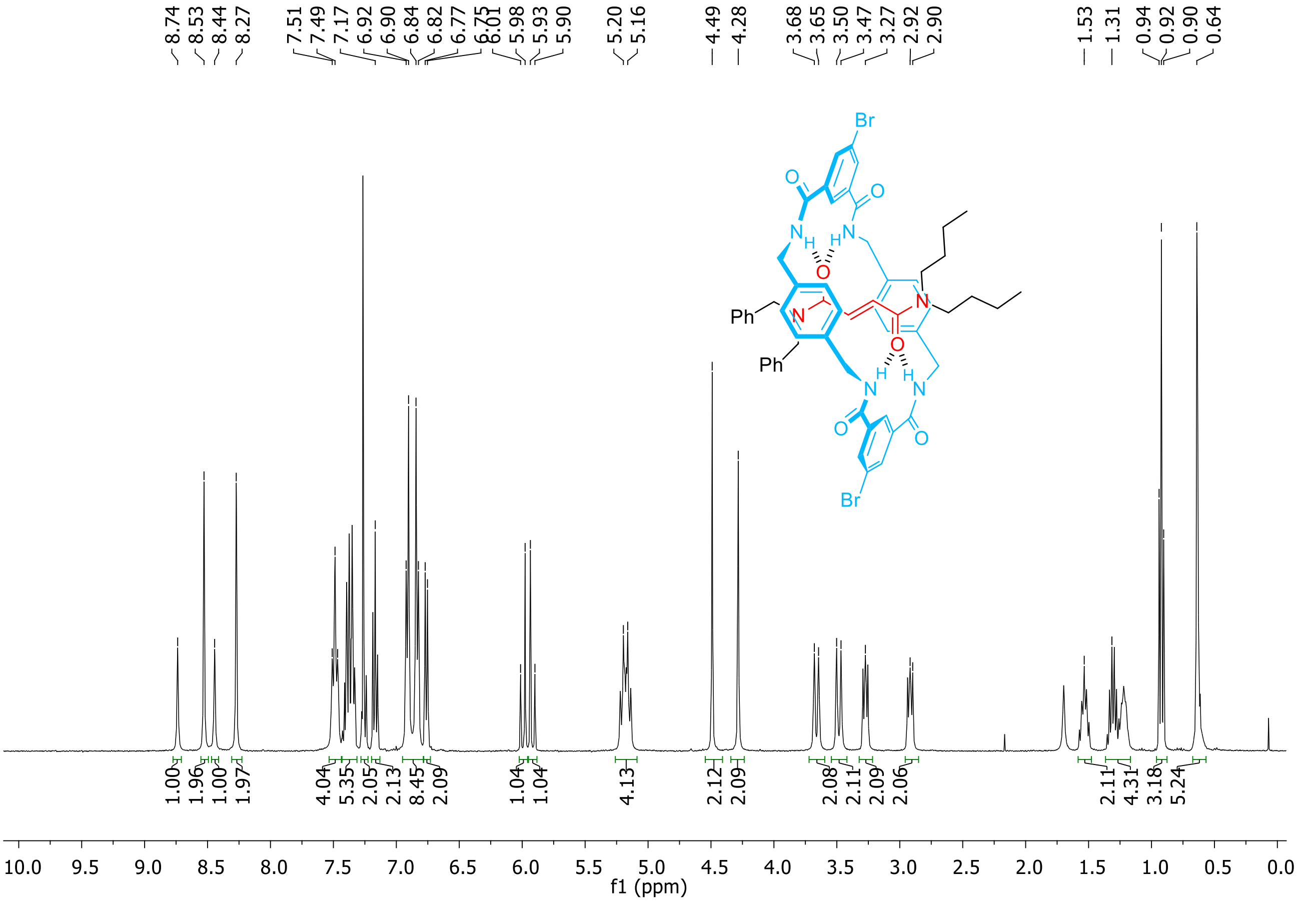

1f (^{13}C NMR, 101 MHz, $CDCl_3$, 298K)

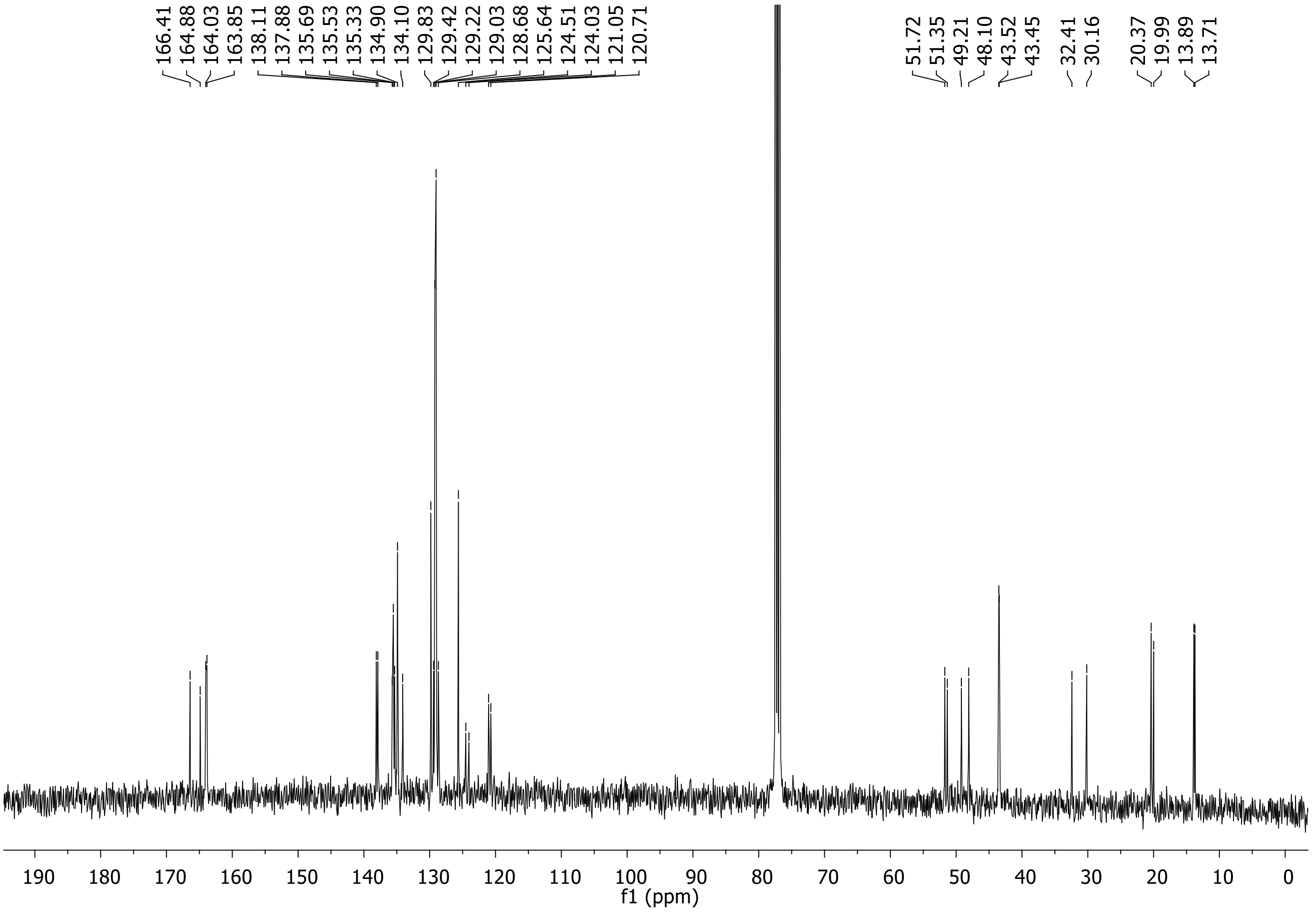

1g (^{1}H NMR, 600 MHz, $CDCl_3$, 298K)

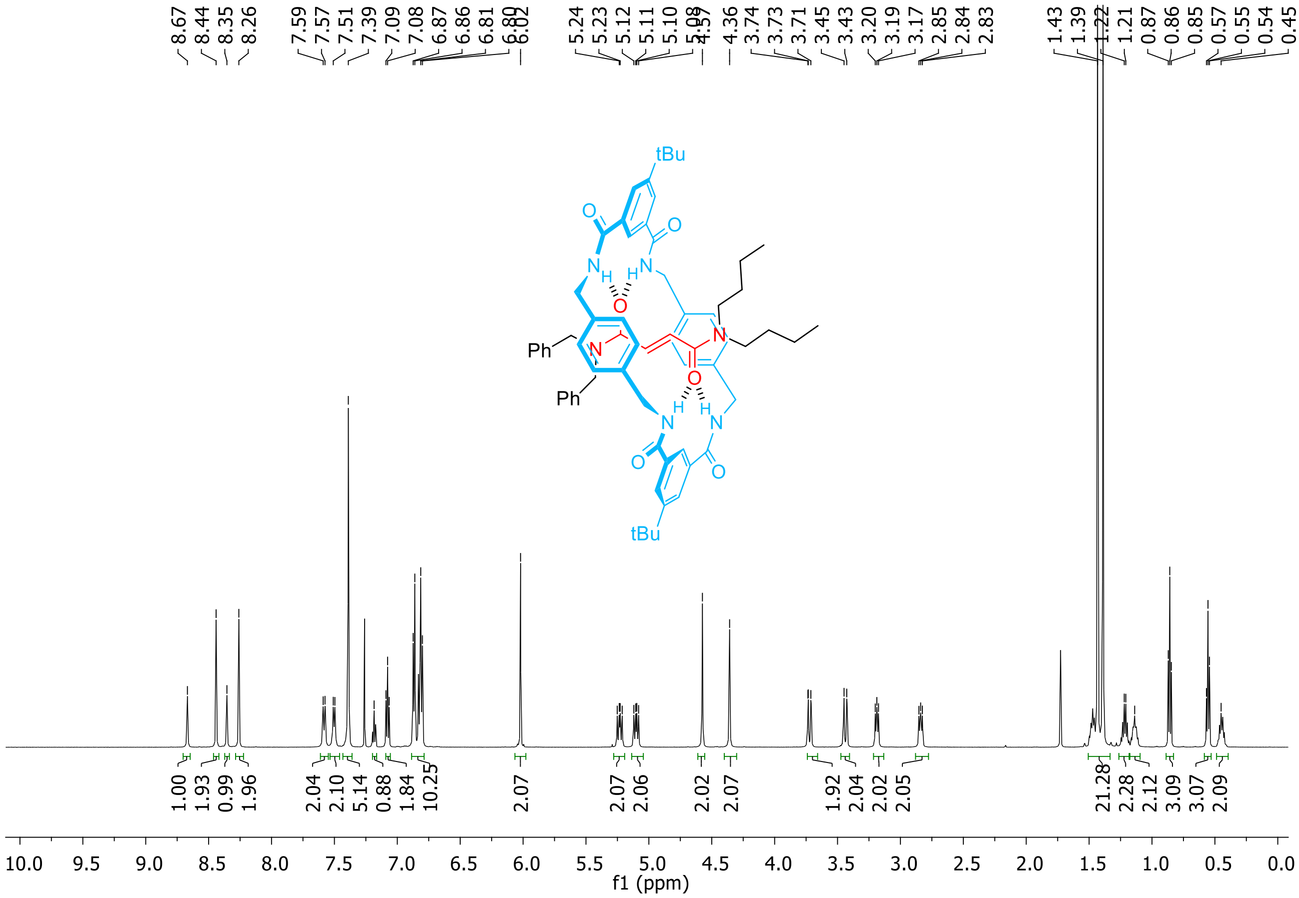

1g (^{13}C NMR, 151 MHz, $CDCl_3$, 298K)

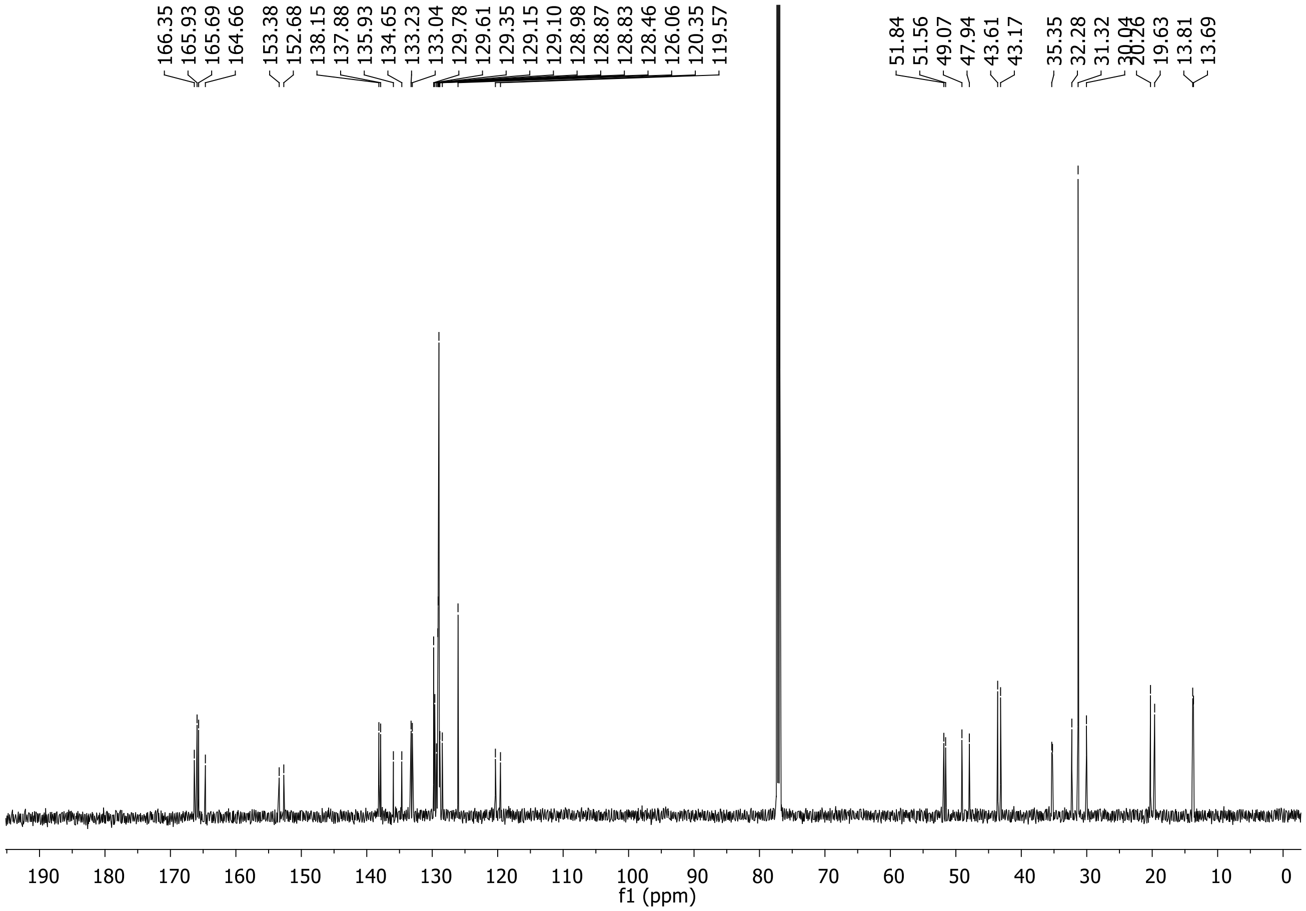

1h (^{1}H NMR, 300 MHz, $CDCl_3$, 298K)

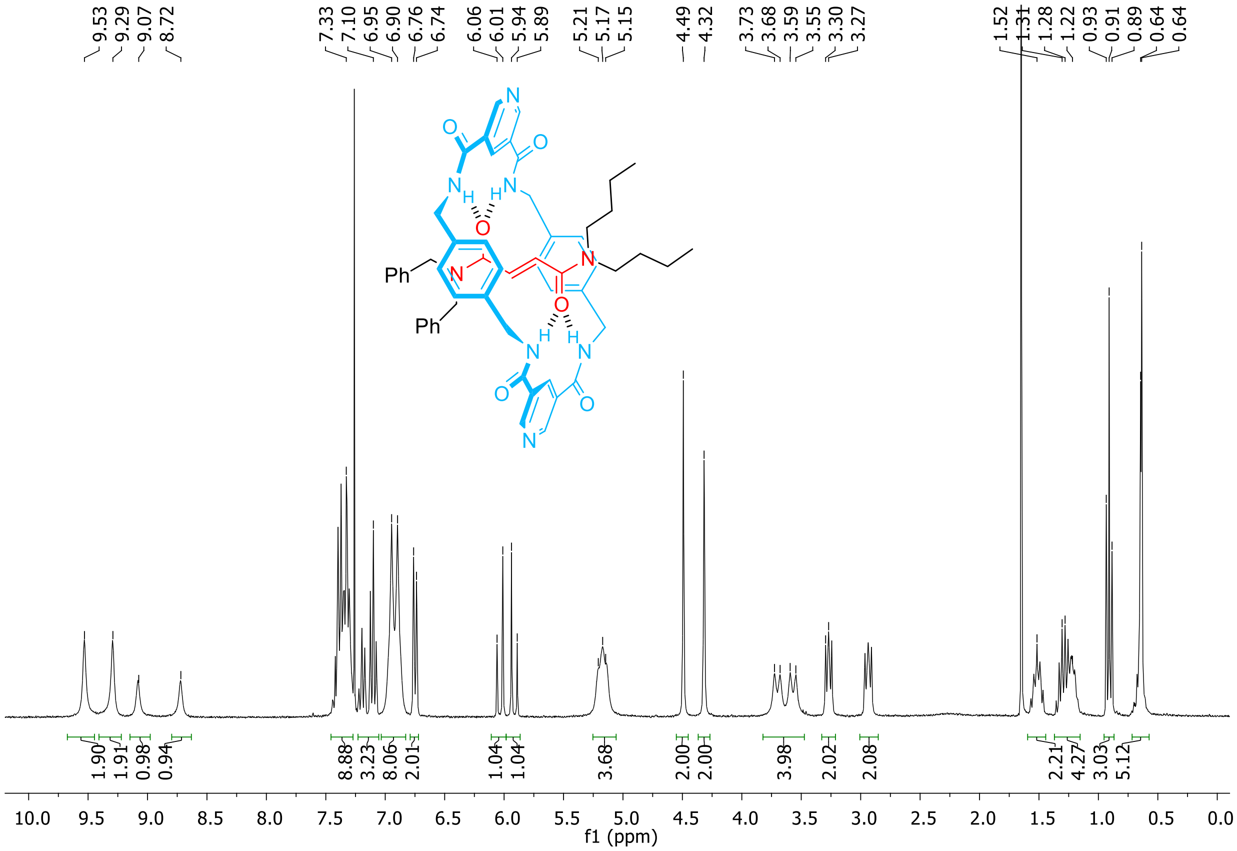

1h (^{13}C NMR, 75 MHz, $CDCl_3$, 298K)

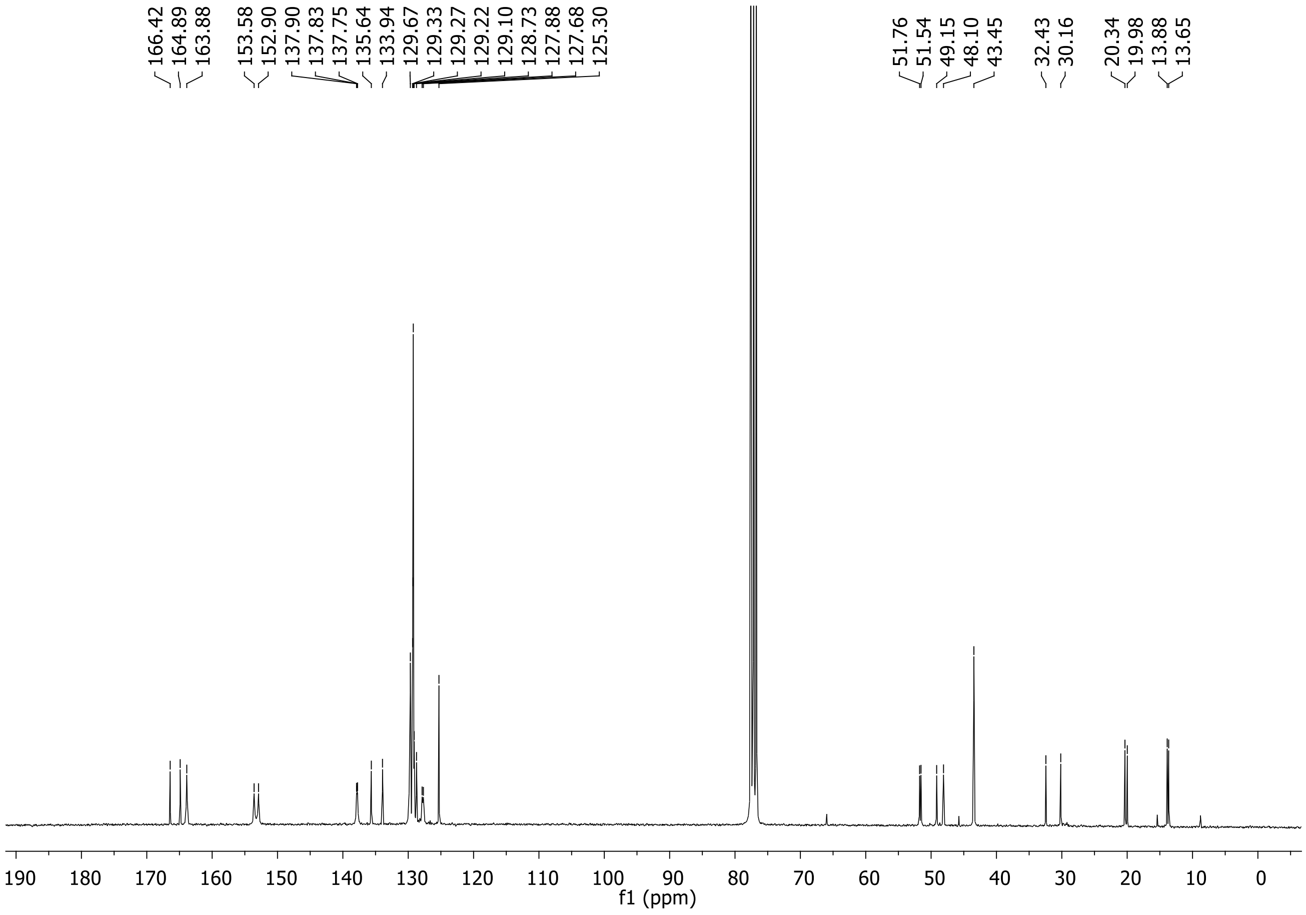

1i (^{1}H NMR, 400 MHz, $CDCl_3$, 298K)

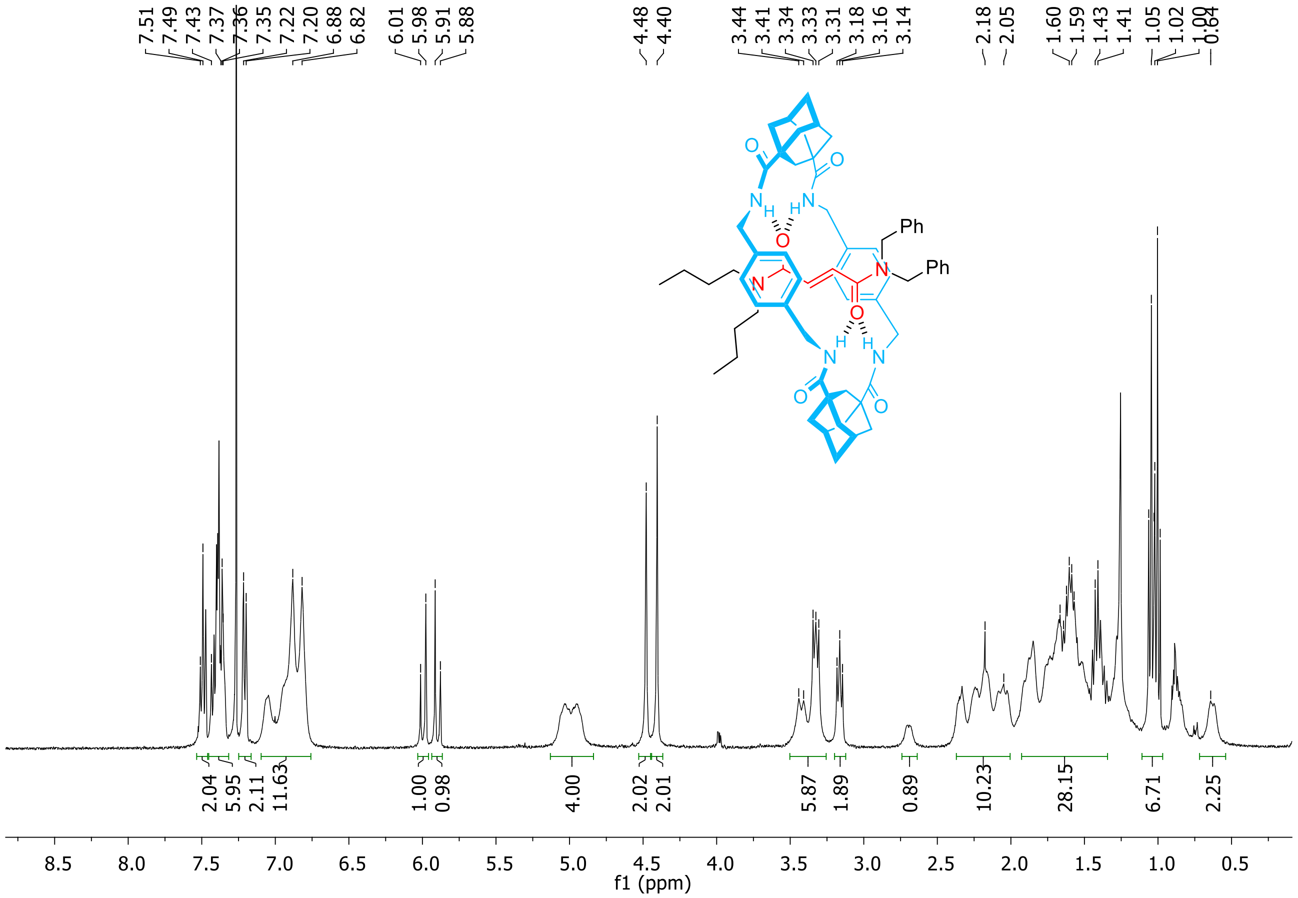

1i (^{13}C NMR, 101 MHz, $CDCl_3$, 298K)

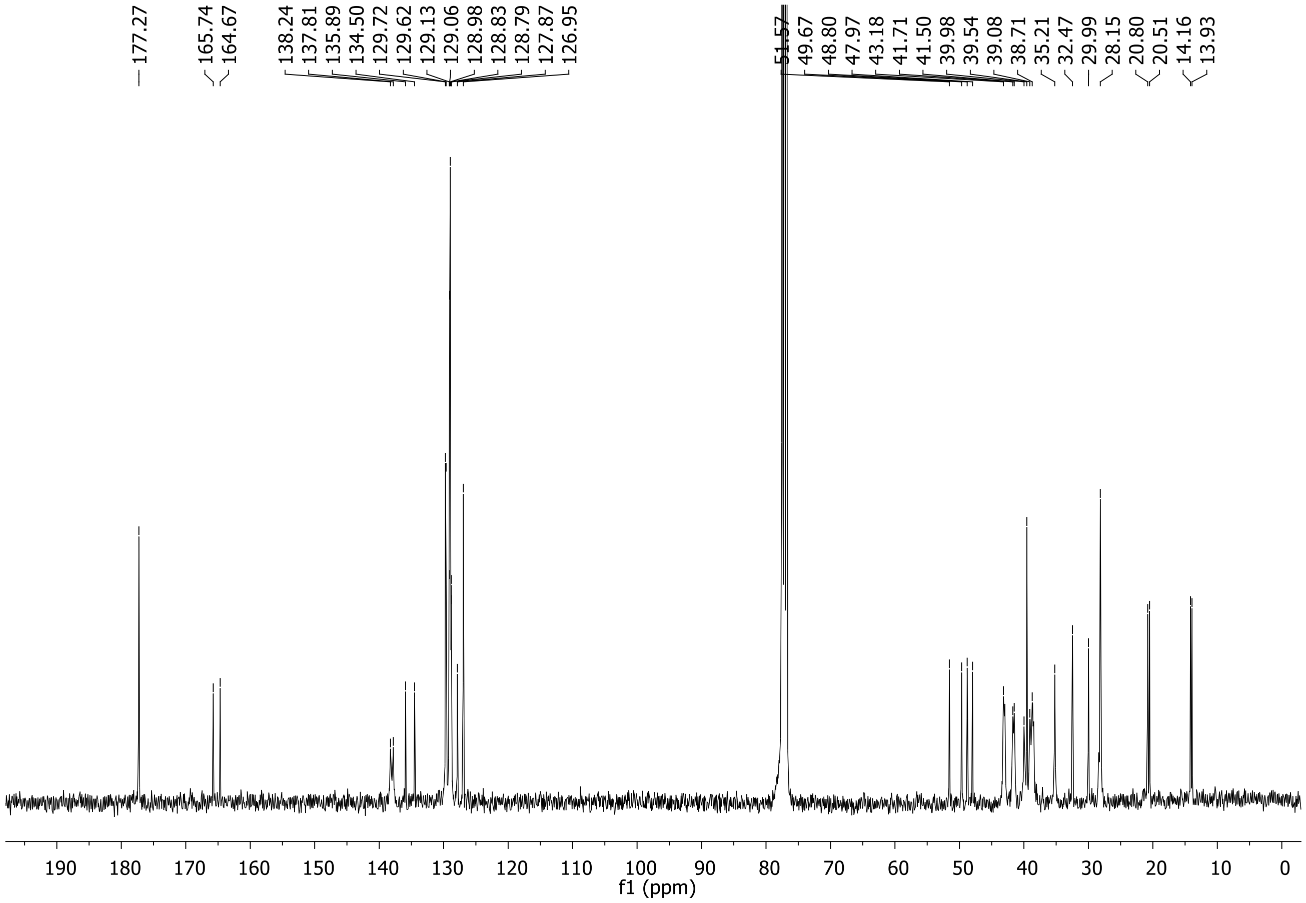

1a-*d₂* (^{1}H NMR, 400 MHz, $CDCl_3$, 298K)

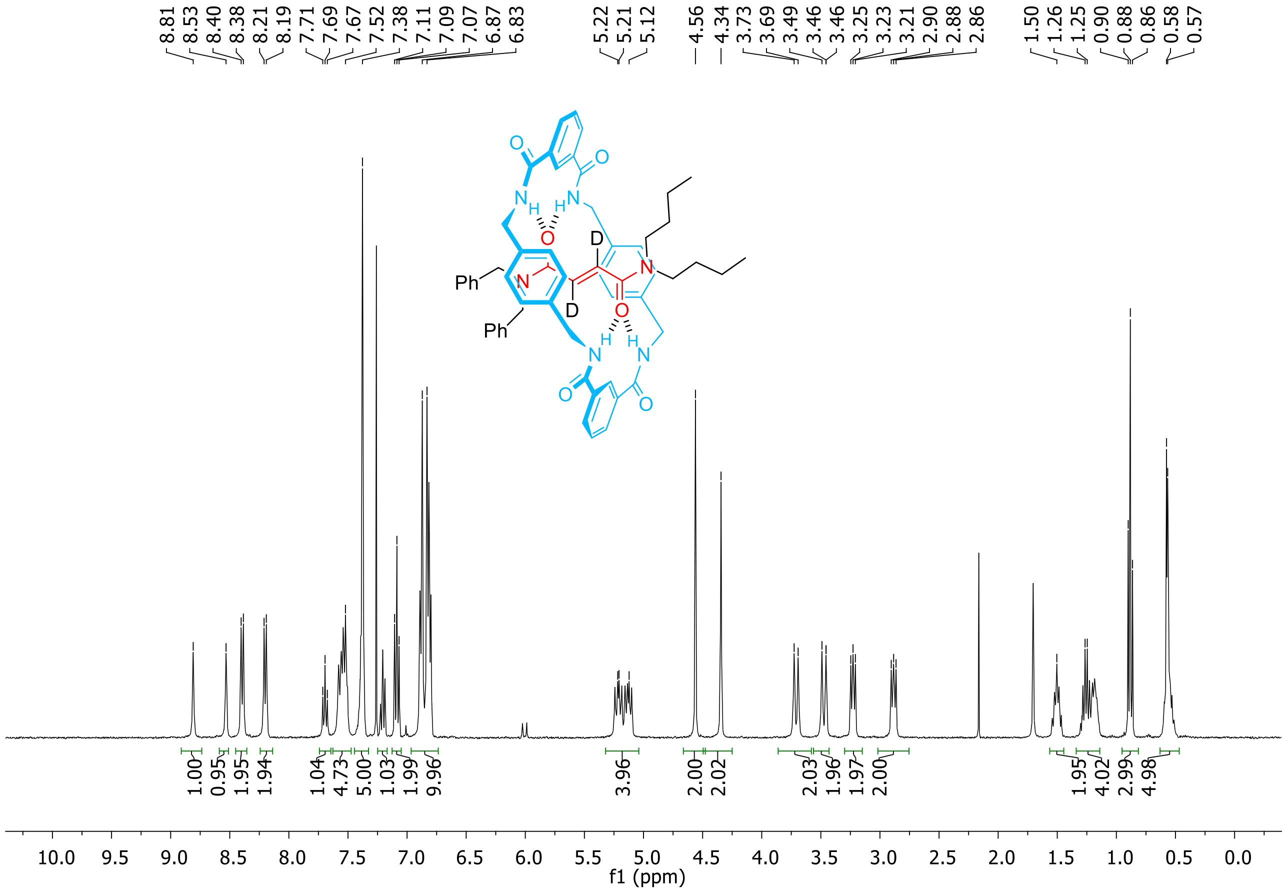

1a-*d₂* (^{13}C NMR, 75 MHz, $CDCl_3$, 298K)

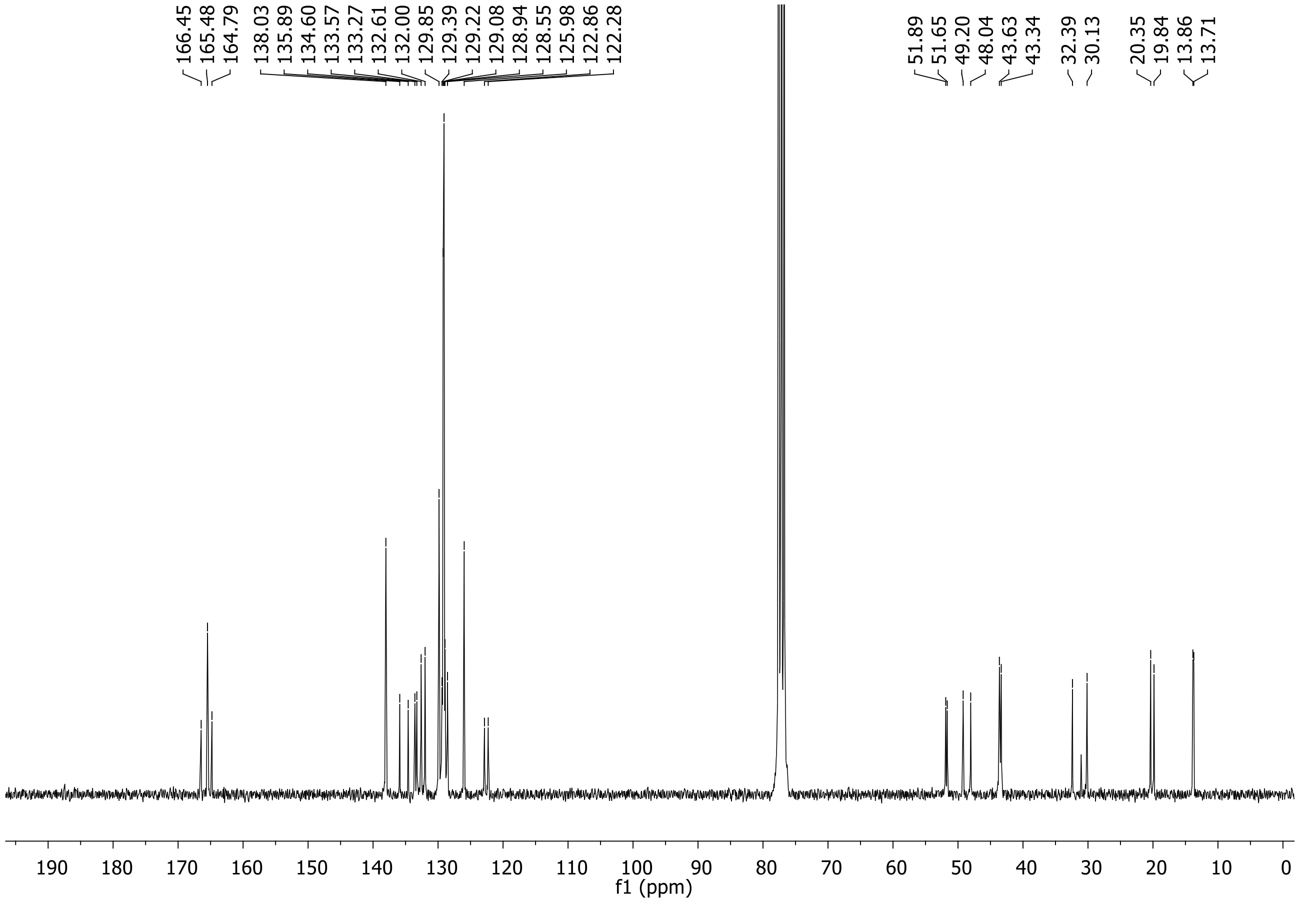

4-*d*₂ (^{1}H NMR, 300 MHz, $CDCl_3$, 298K)

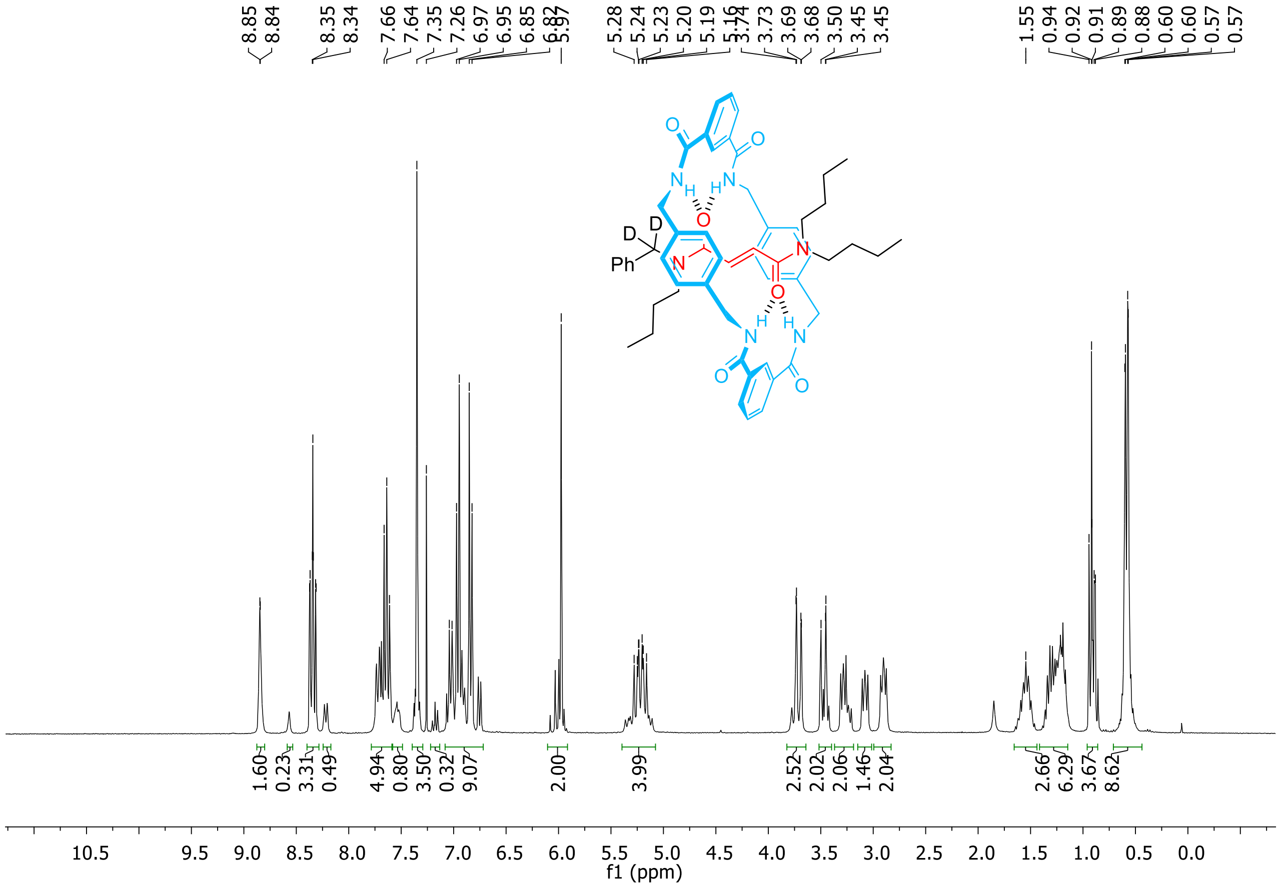

4-*d*₂ (^{13}C NMR, 75 MHz, $CDCl_3$, 298K)

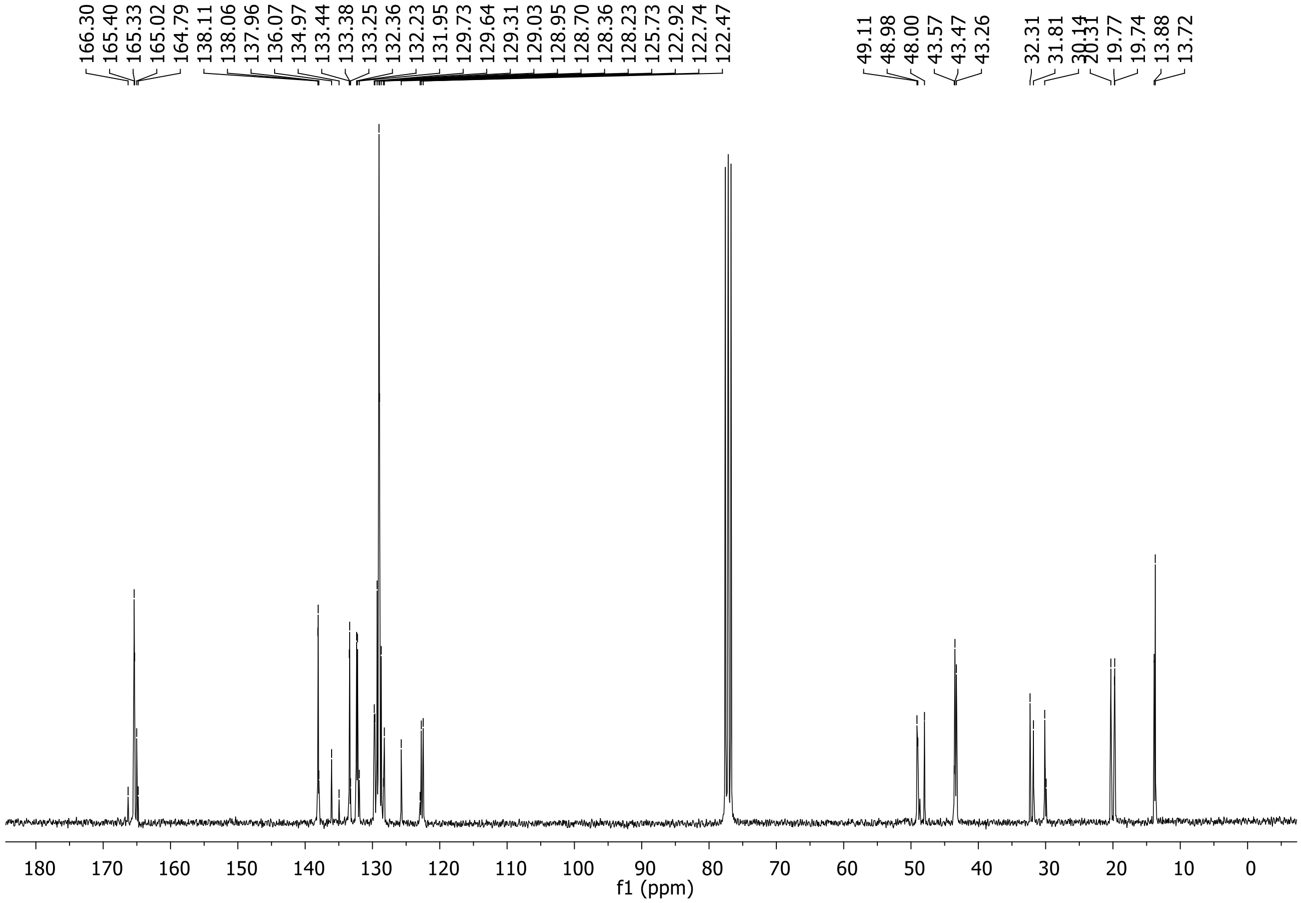

7i (^{1}H NMR, 400 MHz, $CDCl_3$, 298K)

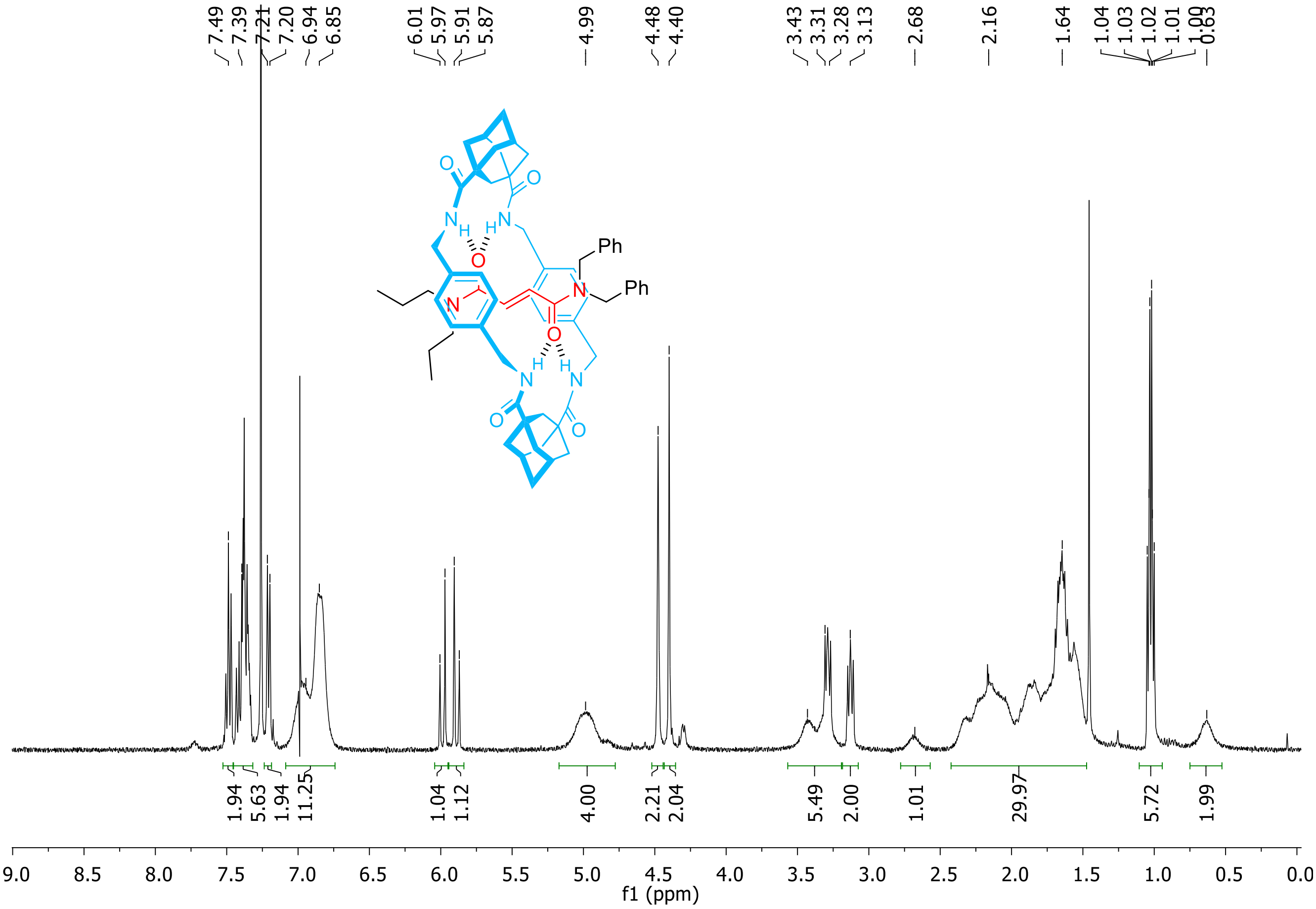

7i (^{13}C NMR, 75 MHz, $CDCl_3$, 298K)

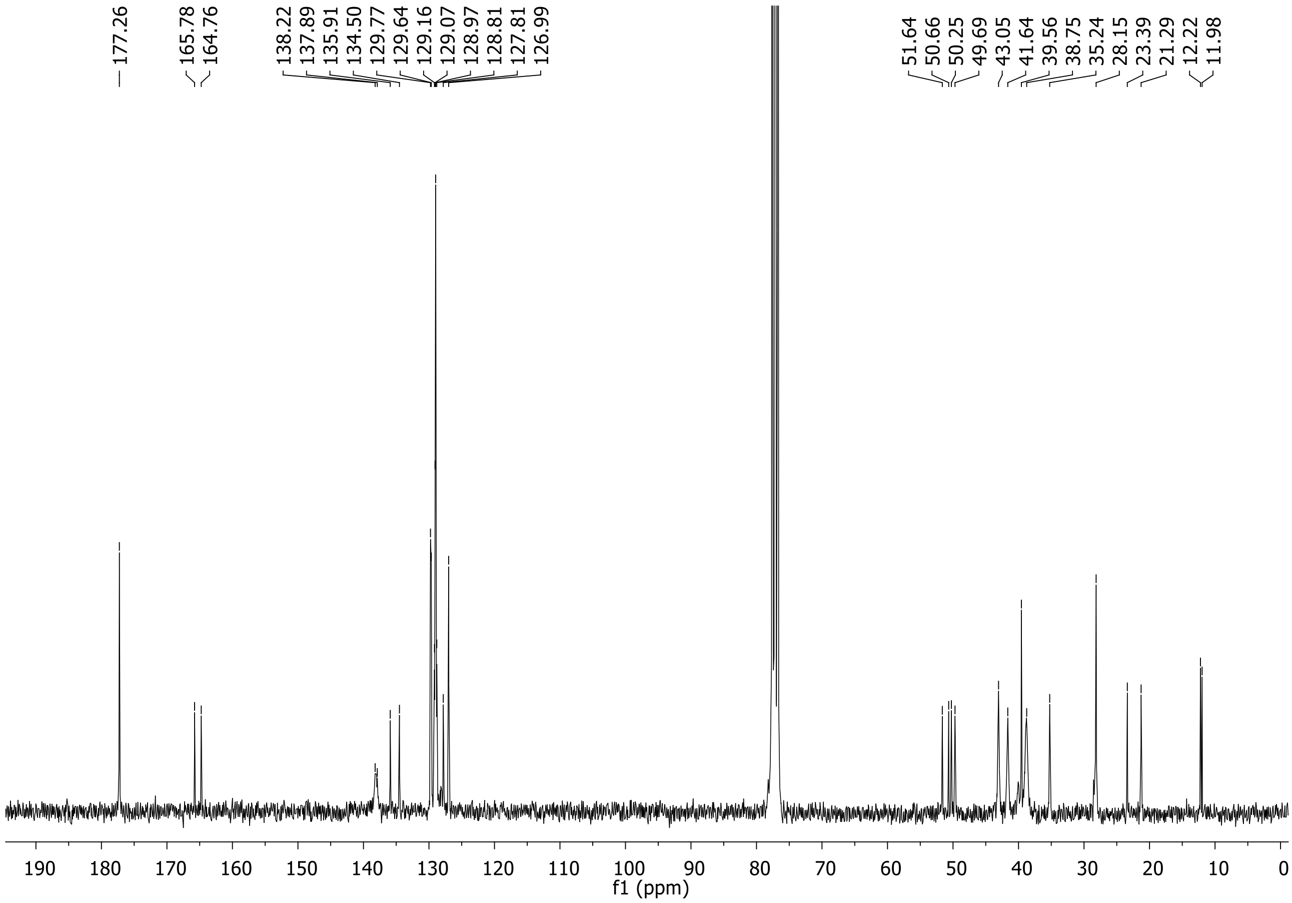

***trans*-2a** (^{1}H NMR, 300 MHz, $CDCl_3$, 298K)

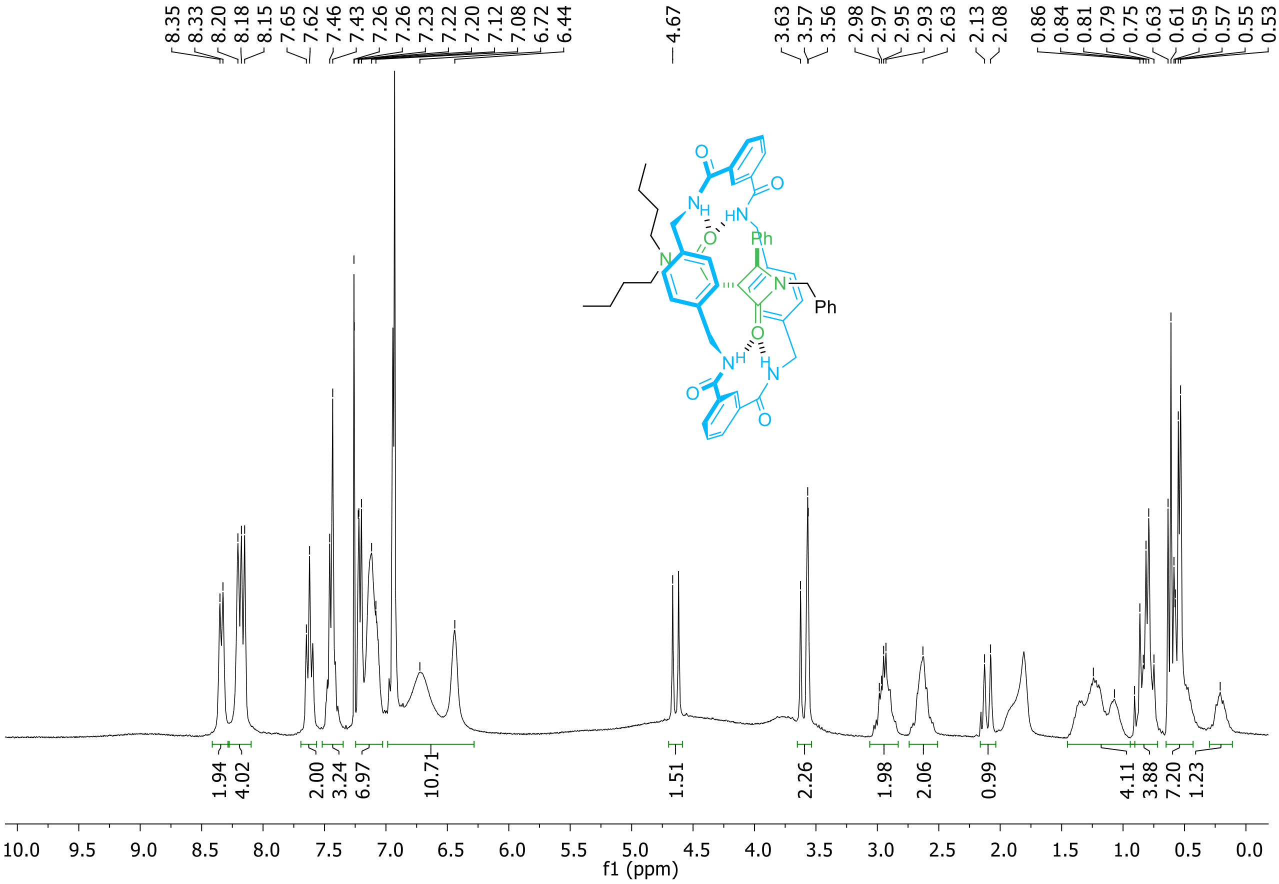

***trans*-2a** (^{13}C NMR, 75 MHz, $CDCl_3$, 298K)

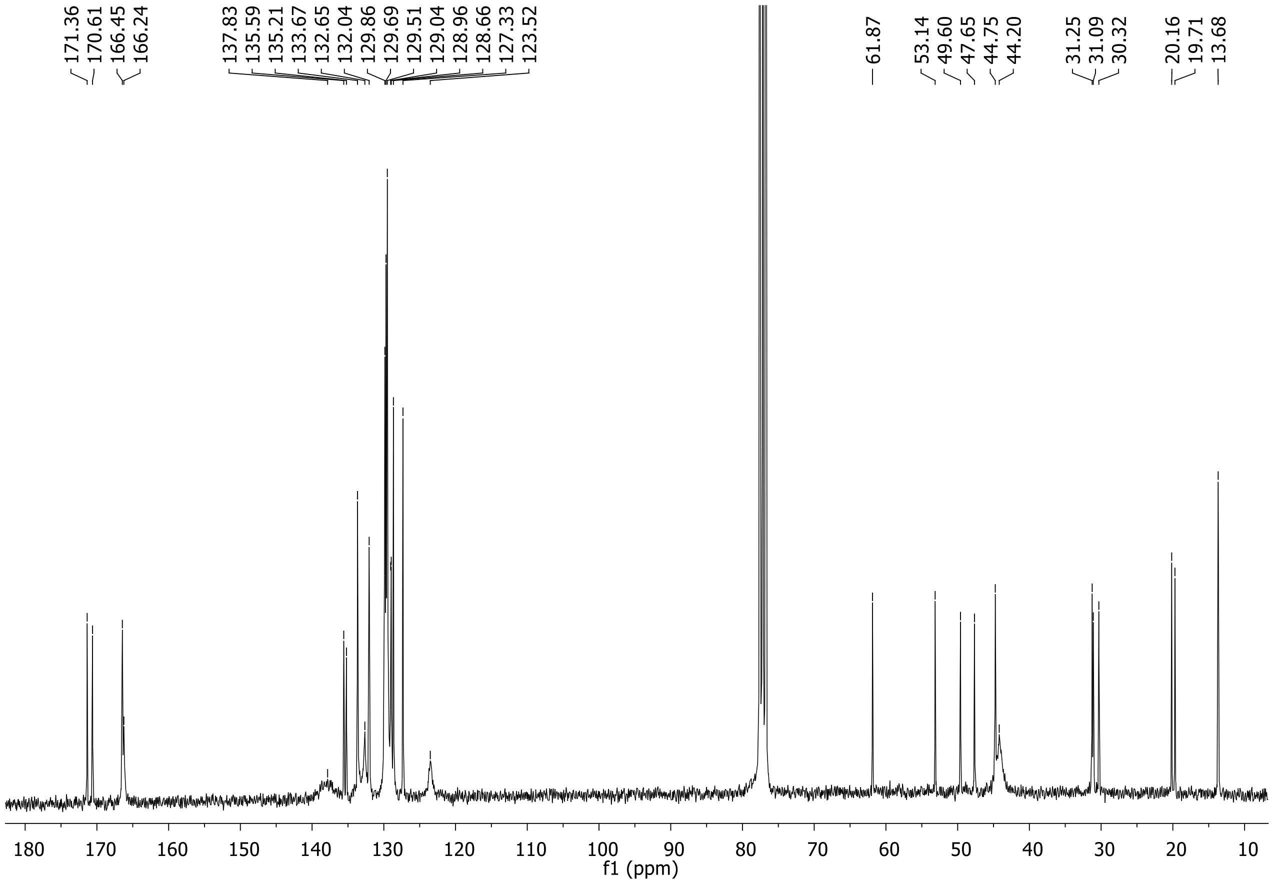

***trans*-2d** (^{1}H NMR, 400 MHz, $CDCl_3$, 328K)

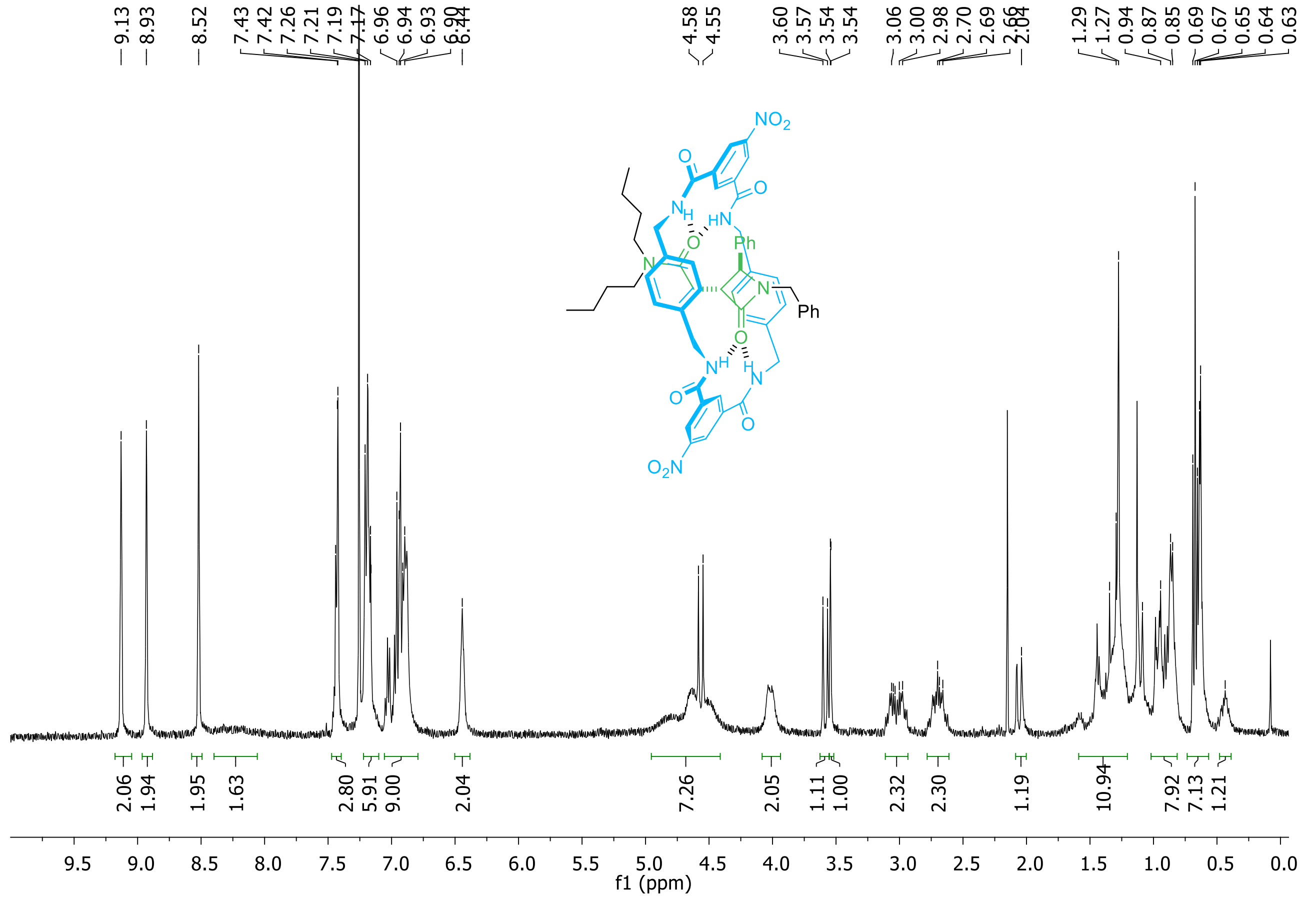

***trans*-2d** (^{13}C NMR, 101 MHz, $CDCl_3$, 328K)

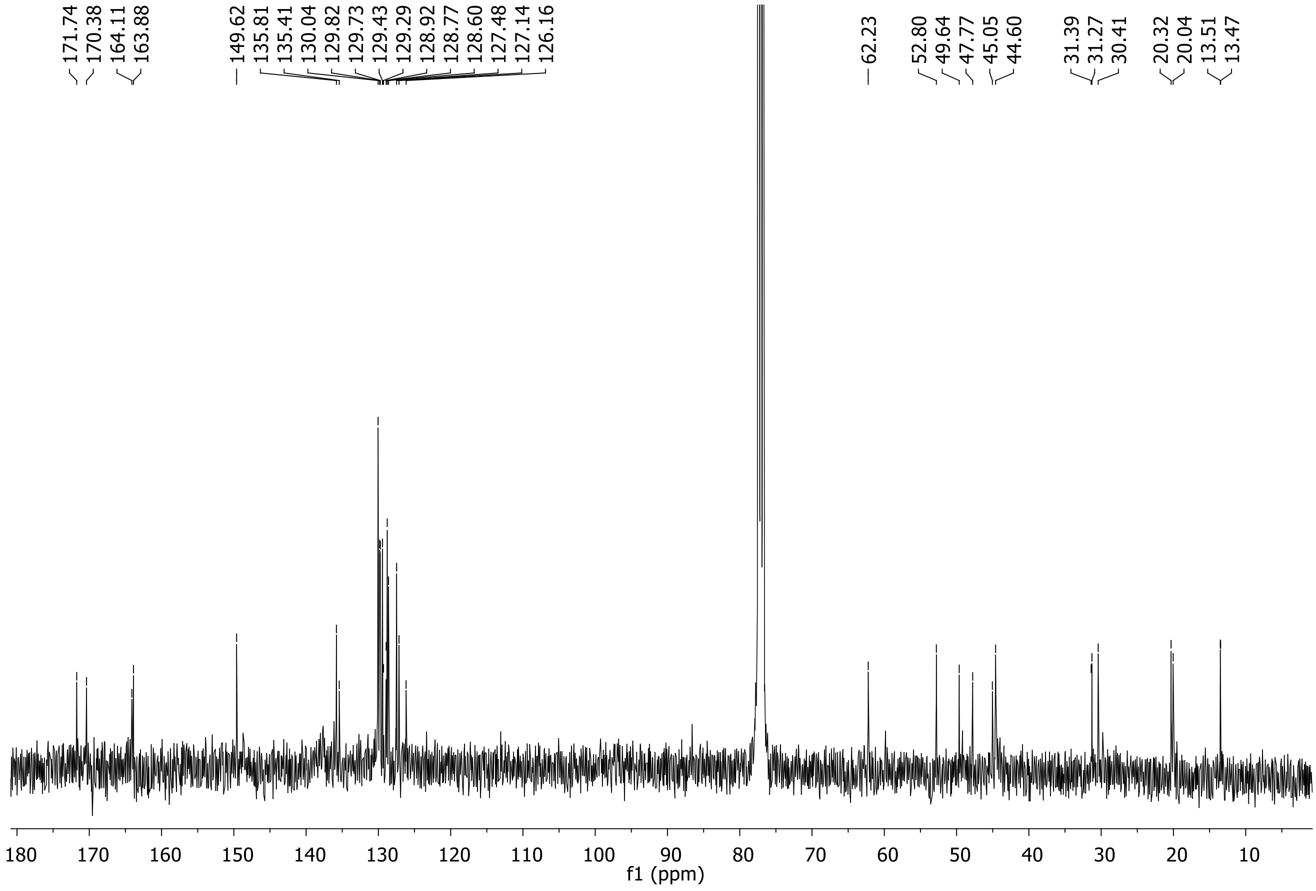

***trans*-2e** (^{1}H NMR, 400 MHz, $CDCl_3$, 328K)

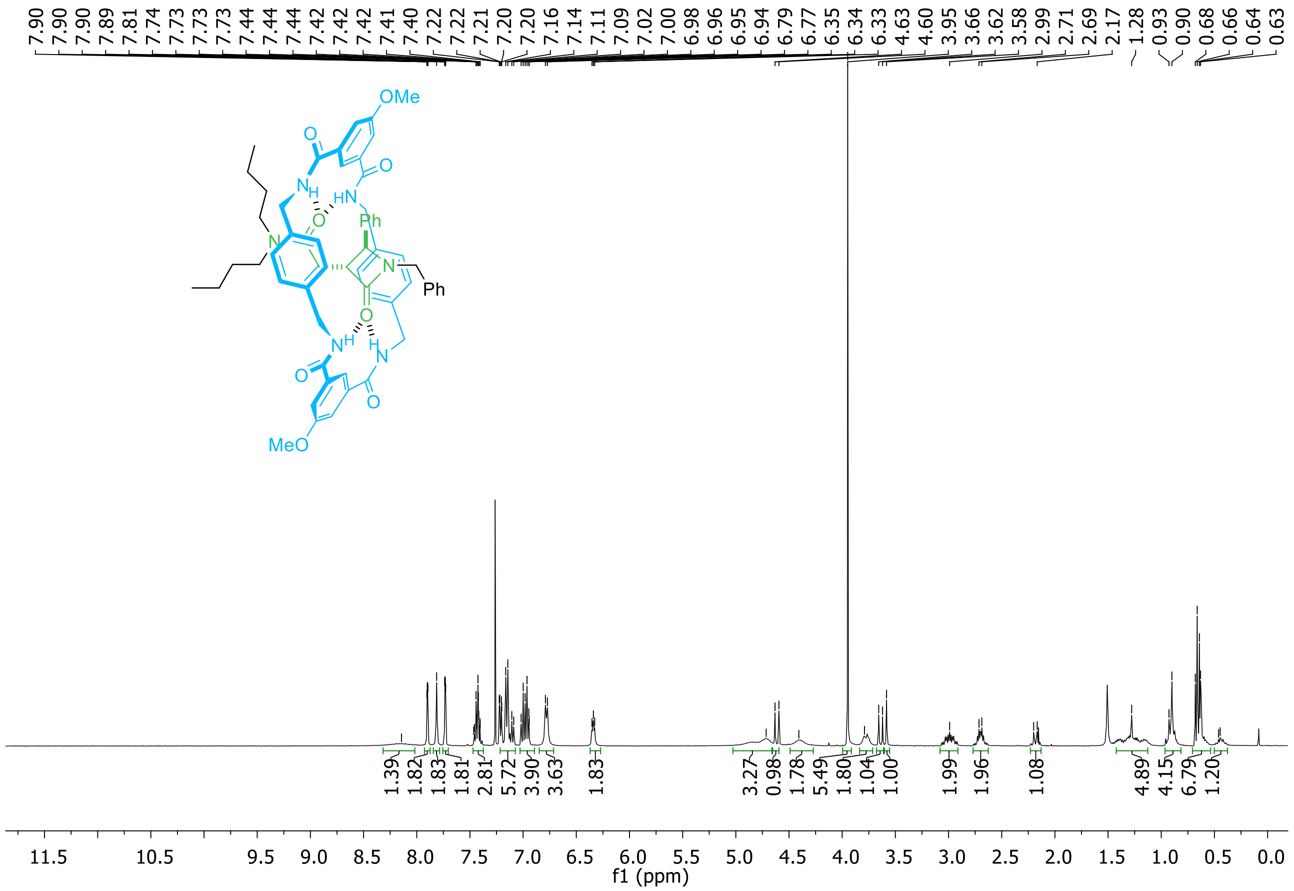

***trans*-2e** (^{13}C NMR, 101 MHz, $CDCl_3$, 328K)

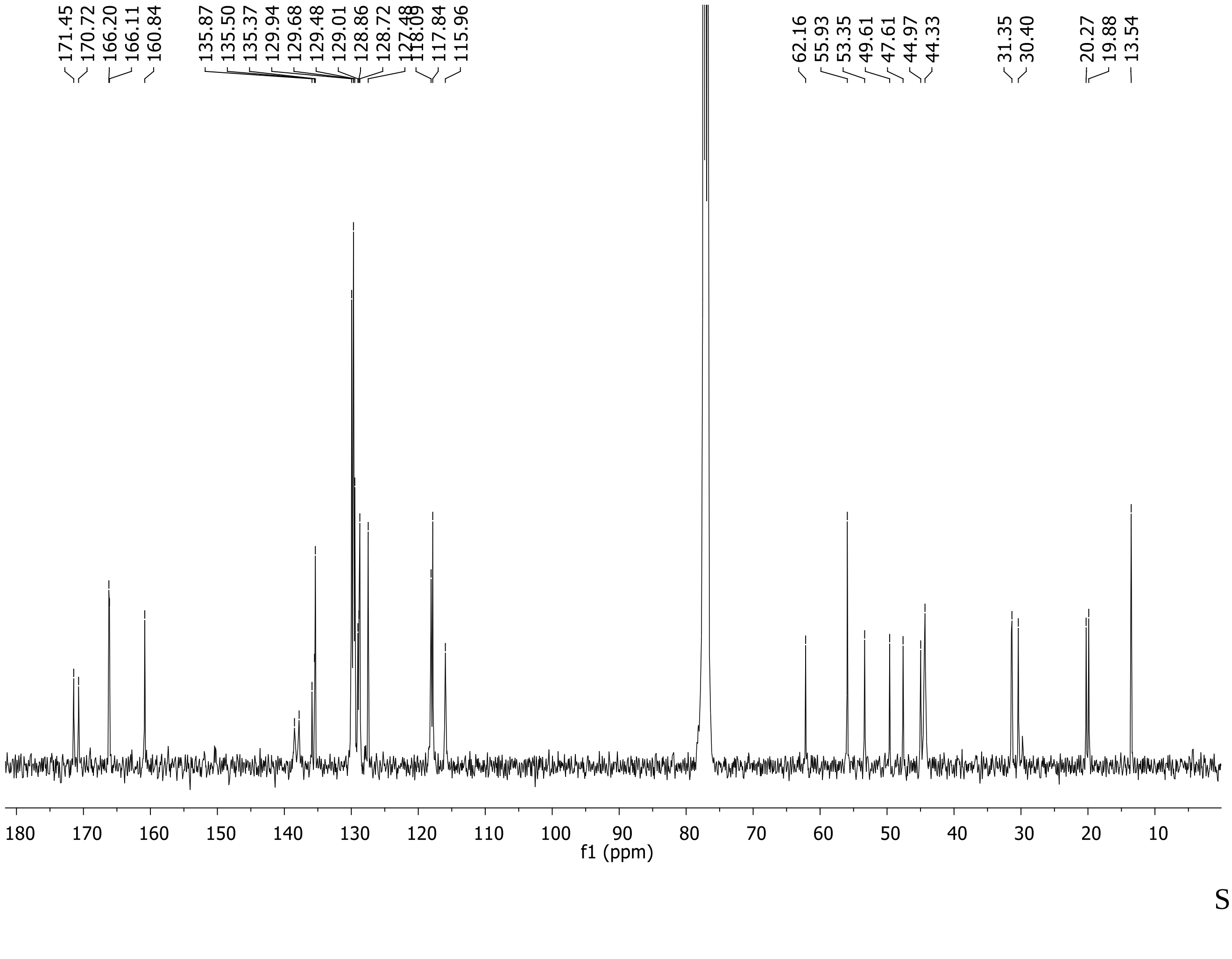

***trans*-2f** (^{1}H NMR, 400 MHz, $CDCl_3$, 328K)

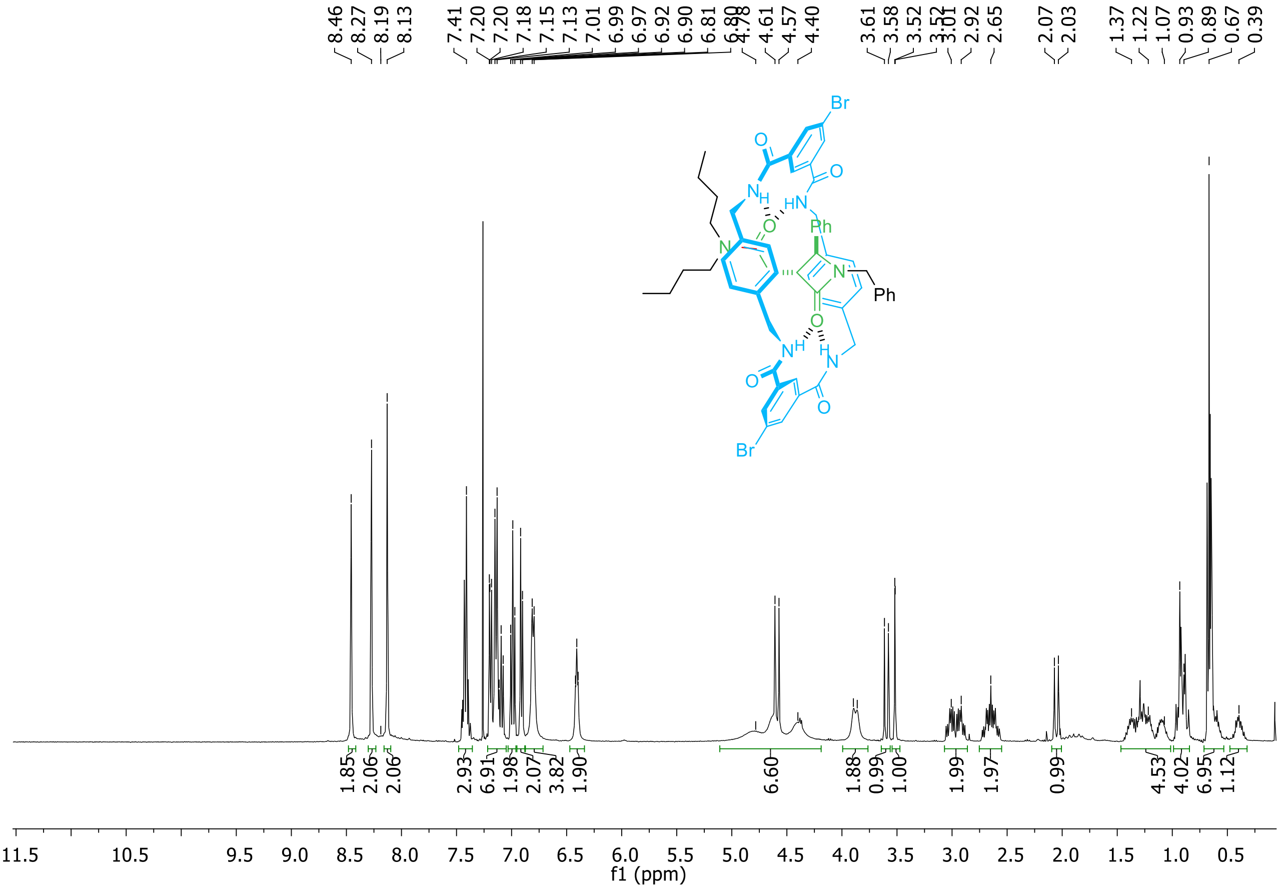

***trans*-2f** (^{13}C NMR, 101 MHz, $CDCl_3$, 328K)

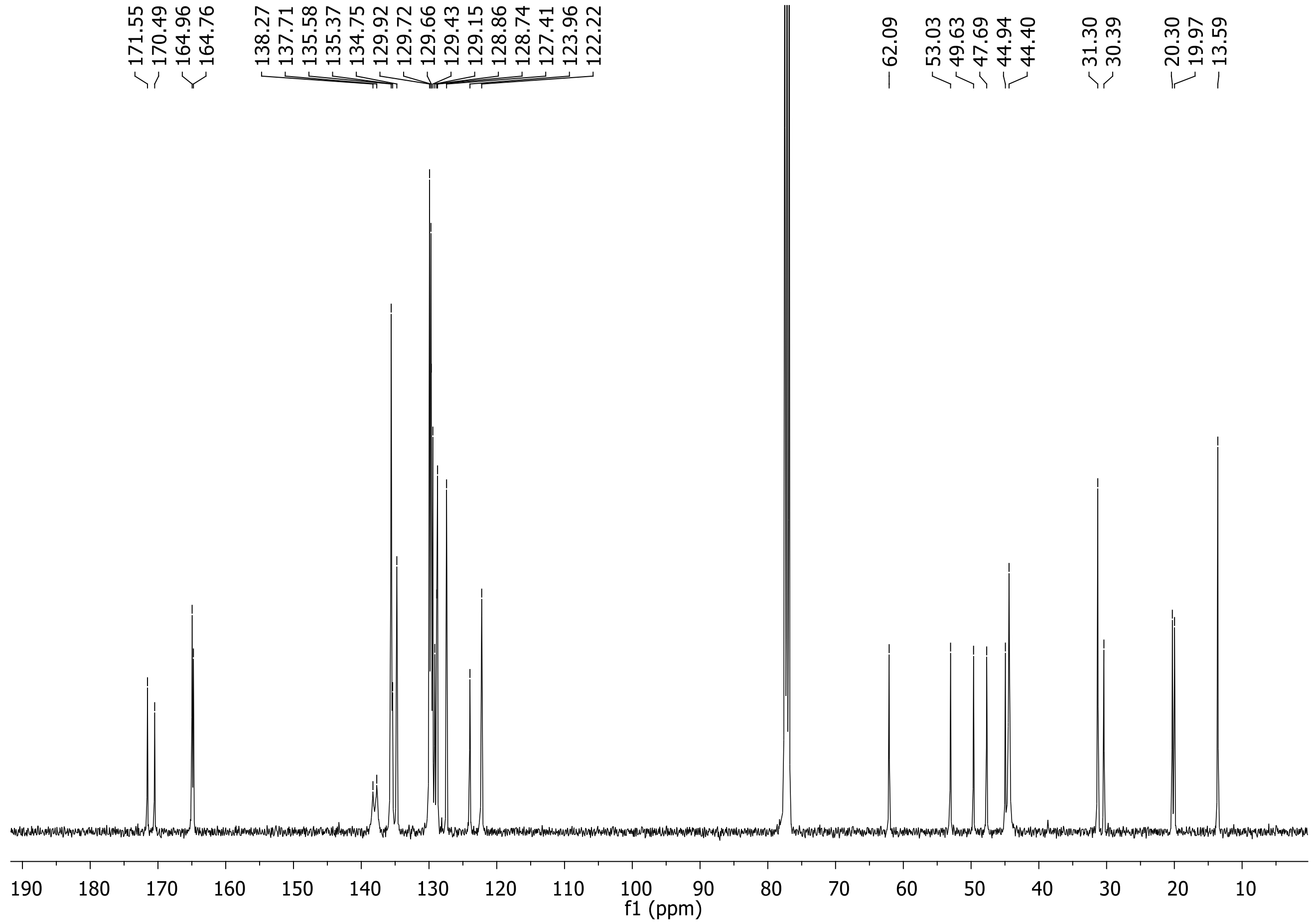

***trans*-2g** (^{1}H NMR, 400 MHz, $CDCl_3$, 328K)

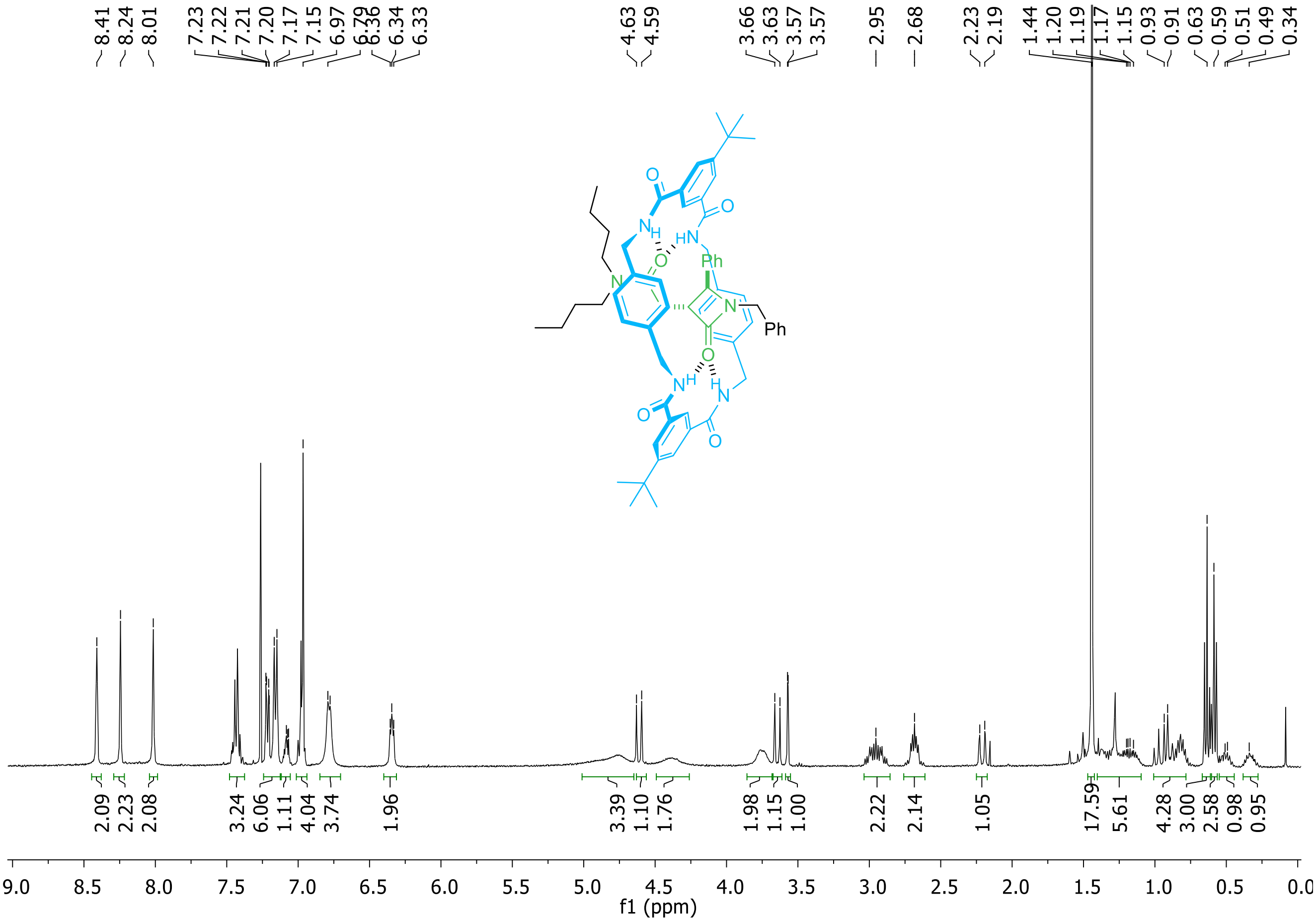

***trans*-2g** (^{13}C NMR, 101 MHz, $CDCl_3$, 328K)

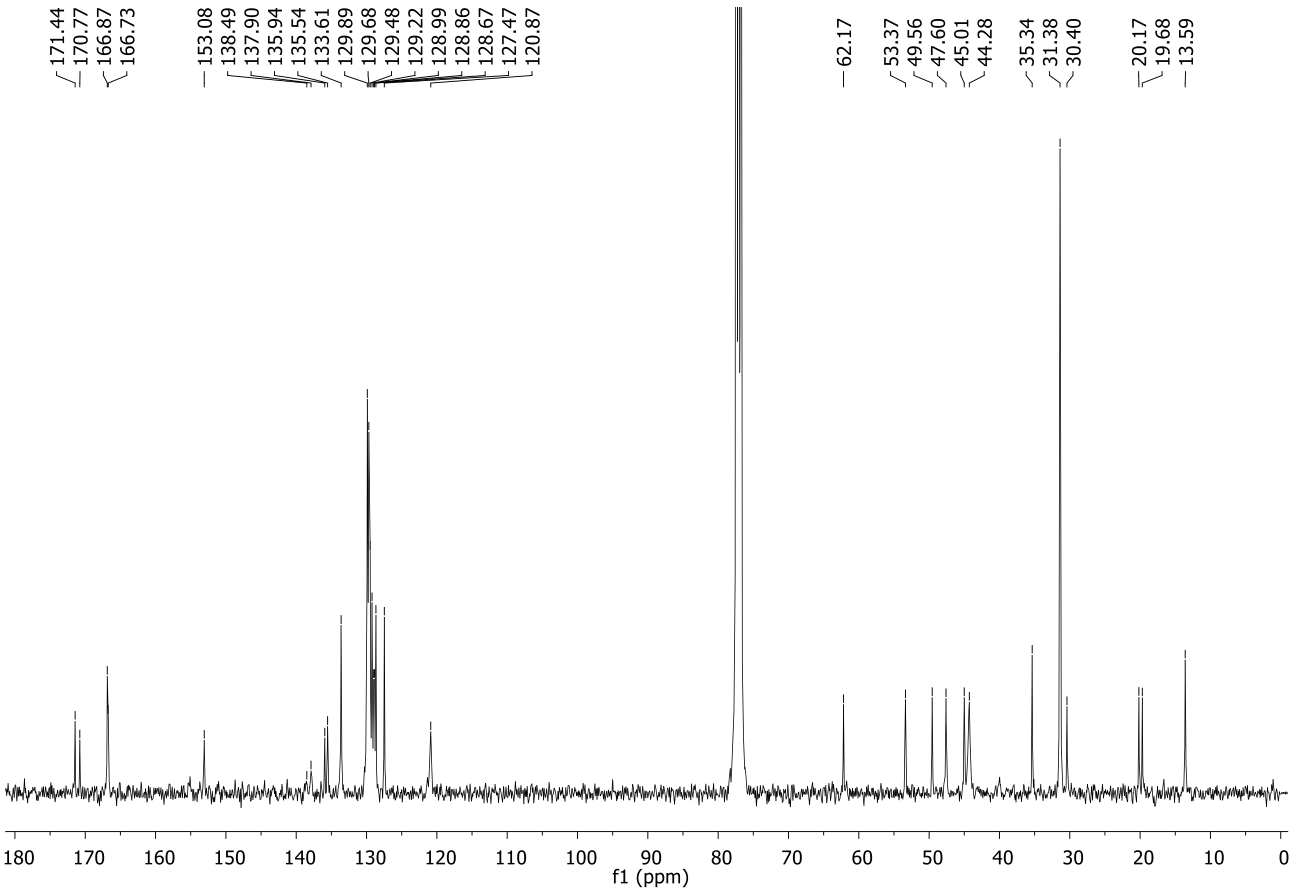

***trans*-2h** (^{1}H NMR, 300 MHz, $CDCl_3$, 298K)

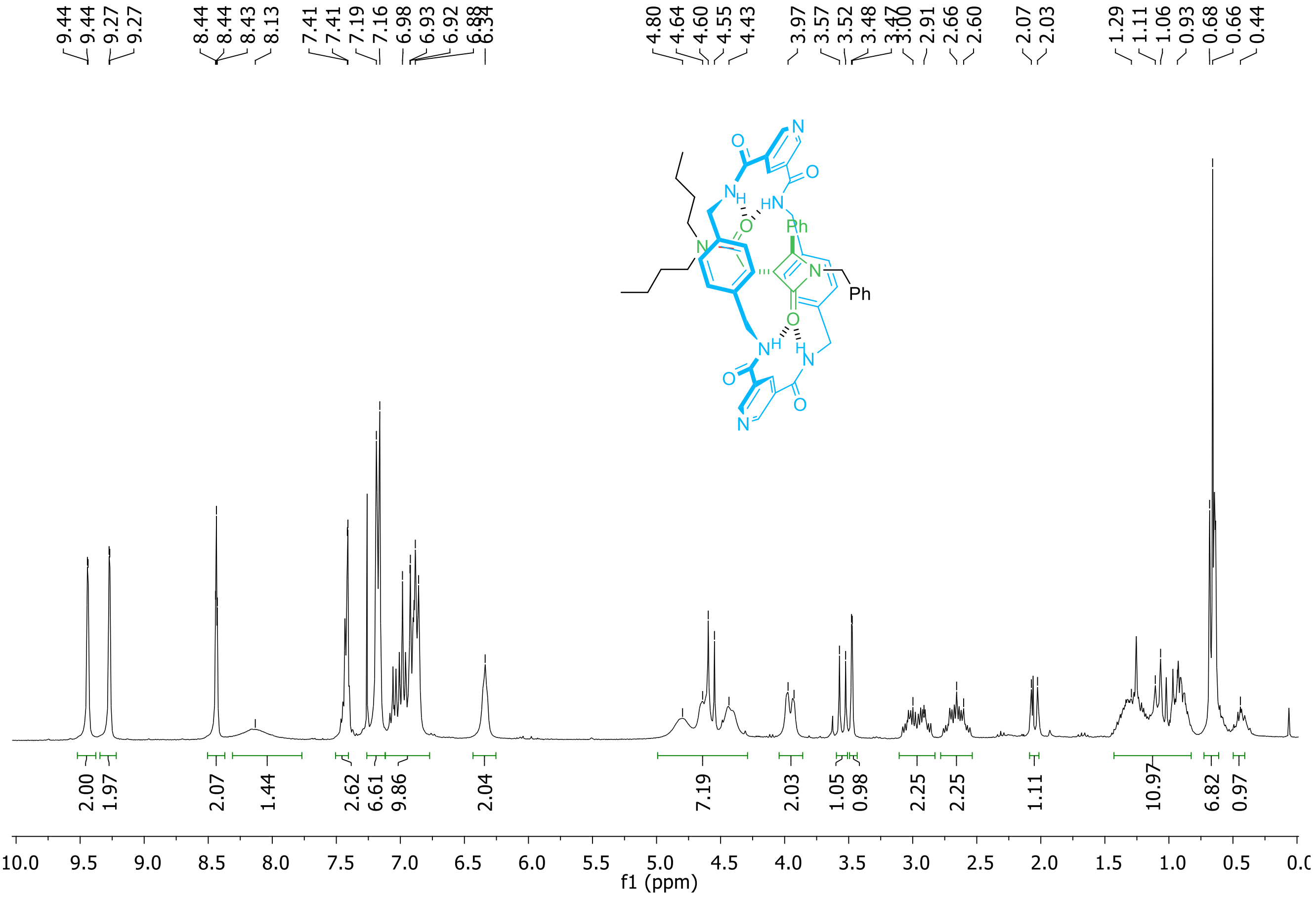

***trans*-2h** (^{13}C NMR, 75 MHz, $CDCl_3$, 298K)

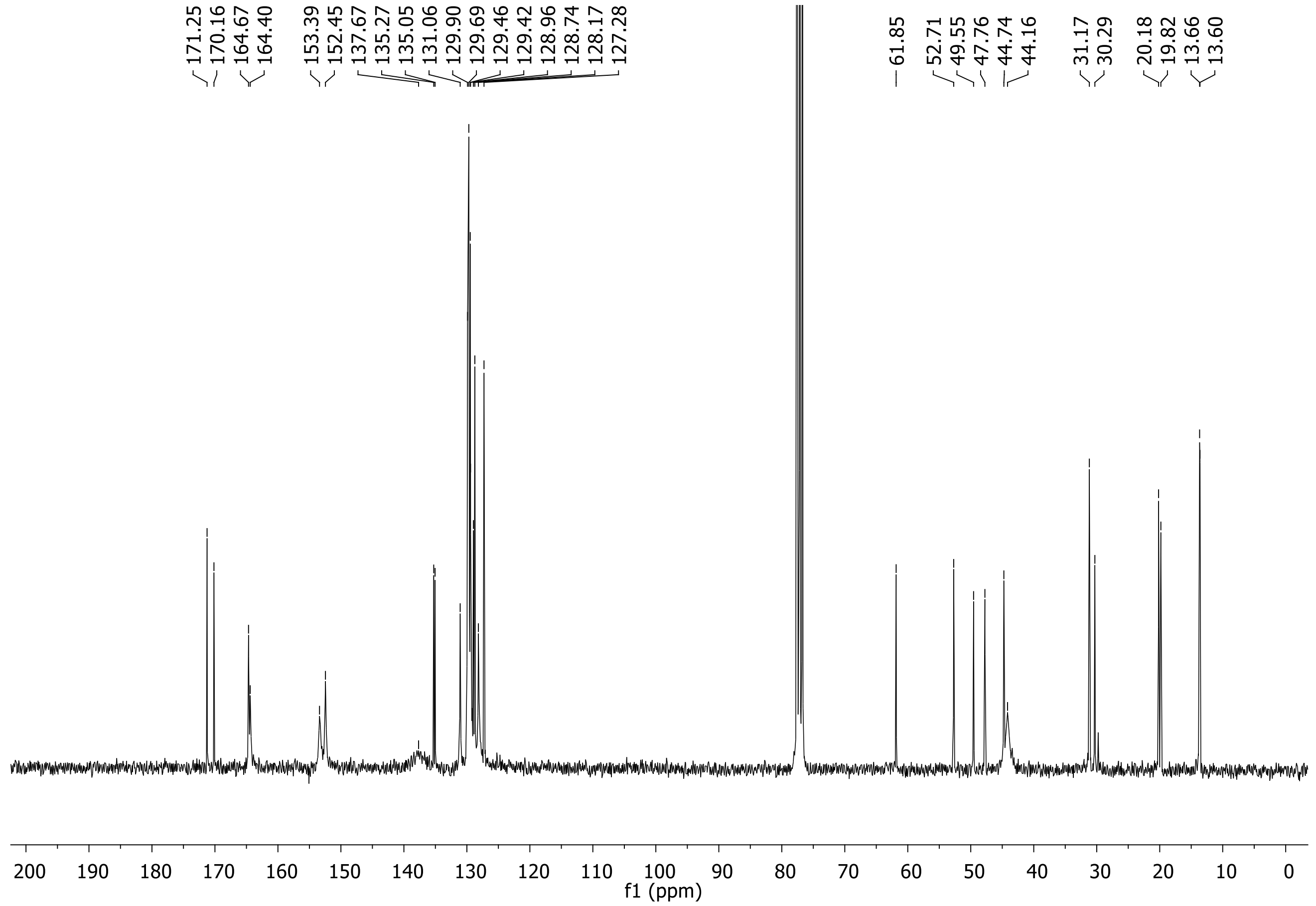

***trans*-2i** (^{1}H NMR, 400 MHz, $CDCl_3$, 328K)

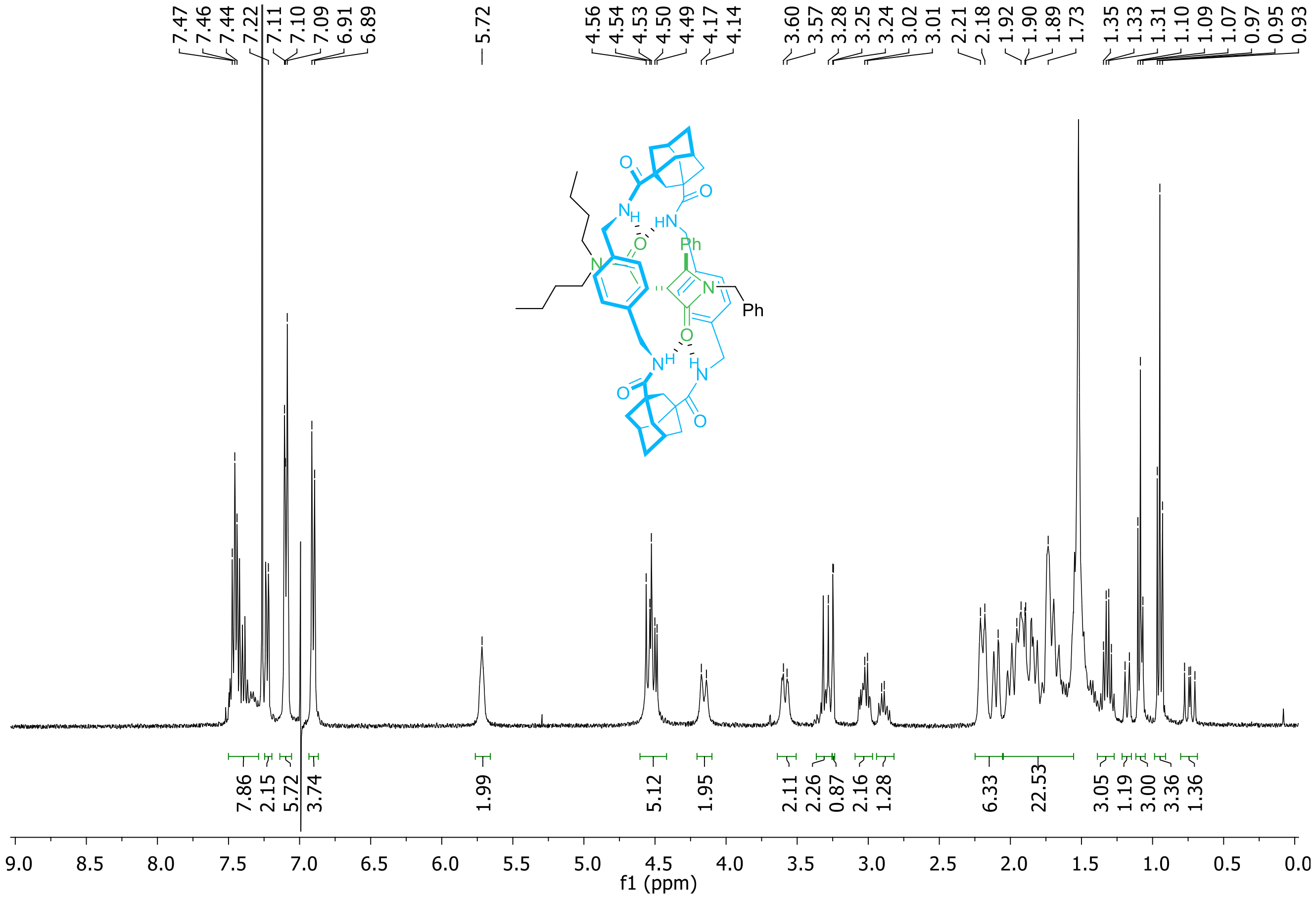

***trans*-2i** (^{13}C NMR, 101 MHz, $CDCl_3$, 328K)

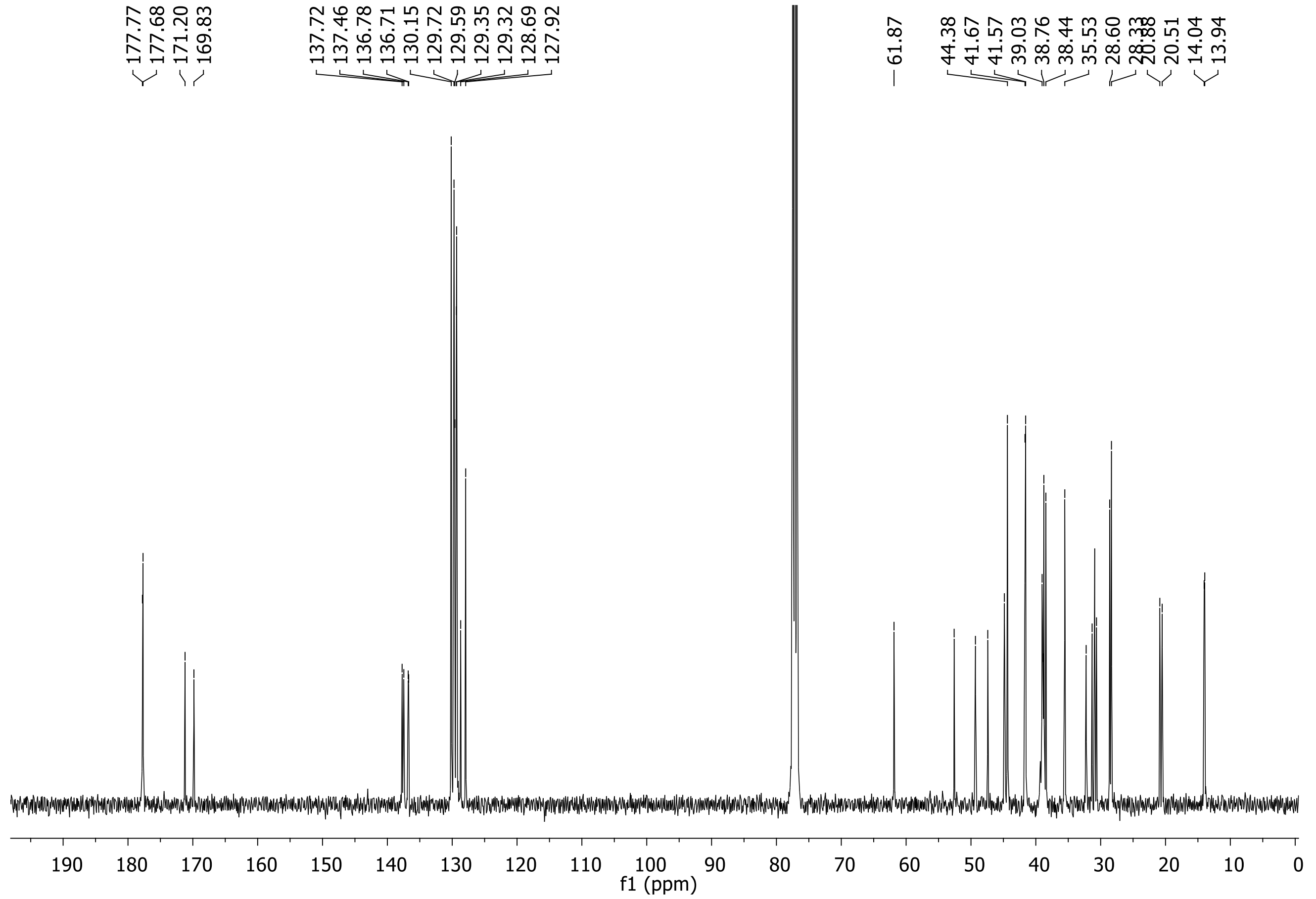

***trans*-2a-*d*$_2$** (^{1}H NMR, 400 MHz, $CDCl_3$, 298K)

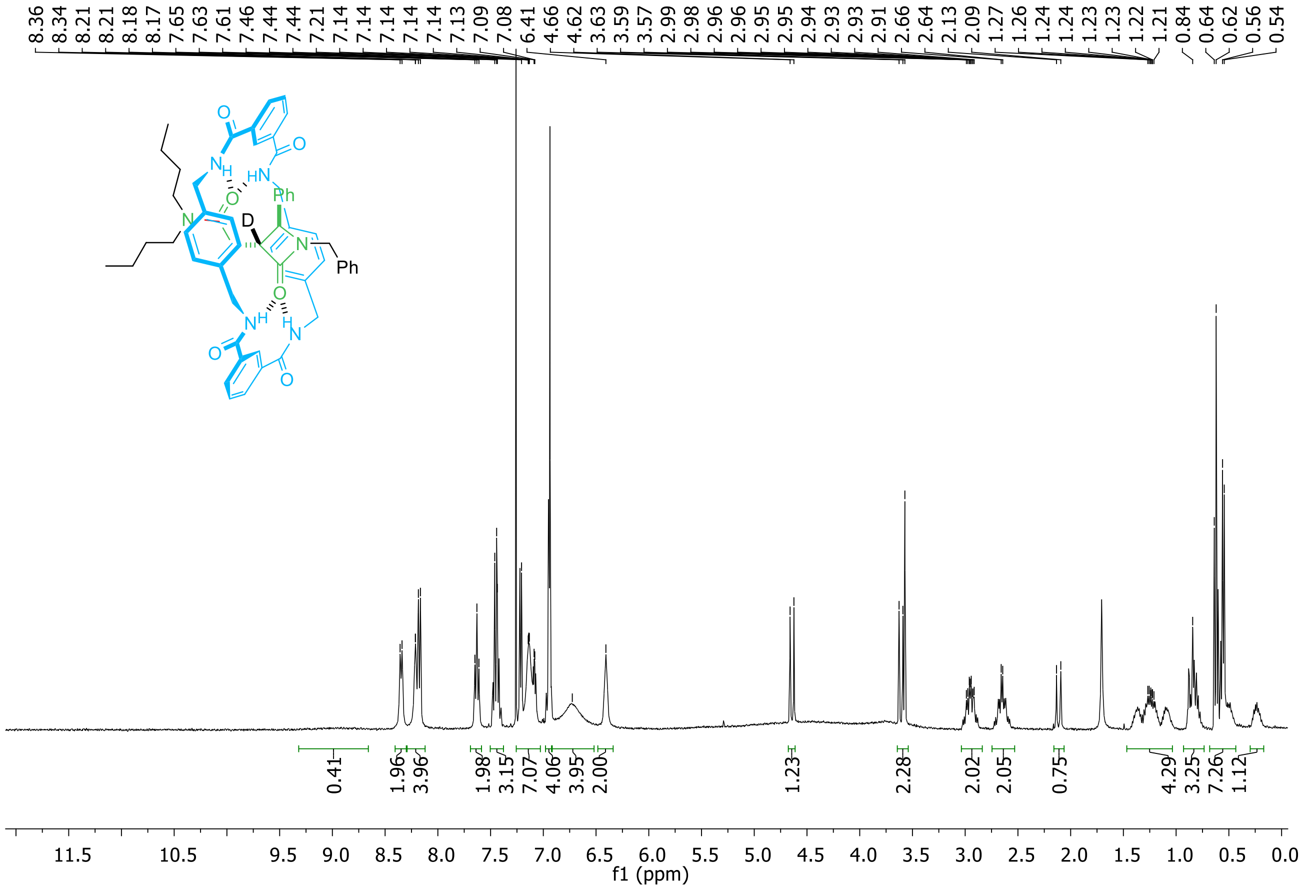

***trans*-2a-*d*$_2$** (^{13}C NMR, 75 MHz, $CDCl_3$, 298K)

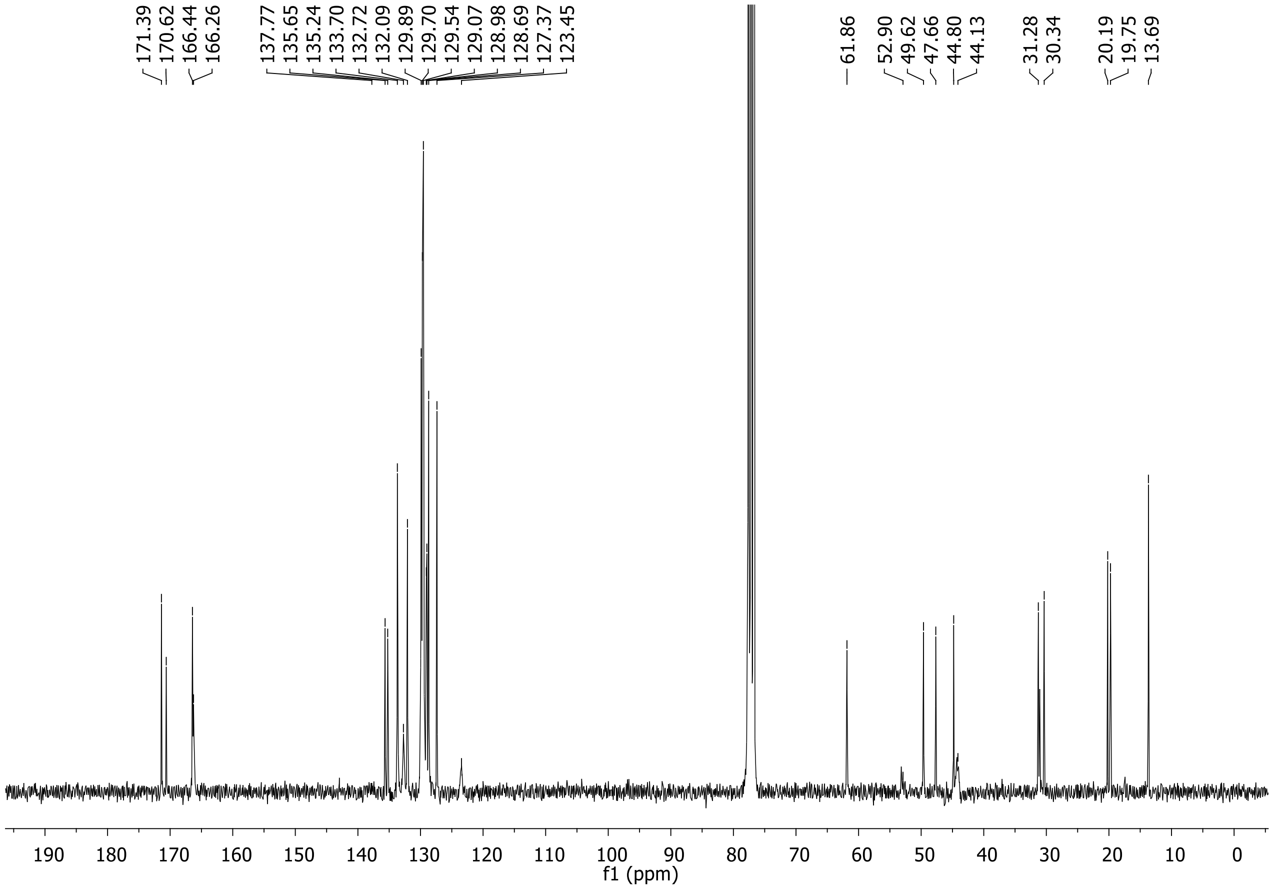

***trans*-5-*d*₁** (^{1}H NMR, 400 MHz, CDCl$_3$, 298K)

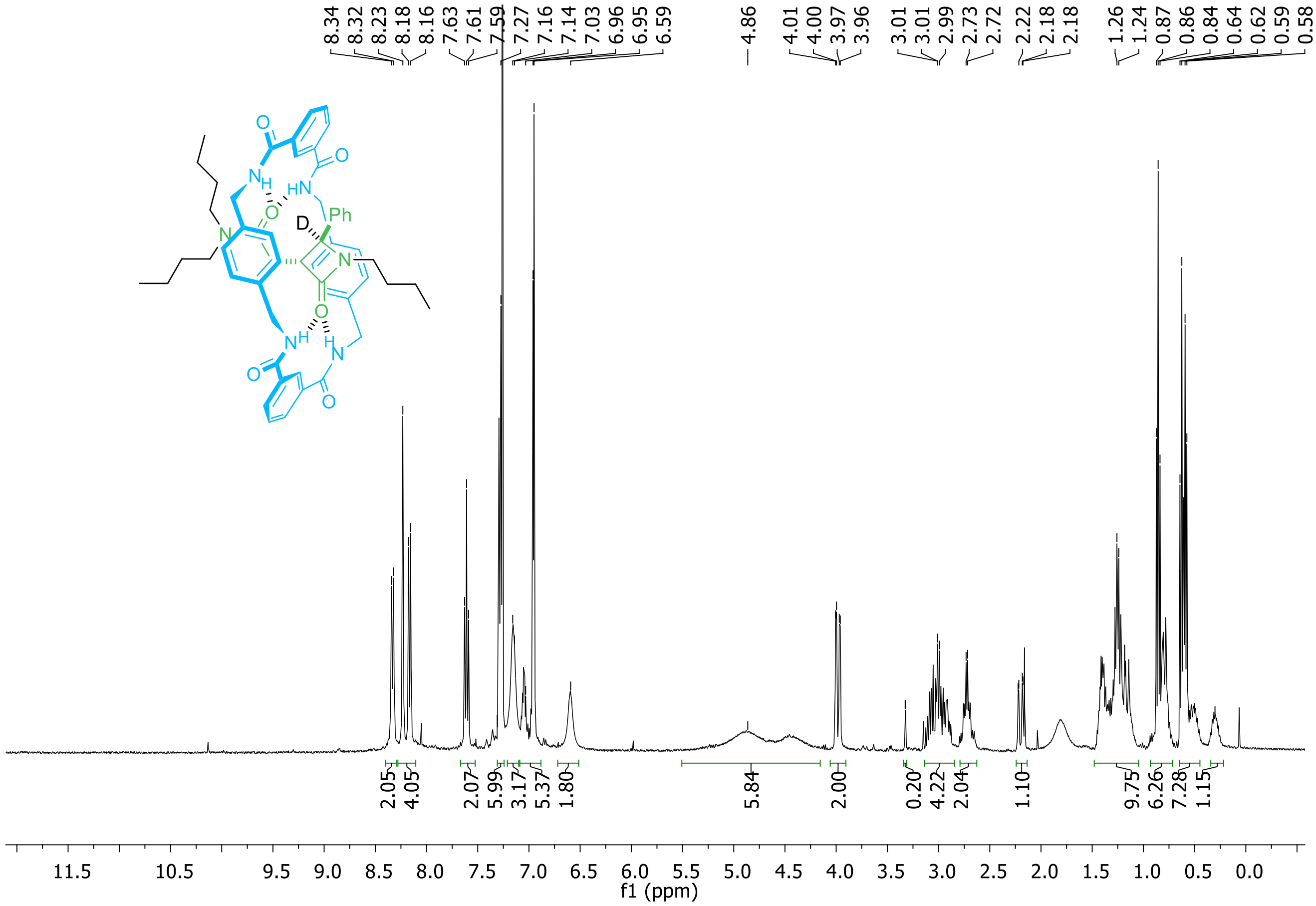

***trans*-5-*d*₁** (^{13}C NMR, 100 MHz, CDCl$_3$, 298K)

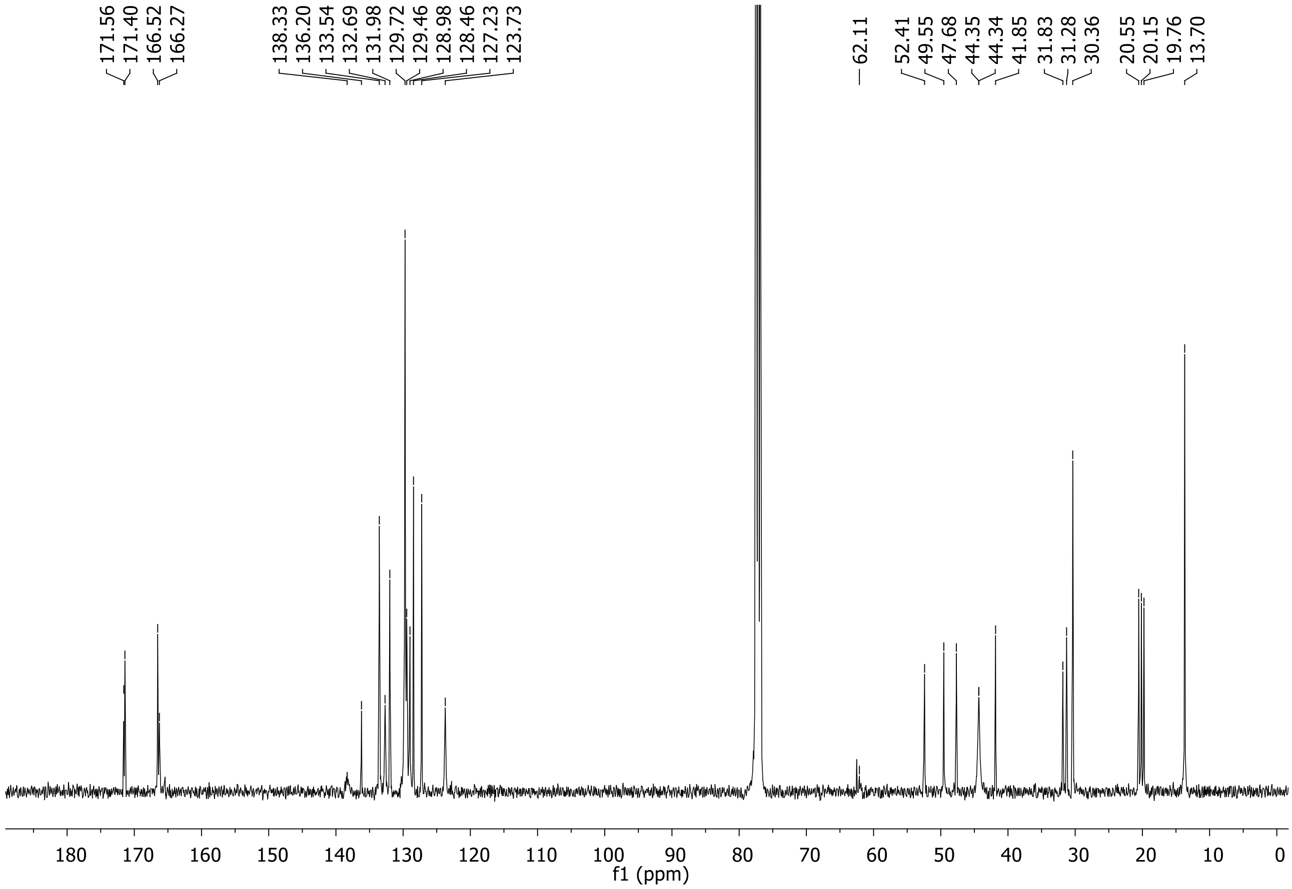

***trans*-8i** (^{1}H NMR, 400 MHz, $CDCl_3$, 298K)

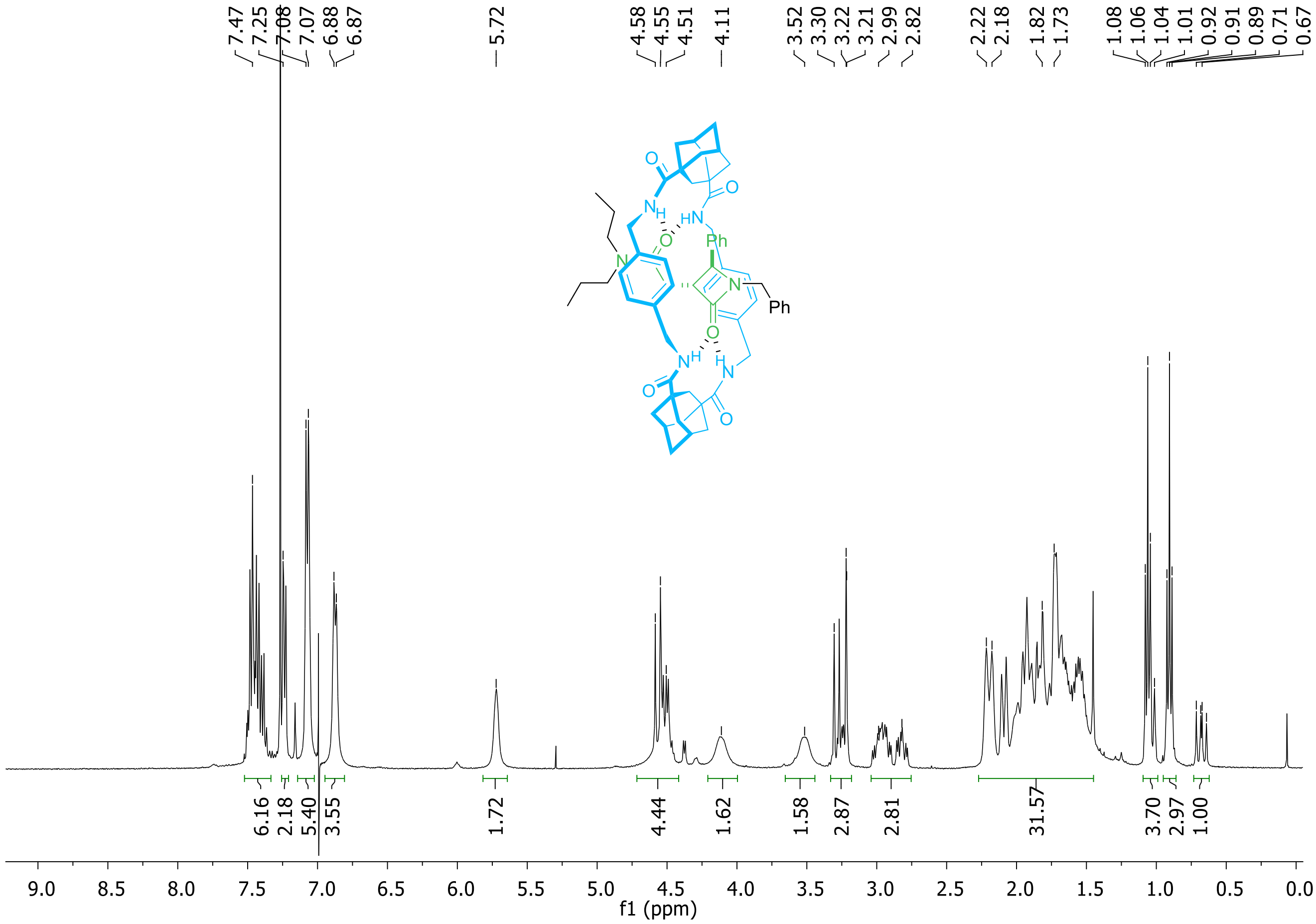

***trans*-8i** (^{13}C NMR, 75 MHz, $CDCl_3$, 318K)

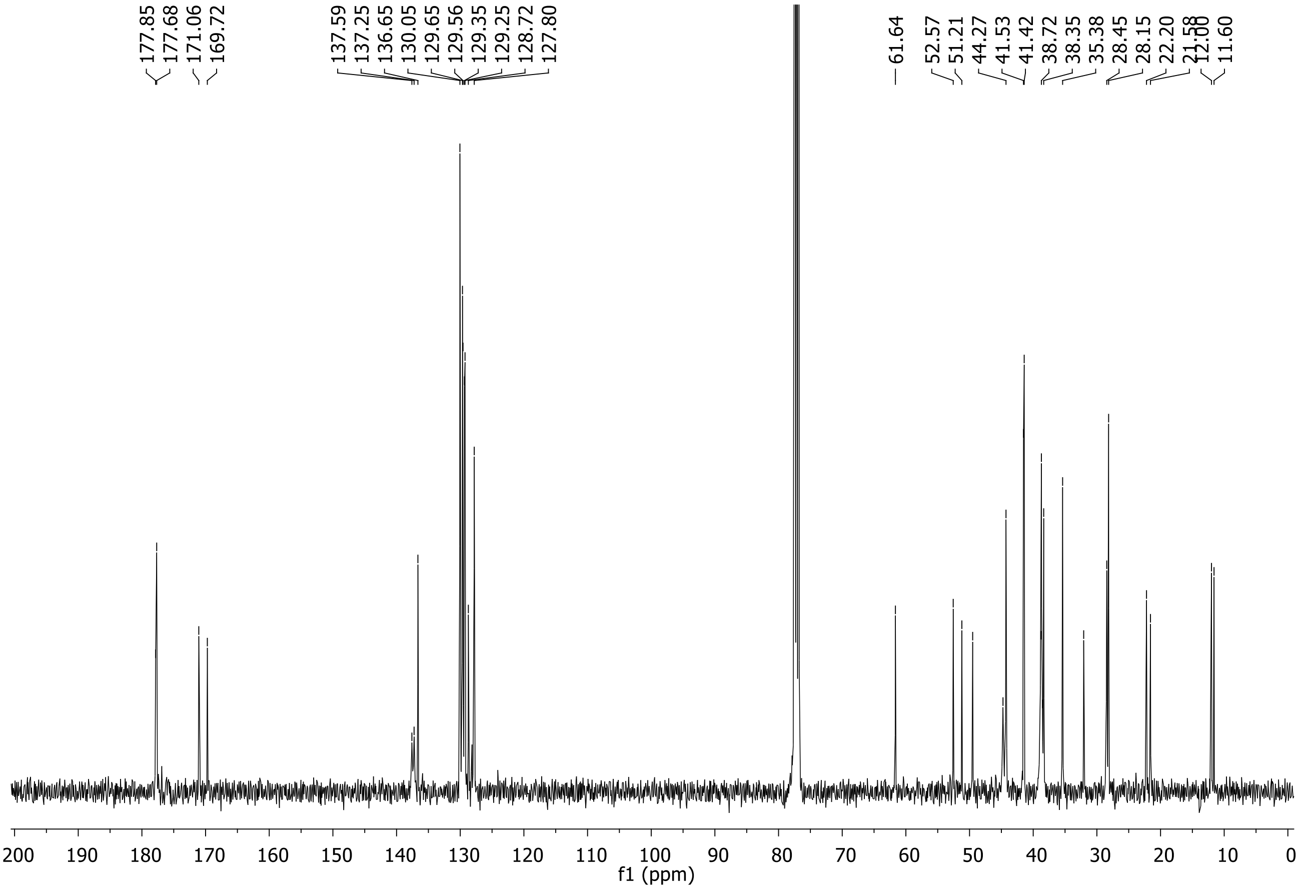

***trans*-9** (^{1}H NMR, 400 MHz, $CDCl_3$, 298K)

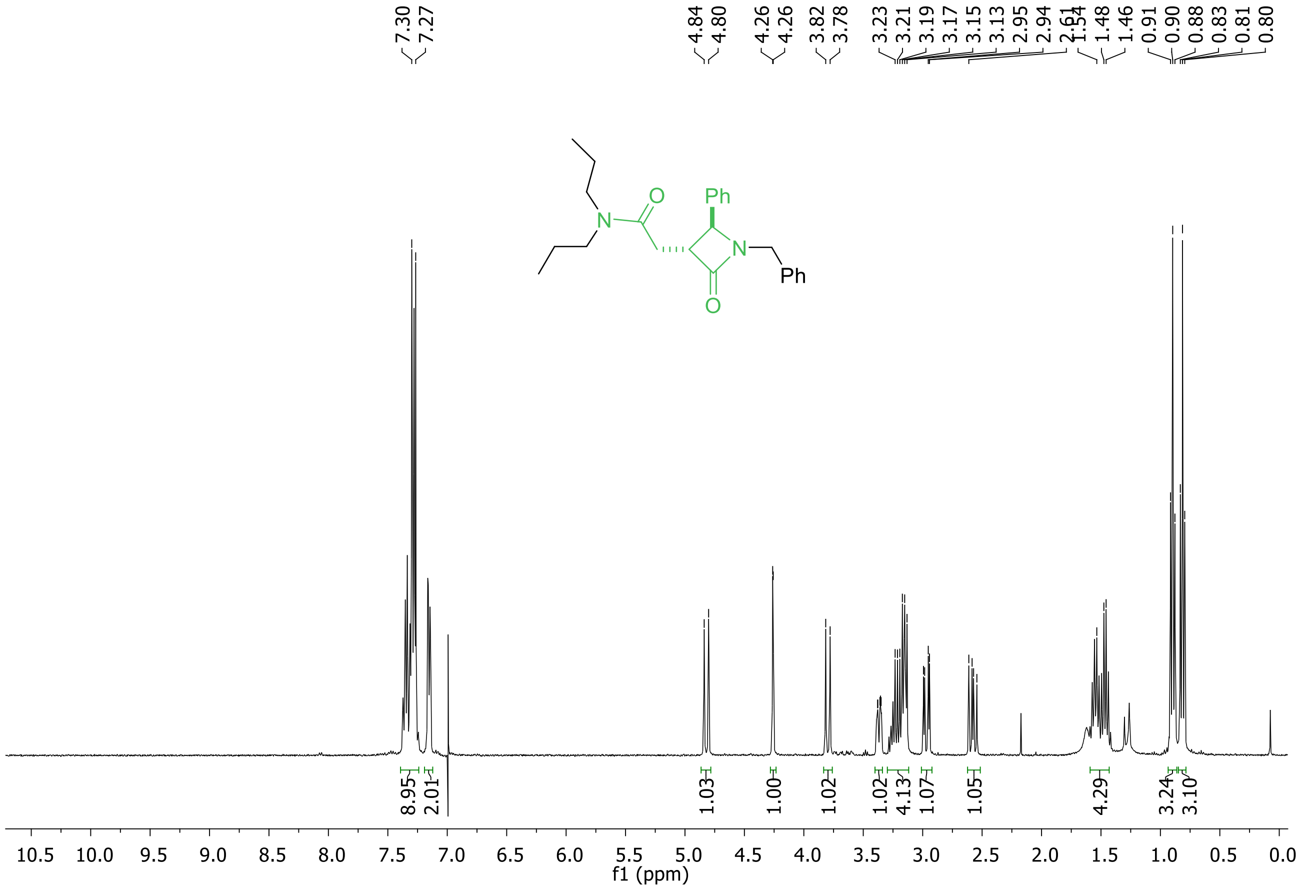

***trans*-9** (^{13}C NMR, 101 MHz, $CDCl_3$, 298K)

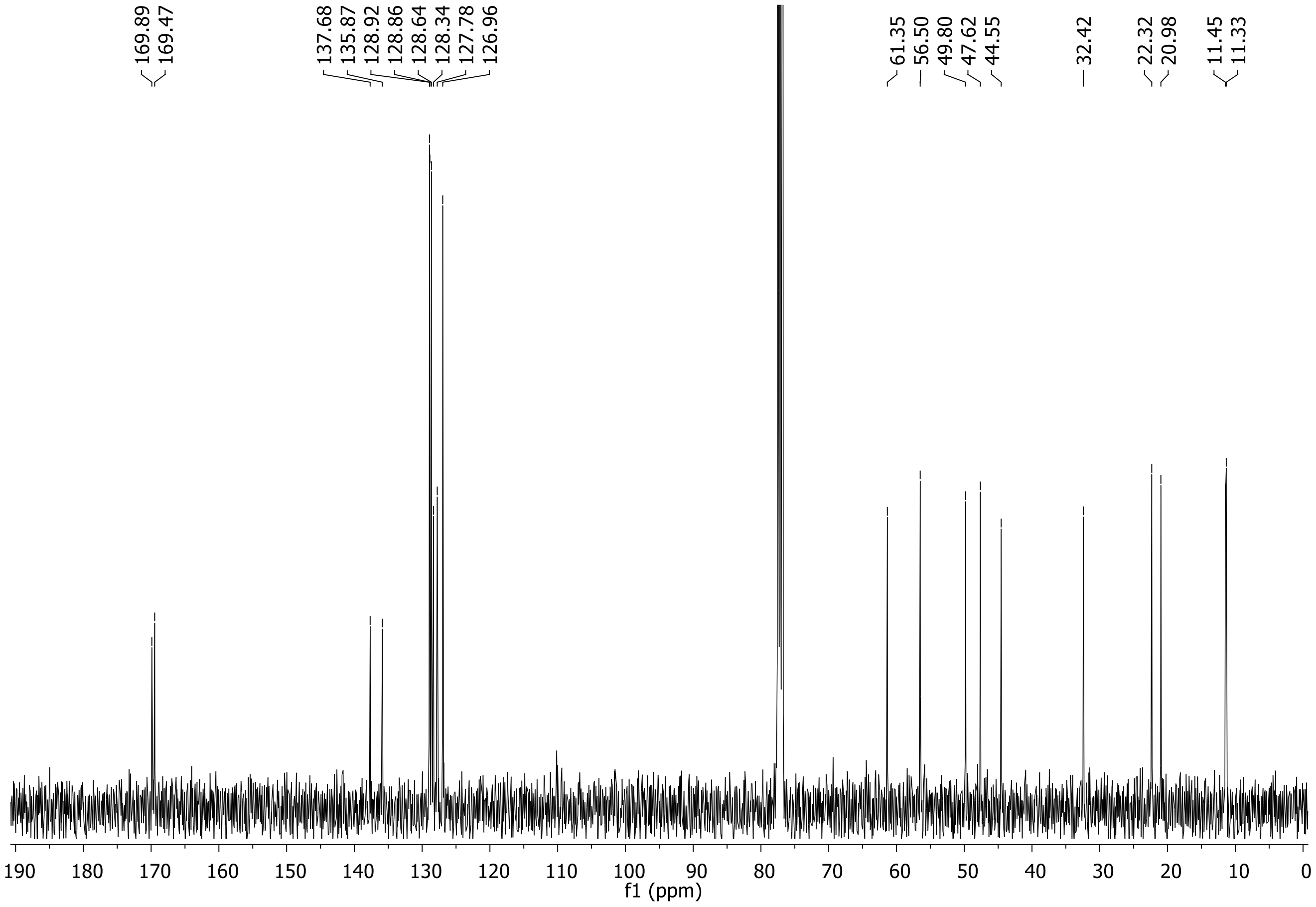

Mixture ***trans*-3** and ***cis*-3** (^{1}H NMR, 400 MHz, $CDCl_3$, 298K)

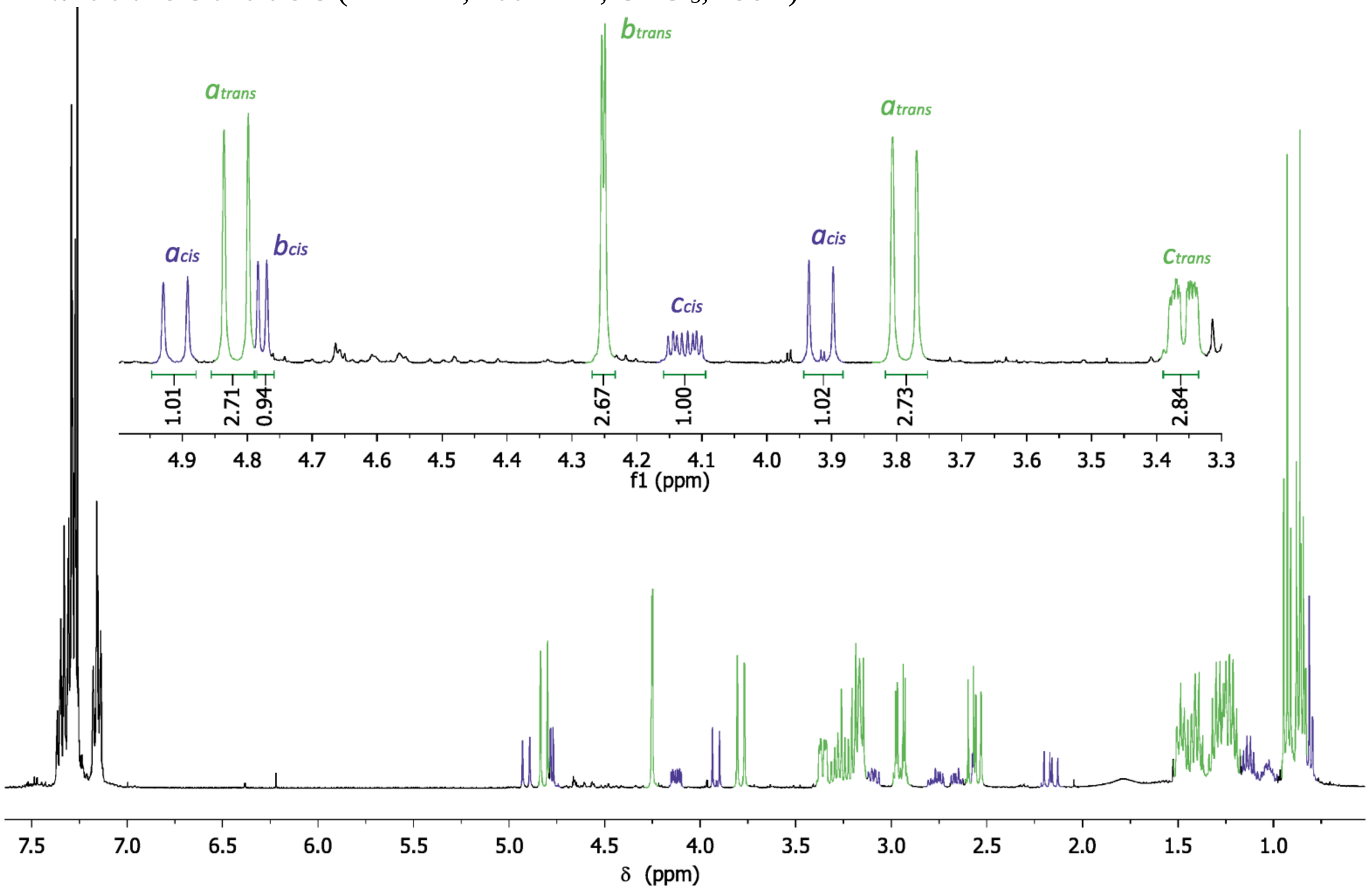

Mixture ***trans*-9** and ***cis*-9** (^{1}H NMR, 400 MHz, $CDCl_3$, 298K)

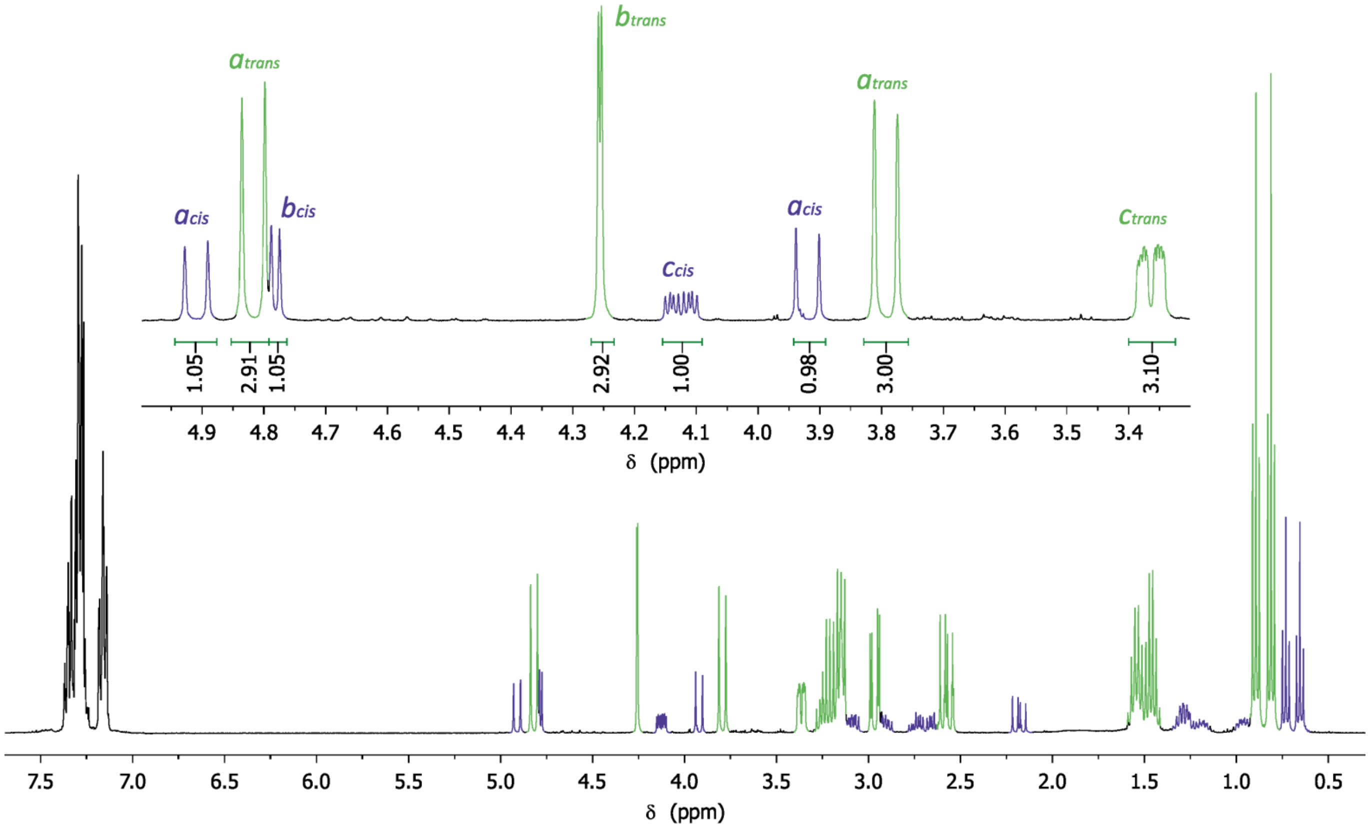

16. Computational methods

The geometries of the molecules were optimized by using the wB97X-D[5] hybrid-functional with the def2-SVP basis sets.[6] The nature of minimum and transition structures of all stationary points on the potential energy surface was confirmed by frequency analysis at the same level of theory. The stability of the resulting wave functions were checked for all the optimized structures.[7] The computed thermochemical corrections at wB97X-D/def2-SVP level were combined with single point energy calculations at the SMD(DMF)/DLPNO-CCSD(T)[8] /ma-def2-SVP[9]//wB97X-D/def2-SVP level to yield Gibbs free energies *G* at 298.15 K ($G_{298,sol}$). Solvent effects were calculated with the SMD solvation model with *N,N*-dimethylformamide parameters.[10] The RIJCOSX approximation was applied. AutoAux keyword was used. To avoid numerical noise due to the RIJCOSX approximation the radial and the angular grids were combined as IntaccX 4.01,4.01,4.34 and GridX 1,1,2, as the developers of the ORCA program recommend. The conformational analysis of the monoamidates **6** was performed at the SMD(DMF)/wB97x-D/6-31+G(d,p) theoretical level The ultrafine grid implemented in Gaussian 09 E. 01 was used.[11] Single point calculations were performed by using the program ORCA 4.2.1.[12]

17. Alternative computed mechanism for the cyclization of the interlocked fumaramides 1

Two alternative pathways were computationally scrutinized and are depicted in Figure S6. For clarity, the notation of the structures is different than that of the main text.

Following the pathway A, **INT0** evolves through an intramolecular proton transfer, from the thread to the macrocycle ($\Delta G_{TS1\text{-}A}$= +75.3 kJ mol^{-1}) to give the resonance-stabilized carbanion **INT1-A** (+37.8 kJ mol^{-1}). The intramolecular Michael addition of the *N*-benzyl carbanion to the fumaramide leads to the enolates *cis*- and *trans*-**INT2-A**, +87.0 and +72.9 kJ mol^{-1} respectively. Both stereoisomeric enolates **INT2-A** are subsequently protonated from one of the amide functions of the macrocycle to give *cis*- and *trans*-**INT3**, depending on the approaching trajectory followed. Besides, the energy profile displayed by path **A** agrees with the observed diastereoselectivity since the transition state *trans*-**TS2-A** is 14.1 kJ mol^{-1} lower in energy compared to *cis*-**TS2-A** (which means >99:1 d.r.).

Pathway **B** comprises our former mechanistic proposal.[1] Thus, the amidate **INT0** adds to the electrophilic carbon atom of the fumaramide via an intramolecular aza-Michael reaction (IMAMR,

$\Delta G_{TS1\text{-}B}$= +104.8 kJ mol^{-1}) leading to **INT1-B** (+39.5 kJ mol^{-1}). In the intermediate **INT1-B** the macrocycle and the thread are convalently bonded. **INT1-B** is an enolate able to abstract one of the benzylic protons from the dibenzylamido moiety to give **INT2-B** (+115.1 kJ mol^{-1}). The resultant carbanion **INT2-B**, evolves through an energetic 4-*exo-tet* ring closure, displacing the anchimeric assistant macrocycle and forming the new azetidinone ring at *cis*- and *trans*-**INT3**.

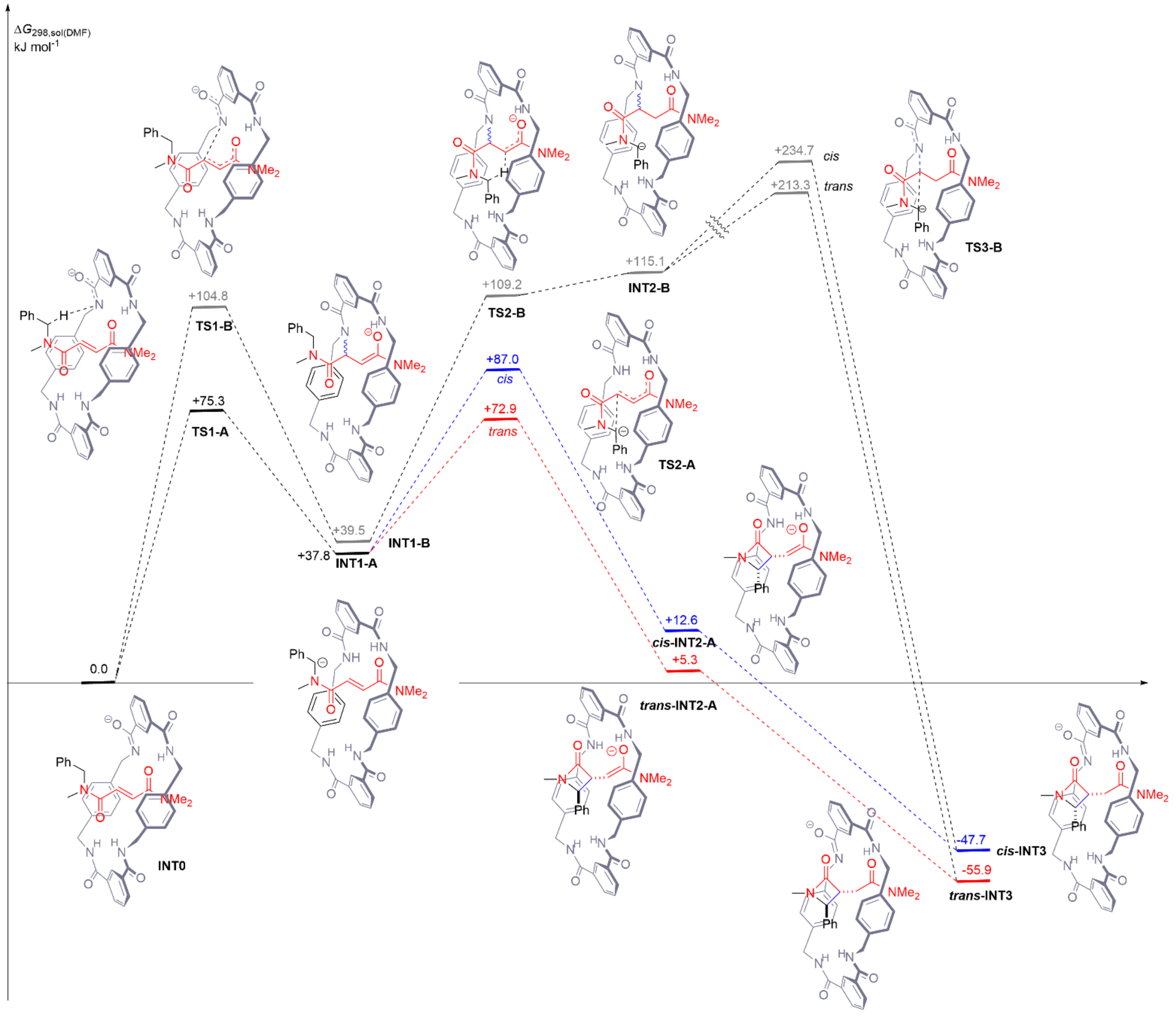

Figure S6. Proposed mechanism for the formation of the β-lactams **cis-INT3** and **trans-INT3** from the amidate **INT0** at the SMD(DMF)/DLPNO-CCSD(T)/ma-def2-SVP//wB97X-D/def2-SVP theoretical level. Gibbs free energies are reported in kJ mol^{-1} (1 atm and 298 K), relative to **INT0.** Path colors refer to the alternative trajectories for the 4-*exo-trig* cyclization step: *trans* (red) and *cis* (blue).

18. Computed mechanism for the cyclization of *N-benzyl-N,N',N'*-trimethylfumaramide

We have computed the mechanism of the cyclization of the amidate **Th** towards the β-lactams *cis*-**Lac** and *trans*-**Lac** (Figure S7)**.** A conformational analysis on **Th** showed that *cis*-**Th**, which is the conformer of lowest energy and is pre-oriented for cyclizing towards *cis*-**Lac**, is 12.2 kJ mol^{-1} lower in energy respect to the pre-oriented *trans*-**Th**. Both conformers convert each other involving the transition structure **TSrot** (+10.9 kJ mol^{-1}). On the other hand, two transition structures for the 4-*exo-trig* ring closure cyclization towards the β-lactams *cis*-**Lac** and *trans*-**Lac** were located. Our calculations predicted that the energy barrier for the cyclization of *cis*-**Th** is 17.3 kJ mol^{-1} (**TS*cis***) whereas that of *trans*-**Th** is 15.6 kJ mol^{-1} (**TS*trans***), which means 33:67 d.r. Moreover, the β-lactams *trans*-**Lac** was computed as the most stable thermodynamically (-42.5 kJ mol^{-1}) compared to the alternative *cis*-**Lac** (-34.5 kJ mol^{-1}). These calculations are in line with those experimentally observed in which a mixture of the *cis*- and *trans*- β-lactams is obtained.

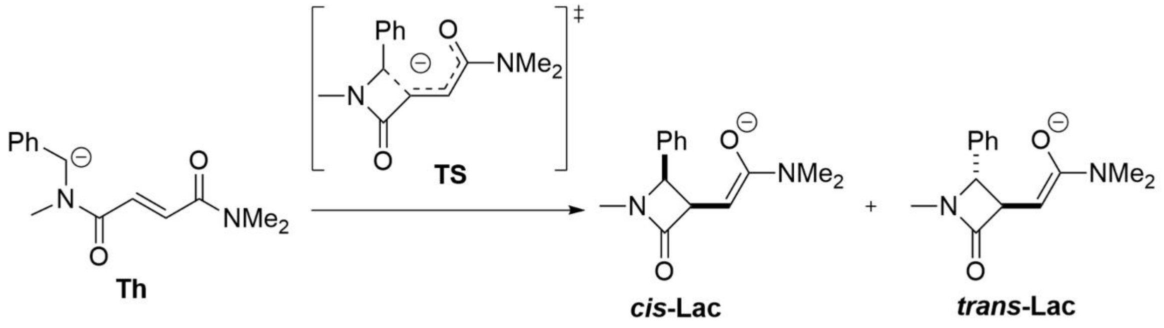

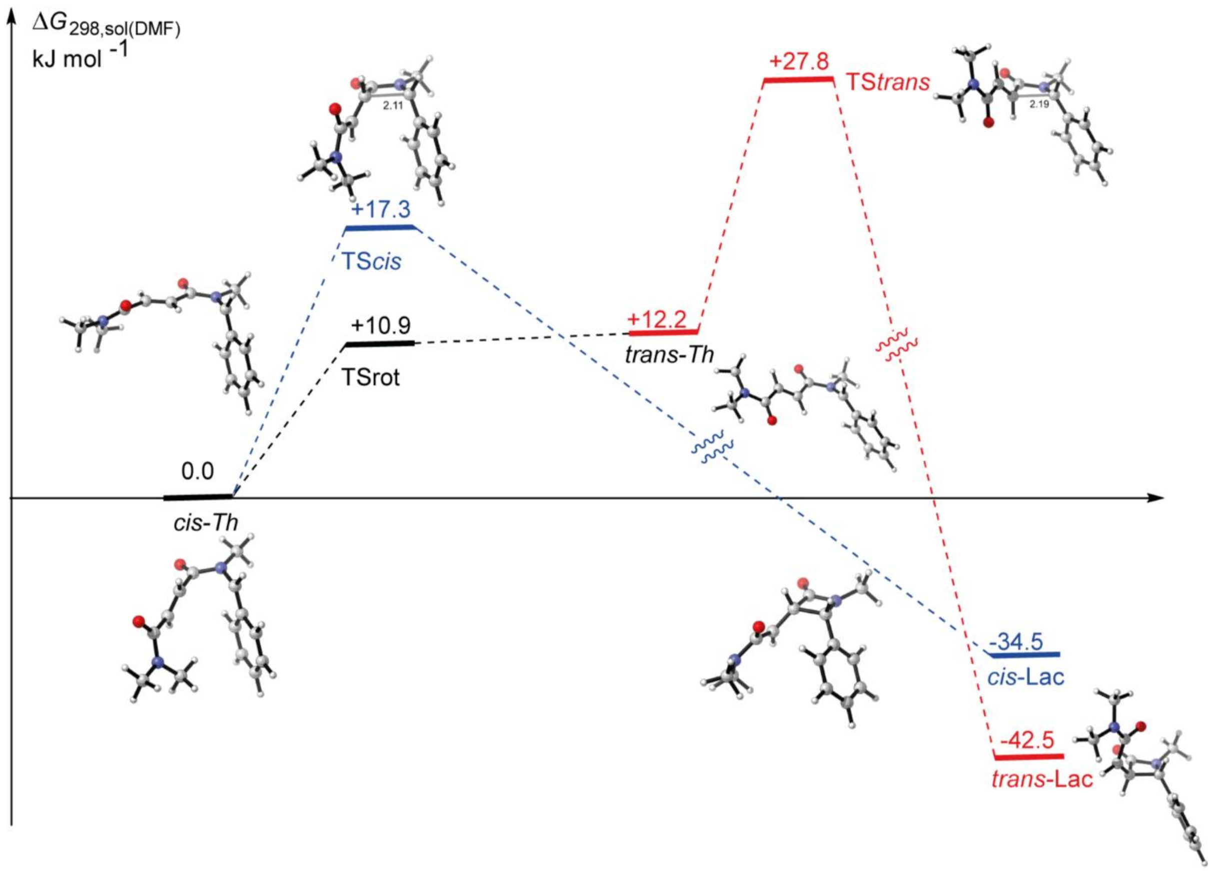

Figure S7. Proposed mechanism for the formation of the β-lactams ***cis*-Lac** and ***trans*-Lac** from the amidate **Th** at the SMD(DMF)/DLPNO-CCSD(T)/ma-def2-SVP//wB97X-D/def2-SVP theoretical level. Gibbs free energies are reported in kJ mol^{-1} (1 atm and 298 K), relative to ***cis*-Th.** Path colors refer to the alternative trajectories for the 4-*exo-trig* cyclization step: *trans* (red) and *cis* (blue).

19. Conformational analysis of the monoamidates 6 and their corresponding *N*,*N*'-dimethylisophthalamides 6’

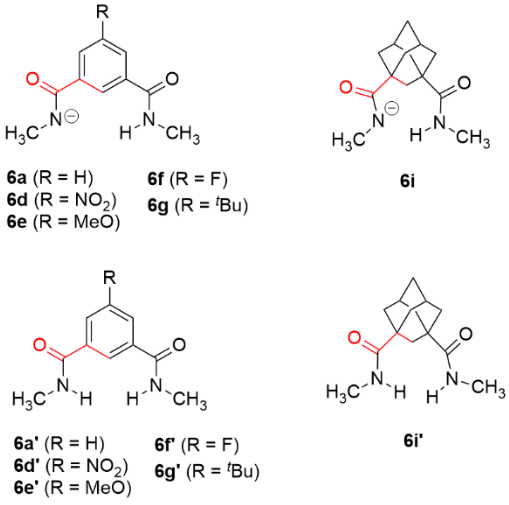

Figure S8. Monoamidates **6** and the corresponding *N*,*N*'-dimethylisophthalamides **6’**.

The rotational energy barrier along the NCO-Ar bond of the monoamidates **6** and the *N*,*N*'-dimethylisophthalamides **6’** was estimated at DFT level. The dihedral angle formed by the plane containing the phenyl ring and the plane containing the amide moiety was scanned with intervals of five degrees (bonds highlighted in red, Figure S8). Rotational energy barriers were computed as the energy difference between the points of highest and lowest energy along the scan profile (see Figures S8-S11). Note that the notation *in* (inside) and *out* (outside) refers to the orientation of the carbonyl groups, for instance the monoamidates shown in Figure S8 present a disposition *out/out*.

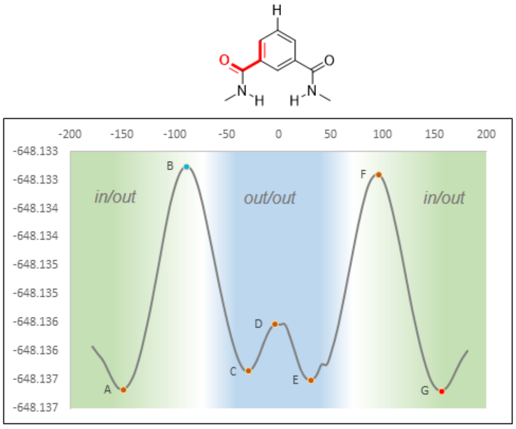

	Dihedral	E_{SCF}	ΔE (kJ/mol)
A	-148.7	-648.136667	0.1
B	-88.7	-648.132757	10.3
C	-28.7	-648.136349	0.9
D	-3.7	-648.135521	3.1
E	31.3	-648.136501	0.5
F	96.3	-648.132898	10.0
G	156.3	-648.13669	0.0

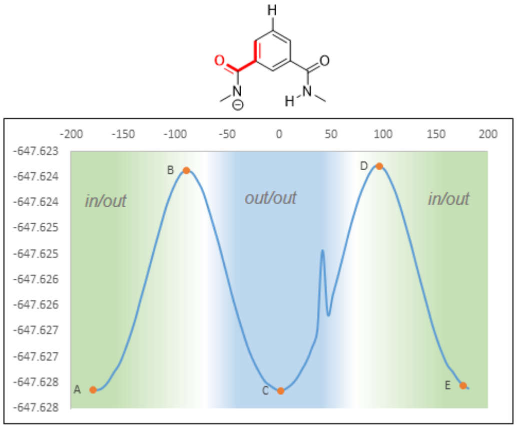

	Dihedral	E_{SCF}	ΔE (kJ/mol)
A	-178.7	-647.627642	0.0
B	-88.7	-647.623374	11.3
C	1.3	-647.62766	0.0
D	96.3	-647.623285	11.2
E	176.3	-647.627547	0.0

Figure S9. Rotational energy barriers for the *N*,*N*'-dimethylisophthalamide **6a'** and the monoamidate **6a**.

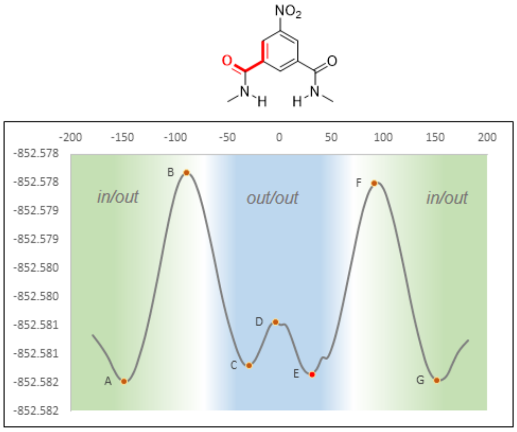

	Dihedral	E_{SCF}	ΔE (kJ/mol)
A	-148.7	-852.5814841	0.0
B	-88.7	-852.5778280	9.6
C	-28.7	-852.5811986	0.7
D	-3.7	-852.5804376	2.7
E	31.3	-852.5813489	0.4
F	91.3	-852.5780096	9.1
G	151.3	-852.5814566	0.1

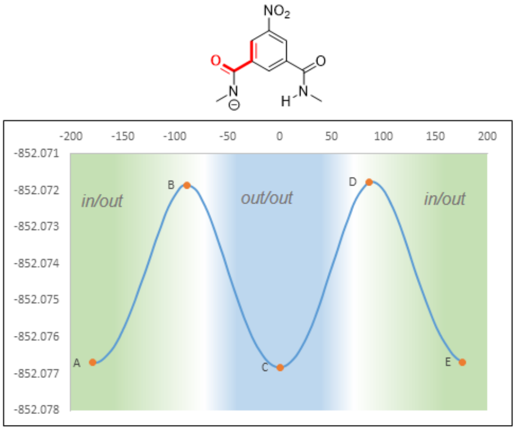

	Dihedral	E_{SCF}	ΔE (kJ/mol)
A	-178.7	-852.076692	0.3
B	-88.7	-852.071841	13.1
C	1.3	-852.076813	0.0
D	86.3	-852.071766	13.3
E	176.3	-852.076673	0.4

Figure S10. Rotational energy barriers for the *N*,*N*'-dimethylisophthalamide **6d'** and the monoamidate **6d**.

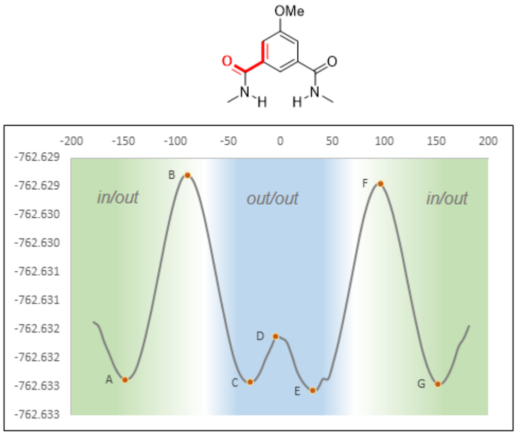

	Dihedral	E_{SCF}	ΔE (kJ/mol)
A	-148.7	-762.632376	0.5
B	-88.7	-762.628795	9.9
C	-28.7	-762.632429	0.4
D	-3.7	-762.631626	2.5
E	31.3	-762.632576	0.0
F	96.3	-762.628951	9.5
G	151.3	-762.63246	0.3

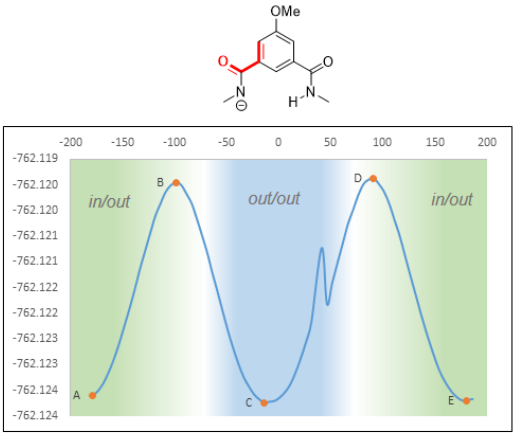

	Dihedral	E_{SCF}	ΔE (kJ/mol)
A	-178.7	-762.123607	0.3
B	-98.7	-762.119453	11.3
C	-13.7	-762.123738	0.0
D	91.3	-762.11937	11.5
E	180.0	-762.123699	0.1

Figure S11. Rotational energy barriers for the *N*,*N'*-dimethylisophthalamide **6e'** and the monoamidate **6e**.

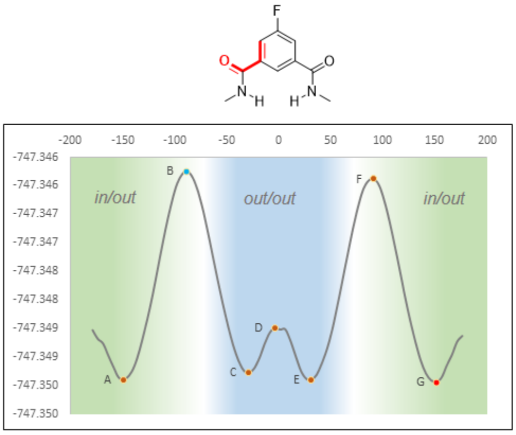

	Dihedral	E_{SCF}	ΔE (kJ/mol)
A	-148.7	-747.34939	0.1
B	-88.7	-747.345741	9.7
C	-28.7	-747.349272	0.4
D	-3.7	-747.348485	2.5
E	31.3	-747.349394	0.1
F	91.3	-747.345878	9.3
G	151.3	-747.349429	0.0

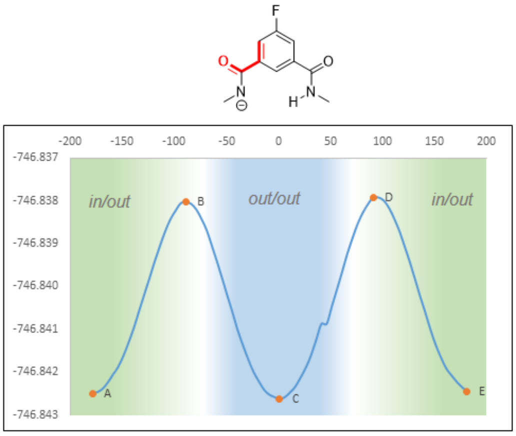

	Dihedral	E_{SCF}	ΔE (kJ/mol)
A	-178.7097	-746.842493	0.3
B	-88.7097	-746.838027	12.1
C	1.2903	-746.842624	0.0
D	91.2902	-746.837919	11.9
E	181	-746.842444	0.0

Figure S12. Rotational energy barriers for the *N*,*N'*-dimethylisophthalamide **6f'** and the monoamidate **6f**.

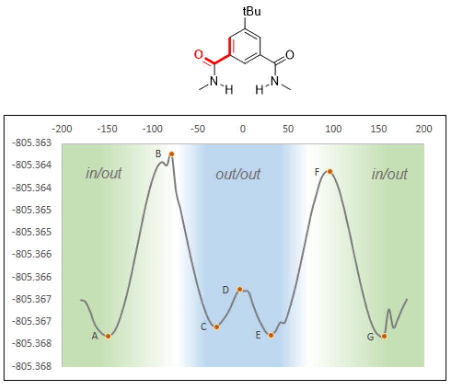

	Dihedral	E_{SCF}	ΔE (kJ/mol)
A	-148.7	-805.367318	0.0
B	-78.7	-805.363224	10.8
C	-28.7	-805.367101	0.6
D	-3.7	-805.366275	2.8
E	31.3	-805.36729	0.1
F	96.3	-805.363632	9.7
G	156.3	-805.367325	0.0

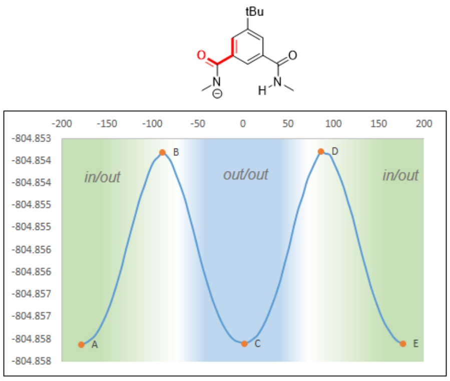

	Dihedral	E_{SCF}	ΔE (kJ/mol)
A	-178.7	-804.857615	0.0
B	-88.7	-804.853313	11.3
C	1.3	-804.85759	0.1
D	86.3	-804.853292	11.4
E	176.3	-804.857595	0.1

Figure S13. Rotational energy barriers for the *N*,*N*'-dimethylisophthalamide **6g'** and the monoamidate **6g**.

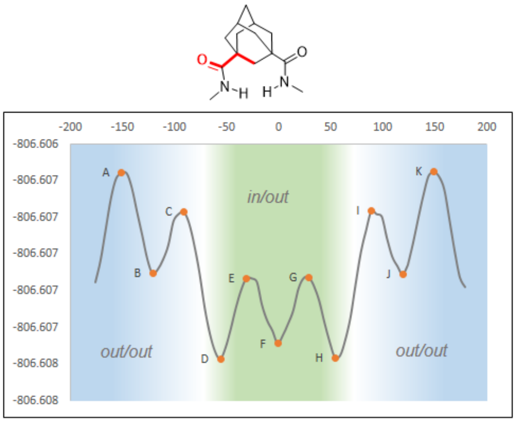

	Dihedral	E_{SCF}	ΔE (kJ/mol)
A	-150.7	-806.6065557	2.7
B	-120.7	-806.6071058	1.2
C	-90.7	-806.6067687	2.1
D	-55.7	-806.6075728	0.0
E	-30.7	-806.6071313	1.2
F	-0.7	-806.6074876	0.2
G	29.3	-806.6071263	1.2
H	54.3	-806.6075646	0.0
I	89.3	-806.6067622	2.1
J	119.3	-806.6071100	1.2
K	149.3	-806.6065502	2.7

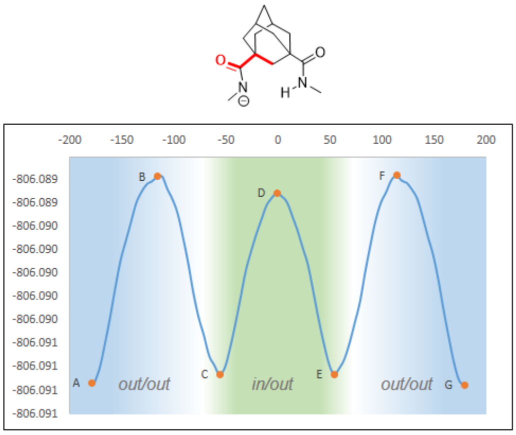

	Dihedral	E_{SCF}	ΔE (kJ/mol)
A	-178.7	-806.090934	0.1
B	-115.7	-806.08917	4.7
C	-55.7	-806.090862	0.2
D	-0.7	-806.089312	4.3
E	54.3	-806.090861	0.3
F	114.3	-806.089161	4.7
G	179.3	-806.090957	0.0

Figure S14. Rotational energy barriers for the *N*,*N*'-dimethylisophthalamide **6i'** and the monoamidate **6i**.

20. Computational data

Table S5. Imaginary frequencies, electronic ($E_{SCF,298,sol}$), Gibbs free ($G_{298,sol}$), and enthalpy ($H_{298,sol}$) energies for systems shown in Figures S6 and S7 (in Hartree) computed at SMD(DMF)/DLPNO-CCSD(T)/ma-def2-SVP2//wB97X-D/def2-SVP level. The wB97X-D/def2-SVP level of theory was used to optimize the geometries and calculate the thermal corrections.

Filename	Freqs	E_{tot} wB97X-D/def2-SVP	G_{298} wB97X-D/def2-SVP	H_{298} wB97X-D/def2-SVP	$E_{tot,sol}$ SMD/DLPNO-CCSD(T)/ /ma-def2-SVP2// wB97X-D/def2-SVP	$G_{298,sol}$ SMD/ DLPNO-CCSD(T)/ /ma-def2-SVP2// wB97X-D/def2-SVP
**INT0**						
rtx_an_01	-	-2557.427249	-2556.670176	-2556.526344	-2551.279298	-2550.522225
rtx_an_02	-	-2557.42637	-2556.663314	-2556.524658	-2551.285226	-2550.52217
rtx_an_03	-	-2557.428501	-2556.667538	-2556.527663	-2551.277149	-2550.516186
**TS1-A**						
ts1_rtx_anp_04	-1316.9	-2557.389669	-2556.634747	-2556.49565	-2551.248473	-2550.493551
ts1_rtx_anp_02	-924.4	-2557.383645	-2556.628307	-2556.489018	-2551.237144	-2550.481806
**INT1-A**						
rtx_anp_3	-	-2557.42261	-2556.666089	-2556.523723	-2551.264358	-2550.507837
rtx_anp_2	-	-2557.416989	-2556.656737	-2556.517418	-2551.264769	-2550.504517
rtx_anp_1	-	-2557.418507	-2556.660455	-2556.518997	-2551.257857	-2550.499805
rtx_anp_03	-	-2557.423119	-2556.662804	-2556.523365	-2551.258973	-2550.498658
rtx_anp_02	-	-2557.423119	-2556.662731	-2556.523345	-2551.258992	-2550.498604
rtx_anp_6	-	-2557.419873	-2556.659054	-2556.520257	-2551.258839	-2550.49802
rtx_anp_4	-	-2557.419873	-2556.658989	-2556.520247	-2551.258839	-2550.497955
rtx_anp_12	-	-2557.412636	-2556.650475	-2556.513115	-2551.253211	-2550.49105
rtx_anp_11	-	-2557.385993	-2556.627111	-2556.487056	-2551.245907	-2550.487025
***trans*-TS2-A**						
ts3t_rtx_anp_02	-157.6	-2557.420927	-2556.659807	-2556.522014	-2551.255569	-2550.494449

ts3t_rtx_anp_red	-219.8	-2557.41543	-2556.656552	-2556.516787	-2551.252517	-2550.493639
***cis*-TS2-A**						
ts3c_rtx_anp_01	-112.4	-2557.412466	-2556.649783	-2556.51397	-2551.251777	-2550.489094
ts3c_rtx_anp_07	-112.8	-2557.412466	-2556.649734	-2556.513965	-2551.251289	-2550.488557
***trans*-INT2-A**						
lactam_anp_01	-	-2557.441961	-2556.680198	-2556.541663	-2551.281983	-2550.52022
lactam_anp_02	-	-2557.44096	-2556.676884	-2556.540532	-2551.282761	-2550.518685
lactamtrans_anp04	-	-2557.429853	-2556.671104	-2556.53006	-2551.267632	-2550.508883
lactamtrans_anp03	-	-2557.427671	-2556.672067	-2556.52766	-2551.257599	-2550.501995
***cis*-INT2-A**						
lactam_cis_anp_01	-	-2557.439673	-2556.675482	-2556.539552	-2551.281598	-2550.517407
lactam_cis_anp_02	-	-2557.418346	-2556.658608	-2556.518669	-2551.249032	-2550.489294
***trans*-INT3**						
lactam_an_trans_01	-	-2557.459213	-2556.694231	-2556.557964	-2551.308494	-2550.543512
lactam_an_trans_04	-	-2557.43754	-2556.676208	-2556.537245	-2551.297664	-2550.536332
lactam_an_trans_02	-	-2557.436004	-2556.673378	-2556.535744	-2551.292854	-2550.530228
lactam_an_trans_03	-	-2557.425317	-2556.664507	-2556.525957	-2551.291153	-2550.530343
lactam_an_trans_07	-	-2557.414539	-2556.658895	-2556.515427	-2551.279216	-2550.523572
***cis*-INT3**						
lactam_an_cis_01	-	-2557.452916	-2556.686082	-2556.551454	-2551.307217	-2550.540383
lactam_an_cis_02	-	-2557.434229	-2556.671067	-2556.533986	-2551.294181	-2550.531019
lactam_an_cis_04	-	-2557.436388	-2556.672269	-2556.535772	-2551.293393	-2550.529274
**TS1-B**						
ts1_rtx_an_09	-255.9	-2557.397278	-2556.634274	-2556.497644	-2551.245324	-2550.48232
ts1_rtx_an_01	-242	-2557.395257	-2556.633128	-2556.496108	-2551.243377	-2550.481248

**INT1-B**						
rtx_an_int1_3	-	-2557.416331	-2556.650144	-2556.515499	-2551.273365	-2550.507178
rtx_an_int1_1	-	-2557.414194	-2556.648488	-2556.513062	-2551.267694	-2550.501988
rtx_an_int1_6	-	-2557.413461	-2556.647916	-2556.512965	-2551.261834	-2550.496289
rtx_an_int1_02	-	-2557.411106	-2556.645124	-2556.509903	-2551.256734	-2550.490752
**TS2-B**						
ts2_rtx_an_8	-1038.3	-2557.389477	-2556.627761	-2556.494094	-2551.242356	-2550.48064
ts2_rtx_an_1	-1038.4	-2557.389477	-2556.627697	-2556.494083	-2551.242192	-2550.480412
ts2_rtx_an_3	-1038.4	-2557.389477	-2556.627743	-2556.494091	-2551.242055	-2550.480321
ts2_rtx_an_01	-1207.8	-2557.386476	-2556.624169	-2556.491044	-2551.237579	-2550.475272
**INT2-B**						
rtx_an_int2_01	-	-2557.393937	-2556.628149	-2556.49408	-2551.244183	-2550.478395
rtx_an_int2_04	-	-2557.386174	-2556.622379	-2556.485021	-2551.235694	-2550.471899
rtx_an_int2_02	-	-2557.373717	-2556.606568	-2556.47336	-2551.232627	-2550.465478
rtx_an_int2_03	-	-2557.370901	-2556.603956	-2556.470685	-2551.231111	-2550.464166
***trans*-TS3-B**						
ts3t_rtx_an_02r	-522.2	-2557.325548	-2556.562342	-2556.428111	-2551.204183	-2550.440977
ts3t_rtx_an_04	-686.5	-2557.32925	-2556.568016	-2556.431943	-2551.199853	-2550.438619
ts3t_rtx_an_07	-687.3	-2557.33104	-2556.572821	-2556.43395	-2551.191667	-2550.433448
ts3t_rtx_an_09	-698.4	-2557.325602	-2556.567672	-2556.428235	-2551.190219	-2550.432289
***cis*-TS3-B**						
ts3c_rtx_an_03	-705.0	-2557.331457	-2556.573043	-2556.434305	-2551.19126	-2550.432846
ts3c_rtx_an_04	-705.0	-2557.331457	-2556.573065	-2556.434311	-2551.190987	-2550.432595
**Thread**						
eje13_c	-	-802.8520592	-802.6115640	-802.5451210	-800.963406	-800.7229103

eje_09	-	-802.8537784	-802.6130240	-802.5466020	-800.962356	-800.7216020
eje_00	-	-802.8537783	-802.6130280	-802.5466040	-800.962069	-800.7213192
eje_06	-	-802.8510821	-802.6111920	-802.5439900	-800.958825	-800.7189348
eje_02	-	-802.8473446	-802.6087270	-802.5402170	-800.956878	-800.7182608
eje_07	-	-802.8527011	-802.6128390	-802.5456740	-800.95758	-800.7177179
eje_04	-	-802.8527012	-802.6128370	-802.5456740	-800.957572	-800.7177083
eje_11	-	-802.8445933	-802.6047510	-802.5376800	-800.955796	-800.7159536
eje_05	-	-802.8481904	-802.6096010	-802.5412230	-800.953083	-800.7144931
eje_10	-	-802.8300128	-802.5904560	-802.5228780	-800.947389	-800.7078317
eje_08	-	-802.8339187	-802.5942120	-802.5264080	-800.947339	-800.7076324
eje_12	-	-802.8294367	-802.5897370	-802.5223440	-800.947289	-800.7075889
**TS*cis***						
ts_c01	-276.7	-802.8475120	-802.6072300	-802.5418380	-800.9566101	-800.7163281
tsc_02	-289.0	-802.8437150	-802.6030150	-802.5379380	-800.9564817	-800.7157817
**TS*trans***						
tst_01	-172.5	-802.8432214	-802.6037040	-802.5374700	-800.9518274	-800.7123100
tst_02	-274.5	-802.8400413	-802.5999660	-802.5341700	-800.9478352	-800.7077599
**TSrot**						
ts_rot	-43.6	-802.8442166	-802.6057350	-802.5384050	-800.957251	-800.7187697
***cis*-Lac**						
lcis_01	-	-802.8621008	-802.6189390	-802.5543840	-800.979214	-800.7360519
lcis_02	-	-802.8601538	-802.6164060	-802.5522870	-800.976851	-800.7331030
lcis_03	-	-802.8601539	-802.6164150	-802.5522910	-800.976815	-800.7330762
lcis_04	-	-802.8601539	-802.6164170	-802.5522910	-800.97681	-800.7330734
lcis_10	-	-802.8504756	-802.6090730	-802.5432120	-800.974321	-800.7329188
lcis_11	-	-802.8504756	-802.6090750	-802.5432120	-800.974281	-800.7328805
lcis_08	-	-802.8504756	-802.6090680	-802.5432100	-800.974283	-800.7328750

lcis_05	-	-802.8491279	-802.6070360	-802.5415370	-800.973224	-800.7311317
lcis_07	-	-802.8498072	-802.6071210	-802.5424500	-800.972247	-800.7295605
lcis_09	-	-802.8484245	-802.6042750	-802.5408760	-800.972799	-800.7286496
lcis_06	-	-802.8394975	-802.5984410	-802.5319990	-800.959278	-800.7182220
***trans*-Lac**						
ltrans_01	-	-802.8639997	-802.6216310	-802.5561680	-800.981414	-800.7390456
ltrans_03	-	-802.8642496	-802.6220300	-802.5565690	-800.977918	-800.7356988
ltrans_02	-	-802.8642496	-802.6220130	-802.5565620	-800.977918	-800.7356818

21. Cartesian coordinates

Just the conformer of lowest energy is shown

INT0
SCF = -2557.38647560
Num. Imaginary Freq = 1

O	1.837344	0.783005	-1.909218
N	-0.030064	0.398776	-3.122989
C	-0.110084	0.120983	-0.706225
C	0.663850	0.705583	-4.353496
H	0.698134	-0.185736	-5.002372
H	1.684089	1.029800	-4.125812
C	0.637918	0.455195	-1.951736
C	-1.444845	0.100141	-3.265154
H	-1.846625	-0.399861	-2.381661
H	-1.590439	-0.581652	-4.114754
O	-1.478157	0.716966	1.991333
N	0.241368	-0.606721	2.604311
C	0.345750	0.549709	0.470909
C	-0.402079	-0.888152	3.875365
H	0.379240	-0.956680	4.649318
H	-1.039043	-0.024344	4.112225
C	-0.381993	0.231689	1.738586
C	1.479177	-1.300637	2.317678
H	1.718473	-1.243025	1.249201
H	2.320056	-0.881820	2.896214
O	-3.937345	-2.590535	-2.501528
N	-2.848500	-2.054533	-0.524363
N	3.786332	-1.714377	-2.137556
H	3.328803	-0.806565	-2.109168
O	5.253279	-3.126047	-1.132215
C	-4.646292	1.778967	-0.664268
C	-5.325504	-0.211511	-2.498846
H	-5.544543	-1.011581	-3.209134
C	1.705345	-2.989897	-2.304453
C	-0.859572	-3.320399	-1.171703
C	-5.609683	2.028651	-1.649356
H	-6.061109	3.021726	-1.686002
C	0.290918	-3.400947	-0.381553
H	0.181593	-3.576867	0.692830
C	-4.367015	-0.479925	-1.519018
C	-4.040171	0.519520	-0.598792
H	-3.279267	0.267910	0.143486
C	-0.708125	-3.103085	-2.546678
H	-1.616088	-3.038975	-3.153244
C	4.751447	-2.017091	-1.231516
C	3.085657	-2.746643	-2.872958
H	3.706384	-3.651831	-2.809035
H	3.013603	-2.461979	-3.934799
C	-3.678960	-1.839185	-1.533837
C	-2.235450	-3.367053	-0.552338
H	-2.855983	-4.083530	-1.121525
H	-2.125480	-3.748854	0.475673
C	1.556362	-3.243911	-0.937531
H	2.445652	-3.312344	-0.303767
C	0.558496	-2.930867	-3.100695
H	0.659632	-2.737133	-4.173989
C	-5.953207	1.032267	-2.557213
H	-6.706300	1.230833	-3.324809
O	4.660389	3.539020	1.443494
N	3.316833	2.928411	-0.276957
H	2.964702	2.187423	-0.881394
N	-3.406946	2.658309	1.237137
H	-2.950585	1.750804	1.301052
O	-4.697910	4.061436	0.017662
C	5.207672	-0.882595	-0.349699
C	6.165465	1.184128	1.269399
H	6.521078	2.013938	1.882978
C	-1.327613	3.924642	1.383402
C	1.246615	4.152798	0.261282
C	6.398327	-1.042836	0.363993
H	6.934898	-1.988132	0.262975
C	0.107570	4.292668	-0.533603
H	0.217508	4.477607	-1.606195
C	4.973927	1.354088	0.559667
C	4.487012	0.304975	-0.221006
H	3.523510	0.408220	-0.721938
C	1.086334	3.913242	1.629681
H	1.975046	3.802355	2.257663
C	-4.266675	2.932827	0.223957
C	-2.720035	3.713498	1.941722
H	-3.322981	4.625875	1.828188
H	-2.659987	3.468664	3.013146
C	4.296442	2.698130	0.634335
C	2.630670	4.201927	-0.341106
H	3.258212	4.924759	0.198282
H	2.568904	4.519995	-1.393526
C	-1.164677	4.187644	0.020141
H	-2.049635	4.297481	-0.611677
C	-0.187315	3.794658	2.179650
H	-0.300069	3.575786	3.245068
C	6.869415	-0.014688	1.177006
H	7.799566	-0.144160	1.735064
H	1.370913	-2.365826	2.575477
H	0.136411	1.506855	-4.895849
H	-2.025313	1.020203	-3.442322
H	-1.013600	-0.503456	-0.731384
H	1.254237	1.158247	0.538227
C	-1.250663	-2.143731	3.885130
C	-1.140293	-3.056879	4.938444
C	-2.196025	-2.379065	2.878010
C	-1.966739	-4.178888	5.001739
H	-0.397917	-2.886230	5.724256
C	-3.025831	-3.496307	2.947099
H	-2.303959	-1.707794	2.019549
C	-2.917628	-4.397261	4.006523
H	-1.864215	-4.884201	5.830215
H	-3.759103	-3.651200	2.152483
H	-3.569741	-5.272789	4.051854

TS1-A
SCF = -2557.38966886
Num. Imaginary Freq = 1

O	3.280754	1.265570	-1.443190
N	2.193273	2.166426	-3.210418
C	0.972074	0.764312	-1.656363
C	3.404987	2.801729	-3.679756
H	4.236480	2.484066	-3.042191
H	3.313518	3.900013	-3.630725
C	2.228461	1.416215	-2.079601
C	0.980998	2.390053	-3.978366
H	0.135056	2.650380	-3.329225
H	0.706471	1.512848	-4.586904
O	-1.478244	-0.001911	-0.609845
N	-0.446899	-1.391364	0.870531
C	0.854660	0.196369	-0.451943
C	-1.684677	-1.974091	1.360422
H	-1.442335	-2.962102	1.779115
H	-2.567249	-2.415434	0.312210

C	-0.454805	-0.427021	-0.068868
C	0.775276	-1.920636	1.445559
H	1.655928	-1.680982	0.840304
H	0.923898	-1.553578	2.475147
O	-4.068081	-2.268467	-2.664968
N	-3.223136	-2.962226	-0.633975
N	3.608070	-1.993309	-1.304929
H	3.326086	-1.027589	-1.176523
O	4.534120	-3.839396	-0.371380
C	-4.629932	1.386149	-0.380843
C	-5.361522	-1.173308	0.435288
H	-5.576592	-2.181001	0.796761
C	1.593355	-3.099398	-2.199255
C	-1.089131	-3.760420	-1.596876
C	-5.469860	1.222078	0.725099
H	-5.775297	2.102776	1.293696
C	-0.025858	-4.432595	-0.988244
H	-0.240141	-5.217631	-0.256669
C	-4.572157	-1.012509	-0.702693
C	-4.249599	0.272287	-1.126089
H	-3.626537	0.378276	-2.013811
C	-0.792063	-2.760685	-2.532558
H	-1.621979	-2.236766	-3.013100
C	4.242607	-2.655045	-0.303382
C	3.033314	-2.695397	-2.439910
H	3.658066	-3.581338	-2.618575
H	3.104724	-2.043121	-3.323652
C	-3.938473	-2.165270	-1.440528
C	-2.529667	-4.074680	-1.241750
H	-3.062688	-4.374941	-2.159581
H	-2.539062	-4.933033	-0.547419
C	1.297910	-4.113366	-1.283176
H	2.118642	-4.638311	-0.787148
C	0.530718	-2.439194	-2.825270
H	0.744816	-1.647579	-3.550439
C	-5.846512	-0.057388	1.119562
H	-6.468425	-0.191566	2.007020
O	4.258378	2.205706	3.499114
N	3.697983	2.309264	1.306584
H	3.613513	1.839980	0.406391
N	-2.746919	2.664431	-1.112896
H	-2.261904	1.763077	-1.053630
O	-4.621069	3.786493	-0.469797
C	4.515542	-1.861386	0.950443
C	4.818939	-0.526225	3.387669
H	4.904368	0.017063	4.330457
C	-0.640728	3.838455	-0.630410
C	1.860173	3.780753	0.684524
C	4.888674	-2.567082	2.096934
H	5.031994	-3.645851	2.012815
C	1.719493	4.403671	-0.554958
H	2.593467	4.858200	-1.030673
C	4.453458	0.192526	2.246133
C	4.329372	-0.480447	1.029638
H	4.077309	0.075459	0.127463
C	0.727002	3.198425	1.267607
H	0.816281	2.698129	2.236206
C	-4.038863	2.731045	-0.663161
C	-1.973079	3.851627	-1.347131
H	-1.797449	4.012333	-2.427859
H	-2.588626	4.699524	-1.003212
C	4.144357	1.661364	2.412409
C	3.210915	3.670361	1.353779
H	3.159047	3.953050	2.414755
H	3.925265	4.356367	0.866247
C	0.485177	4.430472	-1.204273
H	0.400947	4.911374	-2.183676
C	-0.503987	3.232567	0.624005
H	-1.375010	2.758020	1.080762
C	5.048053	-1.897762	3.308228
H	5.332996	-2.454358	4.203797
H	0.709184	-3.017310	1.472682
H	3.614855	2.514543	-4.722858
H	1.149416	3.236489	-4.656857
H	0.104244	0.729335	-2.314842
H	1.688612	0.241730	0.251027
C	-2.447098	-1.173180	2.330699
C	-2.332945	0.223873	2.460548
C	-3.395200	-1.812359	3.159345
C	-3.119023	0.937903	3.362077
H	-1.615587	0.759036	1.838855
C	-4.176222	-1.099353	4.059559
H	-3.513264	-2.897369	3.080313
C	-4.048652	0.288062	4.171485
H	-3.004308	2.024026	3.426543
H	-4.898204	-1.632904	4.684967
H	-4.666785	0.851164	4.874630

INT1-A
SCF = -2557.42260976
Num. Imaginary Freq = 0

O	-1.203164	-0.279545	2.732739
N	0.798675	-0.798232	3.652417
C	0.696951	-0.099450	1.327237
C	2.214173	-1.080044	3.554311
H	2.518711	-1.247072	2.514949
H	2.444862	-1.999126	4.116751
C	0.018197	-0.395975	2.602955
C	0.197984	-0.983676	4.952711
H	0.254681	-2.040360	5.266842
H	-0.855013	-0.686951	4.898415
O	2.004403	-0.126841	-1.221935
N	-0.032397	0.329666	-2.147100
C	0.061360	-0.255143	0.155843
C	-1.348832	0.797036	-1.903860
H	-1.454095	1.644333	-1.229298
C	0.756093	-0.020258	-1.120565
C	0.593038	0.495200	-3.450531
H	-0.194312	0.557611	-4.210647
H	1.189159	1.422977	-3.477858
O	5.370155	-3.324293	1.061218
N	3.437352	-2.385555	0.375607
H	2.949548	-1.660751	-0.161854
N	-3.462577	-2.067984	-0.487967
H	-3.100335	-1.223024	-0.923962
O	-5.276814	-2.957383	0.561887
C	5.654267	1.041421	-0.912181
C	6.934151	-1.320517	-0.143604
H	7.407761	-2.254865	0.163790
C	-1.439580	-3.426241	-0.466164
C	1.251617	-3.506733	0.377668
C	7.040893	0.924006	-1.032826
H	7.601655	1.775468	-1.423706
C	0.931539	-3.445572	-0.980603
H	1.732592	-3.408192	-1.723442
C	5.547135	-1.208690	-0.012427
C	4.919919	-0.018821	-0.379737
H	3.842665	0.086196	-0.253405
C	0.207865	-3.530266	1.307581
H	0.440268	-3.556041	2.375938
C	-4.615566	-1.989396	0.222538
C	-2.883033	-3.321262	-0.904022
H	-2.954078	-3.421253	-1.998581
H	-3.492346	-4.115033	-0.448703
C	4.788582	-2.392258	0.524904
C	2.689208	-3.545290	0.825881
H	2.739954	-3.621087	1.925221

H	3.188568	-4.453760	0.446927
C	-0.397346	-3.401869	-1.395173
H	-0.632543	-3.332602	-2.459973
C	-1.119242	-3.488064	0.893693
H	-1.921923	-3.473144	1.635375
C	7.676464	-0.256063	-0.651298
H	8.760696	-0.347277	-0.750143
O	-3.780100	3.933551	1.521815
N	-2.291928	2.335919	2.129759
H	-2.070214	1.342712	2.235936
N	3.672197	2.247420	-1.596149
O	5.628836	3.380851	-1.347800
C	-5.018231	-0.585796	0.578287
C	-5.811323	2.018583	1.176098
H	-6.090215	3.052427	1.387975
C	1.779678	3.443185	-0.614564
C	-0.178144	3.389426	1.417577
C	-6.371100	-0.244892	0.554672
H	-7.101022	-1.013971	0.294169
C	1.166635	3.160434	1.714244
H	1.458939	2.924231	2.741977
C	-4.460184	1.670917	1.257966
C	-4.067256	0.368867	0.942116
H	-3.013943	0.084339	0.979033
C	-0.527398	3.678630	0.096425
H	-1.575738	3.864265	-0.150879
C	4.994506	2.337257	-1.307711
C	2.830793	3.414669	-1.705376
H	3.492517	4.291523	-1.640810
H	2.338883	3.449409	-2.691728
C	-3.484424	2.751531	1.641136
C	-1.253505	3.278052	2.477827
H	-1.741625	4.253204	2.622642
H	-0.800487	2.976400	3.435341
C	2.134489	3.190799	0.713699
H	3.179175	2.984146	0.961471
C	0.437259	3.693009	-0.907317
H	0.131409	3.871129	-1.941801
C	-6.764212	1.061014	0.837695
H	-7.820654	1.335665	0.791516
H	1.265865	-0.346208	-3.668789
H	2.827557	-0.263530	3.975132
H	0.717065	-0.372049	5.710486
H	1.723280	0.270311	1.324097
H	-0.967503	-0.615457	0.112827
C	-2.457580	0.258923	-2.587573
C	-2.386683	-0.889044	-3.437930
C	-3.764777	0.816994	-2.413230
C	-3.523711	-1.430996	-4.028730
H	-1.420540	-1.374331	-3.597068
C	-4.884660	0.250474	-2.993897
H	-3.876136	1.696237	-1.772646
C	-4.790328	-0.886722	-3.811589
H	-3.416726	-2.317198	-4.663093
H	-5.862540	0.701504	-2.800545
H	-5.679501	-1.329703	-4.264256
H	3.185808	1.345231	-1.565923

***trans*-TS2-A**
SCF = -2557.42092659
Num. Imaginary Freq = 1

O	4.568320	3.141705	1.350182
O	4.451274	-3.897903	-0.015807
O	-5.086012	-3.366783	-0.484303
N	2.741014	1.922982	1.924708
H	2.265846	1.017489	1.950291
N	2.685102	-2.940232	1.042841
H	2.261189	-2.088746	1.418181
N	-3.628691	-2.030398	-1.593184
H	-3.249909	-1.093726	-1.733369
C	-5.412059	1.370191	0.222693
C	-6.700427	1.233751	0.745311
H	-7.211178	2.131697	1.098631
C	-7.298207	-0.023376	0.810187
H	-8.304616	-0.127310	1.222536
C	-6.621223	-1.147404	0.340732
H	-7.071818	-2.141414	0.368002
C	-5.333934	-1.018674	-0.187348
C	-4.724764	0.235821	-0.210424
H	-3.695264	0.330845	-0.560437
C	-2.867396	4.037216	-0.597069
H	-3.451898	4.843978	-0.129931
H	-2.618802	4.353163	-1.622713
C	-1.593829	3.790499	0.186499
C	-0.349753	4.212744	-0.289191
H	-0.284978	4.715721	-1.258699
C	0.808575	3.994151	0.452954
H	1.780322	4.316642	0.069509
C	0.749313	3.337833	1.684910
C	-0.491760	2.900905	2.151120
H	-0.550308	2.362205	3.100732
C	-1.648858	3.126069	1.414413
H	-2.609378	2.774064	1.796897
C	2.011250	3.047603	2.459683
H	2.692453	3.908682	2.429457
H	1.756315	2.851250	3.514755
C	3.990266	2.065371	1.412799
C	4.668610	0.788371	0.998619
C	6.054720	0.797772	0.831989
H	6.580119	1.747382	0.949099
C	6.729491	-0.380783	0.522434
H	7.813699	-0.368478	0.386943
C	6.027429	-1.578220	0.398418
H	6.533570	-2.519091	0.173329
C	4.641536	-1.595491	0.567705
C	3.967284	-0.402624	0.826825
H	2.878274	-0.400374	0.886862
C	3.924168	-2.914441	0.484383
C	1.858070	-4.119962	1.009631
H	1.553953	-4.396933	2.033111
H	2.488309	-4.932929	0.618670
C	0.625553	-3.945575	0.149507
C	-0.639603	-4.316960	0.611064
H	-0.741349	-4.754238	1.608961
C	-1.774168	-4.111059	-0.168643
H	-2.765783	-4.379834	0.205732
C	-1.666905	-3.508998	-1.426252
C	-0.399905	-3.168978	-1.900411
H	-0.301430	-2.694902	-2.881302
C	0.733592	-3.385042	-1.124010
H	1.715633	-3.080914	-1.493990
C	-2.912765	-3.137753	-2.197062
H	-2.649127	-2.860374	-3.228702
H	-3.609881	-3.986302	-2.234498
C	-4.667670	-2.247154	-0.746774
O	-5.281236	3.675780	0.797742
N	-3.712545	2.869506	-0.635672
H	-3.301384	2.054360	-1.097078
C	-4.800463	2.747275	0.164321
O	1.084778	-0.564140	2.008258
O	-2.117186	0.765350	-1.850089
N	-0.865510	-1.511328	2.678304
N	0.058371	0.941937	-2.588851
C	-0.199565	1.490492	-3.896729
H	-0.985360	2.256346	-3.828675
H	-0.534673	0.713772	-4.606186
C	2.496922	0.705731	-2.258533

C	3.636028	-0.106810	-2.451350
H	3.503378	-1.183564	-2.591529
C	4.913617	0.435895	-2.464424
H	5.774878	-0.222164	-2.605037
C	5.106598	1.806690	-2.273929
H	6.113994	2.227911	-2.260670
C	3.996117	2.624156	-2.064247
H	4.135225	3.691837	-1.877524
C	2.712477	2.086633	-2.058953
H	1.847043	2.728739	-1.876895
C	-2.292300	-1.692102	2.536635
H	-2.755387	-0.802635	2.086242
H	-2.736080	-1.834105	3.533419
C	-0.235207	-2.085643	3.838540
H	-0.367548	-3.183443	3.865196
H	-0.654665	-1.671958	4.773019
C	1.168115	0.141035	-2.223636
H	1.066157	-0.926299	-2.434377
C	-0.170616	0.327853	-0.423999
H	0.617167	1.005354	-0.084705
C	-0.886523	0.732951	-1.653243
C	-0.726296	-0.561880	0.472321
C	-0.107673	-0.853811	1.732010
H	0.723721	1.945365	-4.282668
H	0.834926	-1.852192	3.805372
H	-2.558198	-2.565179	1.915466
H	-1.617999	-1.114652	0.176840

***cis*-TS2-A**
SCF = -2557.41238293
Num. Imaginary Freq = 1

O	-6.295815	2.499278	-0.129322
O	-4.091458	-4.293361	0.546870
O	5.141163	-3.076435	-1.701202
N	-4.151981	2.039559	0.479920
H	-3.399504	1.342814	0.532031
N	-2.711099	-2.572992	1.103291
H	-2.488236	-1.582234	0.963868
N	3.199278	-2.550396	-2.757482
H	2.552752	-1.785129	-2.894495
C	3.683757	1.509717	-1.682679
C	4.472658	1.776705	-0.559536
H	4.432710	2.755218	-0.079443
C	5.299249	0.781985	-0.039272
H	5.920877	0.995053	0.832685
C	5.301443	-0.492434	-0.597465
H	5.907013	-1.298284	-0.178567
C	4.480897	-0.784088	-1.691858
C	3.719212	0.239451	-2.257504
H	3.112063	0.091577	-3.153764
C	1.373780	4.492785	-1.941210
H	1.316192	4.425909	-3.035549
H	1.742847	5.497345	-1.676518
C	0.016592	4.269170	-1.315098
C	-0.284941	4.821729	-0.069732
H	0.454534	5.449311	0.433642
C	-1.494451	4.540244	0.558992
H	-1.700909	4.960841	1.547268
C	-2.439273	3.709638	-0.045820
C	-2.139483	3.160476	-1.296530
H	-2.858913	2.490976	-1.774250
C	-0.925625	3.431895	-1.919720
H	-0.694672	2.979622	-2.888170
C	-3.753837	3.415151	0.642276
H	-3.667845	3.674815	1.711931
H	-4.564338	4.035757	0.227929
C	-5.402898	1.693476	0.085343
C	-5.602996	0.216644	-0.131714
C	-6.604312	-0.207942	-1.008632
H	-7.257346	0.545825	-1.453305
C	-6.740554	-1.562653	-1.307234
H	-7.522724	-1.892170	-1.995212
C	-5.863459	-2.495148	-0.755183
H	-5.925532	-3.557383	-0.999996
C	-4.863812	-2.078780	0.127092
C	-4.776522	-0.730371	0.470728
H	-4.033986	-0.407211	1.197719
C	-3.865348	-3.093062	0.620200
C	-1.616950	-3.422786	1.524487
H	-2.043599	-4.406190	1.763310
H	-1.171553	-3.002176	2.438613
C	-0.543869	-3.557045	0.466288
C	-0.724019	-4.376986	-0.654472
H	-1.656941	-4.938589	-0.756672
C	0.264649	-4.465453	-1.634593
H	0.101900	-5.102284	-2.509605
C	1.462407	-3.751897	-1.511237
C	1.631629	-2.935182	-0.390083
H	2.545312	-2.355042	-0.248756
C	0.639364	-2.827536	0.577558
H	0.797641	-2.161284	1.428463
C	2.554896	-3.835146	-2.566149
H	3.343374	-4.539081	-2.260094
H	2.138334	-4.200295	-3.518109
C	4.345685	-2.235756	-2.074993
O	2.433680	2.412179	-3.492849
N	2.334051	3.503892	-1.496029
H	2.385832	3.366544	-0.473173
C	2.761320	2.521582	-2.316615
O	-1.864862	0.249365	0.598235
O	2.275919	3.197141	1.278570
N	-0.831433	-0.246754	-1.354551
N	1.806915	2.155388	3.266242
C	2.852730	2.776968	4.039882
H	2.497746	2.952299	5.067252
H	3.134369	3.731279	3.573403
C	1.780043	-0.313516	3.417098
C	2.865745	-0.500603	2.517963
H	3.259590	0.352676	1.958801
C	3.461365	-1.747570	2.349961
H	4.300602	-1.843774	1.655284
C	2.998210	-2.872670	3.032794
H	3.462767	-3.848840	2.881799
C	1.905993	-2.719779	3.895308
H	1.512447	-3.587925	4.432610
C	1.309407	-1.481004	4.079945
H	0.464450	-1.385086	4.768556
C	0.341749	-0.245071	-2.197808
H	0.145441	-0.878948	-3.073667
H	1.206737	-0.667685	-1.667258
C	-2.021140	-0.885376	-1.870841
H	-1.948858	-1.986081	-1.813940
H	-2.177791	-0.595619	-2.921639
C	1.151118	0.950213	3.643334
H	0.475882	1.030670	4.504175
C	0.400416	1.645891	1.532052
H	-0.543672	1.827161	2.049365
C	1.583388	2.443105	1.973038
C	0.392506	0.939770	0.363590
C	-0.826792	0.302869	-0.102942
H	3.744932	2.128650	4.089315
H	-2.891966	-0.564621	-1.291185
H	0.606600	0.763831	-2.554468
H	1.316754	0.851901	-0.206410

***trans*-INT2-A**
SCF = -2557.44196130
Num. Imaginary Freq = 0

O	-3.933900	4.223005	0.279044
O	-4.961382	-2.699537	-1.315094
O	3.100691	-2.589405	2.091791
N	-2.321307	3.055787	-0.817676
H	-1.952460	2.150244	-1.151033
N	-3.036557	-1.666659	-1.933448
H	-2.420441	-0.843468	-1.881409
N	3.297805	-4.008945	0.332192
H	3.599312	-4.039675	-0.633319
C	5.119334	0.404578	-0.092613
C	6.016773	-0.010456	-1.081239
H	6.567259	0.761977	-1.621000
C	6.189519	-1.362829	-1.358523
H	6.906867	-1.678805	-2.119151
C	5.439276	-2.315487	-0.672528
H	5.585209	-3.376078	-0.894582
C	4.525983	-1.912059	0.307104
C	4.392247	-0.555208	0.613719
H	3.686865	-0.287054	1.403135
C	3.780976	3.690086	1.252031
H	4.554986	4.291379	0.757282
H	3.777064	3.937065	2.324752
C	2.419161	3.950705	0.651310
C	1.274353	3.967290	1.452344
H	1.376481	3.860345	2.535002
C	0.011604	4.071934	0.881160
H	-0.882175	4.068854	1.511588
C	-0.136480	4.152392	-0.505864
C	1.008182	4.147630	-1.304044
H	0.904817	4.197091	-2.391769
C	2.274449	4.052304	-0.733698
H	3.166070	4.018257	-1.366102
C	-1.516525	4.217001	-1.113855
H	-2.064388	5.089117	-0.728793
H	-1.425339	4.332772	-2.207845
C	-3.516337	3.169110	-0.185543
C	-4.371678	1.927536	-0.173504
C	-5.721313	2.051657	0.162043
H	-6.082775	3.026699	0.494975
C	-6.573153	0.953465	0.042995
H	-7.629513	1.053430	0.305192
C	-6.089296	-0.260853	-0.438489
H	-6.740472	-1.127222	-0.570582
C	-4.737396	-0.392881	-0.765061
C	-3.881146	0.690254	-0.587689
H	-2.811618	0.570420	-0.767791
C	-4.256460	-1.696566	-1.342080
C	-2.477602	-2.848051	-2.550472
H	-2.159861	-2.599992	-3.577039
H	-3.280608	-3.594117	-2.608706
C	-1.294697	-3.386802	-1.779821
C	-0.074723	-2.714698	-1.838476
H	0.040033	-1.825380	-2.464768
C	1.005639	-3.136122	-1.069453
H	1.921477	-2.544634	-1.109484
C	0.904796	-4.253310	-0.237906
C	-0.316385	-4.935403	-0.188098
H	-0.421694	-5.810380	0.460765
C	-1.404464	-4.502747	-0.942612
H	-2.361143	-5.027602	-0.872869
C	2.073515	-4.720792	0.614780
H	1.849891	-4.561133	1.679414
H	2.232475	-5.802339	0.474000
C	3.596257	-2.857793	1.014859
O	5.611562	2.671972	-0.596043
N	4.146185	2.296050	1.094268
H	3.500099	1.649795	1.558514
C	4.978845	1.897847	0.112404
O	-1.085176	0.574024	-1.643218
O	2.098103	1.014133	2.566481
N	0.896836	0.474591	-2.793617
N	0.448067	-0.626337	2.674900
C	0.706573	-1.448772	3.818541
H	1.450132	-0.935818	4.443494
H	1.126004	-2.420219	3.516287
C	-1.895741	-0.884091	1.875709
C	-2.656044	-2.004660	1.532750
H	-2.186374	-2.812355	0.966577
C	-4.002699	-2.088528	1.882599
H	-4.588734	-2.954364	1.568332
C	-4.607686	-1.044285	2.578174
H	-5.669557	-1.094585	2.828976
C	-3.859613	0.081132	2.923788
H	-4.332991	0.912904	3.450473
C	-2.511924	0.155659	2.582326
H	-1.929072	1.037867	2.860424
C	2.336144	0.488990	-2.786151
H	2.731287	-0.309790	-2.139635
H	2.766180	1.446867	-2.425195
C	0.284530	1.136231	-3.918223
H	-0.804250	1.067634	-3.812659
H	0.587697	0.654250	-4.863425
C	-0.435603	-0.819809	1.523991
H	-0.142255	-1.696807	0.926436
C	0.195052	0.503286	0.932168
H	-0.538436	1.326714	1.012981
C	1.100698	0.433882	2.160822
C	0.902625	0.501753	-0.374774
C	0.200230	0.534769	-1.555865
H	-0.217791	-1.601020	4.398568
H	0.559599	2.211554	-3.986201
H	2.709029	0.310849	-3.806895
H	1.987948	0.578082	-0.351141

***cis*-INT2-A**
SCF = -2557.43967330
Num. Imaginary Freq = 0

O	-5.463643	2.538747	1.000946
O	-3.623831	-4.222196	-0.398485
O	4.558947	-2.750689	1.257151
N	-3.775347	2.130351	-0.465751
H	-3.043476	1.475936	-0.797111
N	-2.518191	-2.527823	-1.442106
H	-2.274654	-1.527373	-1.434034
N	4.298916	-2.116259	-0.897909
H	4.100967	-1.340766	-1.514739
C	3.877102	1.944594	0.511867
C	4.239884	2.210628	1.834032
H	4.185715	3.239994	2.190011
C	4.636631	1.171955	2.670306
H	4.899908	1.379326	3.709895
C	4.698736	-0.133187	2.187537
H	5.005310	-0.962757	2.826799
C	4.358181	-0.414271	0.861809
C	3.937631	0.635478	0.034603
H	3.636860	0.461348	-1.000193
C	1.957799	3.899846	-2.105176
H	2.194881	3.696317	-3.163797
H	2.464859	4.833660	-1.822882
C	0.463221	3.995001	-1.921343
C	-0.060735	4.546821	-0.749552
H	0.615300	4.981866	-0.011671
C	-1.419927	4.448447	-0.473165

H	-1.807724	4.830370	0.475214
C	-2.287120	3.805631	-1.360786
C	-1.770619	3.311064	-2.560407
H	-2.430208	2.787953	-3.256770
C	-0.409626	3.401710	-2.834546
H	-0.010430	2.955155	-3.749867
C	-3.707769	3.492033	-0.961798
H	-4.066086	4.166544	-0.170457
H	-4.386980	3.620314	-1.824822
C	-4.678624	1.762981	0.472255
C	-4.656320	0.299759	0.840851
C	-5.202723	-0.109688	2.059495
H	-5.636699	0.648641	2.714573
C	-5.194912	-1.460278	2.409254
H	-5.625703	-1.777564	3.361888
C	-4.629914	-2.406026	1.554481
H	-4.599861	-3.466475	1.813258
C	-4.067009	-2.002011	0.341106
C	-4.122080	-0.657395	-0.020803
H	-3.729176	-0.345992	-0.984883
C	-3.394124	-3.028936	-0.531171
C	-1.805074	-3.388503	-2.350296
H	-1.973118	-3.056853	-3.388877
H	-2.250147	-4.390299	-2.246437
C	-0.313252	-3.470343	-2.096028
C	0.567830	-3.792603	-3.130787
H	0.175169	-3.980541	-4.134955
C	1.947052	-3.835968	-2.913247
H	2.620400	-4.048778	-3.749485
C	2.471979	-3.566180	-1.649708
C	1.582758	-3.288643	-0.607124
H	1.966768	-3.078669	0.391168
C	0.213842	-3.231700	-0.824677
H	-0.446950	-2.960342	-0.000431
C	3.959729	-3.441355	-1.390560
H	4.287508	-4.148232	-0.615693
H	4.533162	-3.651447	-2.306659
C	4.417542	-1.858170	0.435286
O	4.089115	4.163345	-0.299417
N	2.494403	2.846882	-1.259797
H	1.832165	2.096676	-1.023362
C	3.491639	3.102926	-0.373460
O	-1.799750	0.366549	-1.495953
O	1.204590	3.082872	1.653181
N	0.073263	-0.298069	-2.594402
N	-0.071233	1.552499	2.868327
C	0.205720	1.755350	4.255911
H	0.661938	0.857487	4.707122
H	-0.706532	2.006631	4.822986
C	-0.296071	-0.876379	2.403044
C	-1.188995	-1.942400	2.530187
H	-2.260717	-1.752398	2.443095
C	-0.727091	-3.238102	2.759939
H	-1.441648	-4.061297	2.835264
C	0.640207	-3.478002	2.885896
H	1.007627	-4.491536	3.062755
C	1.542881	-2.418991	2.766286
H	2.618469	-2.603127	2.826386
C	1.076875	-1.130385	2.516016
H	1.791093	-0.308668	2.411429
C	1.499590	-0.352098	-2.719056
H	1.766052	-0.970277	-3.588787
H	1.940770	-0.836119	-1.836767
C	-0.667746	-0.341560	-3.824554
H	-0.451845	-1.276707	-4.372827
H	-0.428148	0.506851	-4.500140
C	-0.810239	0.513688	2.165096
H	-1.897672	0.547095	2.353187
C	-0.393511	1.259357	0.816375
H	-1.274943	1.821502	0.473971
C	0.418200	2.166873	1.754788
C	0.252898	0.614784	-0.350176
C	-0.525824	0.250100	-1.445811
H	0.915773	2.590950	4.336329
H	-1.737547	-0.295161	-3.592861
H	1.966099	0.647759	-2.842577
H	1.285639	0.281804	-0.255295

***trans*-INT3**
SCF = -2557.45921258
Num. Imaginary Freq = 0

O	4.599345	-3.547490	-1.011974
O	4.798476	3.601515	-1.075647
O	-2.695610	1.618744	1.873978
N	2.632105	-2.474345	-1.341965
H	2.093690	-1.608565	-1.295678
N	2.781091	2.616400	-1.390455
H	2.222455	1.766686	-1.327911
N	-3.769263	3.492788	1.023921
C	-5.154170	-0.890662	-0.367802
C	-6.086055	-0.328390	-1.246233
H	-6.627365	-0.993865	-1.921350
C	-6.285300	1.051239	-1.259477
H	-7.021805	1.486194	-1.940815
C	-5.524020	1.880353	-0.436959
H	-5.612470	2.968222	-0.460891
C	-4.573498	1.334349	0.431917
C	-4.432992	-0.051209	0.489208
H	-3.694501	-0.423633	1.199772
C	-3.457610	-4.208395	0.260163
H	-4.100736	-4.766626	-0.435165
H	-3.448959	-4.737493	1.225260
C	-2.046944	-4.118095	-0.287632
C	-0.941100	-4.558092	0.443208
H	-1.093155	-4.998982	1.431728
C	0.350355	-4.423407	-0.064351
H	1.206745	-4.771956	0.519954
C	0.569394	-3.831403	-1.311182
C	-0.538743	-3.386377	-2.039574
H	-0.385610	-2.919872	-3.016894
C	-1.828835	-3.532600	-1.539698
H	-2.688646	-3.186063	-2.120043
C	1.965322	-3.654703	-1.853315
H	2.598925	-4.511940	-1.589613
H	1.920596	-3.612069	-2.956740
C	3.957396	-2.507156	-1.045045
C	4.636217	-1.185889	-0.792744
C	5.958517	-1.212246	-0.341166
H	6.419037	-2.185492	-0.162752
C	6.653413	-0.024408	-0.136247
H	7.686735	-0.050938	0.217620
C	6.034798	1.198690	-0.383784
H	6.556374	2.146662	-0.239664
C	4.715725	1.238317	-0.840902
C	4.028330	0.041443	-1.052096
H	3.013426	0.063347	-1.443620
C	4.111164	2.592442	-1.111030
C	2.077366	3.854247	-1.634394
H	1.803069	3.940791	-2.700808
H	2.794792	4.663054	-1.427875
C	0.837411	4.001321	-0.781211
C	-0.303148	4.649598	-1.265696
H	-0.305180	5.049070	-2.285461
C	-1.438493	4.783934	-0.470145
H	-2.335402	5.262247	-0.872459
C	-1.474471	4.276126	0.834105
C	-0.330744	3.636003	1.315626

H	-0.352418	3.211582	2.321853
C	0.807799	3.507700	0.523794
H	1.690554	2.995116	0.910401
C	-2.760284	4.321423	1.636220
H	-2.529464	4.019621	2.676657
H	-3.122429	5.365064	1.666132
C	-3.599748	2.207239	1.211353
O	-5.367431	-3.028984	-1.401830
N	-4.041684	-2.900385	0.429476
H	-3.571042	-2.320156	1.124773
C	-4.884643	-2.367044	-0.490100
O	0.911404	0.075263	-1.416195
O	-2.206635	-1.907464	2.327940
N	-0.918751	1.123720	-2.244643
N	-0.448819	-0.466513	2.860199
C	-0.773887	0.195002	4.092909
H	0.143087	0.400406	4.665309
H	-1.424808	-0.465326	4.681425
C	1.907065	-0.396568	2.045969
C	2.849369	0.608255	2.271927
H	2.540610	1.654898	2.222442
C	4.176747	0.294603	2.565411
H	4.901710	1.095780	2.723624
C	4.575734	-1.036073	2.638663
H	5.617299	-1.285434	2.851751
C	3.643972	-2.050985	2.414420
H	3.955793	-3.097227	2.447684
C	2.321853	-1.732364	2.124300
H	1.599220	-2.534062	1.954062
C	-2.310889	1.533252	-2.099252
H	-2.631964	2.007799	-3.034731
H	-2.443689	2.258487	-1.280572
C	-0.182288	1.673695	-3.361170
H	0.862201	1.349824	-3.302206
H	-0.219890	2.773416	-3.338256
C	0.461620	-0.049898	1.788519
H	0.349704	1.018499	1.551016
C	-0.371159	-0.949532	0.831028
H	0.166237	-1.807034	0.407450
C	-1.211613	-1.256392	2.075868
C	-1.157088	-0.195055	-0.220474
H	-1.929834	-0.855943	-0.646203
H	-1.699034	0.632194	0.275319
C	-0.298026	0.340922	-1.336894
H	-1.321749	1.126894	3.873236
H	-0.611077	1.326834	-4.315969
H	-2.973105	0.676376	-1.913943

***cis*-INT3**
SCF = -2557.45291577
Num. Imaginary Freq = 0

O	-3.918909	4.386997	-1.022464
O	-5.420991	-2.575619	-1.151211
O	2.413153	-2.175003	1.770591
N	-2.221776	2.956580	-1.517400
H	-1.849600	2.007008	-1.428404
N	-3.282047	-1.940773	-1.597632
H	-2.578983	-1.210674	-1.470265
N	3.162867	-3.937147	0.443373
C	5.196245	0.332627	-0.182465
C	6.152309	-0.254232	-1.017721
H	6.825180	0.403069	-1.571212
C	6.208479	-1.639480	-1.148648
H	6.962642	-2.094441	-1.796824
C	5.280995	-2.441911	-0.489196
H	5.248169	-3.524950	-0.618835
C	4.311370	-1.873687	0.344869
C	4.306989	-0.489517	0.526298
H	3.549235	-0.096832	1.209754
C	3.857681	3.794771	0.535579
H	4.639974	4.307334	-0.042963
H	3.799753	4.259173	1.531365
C	2.519405	3.925905	-0.167305
C	1.432230	4.571057	0.428226
H	1.552822	5.020674	1.417516
C	0.189782	4.614229	-0.203212
H	-0.658339	5.098880	0.288486
C	0.003422	4.009782	-1.449828
C	1.097315	3.382495	-2.055422
H	0.970072	2.904717	-3.031070
C	2.338063	3.341494	-1.426218
H	3.183393	2.843989	-1.908688
C	-1.361501	3.969800	-2.093921
H	-1.882957	4.928613	-1.970104
H	-1.247941	3.788947	-3.176939
C	-3.475186	3.250641	-1.083158
C	-4.313250	2.066907	-0.675378
C	-5.386489	2.273725	0.195818
H	-5.574469	3.286667	0.556927
C	-6.193821	1.202732	0.574777
H	-7.032221	1.367594	1.255258
C	-5.931440	-0.078771	0.093428
H	-6.546071	-0.934971	0.378563
C	-4.852376	-0.298015	-0.765660
C	-4.072185	0.784904	-1.170707
H	-3.258384	0.627289	-1.875742
C	-4.558733	-1.712831	-1.195656
C	-2.815844	-3.261265	-1.955692
H	-2.641335	-3.325375	-3.044959
H	-3.641738	-3.953557	-1.729415
C	-1.555863	-3.667797	-1.220186
C	-0.649547	-4.565101	-1.797954
H	-0.848601	-4.960364	-2.800082
C	0.504324	-4.958936	-1.119596
H	1.206895	-5.648571	-1.596329
C	0.789708	-4.465826	0.158528
C	-0.117603	-3.568594	0.724115
H	0.130211	-3.149047	1.696047
C	-1.273245	-3.176596	0.055887
H	-1.954386	-2.467250	0.531326
C	2.083223	-4.783636	0.884955
H	1.891497	-4.692288	1.972965
H	2.350793	-5.836759	0.682848
C	3.197716	-2.725763	0.949953
O	5.742338	2.490048	-1.035270
N	4.258511	2.415368	0.667716
H	3.683195	1.863811	1.303777
C	5.109540	1.835348	-0.214159
O	-1.110582	0.197251	-1.323484
O	2.186717	1.845927	2.441214
N	0.577981	-1.056032	-2.155621
N	-0.130001	1.855749	2.713067
C	-0.418415	2.231590	4.063989
H	-1.098299	3.097724	4.104074
H	0.530829	2.497062	4.549314
C	-1.585009	-0.064917	2.187321
C	-2.963798	-0.249954	2.062015
H	-3.560121	0.503460	1.544498
C	-3.583940	-1.378568	2.595093
H	-4.659853	-1.515481	2.465434
C	-2.829574	-2.321228	3.290733
H	-3.309983	-3.206780	3.712565
C	-1.454963	-2.134564	3.436183
H	-0.853330	-2.876401	3.965482
C	-0.830241	-1.021345	2.878495
H	0.256624	-0.921596	2.966179
C	1.890488	-1.675043	-2.015545

H	2.080962	-2.285099	-2.906036
H	1.945924	-2.345451	-1.147341
C	-0.144675	-1.323118	-3.380487
H	-1.110383	-0.808032	-3.355540
H	-0.315036	-2.404688	-3.492586
C	-0.956064	1.210344	1.687102
H	-1.734606	1.872603	1.275408
C	0.335607	1.183364	0.802886
H	0.278663	2.039968	0.118534
C	1.046392	1.663907	2.069278
C	0.855806	-0.009222	0.033859
H	1.886844	0.228864	-0.268387
H	0.968591	-0.924738	0.645330
C	0.029428	-0.286232	-1.197836
H	-0.877498	1.394079	4.615466
H	0.430643	-0.963138	-4.249339
H	2.692105	-0.924905	-1.936204

TS1-B
SCF = -2557.39727829
Num. Imaginary Freq = 1

O	1.139850	0.568565	0.136439
N	-0.345137	1.255211	1.656581
C	-1.159301	0.504514	-0.575940
C	0.773440	1.555995	2.539271
H	0.416510	2.282138	3.284982
C	-0.042107	0.798188	0.412321
C	-1.669526	1.585084	2.134088
H	-2.439493	1.035485	1.587760
H	-1.881826	2.661771	2.034024
O	-2.920514	-0.708837	-2.460379
N	-1.181246	-1.711450	-3.512815
C	-0.740118	-0.164699	-1.718040
C	0.220933	-2.035761	-3.603626
H	0.335368	-2.990526	-4.139053
H	0.808659	-1.271353	-4.146357
C	-1.684513	-0.847287	-2.554258
C	-2.067612	-2.301831	-4.479979
H	-1.766845	-2.048312	-5.513208
H	-2.086696	-3.404977	-4.395551
O	-2.849991	4.351470	-0.606780
N	-1.849798	2.326093	-1.014344
N	4.570136	2.095588	0.243044
H	3.749851	1.555104	0.495131
O	6.378665	2.079210	-1.126698
C	-5.415858	0.468184	0.391759
C	-4.749059	3.092186	1.091796
H	-4.468175	4.117551	1.342785
C	2.946227	3.669279	-0.539051
C	0.361129	3.287616	-1.572282
C	-6.135300	1.134702	1.388028
H	-6.928267	0.589844	1.904034
C	1.486638	2.917818	-2.312537
H	1.351122	2.409123	-3.270975
C	-3.999900	2.422892	0.120855
C	-4.372149	1.134012	-0.259706
H	-3.802860	0.664757	-1.065394
C	0.545322	3.947367	-0.355170
H	-0.338890	4.274154	0.198231
C	5.477811	1.478105	-0.566450
C	4.299562	3.522678	0.114662
H	5.115184	3.946461	-0.487778
H	4.317049	4.006670	1.103675
C	-2.823088	3.115657	-0.528642
C	-1.019920	2.895566	-2.037427
H	-1.527494	3.774572	-2.468713
H	-0.904109	2.139657	-2.837133
C	2.763725	3.113883	-1.809757
H	3.633105	2.762042	-2.373245
C	1.827981	4.123090	0.162754
H	1.958878	4.577500	1.150204
C	-5.817090	2.451207	1.717985
H	-6.393760	2.973951	2.485573
O	3.164455	-4.149697	0.226457
N	1.839685	-2.316385	0.381118
H	1.695329	-1.326066	0.174108
N	-5.002845	-1.603834	-0.821773
H	-4.358898	-1.060560	-1.403944
O	-6.365855	-1.626439	0.993850
C	5.324774	-0.014406	-0.722361
C	5.208883	-2.786238	-1.045182
H	5.149537	-3.873351	-1.120604
C	-3.192222	-3.084082	-0.190702
C	-0.532320	-2.992237	0.746003
C	6.401293	-0.705399	-1.285376
H	7.280167	-0.130187	-1.581996
C	-1.572721	-2.435657	1.493644
H	-1.343826	-1.944033	2.443771
C	4.115858	-2.108295	-0.497587
C	4.172021	-0.718940	-0.357346
H	3.298623	-0.193395	0.033864
C	-0.839803	-3.611446	-0.466525
H	-0.033625	-4.047857	-1.062446
C	-5.668369	-1.006894	0.200790
C	-4.609909	-2.994164	-0.727381
H	-5.321965	-3.497887	-0.058169
H	-4.669712	-3.471458	-1.717217
C	2.988346	-2.952372	0.049902
C	0.881181	-2.942477	1.267584
H	1.251635	-3.961126	1.449071
H	0.886120	-2.410677	2.231139
C	-2.885303	-2.486846	1.036927
H	-3.685850	-2.041138	1.633252
C	-2.152626	-3.650998	-0.930292
H	-2.369414	-4.101532	-1.902639
C	6.339392	-2.085465	-1.454330
H	7.185348	-2.620411	-1.891969
H	0.662831	-2.158617	-2.604259
H	-3.078037	-1.918713	-4.297302
H	1.559715	2.047424	1.950457
H	-1.759206	1.306459	3.193587
H	-2.105110	0.190683	-0.138910
H	0.317677	-0.130249	-1.967153
C	1.345677	0.339760	3.241365
C	2.641536	-0.100527	2.964288
C	0.587347	-0.374352	4.178050
C	3.166268	-1.232663	3.585683
H	3.252092	0.448598	2.246355
C	1.107976	-1.502120	4.809131
H	-0.426925	-0.045976	4.418737
C	2.399144	-1.938714	4.509847
H	4.174297	-1.568901	3.333617
H	0.498467	-2.048885	5.532176
H	2.803090	-2.831962	4.991000

INT1-B
SCF = -2557.41633116
Num. Imaginary Freq = 0

O	0.764504	-2.456314	-1.695666
N	0.703946	-0.225664	-1.453622
C	-0.314244	-1.676439	0.321032
C	1.400590	-0.100704	-2.715074
H	0.715640	-0.164125	-3.579322
H	2.133203	-0.911746	-2.808519
C	0.440588	-1.498437	-1.012689
C	0.020116	0.969380	-0.999647

H	-0.375474	0.788767	0.011033
O	-2.055298	-0.827869	2.342090
N	-0.418077	0.068972	3.653491
C	0.142808	-0.866670	1.488507
C	0.949241	0.420218	3.883495
H	1.014827	1.099682	4.748932
H	1.612435	-0.447520	4.086261
C	-0.817282	-0.567928	2.455675
C	-1.249108	-0.074877	4.815392
H	-2.234275	-0.419809	4.479564
H	-0.844895	-0.812775	5.539776
O	-1.078926	-5.186137	-0.160366
N	-0.434962	-3.132311	0.605144
N	5.338716	-0.909630	-1.005586
H	4.705681	-0.988013	-1.794214
O	5.599381	0.304146	0.902563
C	-4.259088	-1.760680	-0.767215
C	-2.779959	-3.736980	-2.089637
H	-2.171246	-4.484472	-2.601848
C	4.515083	-2.839001	0.234024
C	1.955162	-3.562775	1.148985
C	-4.616804	-2.164653	-2.057736
H	-5.456385	-1.659445	-2.539975
C	2.818462	-2.815878	1.953884
H	2.460688	-2.451122	2.919866
C	-2.429431	-3.351860	-0.795911
C	-3.207209	-2.409278	-0.120117
H	-2.923840	-2.138538	0.902164
C	2.434706	-4.050209	-0.069492
H	1.772197	-4.647222	-0.697377
C	5.185193	0.209764	-0.237689
C	5.723770	-2.176489	-0.385573
H	6.488422	-1.948473	0.368182
H	6.178171	-2.813042	-1.159508
C	-1.239216	-3.974407	-0.112092
C	0.504434	-3.741500	1.536884
H	0.247873	-4.807894	1.572324
H	0.341268	-3.297865	2.530222
C	4.085576	-2.453857	1.508074
H	4.717271	-1.799852	2.112780
C	3.697966	-3.683668	-0.524371
H	4.027538	-4.012006	-1.515233
C	-3.887859	-3.157274	-2.709000
H	-4.165639	-3.460467	-3.721378
O	1.683004	5.181643	-0.220364
N	1.361595	3.455365	1.220752
H	1.409287	2.445743	1.313214
N	-4.286160	0.051383	0.843753
H	-3.436264	-0.344039	1.279606
O	-5.912185	-0.045409	-0.738692
C	4.451793	1.345943	-0.898494
C	3.103648	3.528942	-1.996572
H	2.577405	4.398033	-2.395522
C	-3.465813	2.212327	1.564931
C	-1.002411	3.536824	1.903836
C	4.638458	1.682812	-2.240664
H	5.328258	1.100615	-2.856519
C	-1.662437	3.560773	0.669823
H	-1.201886	4.079018	-0.174709
C	2.886007	3.181927	-0.658023
C	3.581983	2.104231	-0.109746
H	3.481833	1.835742	0.944051
C	-1.611763	2.885950	2.974155
H	-1.108303	2.846098	3.943579
C	-4.919722	-0.525860	-0.203024
C	-4.687156	1.348031	1.348244
H	-5.373928	1.788616	0.611742
H	-5.242105	1.242862	2.297455
C	1.924510	4.038047	0.129837
C	0.375972	4.141112	2.039092
H	0.695766	4.111621	3.093046
H	0.384820	5.189132	1.709497
C	-2.872454	2.898625	0.497958
H	-3.344487	2.882856	-0.488573
C	-2.833918	2.238777	2.806993
H	-3.273692	1.693745	3.644529
C	3.971566	2.781147	-2.784184
H	4.133139	3.054094	-3.829072
H	1.369664	0.950856	3.014248
H	-1.366101	0.886379	5.352452
H	1.912095	0.872425	-2.745301
H	0.760583	1.776275	-0.905357
H	-1.325404	-1.314739	0.104438
H	1.209065	-0.746249	1.661089
C	-1.076885	1.431663	-1.932369
C	-2.197193	0.627803	-2.164107
C	-1.013969	2.687130	-2.541260
C	-3.254331	1.087348	-2.944081
H	-2.257266	-0.363350	-1.709865
C	-2.067426	3.152048	-3.329237
H	-0.137351	3.320534	-2.372688
C	-3.195245	2.356162	-3.523744
H	-4.139046	0.461033	-3.073019
H	-2.008624	4.143475	-3.785210
H	-4.030426	2.722501	-4.125298

TS2-B
SCF = -2557.38947723
Num. Imaginary Freq = 1

O	-3.606784	2.891931	2.133364
N	-2.902061	0.914116	3.009405
C	-2.029608	1.655432	0.810925
C	-3.827224	1.031406	4.118295
H	-3.277324	1.042073	5.075191
H	-4.510379	0.165947	4.128175
C	-2.900981	1.890886	2.064199
C	-2.071780	-0.269424	2.961611
O	-0.623443	0.047958	-0.973456
N	1.356008	0.198684	0.101146
C	-0.635165	1.162125	1.122814
C	2.170150	0.718182	1.171244
H	1.678054	0.588787	2.145289
H	3.121135	0.168193	1.200915
C	0.010159	0.460095	0.013293
C	2.029879	-0.434141	-1.006827
H	2.766040	0.245683	-1.472668
H	2.549409	-1.349942	-0.682220
O	-3.510664	4.151538	-1.296468
N	-2.113361	2.823533	-0.067798
N	4.942530	2.969232	-0.506285
H	4.674953	2.967552	0.470291
O	5.456274	1.552291	-2.212060
C	-4.290846	-0.411584	-1.960163
C	-5.444177	1.858873	-0.793792
H	-5.871392	2.735685	-0.303938
C	2.809571	4.046073	-0.985544
C	0.105537	3.982733	-0.158662
C	-5.650540	-0.401312	-1.635159
H	-6.235705	-1.303003	-1.827190
C	0.538195	3.227584	-1.252159
H	-0.176017	2.579324	-1.763131
C	-4.092546	1.859856	-1.135536
C	-3.533834	0.738654	-1.748249
H	-2.471914	0.758430	-1.992805
C	1.041032	4.786395	0.501406
H	0.721696	5.387614	1.357738
C	5.363760	1.777079	-1.021020

C	4.280936	3.975879	-1.324530
H	4.442548	3.685837	-2.370992
H	4.761486	4.954606	-1.163842
C	-3.231858	3.058600	-0.837055
C	-1.334285	3.983068	0.310110
H	-1.369553	4.104059	1.405308
H	-1.848464	4.855943	-0.118914
C	1.872422	3.249434	-1.651625
H	2.195559	2.623265	-2.487841
C	2.370895	4.824961	0.090053
H	3.083615	5.462515	0.623566
C	-6.221888	0.729597	-1.057338
H	-7.280483	0.726029	-0.787287
O	5.085244	-3.560813	-0.691220
N	3.686905	-3.333573	1.093269
H	3.285150	-2.628298	1.699873
N	-2.335274	-1.786928	-2.312944
H	-1.806125	-1.074990	-1.799867
O	-4.362806	-2.572391	-2.967436
C	5.622901	0.718915	0.020347
C	5.622059	-1.280714	1.969305
H	5.579750	-2.056899	2.737151
C	-0.450854	-3.295341	-1.833498
C	1.680439	-4.071167	-0.155404
C	6.010424	1.013178	1.332727
H	6.293673	2.032534	1.606542
C	0.505296	-3.530418	0.377734
H	0.387205	-3.414655	1.459428
C	5.265543	-1.586888	0.652855
C	5.325296	-0.597871	-0.328533
H	5.052161	-0.828174	-1.359411
C	1.790107	-4.192515	-1.543044
H	2.716772	-4.577071	-1.976350
C	-3.677293	-1.688410	-2.472694
C	-1.610707	-2.955166	-2.741986
H	-2.326930	-3.793036	-2.774876
H	-1.235215	-2.831146	-3.774459
C	4.698570	-2.929134	0.271703
C	2.831548	-4.459111	0.746685
H	3.468981	-5.206321	0.256245
H	2.452617	-4.896488	1.682711
C	-0.546530	-3.143703	-0.446422
H	-1.450000	-2.721410	-0.002360
C	0.734318	-3.808897	-2.367841
H	0.835369	-3.910632	-3.452304
C	6.034859	0.009571	2.297635
H	6.348843	0.240315	3.317718
H	2.401024	1.793563	1.038105
H	1.279807	-0.716852	-1.752404
H	-4.405128	1.958020	4.019138
H	-1.557474	-0.418659	3.924441
H	-2.505410	0.816697	0.293970
H	-0.009387	1.886204	1.655342
C	-2.650514	-1.484608	2.420503
C	-3.675957	-1.487493	1.441370
C	-2.143095	-2.754694	2.797579
C	-4.141355	-2.672282	0.877527
H	-4.117748	-0.540091	1.118955
C	-2.617501	-3.932965	2.235131
H	-1.361341	-2.798140	3.563472
C	-3.620969	-3.909671	1.261742
H	-4.918720	-2.626417	0.110188
H	-2.192671	-4.889211	2.555495
H	-3.982644	-4.833157	0.805309
H	-1.017930	0.289744	1.994673

INT2-B
SCF = -2557.39393688
Num. Imaginary Freq = 0

O	2.247407	-3.135746	1.517465
N	0.335281	-2.065254	2.068350
C	1.679579	-1.448822	-0.029105
C	0.305562	-2.815739	3.317677
H	0.018728	-2.131720	4.129791
H	1.284688	-3.264846	3.522147
C	1.389963	-2.296642	1.251587
C	-0.817301	-1.253747	1.824662
O	2.174993	0.573776	-2.040399
N	-0.023578	0.935167	-2.412830
C	0.587479	-0.562668	-0.611011
C	-1.410595	0.764383	-2.019154
H	-2.024070	1.502574	-2.550530
H	-1.786766	-0.243447	-2.260571
C	1.000464	0.339292	-1.751801
C	0.261167	1.914198	-3.440760
H	1.335987	1.900044	-3.649420
H	-0.295585	1.676468	-4.361226
O	4.053087	-3.772957	-1.371905
N	2.323199	-2.322278	-1.011906
N	-4.334844	-2.947366	0.290910
H	-3.862258	-2.586239	1.114009
O	-5.235700	-2.261785	-1.684159
C	5.310319	0.402694	0.345574
C	5.335226	-2.328778	0.986254
H	5.296585	-3.384640	1.258005
C	-2.442753	-4.079718	-0.793183
C	0.171967	-3.462373	-1.654808
C	6.116836	-0.068682	1.385329
H	6.695078	0.655751	1.962355
C	-0.943533	-3.095776	-2.416835
H	-0.796492	-2.509967	-3.329320
C	4.531966	-1.862434	-0.052411
C	4.569643	-0.514078	-0.399063
H	3.936920	-0.196388	-1.228014
C	-0.034414	-4.225827	-0.503878
H	0.823925	-4.538271	0.093920
C	-4.907330	-2.023986	-0.536200
C	-3.845197	-4.213361	-0.239110
H	-4.548230	-4.522902	-1.023982
H	-3.874712	-4.963520	0.564813
C	3.619187	-2.769784	-0.835198
C	1.542697	-2.968951	-2.058410
H	2.161241	-3.805775	-2.412157
H	1.425153	-2.268455	-2.904351
C	-2.233425	-3.403801	-2.002294
H	-3.095815	-3.073605	-2.585555
C	-1.328901	-4.512677	-0.071143
H	-1.468749	-5.054842	0.868749
C	6.140644	-1.430135	1.687263
H	6.765761	-1.790909	2.507581
O	-3.902117	3.018331	-1.946214
N	-2.909058	3.468051	0.054588
H	-2.722731	3.086959	0.980729
N	4.107749	2.276124	-0.611150
H	3.507190	1.589379	-1.067315
O	5.899969	2.687162	0.717765
C	-5.063167	-0.658517	0.072333
C	-5.157709	1.900659	1.172962
H	-5.175751	2.900677	1.611773
C	2.210117	3.836378	-0.677140
C	-0.590655	4.231591	-0.531796
C	-5.666850	-0.453639	1.315797
H	-6.081006	-1.302008	1.865664
C	0.025527	3.208988	0.193102
H	-0.556282	2.543021	0.835582
C	-4.521661	1.694947	-0.054572
C	-4.537423	0.425961	-0.630443

H	-4.091982	0.268427	-1.613676
C	0.212742	5.051322	-1.332512
H	-0.249019	5.853166	-1.916884
C	5.162336	1.891452	0.156873
C	3.713372	3.653532	-0.750966
H	4.214346	4.216555	0.053204
H	4.079671	4.080361	-1.702452
C	-3.765551	2.792358	-0.755383
C	-2.073423	4.539059	-0.429963
H	-2.462404	4.819775	-1.418712
H	-2.201357	5.413834	0.231957
C	1.405999	3.023266	0.125524
H	1.853534	2.217717	0.713000
C	1.588128	4.852499	-1.410024
H	2.189862	5.493545	-2.061024
C	-5.749167	0.833541	1.843813
H	-6.244187	0.999667	2.802553
H	-1.552934	0.933072	-0.940538
H	-0.023636	2.923310	-3.101846
H	-0.449987	-3.619575	3.260936
H	-1.680135	-1.756292	1.373087
H	2.450087	-0.764722	0.347529
H	-0.302915	-1.140314	-0.870996
C	-0.911105	0.019553	2.374449
C	0.217499	0.715092	2.944434
C	-2.143813	0.763408	2.395619
C	0.099464	1.979337	3.501382
H	1.191274	0.216963	2.949314
C	-2.237555	2.013149	2.987138
H	-3.040272	0.287528	1.991099
C	-1.123442	2.662857	3.543100
H	0.995239	2.453409	3.915607
H	-3.221169	2.494686	3.040044
H	-1.209184	3.650392	3.999867
H	0.242527	0.103506	0.192828

***trans*-TS3-B**
SCF = -2557.32554845
Num. Imaginary Freq = 1

O	1.082366	3.210569	1.278675
N	1.274108	1.004224	1.881368
C	0.266555	1.328846	-0.134169
C	1.714567	1.255856	3.231330
H	0.870286	1.504557	3.900274
H	2.408270	2.108743	3.230335
C	0.959648	2.035275	1.030854
C	0.336133	-0.036104	1.525521
O	-1.276270	-0.210730	-1.792890
N	0.416740	-1.472003	-2.586592
C	0.935792	0.454890	-1.150128
C	1.806781	-1.641317	-2.949502
H	1.980060	-1.326815	-3.995088
H	2.474055	-1.053513	-2.310465
C	-0.073699	-0.432482	-1.852346
C	-0.507649	-2.335364	-3.288928
H	-0.217063	-3.384498	-3.127206
H	-1.518173	-2.172234	-2.900802
O	0.020956	5.384941	-1.127004
N	0.244966	3.120937	-1.590042
N	4.773067	0.037821	1.092195
H	3.963323	0.389623	1.598158
O	5.258741	-1.562898	-0.458441
C	-3.717042	2.628906	-0.132348
C	-2.066013	4.729548	0.695393
H	-1.401512	5.542789	0.993156
C	4.805926	1.916359	-0.450641
C	2.698443	3.073392	-1.912695
C	-4.114935	3.464515	0.915738
H	-5.075326	3.267576	1.396511
C	3.480354	2.052385	-2.464913
H	3.206544	1.640526	-3.441860
C	-1.659804	3.913321	-0.362325
C	-2.502265	2.879320	-0.778793
H	-2.162460	2.273868	-1.622184
C	3.102761	3.627480	-0.694389
H	2.513225	4.443791	-0.274544
C	4.607906	-1.176860	0.495830
C	5.623760	1.058666	0.482358
H	6.427030	0.529485	-0.046354
H	6.072527	1.660579	1.287334
C	-0.346872	4.200250	-1.071372
C	1.347646	3.446416	-2.486857
H	1.334407	4.527985	-2.710599
H	1.220263	2.902662	-3.443558
C	4.516962	1.466633	-1.742547
H	5.047895	0.597618	-2.140292
C	4.135407	3.045113	0.033047
H	4.368828	3.428733	1.031512
C	-3.287066	4.505539	1.330915
H	-3.597507	5.148309	2.159213
O	1.245968	-4.500520	-1.366326
N	-0.187781	-5.033197	0.302239
H	-0.434019	-4.824952	1.259358
N	-4.054393	0.481586	-1.218465
H	-3.054683	0.488546	-1.422413
O	-5.778779	1.429016	-0.094927
C	3.551225	-2.050001	1.122216
C	1.599341	-3.730191	2.193637
H	0.851874	-4.386464	2.644030
C	-3.943845	-1.966174	-1.229429
C	-2.252996	-4.187835	-0.774436
C	3.327391	-2.075575	2.500960
H	3.923915	-1.441008	3.159951
C	-2.192890	-3.053538	0.034925
H	-1.453355	-2.973324	0.830343
C	1.797060	-3.698782	0.807675
C	2.801709	-2.880378	0.289387
H	2.987616	-2.880253	-0.783892
C	-3.205699	-4.214399	-1.796936
H	-3.274518	-5.086328	-2.454576
C	-4.618994	1.476062	-0.481067
C	-4.768700	-0.731788	-1.525362
H	-5.692187	-0.717187	-0.924149
H	-5.081046	-0.753732	-2.585091
C	0.946187	-4.449928	-0.187335
C	-1.294124	-5.351768	-0.574000
H	-0.867070	-5.656417	-1.539359
H	-1.830388	-6.224039	-0.165121
C	-3.031446	-1.966691	-0.175235
H	-2.924455	-1.083958	0.458141
C	-4.029972	-3.113156	-2.025975
H	-4.731082	-3.133161	-2.865672
C	2.363846	-2.927437	3.035135
H	2.199534	-2.959529	4.113779
H	2.079003	-2.703186	-2.854161
H	-0.503751	-2.123344	-4.373791
H	2.219038	0.362725	3.631520
H	0.793469	-1.021049	1.357915
H	-0.794788	1.497404	-0.260899
H	1.396882	1.060054	-1.935435
C	-0.935443	-0.090471	2.274581
C	-1.765239	1.037989	2.432401
C	-1.377997	-1.298354	2.841109
C	-2.990922	0.942062	3.083303
H	-1.468597	2.004140	2.014826
C	-2.611231	-1.397151	3.483035
H	-0.738541	-2.183961	2.784560

C	-3.430739	-0.277208	3.602236
H	-3.619181	1.831564	3.159854
H	-2.933051	-2.358382	3.892944
H	-4.404045	-0.349954	4.092301
H	1.749026	-0.127823	-0.695246

***cis*-TS3-B**
SCF = -2557.33145669
Num. Imaginary Freq = 1

O	-4.348782	2.257932	2.014126
N	-3.715193	0.187035	2.795825
C	-2.643103	0.808275	0.909606
C	-4.642831	0.034460	3.884891
H	-5.359047	-0.780904	3.683691
H	-5.196561	0.977068	4.001423
C	-3.695496	1.239849	1.943223
C	-2.802336	-0.793845	2.302775
O	-0.663199	-0.790243	-0.256614
N	1.046887	0.205995	0.797380
C	-1.190567	1.115362	1.129599
C	1.582845	1.289362	1.595685
H	1.365344	1.156670	2.669640
H	2.673028	1.316996	1.461412
C	-0.270664	0.100369	0.491589
C	1.920742	-0.866017	0.370988
H	2.234785	-0.754900	-0.680527
H	2.813915	-0.867747	1.010091
O	-4.336538	3.070698	-2.171040
N	-2.970473	2.228286	-0.524253
N	3.778810	3.980379	-0.456171
H	3.546438	3.715955	0.493441
O	4.425721	3.086701	-2.446512
C	-4.086371	-1.670375	-2.173690
C	-5.664251	0.623792	-2.324653
H	-6.257365	1.538515	-2.377620
C	1.485381	4.732242	-0.872351
C	-1.211234	3.995133	-0.430535
C	-5.420675	-1.761667	-2.578702
H	-5.822437	-2.749908	-2.809120
C	-0.457558	3.395431	-1.448194
H	-0.927125	2.611308	-2.046266
C	-4.332924	0.736170	-1.907627
C	-3.538253	-0.413451	-1.884020
H	-2.480863	-0.318206	-1.629816
C	-0.595553	4.980966	0.347669
H	-1.164799	5.455403	1.152420
C	4.363576	3.024446	-1.233960
C	2.956705	5.033459	-1.034887
H	3.237439	5.096744	-2.094457
H	3.208185	5.993345	-0.556841
C	-3.852026	2.127582	-1.533439
C	-2.642057	3.581685	-0.146959
H	-2.847361	3.723124	0.927228
H	-3.309002	4.275051	-0.689776
C	0.869303	3.753719	-1.663785
H	1.448099	3.260890	-2.450535
C	0.732003	5.346597	0.130433
H	1.193595	6.113506	0.761078
C	-6.202671	-0.613282	-2.665323
H	-7.245259	-0.686017	-2.985384
O	5.138442	-1.972384	-2.342880
N	4.727808	-2.907720	-0.311301
H	4.420135	-2.671552	0.624239
N	-2.236681	-2.933810	-1.207574
H	-1.893669	-2.061958	-0.797992
O	-3.792498	-4.018252	-2.443007
C	4.910367	1.842547	-0.476282
C	5.764788	-0.452512	0.867756
H	6.102633	-1.347510	1.395917
C	-0.104843	-4.128592	-0.788619
C	2.715735	-4.205574	-0.858158
C	5.414543	1.934923	0.825745
H	5.481582	2.907046	1.320323
C	2.011712	-4.819309	0.178914
H	2.556980	-5.332281	0.977348
C	5.261660	-0.552234	-0.433429
C	4.876103	0.602680	-1.113012
H	4.506505	0.531506	-2.137451
C	1.991562	-3.544893	-1.859418
H	2.534207	-3.042044	-2.664534
C	-3.353773	-2.972682	-1.983978
C	-1.614539	-4.153715	-0.765297
H	-1.949939	-4.414222	0.255920
H	-1.986282	-4.954124	-1.424218
C	5.053542	-1.867759	-1.134495
C	4.226935	-4.163495	-0.847755
H	4.629730	-4.251059	-1.865071
H	4.629877	-4.993275	-0.246690
C	0.618484	-4.778239	0.213499
H	0.083441	-5.253333	1.040696
C	0.602158	-3.504933	-1.822741
H	0.047001	-2.966601	-2.593447
C	5.856798	0.791768	1.487934
H	6.271367	0.870374	2.494984
H	1.183456	2.261370	1.267027
H	1.404804	-1.830746	0.467369
H	-4.113122	-0.197100	4.823551
H	-3.259151	-1.624693	1.744345
H	-2.918644	0.081907	0.162609
H	-0.920805	2.100846	0.736508
C	-1.620700	-1.126934	3.056162
C	-0.802184	-2.214027	2.654099
C	-1.129831	-0.327523	4.117964
C	0.431693	-2.453096	3.237732
H	-1.139673	-2.845095	1.828599
C	0.107996	-0.581139	4.707261
H	-1.734951	0.512984	4.468898
C	0.909648	-1.635948	4.270869
H	1.038742	-3.287108	2.872688
H	0.452207	0.062801	5.522397
H	1.881406	-1.827858	4.731921
H	-0.961147	1.160601	2.205540

Th
SCF = -802.852059213
Num. Imaginary Freq = 0

O	2.535783	-2.317413	0.877388
N	2.975297	-0.397445	-0.249519
C	0.783358	-1.439718	-0.469689
C	4.338221	-0.403230	0.213652
H	5.024076	-0.122466	-0.605037
H	4.583910	-1.407761	0.577435
C	2.148005	-1.438839	0.118389
C	2.500614	0.848862	-0.808896
H	1.405928	0.869275	-0.867697
H	2.919363	1.031666	-1.815466
O	-1.997224	-2.575929	-1.218909
N	-2.505444	-1.032971	0.405757
C	-0.274402	-1.806631	0.267412
C	-1.904769	-0.017434	1.207434
H	-2.041245	-0.084139	2.291638
C	-1.674588	-1.865812	-0.275773
C	-3.902531	-0.961342	0.045478
H	-4.524220	-0.938858	0.955537
H	-4.114339	-0.044148	-0.532583
H	-4.165850	-1.835183	-0.567261

H	4.490573	0.313812	1.042813
H	2.793093	1.691712	-0.159476
H	0.633064	-1.074984	-1.489415
H	-0.124223	-2.120609	1.304612
C	-1.319374	1.109239	0.617594
C	-0.686831	2.134716	1.400677
C	-1.254454	1.314696	-0.804204
C	-0.083006	3.237719	0.825434
H	-0.684313	2.026094	2.490217
C	-0.656002	2.439210	-1.358460
H	-1.712131	0.576227	-1.468465
C	-0.053515	3.423543	-0.567994
H	0.384443	3.983460	1.478755
H	-0.650915	2.544960	-2.449325
H	0.420982	4.300704	-1.014111

TS*cis*
SCF = -802.855833232
Num. Imaginary Freq = 1

O	2.183600	-1.949976	1.334081
N	2.979467	-0.603806	-0.316972
C	0.653343	-1.296686	-0.357356
C	4.322060	-0.765176	0.161629
H	5.009656	-1.094892	-0.642426
H	4.304303	-1.519711	0.957805
C	1.946175	-1.325133	0.296728
C	2.756708	0.384545	-1.339134
H	1.758159	0.832810	-1.236062
H	2.855090	-0.016175	-2.368685
O	-2.144601	-2.668203	-1.166753
N	-2.597410	-0.892408	0.245622
C	-0.485069	-1.629454	0.352970
C	-1.747198	-0.099638	1.072851
H	-1.929430	-0.164924	2.154910
C	-1.823120	-1.857605	-0.319539
C	-3.977525	-0.652085	-0.060974
H	-4.608091	-0.711859	0.843895
H	-4.119941	0.346738	-0.510012
H	-4.305569	-1.418166	-0.779963
H	4.728417	0.177014	0.580318
H	3.494869	1.197564	-1.229401
H	0.552131	-0.904481	-1.369882
H	-0.327515	-2.147982	1.306390
C	-1.201604	1.129837	0.579033
C	-0.521052	2.024167	1.443841
C	-1.161309	1.441994	-0.803317
C	0.126166	3.154131	0.966250
H	-0.502027	1.801965	2.515047
C	-0.519069	2.585233	-1.274002
H	-1.654787	0.771615	-1.511754
C	0.130810	3.458239	-0.401622
H	0.641658	3.814057	1.671157
H	-0.519193	2.791152	-2.349162
H	0.639973	4.349986	-0.775494

TS*trans*
SCF = -802.844937379
Num. Imaginary Freq = 1

O	-2.161086	-1.511063	-1.092564
N	-3.914061	-0.825802	0.180243
C	-1.929619	0.580910	-0.006895
C	-4.722503	-1.897903	-0.332275
H	-5.156167	-2.503613	0.485327
H	-4.075999	-2.534557	-0.948271
C	-2.626550	-0.643293	-0.353806
C	-4.568305	0.142159	1.018055
H	-5.416905	-0.339310	1.530202
H	-4.964575	1.015040	0.460815
O	-0.441461	3.214830	-0.769425
N	1.205684	1.931630	0.222750
C	-0.632692	0.786764	-0.425487
C	1.172611	0.635218	0.812414
H	0.753431	0.576376	1.825415
C	0.011924	2.150252	-0.388078
C	2.172519	2.959694	0.493989
H	3.191388	2.598666	0.278717
H	1.946350	3.828808	-0.141396
H	2.139709	3.282710	1.550848
H	-5.560626	-1.530736	-0.959247
H	-3.890859	0.521157	1.796754
H	-2.414860	1.359200	0.582921
H	-0.219722	0.071476	-1.145542
C	2.171755	-0.325884	0.449899
C	2.396618	-1.494314	1.225395
C	2.947907	-0.197970	-0.732623
C	3.325038	-2.453115	0.847746
H	1.813191	-1.633829	2.140368
C	3.879661	-1.162787	-1.099495
H	2.790631	0.678056	-1.367724
C	4.086294	-2.302445	-0.318271
H	3.463074	-3.339956	1.474742
H	4.454905	-1.024968	-2.020821
H	4.818106	-3.059173	-0.611566

TSrot
SCF = -802.844216882
Num. Imaginary Freq = 1

O	2.974592	-0.784028	-1.772592
C	3.036562	-0.340764	-0.638930
N	3.983839	-0.797425	0.258328
C	4.952270	-1.768203	-0.184613
H	4.785119	-2.751453	0.292265
H	4.854012	-1.884326	-1.270040
H	5.976875	-1.438644	0.063295
C	3.987810	-0.497077	1.668703
H	3.092858	0.066285	1.953751
H	4.879677	0.087161	1.961358
H	3.990794	-1.432222	2.256275
C	2.131129	0.736581	-0.148025
C	0.848978	0.815645	-0.511388
C	0.010434	1.944949	0.022348
O	0.525478	2.826565	0.709810
N	-1.307579	1.901538	-0.298775
C	-1.928733	0.868126	-1.074064
C	-2.562423	-0.206261	-0.445177
C	-2.475683	-0.461410	0.968893
C	-3.104318	-1.547782	1.560243
C	-3.860721	-2.464509	0.819591
C	-3.962146	-2.241018	-0.564429
C	-3.348837	-1.164574	-1.177897
H	-3.454464	-1.028031	-2.259558
H	-4.544938	-2.935851	-1.181075
H	-4.349448	-3.317946	1.295941
H	-2.998589	-1.687249	2.642878
H	-1.887158	0.219937	1.590737
H	-2.107936	1.096249	-2.129921
C	-2.167757	2.945276	0.227726
H	-2.977207	2.487429	0.819524
H	-1.585991	3.631888	0.856479
H	-2.630721	3.506667	-0.601544
H	0.373321	0.084400	-1.168060
H	2.510015	1.512762	0.525182

***cis*-Lac**
SCF = -802.862100806
Num. Imaginary Freq = 0

O	1.888862	0.956323	-1.641304
C	1.873386	1.057490	-0.384380
C	0.722081	1.295362	0.370704
C	-0.532704	1.485677	-0.379338
C	-1.814167	1.990065	0.288134
O	-2.177681	2.963856	0.904267
N	-2.548946	0.899193	-0.128861
C	-3.968037	0.784254	-0.262549
H	-4.421677	1.619017	0.293021
H	-4.358092	-0.162822	0.147200
H	-4.286391	0.851604	-1.320084
C	-1.457822	0.255929	-0.841890
C	-1.036993	-1.107715	-0.383566
C	0.166296	-1.627601	-0.884754
C	0.604294	-2.887180	-0.486628
C	-0.138649	-3.642870	0.424389
C	-1.317512	-3.117823	0.948643
C	-1.758176	-1.853256	0.550470
H	-2.667735	-1.432732	0.985196
H	-1.897412	-3.690573	1.677981
H	0.211441	-4.630542	0.737320
H	1.549881	-3.274903	-0.875368
H	0.771455	-0.999269	-1.550225
H	-1.609553	0.249186	-1.937830
H	-0.336776	2.086635	-1.285643
H	0.680337	1.204073	1.455171
N	3.127864	0.904244	0.288099
C	3.180782	0.891632	1.718228
H	2.632071	1.752891	2.127883
H	4.230208	0.969157	2.053314
H	2.748482	-0.027970	2.174661
C	4.120007	0.110406	-0.377509
H	5.140727	0.477046	-0.157259
H	4.085265	-0.966025	-0.087869
H	3.929182	0.183516	-1.455567

***trans*-Lac**
SCF = -802.863999747
Num. Imaginary Freq = 0

O	-2.118572	0.501104	1.026776
C	-2.317967	-0.334042	0.105760
C	-1.452097	-0.523550	-0.974909
C	-0.220965	0.297017	-1.014489
C	-0.284704	1.833802	-0.832481
O	-0.885326	2.763007	-1.307965
N	0.673841	1.818860	0.167668
C	0.781329	2.764441	1.239315
H	0.458256	3.745257	0.860167
H	1.818647	2.847631	1.605241
H	0.121616	2.483033	2.079971
C	0.699298	0.368933	0.280206
C	2.036083	-0.296373	0.208021
C	2.141827	-1.662370	0.509206
C	3.359828	-2.327549	0.392252
C	4.502728	-1.638442	-0.019190
C	4.408436	-0.279458	-0.320616
C	3.186061	0.382811	-0.212890
H	3.099185	1.444296	-0.458555
H	5.296374	0.270283	-0.645841
H	5.460625	-2.158391	-0.104304
H	3.420394	-3.394041	0.626692
H	1.243146	-2.198632	0.825791
H	0.102671	0.019804	1.138081
H	0.391040	0.060892	-1.901920
H	-1.658413	-1.193958	-1.806722
N	-3.491457	-1.147240	0.216438
C	-3.857115	-2.037153	-0.842349
H	-4.199430	-1.520578	-1.767796
H	-4.675213	-2.698153	-0.506608
H	-3.004737	-2.676596	-1.119580
C	-4.573292	-0.620047	0.997265
H	-5.109330	-1.424679	1.536562
H	-4.140277	0.081781	1.721298
H	-5.326749	-0.070293	0.387687

22. References

[1] Martinez-Cuezva, A.; Lopez-Leonardo, C.; Bautista, D.; Alajarin, M.; Berna, J., *Stereocontrolled synthesis of β-lactams within [2]rotaxanes: showcasing the chemical consequences of the mechanical bond. J. Am. Chem. Soc.* **2016,** *138*, 8726-8729.

[2] Carlone, A.; Goldup, S. M.; Lebrasseur, N.; Leigh, D. A.; Wilson, A., *A three-compartment chemically-driven molecular information ratchet. J. Am. Chem. Soc.* **2012,** *134*, 8321-8323.

[3] Martinez-Cuezva, A.; Morales, F.; Marley, G. R.; Lopez-Lopez, A.; Martinez-Costa, J. C.; Bautista, D.; Alajarin, M.; Berna, J., *Thermally and photochemically induced dethreading of fumaramide-based kinetically stable pseudo[2]rotaxanes*. *Eur. J. Org. Chem.* **2019**, 3480-3488.

[4] Sheldrick, G. M. *"Crystal structure refinement with SHELXL*", *Acta Cryst.*, **2015**, C71, 3-8.

[5] Chai, J.-D.; Head-Gordon, M. *Long-Range Corrected Hybrid Density Functionals with Damped Atom–Atom Dispersion Corrections*. *Phys. Chem. Chem. Phys*. **2008**, *10* (44), 6615. https://doi.org/10.1039/b810189b.
[6] Weigend, F. *Accurate Coulomb-Fitting Basis Sets for H to Rn*. *Phys. Chem. Chem. Phys*. **2006**, *8* (9), 1057. https://doi.org/10.1039/b515623h
[7] Bauernschmitt, R.; Ahlrichs, R. *Stability Analysis for Solutions of the Closed Shell Kohn–Sham Equation*. *J. Chem. Phys*. **1996**, *104* (22), 9047–9052. https://doi.org/10.1063/1.471637.
[8] Riplinger, C.; Neese, F. *An Efficient and near Linear Scaling Pair Natural Orbital Based Local Coupled Cluster Method. J. Chem. Phys*. **2013**, *138* (3), 034106. https://doi.org/10.1063/1.4773581.
[9] Zheng, J.; Xu, X.; Truhlar, D. G. *Minimally Augmented Karlsruhe Basis Sets. Theor. Chem. Acc*. **2011**, *128* (3), 295–305. https://doi.org/10.1007/s00214-010-0846-z.
[10] Marenich, A. V.; Cramer, C. J.; Truhlar, D. G. *Universal Solvation Model Based on Solute Electron Density and on a Continuum Model of the Solvent Defined by the Bulk Dielectric Constant and Atomic Surface Tensions. J. Phys. Chem. B* **2009**, *113* (18), 6378–6396. https://doi.org/10.1021/jp810292n.
[11] Frisch, M. J.; Trucks, G. W.; Schlegel, H. B.; Scuseria, G. E.; Robb, M. A.; Cheeseman, J. R.; Scalmani, G.; Barone, V.; Petersson, G. A.; Nakatsuji, H.; et al. *Gaussian 09 Version D.01*; Gaussian, Inc.: Wallingford CT, 2009
[12] Neese, F. *The ORCA Program System. Wiley Interdiscip. Rev. Comput. Mol. Sci*. **2012**, *2* (1), 73–78. https://doi.org/10.1002/wcms.81.
